# A set of *Saccharomyces cerevisiae* integration vectors for fluorescent dye labeling of proteins

**DOI:** 10.1093/g3journal/jkac201

**Published:** 2022-08-09

**Authors:** Inwha Baek, Sarah N Le, Jongcheol Jeon, Yujin Chun, Charlotte Reed, Stephen Buratowski

**Affiliations:** Department of Biological Chemistry and Molecular Pharmacology, Harvard Medical School, Boston, MA 02115, USA; Department of Biological Chemistry and Molecular Pharmacology, Harvard Medical School, Boston, MA 02115, USA; Department of Biological Chemistry and Molecular Pharmacology, Harvard Medical School, Boston, MA 02115, USA; Department of Biological Chemistry and Molecular Pharmacology, Harvard Medical School, Boston, MA 02115, USA; Department of Biological Chemistry and Molecular Pharmacology, Harvard Medical School, Boston, MA 02115, USA; Department of Biological Chemistry and Molecular Pharmacology, Harvard Medical School, Boston, MA 02115, USA

**Keywords:** Saccharomyces cerevisiae, fluorescence microscopy, integration vector, epitope tagging, SNAP, CLIP, HaloTag7, DHFR

## Abstract

Protein fusions are frequently used for fluorescence imaging of individual molecules, both in vivo and in vitro. The SNAP, CLIP, HALO (aka HaloTag7), and DHFR protein tags can be linked to small molecule dyes that provide brightness and photo-stability superior to fluorescent proteins. To facilitate fluorescent dye tagging of proteins in the yeast *Saccharomyces cerevisiae*, we constructed a modular set of vectors with various combinations of labeling protein tags and selectable markers. These vectors can be used in combination to create strains where multiple proteins labeled with different colored dyes can be simultaneously observed.

## Introduction

Fluorescence microscopy is a powerful tool for monitoring protein localization and dynamics. In recent years, it has become feasible to track the movement of individual protein molecules, both in vivo (Zhang *et al.* 2014; Liu and Tjian 2018; Lionnet and Wu 2021) and in vitro (Friedman and Gelles 2015). These single-molecule studies require particularly bright and stable fluorescent labels. However, the fluorescence quantum yield and photo-stability of green fluorescent protein (GFP) and other fluorescent proteins are insufficient for many single-molecule imaging applications. To overcome these limitations, several labeling tags have been developed that efficiently, and often covalently, bind small molecule substrates that can be conjugated to bright and stable fluorescent organic dyes (Keppler *et al.* 2003; Miller *et al.* 2005; Gautier *et al.* 2008; Los *et al.* 2008; Wang *et al.* 2014; Grimm *et al.* 2017).

Here, we describe a set of plasmids for creating protein fusions in the yeast *Saccharomyces cerevisiae* that are amenable to fluorescent labeling in vivo or in vitro. Inspired by tagging vectors created by Hoskins and colleagues (Hoskins *et al.* 2011; Shcherbakova *et al.* 2013), we used a modular strategy to create a wider choice of labeling tags and selectable markers. The new vectors also incorporate an optional epitope tag that can be used for verification and quantitation of protein levels by immunoblotting. Tagging cassettes can be combined within a single yeast strain to label multiple proteins of interest (POIs) for multicolor fluorescence microscopy. We have used these vectors for single-molecule Total Internal Reflection Fluorescence (TIRF) microscopy of in vitro transcription reactions (Rosen *et al.* 2020; Baek *et al.* 2021), but they should also be useful for other types of fluorescence microscopy.

## Materials and methods

### Plasmid construction

All C-terminal tagging cassette plasmids (Table 1, Supplementary Data) were constructed using isothermal assembly (Gibson *et al.* 2009) of four DNA fragments: (1) pBlueScript II SK(+) digested with EcoRI and BamHI, (2) a 176-bp HA3-linker fragment generated by PCR using oligos 3XHA-Forward (O #3908) and 3XHA-Reverse (O #3909) on template plasmid pFA6a-HA-KlURA3 (Sung *et al.* 2008), (3) a PCR-amplified fragment encoding one of the 4 fluorescence-tagging fusion proteins, preceded by an in frame GSGGSGS linker and followed by a 39-bp synthetic transcription terminator (5′-TATATAACTGTCTAGAAATAAAGAGTATCATCTTTCAAA-3′), and (4) a cassette expressing one of 5 MX selection marker genes that was PCR amplified using oligonucleotides complementary to the common TEF promoter [TEFpro-Forward (O #3914)] and TEF terminator [TEFterm-Reverse (O #3915)] sequences.

**Table 1.** Plasmids generated in this study.

Plasmid name	N or C terminal	Fusion	Selectable marker	Catalog number	Freezer number
Yeast tagging plasmids					
pBS-SKII-3XHA-fSNAP-Hygromycin	C	fSNAP	Hygromycin	YV307	4526
pBS-SKII-3XHA-fSNAP-Phleomycin	C	fSNAP	Phleomycin	YV308	4527
pBS-SKII-3XHA-fSNAP-NAT	C	fSNAP	Nourseothricin	YV309	4528
pBS-SKII-3XHA-fSNAP-URA	C	fSNAP	*C. albicans* *URA3*	YV310	4529
pBS-SKII-3XHA-fSNAP-Kan	C	fSNAP	G418/Kanamycin	YV311	4530
pBS-SKII-3XHA-fCLIP-Hygromycin	C	fCLIP	Hygromycin	YV312	4531
pBS-SKII-3XHA-fCLIP-Phleomycin	C	fCLIP	Phleomycin	YV313	4532
pBS-SKII-3XHA-fCLIP-NAT	C	fCLIP	Nourseothricin	YV314	4533
pBS-SKII-3XHA-fCLIP-URA	C	fCLIP	*C. albicans* *URA3*	YV315	4534
pBS-SKII-3XHA-fCLIP-Kan	C	fCLIP	G418/Kanamycin	YV316	4535
pBS-SKII-3XHA-eDHFR-Hygromycin	C	eDHFR	Hygromycin	YV317	4536
pBS-SKII-3XHA-eDHFR-Phleomycin	C	eDHFR	Phleomycin	YV318	4537
pBS-SKII-3XHA-eDHFR-NAT	C	eDHFR	Nourseothricin	YV319	4538
pBS-SKII-3XHA-eDHFR-URA	C	eDHFR	*C. albicans* *URA3*	YV320	4539
pBS-SKII-3XHA-eDHFR-Kan	C	eDHFR	G418/Kanamycin	YV321	4540
pBS-SKII-3XHA-HALO-Hygromycin	C	HALO	Hygromycin	YV328	4640
pBS-SKII-3XHA-HALO-Phleomycin	C	HALO	Phleomycin	YV329	4641
pBS-SKII-3XHA-HALO-NAT	C	HALO	Nourseothricin	YV330	4642
pBS-SKII-3XHA-HALO-URA	C	HALO	*C. albicans* *URA3*	YV331	4643
pBS-SKII-3XHA-HALO-Kan	C	HALO	G418/Kanamycin	YV332	4644
pBS-SKII-URA-GAL1-DHFR-3xHA	N	eDHFR	*C. albicans* *URA3*	YV333	4670
pBS-SKII-URA-GAL1-fSNAP-3xHA	N	fSNAP	*C. albicans* *URA3*	YV334	4671
pBS-SKII-URA-GAL1-fCLIP-3xHA	N	fCLIP	*C. albicans* *URA3*	YV335	4672
pBS-SKII-URA-GAL1-HALO-3xHA	N	HALO	*C. albicans* *URA3*	YV357	4712
pBS-SKII-URA-GAL1-fSNAP-GS	N	fSNAP	*C. albicans* *URA3*	YV358	4713
pBS-SKII-URA-GAL1-fCLIP-GS	N	fCLIP	*C. albicans* *URA3*	YV359	4714
pBS-SKII-URA-GAL1-HALO-GS	N	HALO	*C. albicans* *URA3*	YV360	4715
Bacterial expression constructs					
pET28-his6-HA3-SNAP		SNAP	*KanR*	BE570	4633
pET28-his6-HA3-CLIP		CLIP	*KanR*	BE571	4639
pET28-his6-HA3-HALO		HALO	*KanR*	BE572	4645

The four fusion proteins were HALO/HaloTag7 (from pTG343-H10-SUMO-HALO-XLF; Graham *et al.* 2018)), fast SNAP, fast CLIP, and DHFR (from pAAH13, pAAH12, and pAAH2, respectively; Hoskins *et al.* 2011). The 5 markers chosen are those encoding the *Candida albicansURA3* homolog (from pAG61; Goldstein *et al.* 1999), Kanamycin/G418 resistance (Bahler *et al.* 1998), Nourseothricin resistance (pAG25), Bleomycin/Phleomycin resistance (pUG66), or Hygromycin resistance (pAG32; Goldstein and McCusker 1999). The HA3, GSGGSGS linker and TEF promoter/terminator sequences are common for all plasmids, providing complementary overlaps to the adjacent fragments for Gibson assembly. The correct plasmids were confirmed by colony PCR with oligos 3XHA-Forward (O #3908) and TEFterm-Reverse (O #3915), and by sequencing with M13 forward and reverse primers.

N-terminal tagging cassettes (Table 1, see Supplementary Data for maps and sequences) were also created by isothermal assembly. The plasmid pBS-SKII-URA-GAL1-DHFR-3xHA was constructed first, in a reaction combining 2 DNA fragments: (1) the GAL1 promoter, amplified from yeast genomic DNA using primers GAL1pro-fwd (O #4201) and GAL1pro-rev (O #4202), and (2) a pBlueScript II SK(+) plasmid backbone carrying the *C. albicansURA3* MX cassette and a DHFR-HA3-GSGGSGS cassette [amplified from template pBS-URA3-DST1pro-DHFR-3xHA (YV324, the gift of Karina Herlambang, Brandeis University) using primers GAL1pro-eDHFR-fwd (O #4203) and GAL1pro-TEFt-rev (O #4204)]. In a set of subsequent reactions, the other plasmids were derived from pBS-SKII-URA-GAL1-DHFR-3xHA by replacing the DHFR sequence with coding regions for SNAP, CLIP, or HALO. A backbone from pBS-SKII-URA-GAL1-DHFR-3xHA lacking the DHFR coding region was amplified using primers SNAP/CLIP-3xHA-fwd (O #4205) and GAL1pro-rev (O #4202) for the SNAP and CLIP derivatives, or primers HALO-3xHA-fwd (O #4266) and GAL1pro-rev (O #4202) for the HALO derivative. The appropriate backbone was combined by Gibson assembly with PCR product inserts containing the open reading frame (ORF) for SNAP [amplified with primers GAL1pro-SNAP/CLIP-fwd (O #4206) and SNAP/CLIP-rev (O #4207) on template pBS-URA3-DST1pro-SNAP-3xHA (YV323)], CLIP [amplified with primers GAL1pro-SNAP/CLIP-fwd (O #4206) and SNAP/CLIP-rev (O #4207) from template pBS-SKII-3XHA-fCLIP-URA (YV315)], or HALO [amplified with primers GAL1pro-HALO-fwd (O #4265) and HALO-rev (O #4259) from template pTG-343-H10-SUMO-HALO-XLF (F1232)]. Finally, these plasmids were used to create derivatives lacking the HA3 tag by inverse PCR with primers flanking the HA3 cassette [GS-Linker-F (O#4211) together with SNAP/CLIP-rev (O#4207) or HALO-rev (O#4259)], followed by re-ligation. Plasmid sequences were confirmed by sequencing.

Plasmids for expressing hexa-histidine (his6) and triple influenza hemagglutinin (HA3)-tagged fluorescent tag proteins in *Escherichia coli* (Table 1) were constructed by Gibson assembly. For SNAP and CLIP proteins, the plasmid pET-28a was linearized with BamHI. SNAP or CLIP coding sequence was amplified using oligos T7 tag—HA (O #4111) and polylinker—SNAP 3' (O #4112) from the C-terminal tagging cassettes. For HALO, pET-28a was digested with BamHI and HindIII, and HALO coding sequence amplified using oligos T7 tag—HA (O #4111) and polylinker—HALO (O #4131). Vector and insert were ligated using Gibson isothermal assembly, and plasmids confirmed by sequencing.

All oligonucleotides were ordered from Integrated DNA Technology and are listed in Supplementary Table 1. Integration plasmids are listed in Table 1 and Supplementary Table 3, with maps and sequences in Supplementary Data.

### PCR amplification and transformation of tagging cassettes

**Fig. 1.**
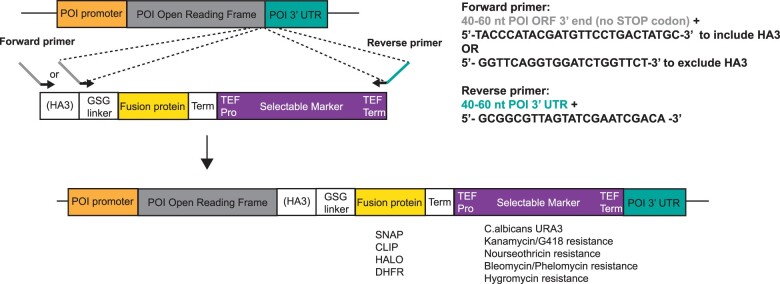
Schematic of C-terminal tagging strategy. Using one of the C-terminal tagging plasmids listed in Table 1 as template, an integration cassette is generated by PCR for transformation into yeast. The upstream primer should have 40–60 nt of complementarity to the 3′ end of the POI open reading frame (gray), up to but not including the stop codon, followed by the indicated sequence that primes either within the HA3 or GSGGSGS linker cassette. The downstream primer should be complementary to the POI 3′ untranslated region (POI 3′ UTR, cyan), followed by sequence that primes within the TEF terminator downstream of the selectable marker module. Transformants are selected on the appropriate media and then tested for the proper integration and fusion protein expression (GSG linker: GSGGSGS, Term: synthetic transcription terminator, TEF Pro: TEF promoter, and TEF Term: TEF transcription terminator).

Oligonucleotides were designed with 40–60 nucleotides (nt) of complementarity to the target gene fused to the appropriate tagging cassette sequences (see Figs. 1 and 3). Integration cassettes were PCR amplified using either of the following conditions.

For C-terminal tagging, Platinum Taq DNA polymerase (ThermoFisher, cat#10966026) was most often used for PCR amplification of the integration cassette. Hot start amplification was initiated with denaturation at 94°C for 2 min, followed by 34 PCR cycles of 94°C for 30 s, 55°C for 30 s, and 72°C for 2 min 30 s, and terminated with a final extension step at 72°C for 10 min. When using Platinum Taq, PCR amplification reactions of plasmids containing the nourseothricin resistance gene (*NatR*) were supplemented with 4% DMSO because of the GC-rich character of NatR.

For both N-terminal and C-terminal tagging cassettes, Phusion High-Fidelity DNA Polymerase (New England Biolabs, cat#M0530) can also be used. After an initial 2-min denaturation at 90°C, 30–34 cycles consisting of 10-s denaturation at 90°C, 30-s annealing at 55°C, and 2 min 30 s extension at 72°C were carried out, followed by a final extension step at 72°C for 10 min.

Genomic integration of fragments with short homology arms is relatively inefficient, so larger amounts of DNA were produced for each transformation. For 30-cycle PCR reactions, multiple reactions were combined to a total of 500 µL, and the DNA was concentrated by ethanol precipitation or PCR clean up column kit (EZNA Cycle Pure, Omega BioTek, catalog #D6492.02). Alternatively, 50-µL reactions were run for 34 cycles and the PCR reactions were directly used for transformation. *Saccharomyces cerevisiae* strains (Supplementary Table 2) were transformed using a version of the standard lithium acetate protocol (Gietz *et al.* 1995). Specifically, overnight precultured yeast cells were diluted into 30 mL of YPD medium at OD600 of 0.3 and grown until the OD600 reached 1.0 at 30°C. Cells were pelleted at 2500 rpm for 3 min at RT and washed with 1X TE buffer (10 mM Tris-Cl, pH 7.5 and 1 mM EDTA). Cell pellets were washed with 1X LiAc-TE buffer (100 mM lithium acetate in 1X TE) and gently resuspended with 500 µL of 1X LiAc-TE buffer, producing 5 aliquots of competent cells (100 µL of competent cells per microfuge tube). Ten microliters of 10 mg/mL denatured salmon testes DNA (Sigma D1626), the PCR-amplified integration cassette, and 350 µL of 40% PEG in 1X LiAc-TE buffer were added serially to the 100 µL of competent cells, followed by a 30-min incubation at 30°C. After adding DMSO (10% v/v), competent cells were incubated at 42°C for 15 min for heat shock, and immediately placed on ice for 30 s before pelleting with a brief microfuge spin.

For the selection of integrants, cells were gently resuspended in 100–200 µL of 1X TE and first plated on nonselective YPD plates. On the next day, the lawn of cells from the YPD plate was replica plated onto the selection media. Selection plates for Kanamycin/G418 resistance contained 500 µg/mL of G418 (Teknova G5005), while those for hygromycin resistance contained 200 µg/mL of Hygromycin B (Gold Biotechnology H-270-EZ25). Selection plates for nourseothricin resistance contained 100 µg/mL of clonNAT (Werner Bioagents 500300), while those for bleomycin/phleomycin resistance contained 7.5 µg/mL of phleomycin. Selection plates for the *URA3* marker were synthetic complete media lacking uracil. Colonies were typically observed 2–4 days after replica plating. When creating strains with multiple tags, it is important to screen against colonies in which an integration cassette recombined into a previously integrated marker module, rather than the POI gene. Therefore, selections for all expected markers were performed, either in parallel or sequentially. Yeast colonies with the proper markers were validated for proper integration by colony PCR, or genomic DNA PCR followed by sequencing.

### Analysis of POI fusion expression and dye labeling

The integrity, levels, and labeling of tagged POIs can be examined by SDS-polyacrylamide gel electrophoresis (PAGE), immunoblotting, and (for SNAP, CLIP, and HALO) in-gel fluorescence imaging. For these analyses, extracts appropriate for the specific POI should be used. We have successfully used both whole-cell lysates and nuclear extracts for analyzing various transcription factors. Nuclear extracts were prepared as previously described (Sikorski *et al.* 2012). For the preparation of soluble whole-cell lysates, yeast strains of interest were grown to OD600 of 1.0 in YPD medium. Cells were harvested by centrifugation, washed once with ice cold water, resuspended in 1 pellet volume of lysis buffer [50 mM Tris-HCl, pH 8.0 or HEPES pH 7.6, 150–300 mM sodium chloride, 0.1% Nonidet-P40 (optional), 1 mM dithiothreitol (optional), 20% glycerol (optional), and protease inhibitors)], and lysed by vortexing with an equal volume of glass beads (425–600 µm, Sigma G8772). After spinning out cell debris and beads, the protein concentration of the soluble supernatant was determined using the Pierce Coomassie protein assay reagent (VWR PI23200).

Dye-labeling conditions should be optimized for each tagged POI. Both dye concentration and labeling time can be varied, taking into consideration the preservation of enzymatic activity. Labeling of SNAP, CLIP, and HALO tags is fairly robust to many conditions, so labeling can often be done during, as well as after, the extract production. We found that incubation of nuclear extracts for 1 h at 4°C with 0.5 µM of SNAP or HALO dyes while gently rotating yields almost complete labeling for RNA polymerase II and transcription factors (Rosen *et al.* 2020; Baek *et al.* 2021). To provide maximal signal-to-noise for CoSMoS microscopy (Rosen *et al.* 2020; Baek *et al.* 2021), residual unreacted dyes were depleted from labeled nuclear extracts using a combination of dialysis and fusion protein-coupled beads (i.e. SNAP- and/or HALO-coupled agarose beads) as previously described (Haraszti and Braun 2020).

For protein analysis, 30–50 µg of whole-cell lysates or nuclear extracts per lane were separated by SDS-PAGE, next to appropriate protein size standard lanes. If POIs were covalently dye-labeled, the protein gel was subjected to fluorescence imaging on a Typhoon imager (GE Healthcare). Note that unreacted dye runs at the buffer front, so levels of free dye can be estimated if electrophoresis is stopped before it runs off. To probe for the HA3 epitope or with POI-specific antibodies, proteins in the gel are transferred to nitrocellulose membranes for immunoblotting. For HA, blots were probed with a peroxidase-conjugated monoclonal anti-HA antibody (3F10, Roche Cat#12013819001, RRID: AB_2314622) at 1:2,000 dilution, followed by detection on film or imager using Pierce SuperSignal West chemiluminescent substrate (VWR PI34580).

To create a standard curve for quantitating both dye-labeling and the HA3 immunoblotting, recombinant SNAP, CLIP, or HALO was expressed as a fusion to poly-histidine (his6) and HA3 in bacteria, driven by a T7 promoter (see “Bacterial Expression Constructs” listed in Table 1). BL21(DE3) Codon Plus cells (Stratagene) carrying the expression plasmid were grown in LB plus 100 µg/mL kanamycin (LB+Kan). A fresh overnight culture was used to inoculate 1 L of LB+Kan, and cells were grown at 37°C to OD600 of 0.6–0.8. Cells were transferred to 18°C, induced with 0.4 mM isopropyl-β-D-thiogalactopyranoside (IPTG), and allowed to grow overnight. Cells were harvested, washed, and lysed by sonication in 50-mL lysis buffer (20 mM HEPES pH 7.6, 10% glycerol, 500 mM NaCl, 5 mM beta-mercaptoethanol, and protease inhibitors). Extract was clarified by centrifugation for 50 min at 13K rpm in a GSA rotor (Sorvall). His-tagged protein was purified from the supernatant by incubation with 1 mL Ni-NTA agarose, washing the beads with 20 mL lysis buffer containing 20 mM imidazole, and elution with lysis buffer containing 300 mM imidazole. Protein-containing fractions were pooled and dialyzed against phosphate-buffered saline (PBS) containing 10% glycerol and 1 mM dithiothreitol. To maximally label the fluorescent-tagged protein standards (which we designated “100%” labeled), 10 µM protein was incubated with 100 µM dye for 1 h at 4°C. We found that a range of protein standards spanning 0.5–10 ng per lane works well for estimating fusion protein amounts in nuclear extracts. By matching the fluorescence and HA3 signals in the yeast extracts containing the fusion proteins to the intensities of the recombinant protein, labeling efficiency can be estimated as the ratio of fluorescent protein to total protein.

## Results and discussion

### Design of the C-terminal tagging plasmids

Cassettes for C-terminal tagging were created using a modular design (Fig. 1, Table 1) that incorporates the following features:

1. An optional triple influenza hemagglutinin (HA3) tag (Wilson *et al.* 1984) that has 2 functions. First, commercially available antibodies allow detection by immunoblotting, immunoprecipitation, and quantification of the fusion protein. Second, together with a Gly-Ser-Gly-Gly-Ser-Gly-Ser (GSGGSGS) linker, it provides distance and rotational flexibility between the targeted POI and the fluorescence module. It has been reported that the HA3 tagging cassette from pFA6a-HA-KlURA3 (Sung *et al.* 2008) destabilizes fusion proteins (Saiz-Baggetto *et al.* 2017). However, that study goes on to show that instability actually maps to an Ile-Phe sequence adjacent to the HA epitopes. While our C-terminal tagging vectors carry the Ile-Phe codons, a PCR primer that omits this sequence is used when amplifying the integration cassette (see below). To create fusions without the HA3 tag, a PCR primer complementary to the GSGGSGS is used.
  If desired, it should be relatively easy to replace HA3 with other epitopes such as MYC, V5, or FLAG. A new plasmid can be generated by Gibson assembly, using the vector and GSGGSGS sequences that flank the HA3 as anchors for the new tag. Alternatively, a new epitope sequence can be incorporated into the upstream primer used for integration cassette amplification (Fig. 1) by placing it between the POI and GSGGSGS sequences.
2. In frame with HA3-GSGGSGS, each cassette next encodes one of 4 protein fusion partners that allows for rapid and efficient fluorescent dye labeling. We chose the well-characterized HALO, SNAP, CLIP, and *E. coli* DHFR proteins. The HALO tag is a mutated dehalogenase that covalently links to chloroalkane substrates (Los *et al.* 2008). Similarly, the SNAP and CLIP tags are modified DNA repair enzymes that covalently link to benzyl-guanine and benzyl-cytidine, respectively (Keppler *et al.* 2003; Gautier *et al.* 2008; https://www.neb.com/products/protein-tools/protein-labeling/protein-labeling). Note that the “fast” versions of SNAP and CLIP were developed to speed the kinetics of substrate linkage, thereby minimizing labeling time and increasing labeling efficiency. The high-affinity interaction between DHFR and the small molecule trimethoprim (TMP) is noncovalent, but still useful for labeling (Miller *et al.* 2005; Calloway *et al.* 2007). Although not used in our vector set, a modified version of the DHFR system has been developed for covalent linkage to TMP (Chen *et al.* 2012; Wang *et al.* 2014). For some of these enzymatic modules, substrates are available not only for coupling to fluorescent dyes but also to other functional groups such as biotin that can be used for linkage to streptavidin-coated beads for purification, immobilizing onto a streptavidin-coated microscope slide surface, or other applications.
3. To prevent transcription readthrough into the downstream selectable marker while also minimizing cassette size, a short synthetic transcription terminator (MacPherson and Saka 2017) is placed downstream of the fusion protein.
4. The last component of the cassette is one of 5 markers providing selection for integration into the genome. These markers all derive from the MX series of vectors, which have no *S. cerevisiae* sequences that could lead to integration at places other than the intended target. All selection markers use the *Ashbya gossypii* TEF promoter and terminator flanking the selection marker coding region. The five chosen are those encoding *C. albicansURA3* homolog (from pAG61; Goldstein *et al.* 1999), Kanamycin/G418 resistance (Bahler *et al.* 1998), Nourseothricin resistance (pAG25), Bleomycin/Phleomycin resistance (pUG66), or Hygromycin resistance (pAG32; Goldstein and McCusker 1999). If other markers are desired, the TEF promoter and terminator can be used as overlap sites for Gibson assembly reactions or for recombinational marker swapping of a tagging cassette that was previously integrated into a yeast chromosome.

### Usage of the C-terminal tagging plasmids

**Fig. 2.**
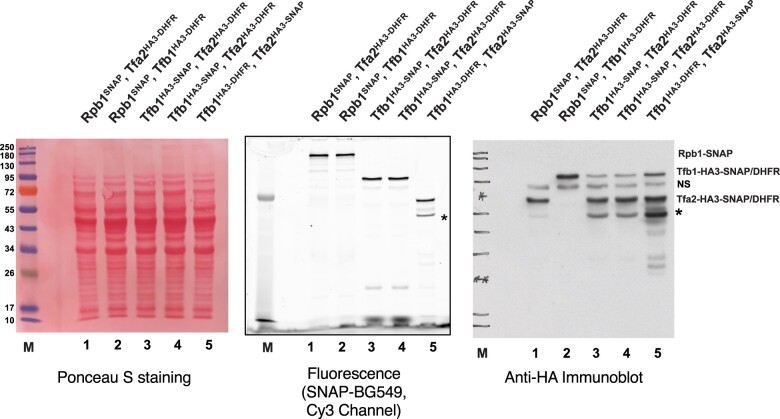
Validation of tagged strains. Yeast nuclear extracts were made from yeast strains YSB3474 (lane 1, Rpb1-SNAP, Tfa2-HA3-DHFR), YSB3473 (lane 2, Rpb1-SNAP, Tfb1-HA3-DHFR), YSB 3535 (lanes 3 and 4, Tfb1-HA3-SNAP, Tfa2-HA3-DHFR; two isolates), and YSB 3537 (lane 5, Tfb1-HA3-DHFR, Tfa2-HA3-SNAP). SNAP fusions in the extracts were labeled with SNAP-Surface 549 (New England Biolabs, cat# S9112S) during nuclear extract preparation. Extracts were then resolved by 10% SDS-PAGE. Center panel shows fluorescence imaging of the gel before transfer to nitrocellulose membrane. The left panel shows Ponceau S staining for total protein and right panel shows immunoblotting for the HA3 tag. Positions of tagged proteins are shown at right, sizes of molecular weight markers are shown at left (lane M). NS marks a nonspecific band that cross-reacts with anti-HA antibody 3F10, and asterisk marks presumed cleavage product of the tagged proteins.

To tag a POI, 2 PCR primers are needed (Fig. 1). The upstream forward primer should have 40–60 nt of complementarity to the POI C-terminal coding region, up to but not including the stop codon, with the reading frame extending 3′ into the HA3 tag (if desired) or the GSGGSGS linker coding sequence. Note that a different epitope tag (e.g. FLAG, MYC, or V5) can also be inserted in the oligo, placed in frame between the POI and GSGGSGS linker, if desired. The reverse primer should contain 40–60 nt of complementarity to the opposite strand downstream of the POI stop codon, followed by complementary to the TEF terminator region. Using standard protocols, the PCR product is transformed into *S. cerevisiae* for recombination into the target POI gene. After selection for the cassette marker, the expected integration event should be confirmed by PCR, DNA sequencing, or southern blot. The expression of the fusion protein can be assayed by immunoblotting with antibodies against the HA3 epitope or POI, as well as by fluorescence imaging of a protein gel after labeling with the appropriate small molecule dye (a typical analysis is shown in Fig. 2, middle). The tagged strain should be tested for any phenotypes that could suggest the tag interferes with POI function. In our experience, in cells with no growth defects, the tagged proteins are generally expressed at the same level as the untagged parent. In a few cases when monitoring by HA3 blotting, the DHFR tag appeared to produce less fusion protein compared to an SNAP fusion. This reduction may reflect degradation of the entire protein, or only cleavage of the DHFR-HA3 module from the POI. Therefore, users may want to test different tags for their particular POI.

### N-terminal tagging plasmid design

**Fig. 3.**
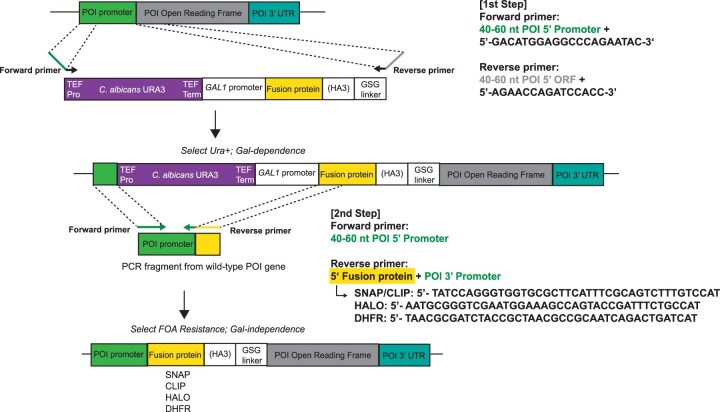
Schematic of the two-step N-terminal tagging strategy. Using one of the N-terminal tagging plasmids described listed in Table 1, an integration cassette is generated by PCR for the first transformation. The upstream primer should have 40–60 nt of complementarity to the POI promoter region, followed by the indicated sequence to prime within the TEF promoter of the *URA3* selectable marker cassette. The downstream primer should have 40–60 nt of complementarity to the 5′ region of the POI ORF, followed by the indicated sequence that primes within the GSGGSGS linker. Note that this primer must preserve the reading frame between the linker and POI. Transformants are selected on galactose media lacking uracil and then tested for the proper integration and galactose-dependent fusion protein expression. Restoration of the endogenous promoter is achieved by a second-step transformation that removes the *URA3* cassette and *GAL1* promoter. A DNA fragment containing the full POI promoter and a short region of complementarity to the fluorescence module is created by PCR using the wild-type POI gene as template. The upstream primer should hybridize to a region of the POI promoter upstream of the *URA3* integration site. Note that in the figure, this is shown as the same sequence used for the PCR reaction in Step 1, but in practice, it can be further upstream to provide a longer region of homology for integration. The downstream primer should have 40 or more nt of sequence complementary to the most 5′ sequence of the fluorescence module to target integration, followed by 16–20 nt that prime at the POI promoter just upstream of the natural initiation codon. Transformants are selected for loss of *URA3* on 5-FOA media and galactose-independent expression of the fusion.

**Fig. 4.**
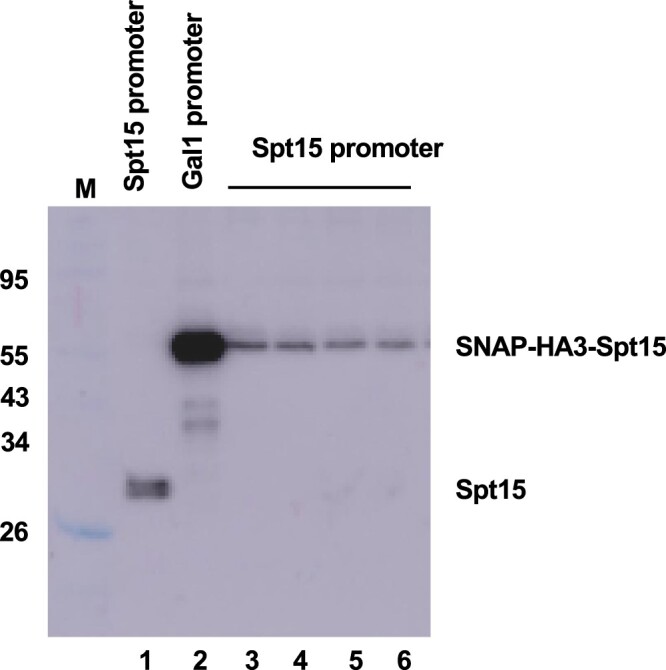
Two-step N-terminal tagging of Spt15. Immunoblotting with anti-Spt15 antibody was performed on whole-cell extracts from the following strains: (lane 1) parent strain YF702 expressing wild-type Spt15, (lane 2) YSB3630 grown in galactose media, over-expressing the SNAP-HA3-Spt15 fusion from the GAL1 promoter, and (lanes 3–6) multiple isolates of YSB3643, in which the Spt15 promoter is restored to allow normal expression levels on glucose media.

The N-terminal tagging cassettes were designed for 2-step integration using the method of Booher and Kaiser (2008). In the first step, *URA3* complementation is used to select for integration of a galactose-inducible promoter expressing the fluorescence labeling fusion protein. In a second transformation, a DNA fragment carrying the natural POI promoter is used to replace the *URA3* cassette and galactose-inducible promoter just upstream of the fusion protein (Figs. 3 and 4).

Similar to the C-terminal tagging vectors, these constructs have a modular design (Table 1), incorporating the following features:

1. A selectable marker cassette consisting of the TEF promoter and terminator expressing *C. albicans URA3*. This allows selection for the initial integration, as well as counter-selection for the second-step excision that restores the natural POI promoter.
2. The *GAL1* promoter, which is used to drive the expression of the fusion protein during the first stage of integration.
3. The fluorescent labeling module (DHFR, SNAP, CLIP, or HALO) as described for the C-terminal tagging cassettes.
4. A linker region cassette consisting of the HA3 tag and GSGGSGS, or just GSGGSGS.

### Usage of the N-terminal tagging plasmids

To tag a POI, the integration cassette is PCR amplified using 2 primers (Fig. 3). The upstream primer carries 40–60 nt of complementarity to the POI promoter region followed by 19 nt that hybridize at the upstream edge of the TEF promoter (GACATGGAGGCCCAGAATAC). The downstream primer contains 40–60 nt of homology to the 5′ end of the POI open reading frame, followed by the 15 nt of the GSGGSGS linker sequence (AGAACCAGATCCACC), linking the fluorescence tag and POI.

The resulting fragment is transformed into the recipient strain, selecting for the *URA3* marker. Note that this first transformation must be plated on media containing galactose if the POI is to be expressed. Indeed, for essential genes, the ability to grow on galactose but not glucose can be used as a secondary screen for proper integration. The *GAL1* promoter is quite strong, so this intermediate strain will usually overexpress the POI. If this causes toxicity, expression may be tuned down using a mixture of galactose and raffinose in the media (Biggar and Crabtree 2001).

To restore normal expression levels, the natural POI promoter can be reintegrated to replace the *URA3* gene and *GAL1* promoter. The restoring DNA fragment is made by PCR from the wild-type POI gene. The upstream primer hybridizes in the POI promoter upstream of the *URA3* integration site, while the downstream primer carries 40 nt of homology to the 5′ ORF region (including the initiation codon) of the fluorescent module (i.e. SNAP, CLIP, HALO, or DHFR) followed by the POI promoter sequence just upstream of the natural initiation codon. This second transformation is plated on media containing 5-FOA (selecting for loss of *URA3*) and glucose (selecting for loss of galactose-dependence in the case of essential genes). Proper expression of the intact fusion protein can be verified using antibodies against the POI, the fluorescent tag, or HA3 tag. Figure 4 shows an example in which the essential Spt15 (TATA Binding Protein) protein (lane 1) was first tagged and expressed from the *GAL1* promoter (lane 2), followed by the replacement of the natural *SPT15* promoter to restore normal expression levels (lanes 3–6).

### Determining protein levels and labeling efficiency

It is important to check integrity of the tagged proteins by gel electrophoresis and immunoblotting of whole-cell or nuclear extracts. The best option is to use an antibody against the POI itself, if available, as this allows direct comparison of tagged and untagged protein levels (e.g. see Fig. 4). If the fusion is sensitive to proteolysis, one can see how much of the protein exists in the cleaved (and therefore unlabeled state). If a specific antibody is not available, fusion proteins can be visualized using commercially available antibodies against the HA3 epitope (multiple vendors), SNAP/CLIP (New England Biolabs), or the HALO tag (Promega and Chromotek). These blots may also reveal proteolytic fragments that retain the HA3 epitope or fusion labeling tags (Fig. 2, right panel). In our experience, levels of cleavage can range from negligible to significant, depending on the specific POI. In some cases, cleavage appears to map to the HA3-GSGGSGS region, and if so, one or both parts of this linker can be omitted to see if that improves stability. We also observe that cleavage can vary with the particular extract procedure used, indicating that degradation can occur after cell lysis. A denaturing extract is therefore most useful when the fluorescence tags will be used for in vivo imaging, while quality checks for in vitro applications should use the extract conditions to be used for the assay.

**Fig. 5.**
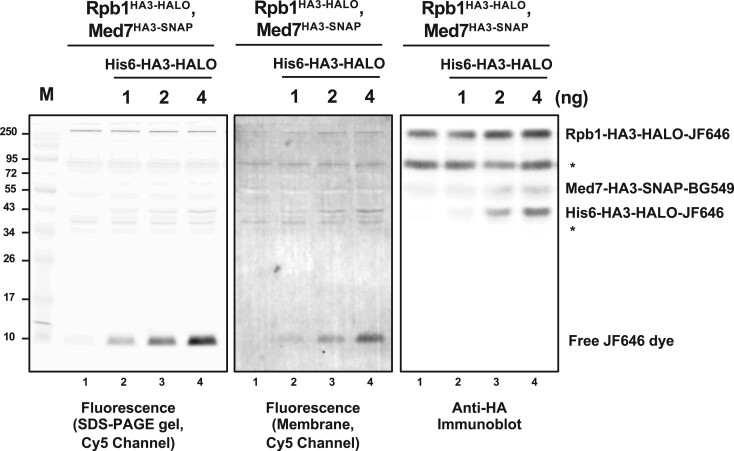
Estimating labeling efficiency using recombinant his6-HA3-HALO as a standard. Nuclear extract from yeast strain YSB3687, containing Rpb1-HA3-HALO labeled with JF646-HaloTag ligand and Med7-HA3-SNAP labeled with SNAP-Surface 549, was prepared and separated by 10% PAGE. Lanes 1–4 all contain the same amount of extract, together with increasing amounts (0, l, 2, or 4 ng) of recombinant his6-HA3-HALO protein that was labeled to saturation using JF646. Levels of fluorescently labeled HALO protein were analyzed in the red channel (Cy5 filter set), both in the wet gel (left panel) and after blotting onto a membrane (central panel). Total HALO protein was analyzed using anti-HA immunoblotting and chemiluminescence. Densitometry (in arbitrary units) showed that the fluorescence/HA ratio for Rpb1-HA3-HALO (lane 3: 410/4391 = 0.093, lane 4: 396/5034 = 0.079) and recombinant his6-HA3-HALO (lane 3: 139/1606 = 0.086, lane 4: 243/3093 = 0.079) were found to be essentially the same, suggesting that labeling of Rpb1 in the extract was nearly complete. Asterisks mark presumed cleavage products of the tagged proteins.

To quantitatively estimate fusion protein amounts and labeling efficiency, a fully labeled standard can be generated and run on a polyacrylamide gel next to the endogenous tagged POI (Fig. 5). For the standard, recombinant his6-HA3-SNAP/CLIP/HALO protein was expressed in *E. coli* and purified using nickel affinity chromatography. This protein was then labeled to saturation in vitro using the appropriate dye. If one assumes the labeling efficiency of this recombinant protein is close to 100%, the amount of fluorescent POI in the extract can be compared with the standard using fluorescence imaging of the gel. The absolute levels of protein (labeled and unlabeled) can similarly be determined by blotting the gel and probing for the HA3 tag present on both the protein standard and POI (Fig. 5). The ratio of fluorescently labeled POI to total POI then provides a rough estimate of labeling efficiency. The tolerance for incomplete labeling will depend on the specific experiment, so the standard curve can serve as a quality checkpoint before proceeding.

### Use of multiple tags within the same strain

**Fig. 6.**
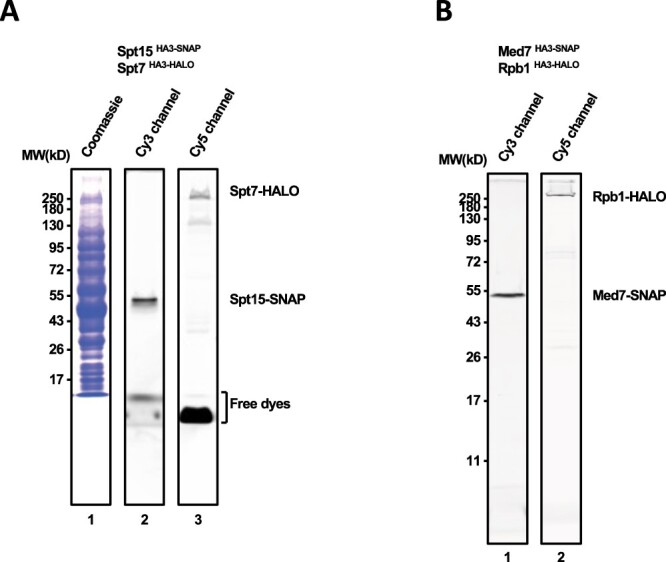
Yeast strains with multiple tags. a) Nuclear extracts were made from YSB3685 cells containing SNAP-tagged TATA-Binding Protein (Spt15-SNAP) and HALO-tagged Spt7 (Spt7-HALO). The extracts were simultaneously labeled with JF646-HaloTag ligand and SNAP-Surface 549 for 1 h at 4°C. Proteins were resolved by SDS-PAGE, stained with Coomassie dye (lane 1), and imaged in the Cy3 (lane 2) and Cy5 (lane 3) channels as described in *Materials and Methods*. b) Same as (a), except from YSB3687 cells containing Rpb1-HALO and Med7-SNAP.

Several things need consideration when constructing strains with multiple tags. Obviously, different fluorescent tagging cassettes must be used if two or more proteins are to be labeled with different colored dyes. We have simultaneously labeled yeast nuclear extracts with SNAP, CLIP, and HALO dyes, with no cross-reactivity between target modules [Rosen *et al.* 2020; Fig. 6, see also Baek *et al.* (2021)]. Because the DHFR–TMP interaction is noncovalent that dye is generally added to reactions just before loading onto the microscope. The different tagging constructs must also carry different selectable markers.

When creating strains with more than one tagging module, individual cassettes are sequentially transformed into the genome. However, it is essential to maintain selection for all pre-existing cassettes as well as the new cassette being inserted. Because of the modular configuration of these constructs, homologous DNA sequences appear through the cassette. Therefore, the new cassette can not only recombine into the target locus but also into any previously integrated cassette. This second pathway results in loss of the pre-existing marker and/or tag, so these transformants can be minimized by selections for all tags. Multiply tagged strains should be verified by immunoblotting and/or fluorescence imaging. Note that if the tagged proteins all contain the HA3 tag, immunoblotting can be used to determine the relative amounts of each fusion (Figs. 2 and 5).

### Conclusions

Using the constructs described here, we have successfully generated yeast strains with up to three differently tagged proteins and used them for multicolor single-molecule imaging of in vitro transcription reactions (Rosen *et al.* 2020; Baek *et al.* 2021). Proteins labeled with SNAP and HALO tags have also proven useful for in vitro FRET experiments (Ishitsuka *et al.* 2015), immobilization on slides or beads, and other assays. These constructs should also prove useful for in vivo imaging techniques, particularly single-molecule tracking (Lionnet and Wu 2021). Note that for in vivo applications, others have found that deletion of the *PDR5* drug efflux pump may improve labeling efficiency by increasing in vivo dye concentrations (Nguyen *et al.* 2021). By creating and sharing these vectors, we hope to provide a useful resource for the *S. cerevisiae* research community.

## Data availability

The data underlying this article are available in the article and in its online Supplementary material. Plasmids may be obtained from Addgene.org or from the corresponding author.

Supplemental material is available at *G3* online.

## Supplementary Material

jkac201_Supplemental_Tables

jkac201_Supplemental_Data

jkac201_Supplemental_Material_Legends
