# Supplementary material for: A set of Saccharomyces cerevisiae integration vectors for fluorescent dye labeling of proteins
Source: G3 (Bethesda). 2022 Aug 9;12(10):jkac201. doi: 10.1093/g3journal/jkac201 (PMC9526040; doi:10.1093/g3journal/jkac201)
Supplement: jkac201_Supplemental_Data [file jkac201_supplemental_data.pdf]

## Supplementary Data. Maps and Sequences of tagging plasmids.

| Plasmid Name | N or C terminal | Fusion | Selectable Marker | Catalog Number | Freezer Number |
|---|---|---|---|---|---|
| *Yeast tagging plasmids* | | | | | |
| pBS-SKII-3XHA-fSNAP-Hygromycin | C | fSNAP | Hygromycin | YV307 | 4526 |
| pBS-SKII-3XHA-fSNAP-Phleomycin | C | fSNAP | Phleomycin | YV308 | 4527 |
| pBS-SKII-3XHA-fSNAP-NAT | C | fSNAP | Nourseothricin | YV309 | 4528 |
| pBS-SKII-3XHA-fSNAP-URA | C | fSNAP | C. albicans URA3 | YV310 | 4529 |
| pBS-SKII-3XHA-fSNAP-Kan | C | fSNAP | G418/Kanamycin | YV311 | 4530 |
| pBS-SKII-3XHA-fCLIP-Hygromycin | C | fCLIP | Hygromycin | YV312 | 4531 |
| pBS-SKII-3XHA-fCLIP-Phleomycin | C | fCLIP | Phleomycin | YV313 | 4532 |
| pBS-SKII-3XHA-fCLIP-NAT | C | fCLIP | Nourseothricin | YV314 | 4533 |
| pBS-SKII-3XHA-fCLIP-URA | C | fCLIP | C. albicans URA3 | YV315 | 4534 |
| pBS-SKII-3XHA-fCLIP-Kan | C | fCLIP | G418/Kanamycin | YV316 | 4535 |
| pBS-SKII-3XHA-eDHFR-Hygromycin | C | eDHFR | Hygromycin | YV317 | 4536 |
| pBS-SKII-3XHA-eDHFR-Phleomycin | C | eDHFR | Phleomycin | YV318 | 4537 |
| pBS-SKII-3XHA-eDHFR-NAT | C | eDHFR | Nourseothricin | YV319 | 4538 |
| pBS-SKII-3XHA-eDHFR-URA | C | eDHFR | C. albicans URA3 | YV320 | 4539 |
| pBS-SKII-3XHA-eDHFR-Kan | C | eDHFR | G418/Kanamycin | YV321 | 4540 |
| pBS-SKII-3XHA-HALO-Hygromycin | C | HALO | Hygromycin | YV328 | 4640 |
| pBS-SKII-3XHA-HALO-Phleomycin | C | HALO | Phleomycin | YV329 | 4641 |
| pBS-SKII-3XHA-HALO-NAT | C | HALO | Nourseothricin | YV330 | 4642 |
| pBS-SKII-3XHA-HALO-URA | C | HALO | C. albicans URA3 | YV331 | 4643 |
| pBS-SKII-3XHA-HALO-Kanamycin | C | HALO | G418/Kanamycin | YV332 | 4644 |
| pBS-SKII-URA-GAL1-DHFR-3xHA | N | eDHFR | C. albicans URA3 | YV333 | 4670 |
| pBS-SKII-URA-GAL1-fSNAP-3xHA | N | fSNAP | C. albicans URA3 | YV334 | 4671 |
| pBS-SKII-URA-GAL1-fCLIP-3xHA | N | fCLIP | C. albicans URA3 | YV335 | 4672 |
| pBS-SKII-URA-GAL1-HALO-3xHA | N | HALO | C. albicans URA3 | YV357 | 4712 |
| pBS-SKII-URA-GAL1-fSNAP-GS | N | fSNAP | C. albicans URA3 | YV358 | 4713 |
| pBS-SKII-URA-GAL1-fCLIP-GS | N | fCLIP | C. albicans URA3 | YV359 | 4714 |
| pBS-SKII-URA-GAL1-HALO-GS | N | HALO | C. albicans URA3 | YV360 | 4715 |
| | | | | | |
| *Bacterial expression constructs* | | | | | |
| pET28-his6-HA3-SNAP | | SNAP | KanR | BE570 | 4633 |
| pET28-his6-HA3-CLIP | | CLIP | KanR | BE571 | 4639 |
| pET28-his6-HA3-HALO | | HALO | KanR | BE572 | 4645 |

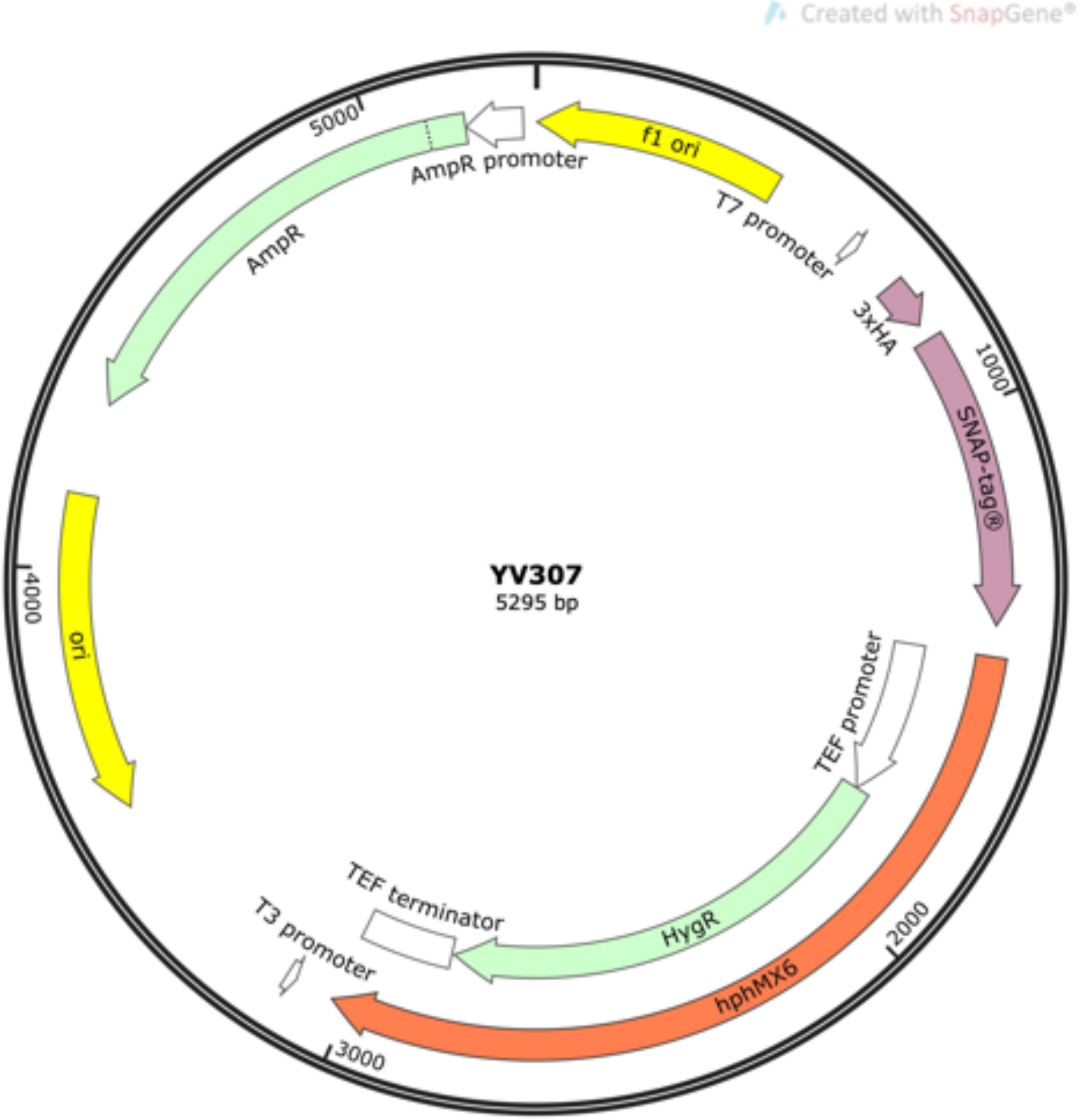
Created with SnapGene®
YV307
5295 bp
f1 ori
T7 promoter
3xHA
SNAP-tag®
TEF promoter
HygR
TEF terminator
T3 promoter
hphMX6
ori
AmpR
AmpR promoter
1000
2000
3000
4000
5000

**YV307: pBS-SKII-3XHA-fSNAP-Hygromycin**

**ctaaattgtaagcgttaatattttgttaaaattcgcgttaaatttttgttaaatcagctcattttttaaccaataggccgaaatcggcaaa**
**atcccttataaatcaaaagaatagaccgagatagggttgagtgttgttccagtttggaacaagagtccactattaaagaacgtggact**
**ccaacgtcaaagggcgaaaaaccgtctatcagggcgatggcccactacgtgaaccatcaccctaatcaagttttttggggtcgaggtg**
**ccgtaaagcactaaatcggaaccctaaagggagcccccgatttagagcttgacggggaaagccggcgaacgtggcgagaaaggaag**
**ggaagaaagcgaaaggagcgggcgctagggcgctggcaagtgtagcggtcacgctgcgcgtaaccaccacacccgccgcgcttaatg**
**cgccgctacagggcgcgtcccattcgccattcaggctgcgcaactgttgggaagggcgatcggtgcgggcctcttcgctattacgccagc**
**tggcgaaagggggatgtgctgcaaggcgattaagttgggtaacgccagggttttcccagtcacgacgttgtaaaacgacggccagtga**
**gcgcgcgtaatacgactcactatagggcgaattgggtaccgggccccccctcgaggtcgacggtatcgataagcttgatatcgggtcga**
**cggatccccgggttaattaacatctttTACCCATACGATGTTCCTGACTATGCGGGCTATCCGTATGACGTCCCGG**
**ACTATGCAGGATCCTATCCATATGACGTTCCAGATTACGCTGCTCAGTGCggttcaggtggatctggttctATG**
**GACAAAGACTGCGAAATGAAGCGCACCACCCTGGATAGCCCTCTGGGCAAGCTGGAACTGTCTGGGT**
**GCGAACAGGGCCTGCACCGTATCATCTTCCTGGGCAAAGGAACATCTGCCGCCGACGCCGTGGAAGT**
**GCCTGCCCCAGCCGCCGTGCTGGGCGGACCAGAGCCACTGATGCAGGCCACCGCCTGGCTCAACGCCT**
**ACTTTCACCAGCCTGAGGCCATCGAGGAGTTCCCTGTGCCAGCCCTGCACCACCCAGTGTTCCAGCAGG**
**AGAGCTTTACCCGCCAGGTGCTGTGGAAACTGCTGAAAGTGGTGAAGTTCGGAGAGGTCATCAGCTA**
**CAGCCACCTGGCCGCCCTGGCCGGCAATCCCGCCGCCACCGCCGCCGTGAAAACCGCCCTGAGCGGAA**
**ATCCCGTGCCCATTCTGATCCCCTGCCACCGGGTGGTGCAGGGCGACCTGGACGTGGGGGGCTACGAG**
**GGCGGGCTCGCCGTGAAAGAGTGGCTGCTGGCCCACGAGGGCCACAGACTGGGCAAGCCTGGGCTG**
**GGTtaaTATATAACTGTCTAGAAATAAAGAGTATCATCTTTCAAAGGTCACCCGGCCAGCGACATGGA**
**GGCCCAGAATACCCTCCTTGACAGTCTTGACGTGCGCAGCTCAGGGGCATGATGTGACTGTCGCCCGT**
**ACATTTAGCCCATACATCCCCATGTATAATCATTTGCATCCATACATTTTGATGGCCGCACGGCGCGAA**
**GCAAAAATTACGGCTCCTCGCTGCgGACCTGCGAGCAGGGAAACGCTCCCCTCACAGACGCGTTGAAT**
**TGTCCCCACGCCGCGCCCCTGTAGAGAAATATAAAAGGTTAGGATTTGCCACTGAGGTTCTTCTTTCAT**
**ATACTTCCTTTTAAAATCTTGCTAGGATACAGTTCTCACATCACATCCGAACATAAACAACCATGGGTA**
**AAAAGCCTGAACTCACCGCGACGTCTGTCGAGAAGTTTCTGATCGAAAAGTTCGACAGCGTCTCCGAC**
**CTGATGCAGCTCTCGGAGGGCGAAGAATCTCGTGCTTTCAGCTTCGATGTAGGAGGGCGTGGATATGT**
**CCTGCGGGTAAATAGCTGCGCCGATGGTTTCTACAAAGATCGTTATGTTTATCGGCACTTTGCATCGGC**
**CGCGCTCCCGATTCCGGAAGTGCTTGACATTGGGGAATTCAGCGAGAGCCTGACCTATTGCATCTCCC**
**GCCGTGCACAGGGTGTCACGTTGCAAGACCTGCCTGAAACCGAACTGCCCGCTGTTCTGCAGCCGGTC**
**GCGGAGGCCATGGATGCGATCGCTGCGGCCGATCTTAGCCAGACGAGCGGGTTCGGCCCATTCGGAC**
**CGCAAGGAATCGGTCAATACACTACATGGCGTGATTTCATATGCGCGATTGCTGATCCCCATGTGTATC**
**ACTGGCAAACTGTGATGGACGACACCGTCAGTGCGTCCGTCGCGCAGGCTCTCGATGAGCTGATGCTT**
**TGGGCCGAGGACTGCCCCGAAGTCCGGCACCTCGTGCACGCGGATTTCGGCTCCAACAATGTCCTGAC**
**GGACAATGGCCGCATAACAGCGGTCATTGACTGGAGCGAGGCGATGTTCGGGGATTCCCAATACGAG**
**GTCGCCAACATCTTCTTCTGGAGGCCGTGGTTGGCTTGTATGGAGCAGCAGACGCGCTACTTCGAGCG**
**GAGGCATCCGGAGCTTGCAGGATCGCCGCGGCTCCGGGCGTATATGCTCCGCATTGGTCTTGACCAAC**
**TCTATCAGAGCTTGGTTGACGGCAATTTCGATGATGCAGCTTGGGCGCAGGGTCGATGCGACGCAATC**
**GTCCGATCCGGAGCCGGGACTGTCGGGCGTACACAAATCGCCCGCAGAAGCGCGGCCGTCTGGACCG**
**ATGGCTGTGTAGAAGTACTCGCCGATAGTGGAAACCGACGCCCCAGCACTCGTCCGAGGGCAAAGGA**
**ATAATCAGTACTGACAATAAAAAGATTCTTGTTTTCAAGAACTTGTCATTTGTATAGTTTTTTTATATTG**
**TAGTTGTTCTATTTTAATCAAATGTTAGCGTGATTTATATTTTTTTTCGCCTCGACATCATCTGCCCAGAT**
**GCGAAGTTAAGTGCGCAGAAAGTAATATCATGCGTCAATCGTATGTGAATGCTGGTCGCTATACTGCT**

GTCGATTCGATACTAACGCCGCcactagttctagagcggccgccaccgcggtggagctccagcttttgttccctttagtgaggg
ttaattgcgcgcttggcgtaatcatggtcatagctgtttcctgtgtgaaattgttatccgctcacaattccacacaacatacgagccggaa
gcataaagtgtaaagcctggggtgcctaatgagtgagctaactcacattaattgcgttgcgctcactgcccgctttccagtcgggaaac
ctgtcgtgccagctgcattaatgaatcggccaacgcgcggggagaggcggtttgcgtattgggcgctcttccgcttcctcgctcactgact
cgctgcgctcggtcgttcggctgcggcgagcggtatcagctcactcaaaggcggtaatacggttatccacagaatcaggggataacgc
aggaaagaacatgtgagcaaaaggccagcaaaaggccaggaaccgtaaaaaggccgcgttgctggcgtttttccataggctccgccc
ccctgacgagcatcacaaaaatcgacgctcaagtcagaggtggcgaaacccgacaggactataaagataccaggcgtttccccctgg
aagctccctcgtgcgctctcctgttccgaccctgccgcttaccggatacctgtccgcctttctcccttcgggaagcgtggcgctttctcatag
ctcacgctgtaggtatctcagttcggtgtaggtcgttcgctccaagctgggctgtgtgcacgaaccccccgttcagcccgaccgctgcgcc
ttatccggtaactatcgtcttgagtccaacccggtaagacacgacttatcgccactggcagcagccactggtaacaggattagcagagc
gaggtatgtaggcggtgctacagagttcttgaagtggtggcctaactacggctacactagaaggacagtatttggtatctgcgctctgct
gaagccagttaccttcggaaaaagagttggtagctcttgatccggcaaacaaaccaccgctggtagcggtggtttttttgtttgcaagc
agcagattacgcgcagaaaaaaaggatctcaagaagatcctttgatcttttctacggggtctgacgctcagtggaacgaaaactcacg
ttaagggattttggtcatgagattatcaaaaaggatcttcacctagatccttttaaattaaaaatgaagttttaaatcaatctaaagtat
atatgagtaaacttggtctgacagttaccaatgcttaatcagtgaggcacctatctcagcgatctgtctatttcgttcatccatagttgcct
gactccccgtcgtgtagataactacgatacgggagggcttaccatctggccccagtgctgcaatgataccgcgagacccacgctcaccg
gctccagatttatcagcaataaaccagccagccggaagggccgagcgcagaagtggtcctgcaactttatccgcctccatccagtctat
taattgttgccgggaagctagagtaagtagttcgccagttaatagtttgcgcaacgttgttgccattgctacaggcatcgtggtgtcacg
ctcgtcgtttggtatggcttcattcagctccggttcccaacgatcaaggcgagttacatgatcccccatgttgtgcaaaaaagcggttag
ctccttcggtcctccgatcgttgtcagaagtaagttggccgcagtgttatcactcatggttatggcagcactgcataattctcttactgtca
tgccatccgtaagatgcttttctgtgactggtgagtactcaaccaagtcattctgagaatagtgtatgcggcgaccgagttgctcttgccc
ggcgtcaatacgggataataccgcgccacatagcagaactttaaaagtgctcatcattggaaaacgttcttcggggcgaaaactctca
aggatcttaccgctgttgagatccagttcgatgtaacccactcgtgcacccaactgatcttcagcatcttttactttcaccagcgtttctgg
gtgagcaaaaacaggaaggcaaaatgccgcaaaaaagggaataagggcgacacggaaatgttgaatactcatactcttcctttttca
atattattgaagcatttatcagggttattgtctcatgagcggatacatatttgaatgtatttagaaaaataaacaaataggggttccgcg
cacatttccccgaaaagtgccac

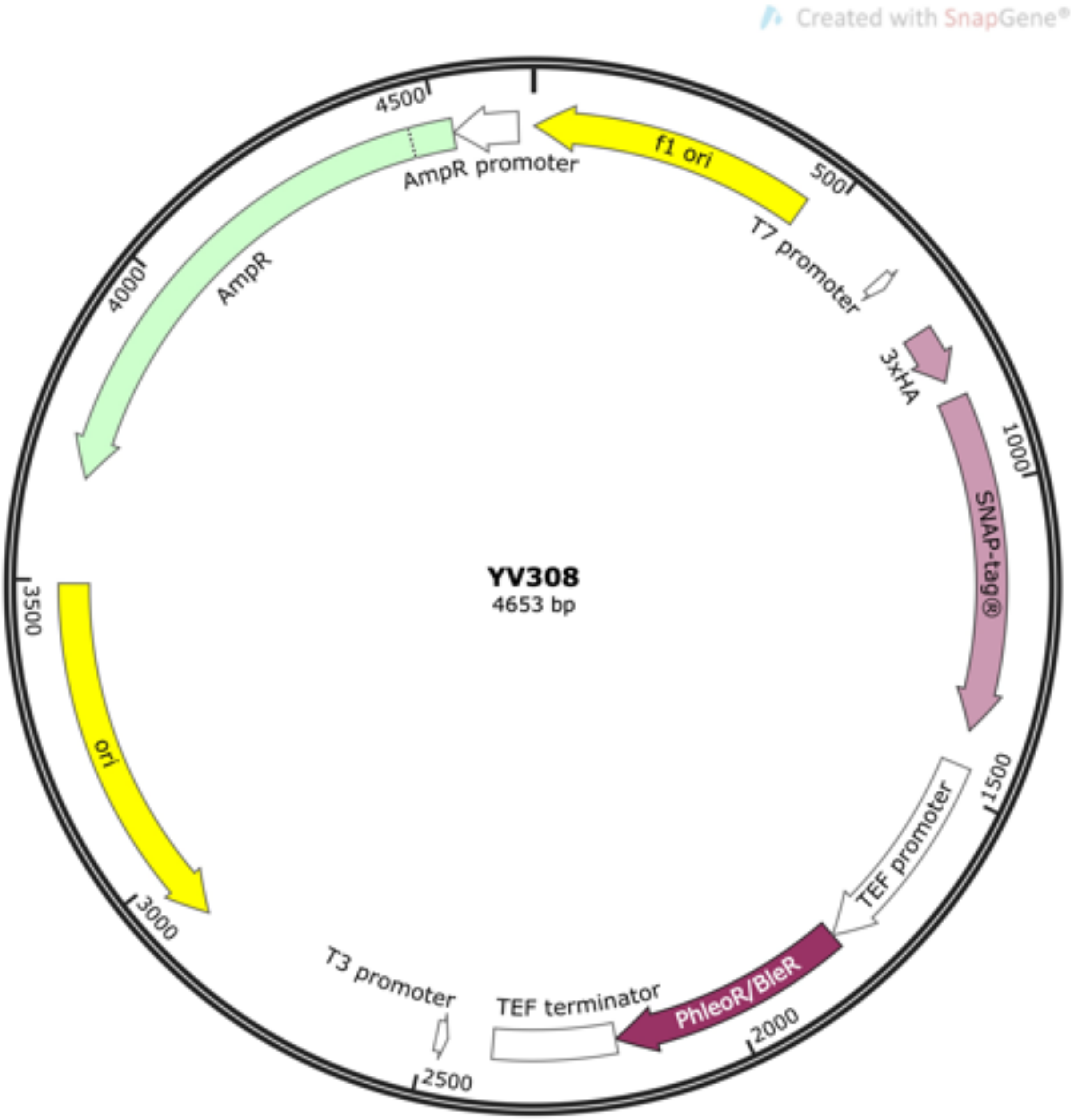

Created with SnapGene®
YV308
4653 bp
f1 ori
T7 promoter
3xHA
SNAP-tag®
TEF promoter
PhleoR/BleR
TEF terminator
T3 promoter
ori
AmpR
AmpR promoter
500
1000
1500
2000
2500
3000
3500
4000
4500

**YV308: pBS-SKII-3XHA-fSNAP-Phleomycin**

**ctaaattgtaagcgttaatattttgttaaaattcgcgttaaatttttgttaaatcagctcattttttaaccaataggccgaaatcggcaaa
atcccttataaatcaaaagaatagaccgagatagggttgagtgttgttccagtttggaacaagagtccactattaaagaacgtggact
ccaacgtcaaagggcgaaaaaccgtctatcagggcgatggcccactacgtgaaccatcaccctaatcaagttttttggggtcgaggtg
ccgtaaagcactaaatcggaaccctaaagggagcccccgatttagagcttgacggggaaagccggcgaacgtggcgagaaaggaag
ggaagaaagcgaaaggagcgggcgctagggcgctggcaagtgtagcggtcacgctgcgcgtaaccaccacacccgccgcgcttaatg
cgccgctacagggcgcgtcccattcgccattcaggctgcgcaactgttgggaagggcgatcggtgcgggcctcttcgctattacgccagc
tggcgaaagggggatgtgctgcaaggcgattaagttgggtaacgccagggttttcccagtcacgacgttgtaaaacgacggccagtga
gcgcgcgtaatacgactcactatagggcgaattgggtaccgggccccccctcgaggtcgacggtatcgataagcttgatatcgggtcga
cggatccccgggttaattaacatctttTACCCATACGATGTTCCTGACTATGCGGGCTATCCCTATGACGTCCCGG
ACTATGCAGGATCCTATCCATATGACGTTCCAGATTACGCTGCTCAGTGCggttcaggtggatctggttctATG
GACAAAGACTGCGAAATGAAGCGCACCACCCTGGATAGCCCTCTGGGCAAGCTGGAACTGTCTGGGT
GCGAACAGGGCCTGCACCGTATCATCTTCCTGGGCAAAGGAACATCTGCCGCCGACGCCGTGGAAGT
GCCTGCCCCAGCCGCCGTGCTGGGCGGACCAGAGCCACTGATGCAGGCCACCGCCTGGCTCAACGCCT
ACTTTCACCAGCCTGAGGCCATCGAGGAGTTCCCTGTGCCAGCCCTGCACCACCCAGTGTTCCAGCAGG
AGAGCTTTACCCGCCAGGTGCTGTGGAAACTGCTGAAAGTGGTGAAGTTCGGAGAGGTCATCAGCTA
CAGCCACCTGGCCGCCCTGGCCGGCAATCCCGCCGCCACCGCCGCCGTGAAAACCGCCCTGAGCGGAA
ATCCCGTGCCCATTCTGATCCCCTGCCACCGGGTGGTGCAGGGCGACCTGGACGTGGGGGGCTACGAG
GGCGGGCTCGCCGTGAAAGAGTGGCTGCTGGCCCACGAGGGCCACAGACTGGGCAAGCCTGGGCTG
GGTtaaTATATAACTGTCTAGAAATAAAGAGTATCATCTTTCAAAGGTCACCCGGCCAGCGACATGGA
GGCCCAGAATACCCTCCTTGACAGTCTTGACGTGCGCAGCTCAGGGGCATGATGTGACTGTCGCCCGT
ACATTTAGCCCATACATCCCCATGTATAATCATTTGCATCCATACATTTTGATGGCCGCACGGCGCGAA
GCAAAAATTACGGCTCCTCGCTGCAGACCTGCGAGCAGGGAAACGCTCCCCTCACAGACGCGTTGAAT
TGTCCCCACGCCGCGCCCCTGTAGAGAAATATAAAAGGTTAGGATTTGCCACTGAGGTTCTTCTTTCAT
ATACTTCCTTTTAAAATCTTGCTAGGATACAGTTCTCACATCACATCCGAACATAAACAACCATGGGTA
TGACCGACCAAGCGACGCCCAACCTGCCATCACGAGATTTCGATcCCACcGCCGCCTTCTATGAAAGGT
TGGGCTTCGGAATCGTTTTCCGGGACGCCGGCTGGATGATCCTCCAGCGCGGGGATCTCAtGCTGGAG
TTCTTCGCCCACCCCGGGCTCGATCCCCTCGCGAGTTGGTTCAGCTGCTGCCTGAGGCTGGACGACCTC
GCGGAGTTCTACCGGCAGTGCAAATCCGTCGGCATCCAGGAAACCAGCAGCGGCTATCCGCGCATCCA
TGCCCCCGAACTGCAGGAGTGGGGAGGCACGATGGCCGCTTTGGTCGACCCGGACGGGACGCTCCTG
CGCCTGATACAGAACGAATTGCTTGCAGGCATCTCATGATCAGTACTGACAATAAAAAGATTCTTGTTT
TCAAGAACTTGTCATTTGTATAGTTTTTTTATATTGTAGTTGTTCTATTTTAATCAAATGTTAGCGTGATT
TATATTTTTTTTCGCCTCGACATCATCTGCCCAGATGCGAAGTTAAGTGCGCAGAAAGTAATATCATGC
GTCAATCGTATGTGAATGCTGGTCGCTATACTGCTGTCGATTCGATACTAACGCCGCcactagttctagagcg
gccgccaccgcggtggagctccagcttttgttccctttagtgagggttaattgcgcgcttggcgtaatcatggtcatagctgtttcctgtgt
gaaattgttatccgctcacaattccacacaacatacgagccggaagcataaagtgtaaagcctggggtgcctaatgagtgagctaact
cacattaattgcgttgcgctcactgcccgctttccagtcgggaaacctgtcgtgccagctgcattaatgaatcggccaacgcgcggggag
aggcggtttgcgtattgggcgctcttccgcttcctcgctcactgactcgctgcgctcggtcgttcggctgcggcgagcggtatcagctcact
caaaggcggtaatacggttatccacagaatcaggggataacgcaggaaagaacatgtgagcaaaaggccagcaaaaggccaggaa
ccgtaaaaaggccgcgttgctggcgtttttccataggctccgcccccctgacgagcatcacaaaaatcgacgctcaagtcagaggtggc
gaaacccgacaggactataaagataccaggcgtttccccctggaagctccctcgtgcgctctcctgttccgaccctgccgcttaccggat
acctgtccgcctttctcccttcgggaagcgtggcgctttctcatagctcacgctgtaggtatctcagttcggtgtaggtcgttcgctccaag
ctgggctgtgtgcacgaaccccccgttcagcccgaccgctgcgccttatccggtaactatcgtcttgagtccaacccggtaagacacgac**

**ttatcgccactggcagcagccactggtaacaggattagcagagcgaggtatgtaggcggtgctacagagttcttgaagtggtggccta
actacggctacactagaaggacagtatttggtatctgcgctctgctgaagccagttaccttcggaaaaagagttggtagctcttgatccg
gcaaacaaaccaccgctggtagcggtggtttttttgtttgcaagcagcagattacgcgcagaaaaaaaggatctcaagaagatccttt
gatcttttctacggggtctgacgctcagtggaacgaaaactcacgttaagggattttggtcatgagattatcaaaaaggatcttcacct a
gatccttttaaattaaaaatgaagttttaaatcaatctaaagtatatatgagtaaacttggtctgacagttaccaatgcttaatcagtga
ggcacctatctcagcgatctgtctatttcgttcatccatagttgcctgactccccgtcgtgtagataactacgatacgggagggcttacca
tctggccccagtgctgcaatgataccgcgagacccacgctcaccggctccagatttatcagcaataaaccagccagccggaagggccg
agcgcagaagtggtcctgcaactttatccgcctccatccagtctattaattgttgccgggaagctagagtaagtagttcgccagttaata
gtttgcgcaacgttgttgccattgctacaggcatcgtggtgtcacgctcgtcgtttggtatggcttcattcagctccggttcccaacgatca
aggcgagttacatgatcccccatgttgtgcaaaaaagcggttagctccttcggtcctccgatcgttgtcagaagtaagttggccgcagtg
ttatcactcatggttatggcagcactgcataattctcttactgtcatgccatccgtaagatgcttttctgtgactggtgagtactcaaccaa
gtcattctgagaatagtgtatgcggcgaccgagttgctcttgcccggcgtcaatacgggataataccgcgccacatagcagaactttaa
aagtgctcatcattggaaaacgttcttcggggcgaaaactctcaaggatcttaccgctgttgagatccagttcgatgtaacccactcgtg
cacccaactgatcttcagcatcttttactttcaccagcgtttctgggtgagcaaaaacaggaaggcaaaatgccgcaaaaaagggaat
aagggcgacacggaaatgttgaatactcatactcttcctttttcaatattattgaagcatttatcagggttattgtctcatgagcggatac
atatttgaatgtatttagaaaaataaacaaataggggttccgcgcacatttccccgaaaagtgccac**

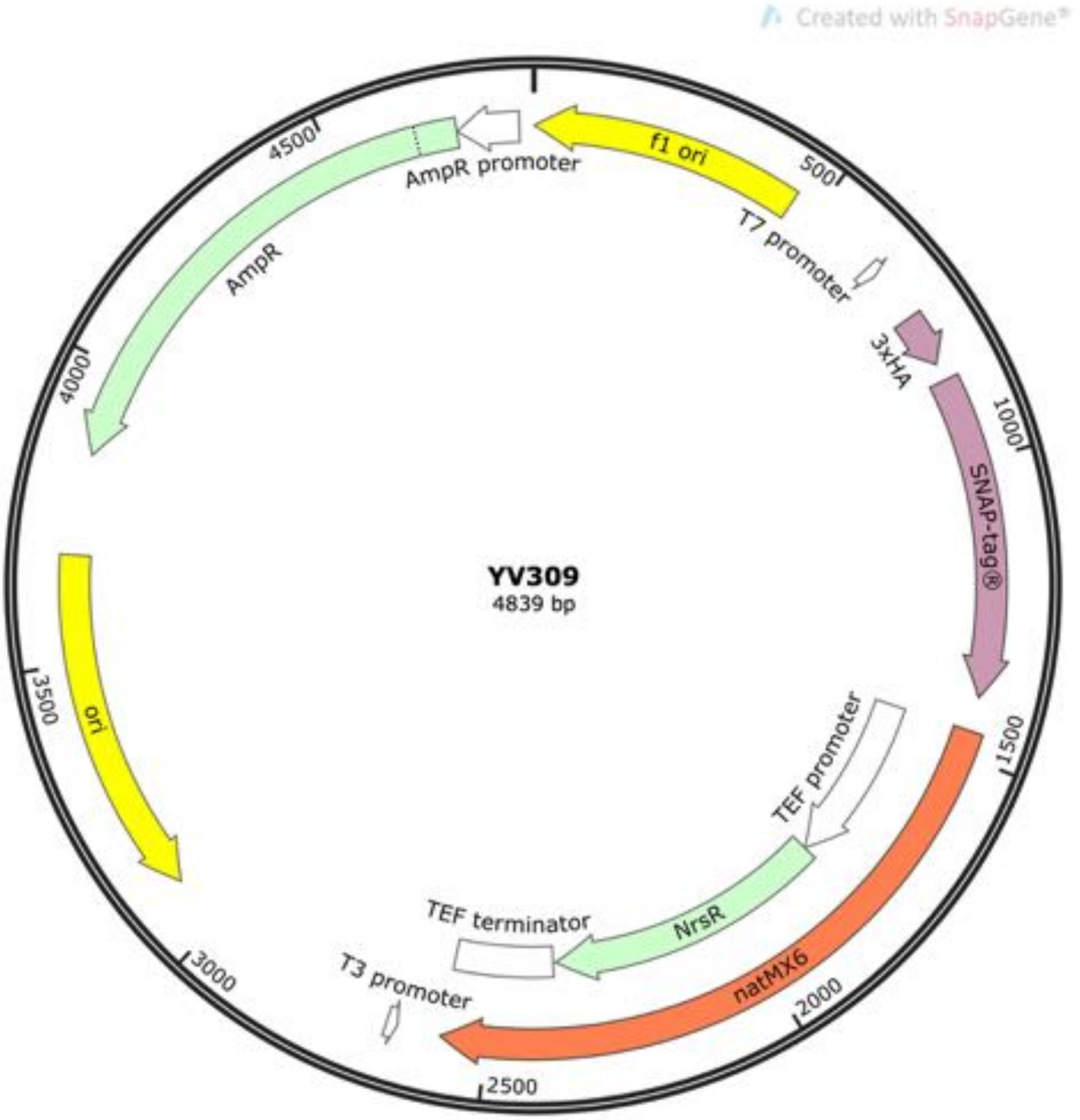
Created with SnapGene®
YV309
4839 bp
f1 ori
AmpR promoter
AmpR
T7 promoter
3xHA
SNAP-tag®
TEF promoter
NrsR
TEF terminator
natMX6
T3 promoter
ori
500
1000
1500
2000
2500
3000
3500
4000
4500

**YV309: pBS-SKII-3XHA-fSNAP-NAT**

**ctaaattgtaagcgttaatattttgttaaaattcgcgttaaatttttgttaaatcagctcattttttaaccaataggccgaaatcggcaaa
atcccttataaatcaaaagaatagaccgagatagggttgagtgttgttccagtttggaacaagagtccactattaaagaacgtggact
ccaacgtcaaagggcgaaaaaccgtctatcagggcgatggcccactacgtgaaccatcaccctaatcaagttttttggggtcgaggtg
ccgtaaagcactaaatcggaaccctaaagggagcccccgatttagagcttgacggggaaagccggcgaacgtggcgagaaaggaag
ggaagaaagcgaaaggagcgggcgctagggcgctggcaagtgtagcggtcacgctgcgcgtaaccaccacacccgccgcgcttaatg
cgccgctacagggcgcgtcccattcgccattcaggctgcgcaactgttgggaagggcgatcggtgcgggcctcttcgctattacgccagc
tggcgaaagggggatgtgctgcaaggcgattaagttgggtaacgccagggttttcccagtcacgacgttgtaaaacgacggccagtga
gcgcgcgtaatacgactcactatagggcgaattgggtaccgggccccccctcgaggtcgacggtatcgataagcttgatatcgggtcga
cggatccccgggttaattaacatctttTACCCATACGATGTTCCTGACTATGCGGGCTATCCCTATGACGTCCCGG
ACTATGCAGGATCCTATCCATATGACGTTCCAGATTACGCTGCTCAGTGCggttcaggtggatctggttctATG
GACAAAGACTGCGAAATGAAGCGCACCACCCTGGATAGCCCTCTGGGCAAGCTGGAACTGTCTGGGT
GCGAACAGGGCCTGCACCGTATCATCTTCCTGGGCAAAGGAACATCTGCCGCCGACGCCGTGGAAGT
GCCTGCCCCAGCCGCCGTGCTGGGCGGACCAGAGCCACTGATGCAGGCCACCGCCTGGCTCAACGCCT
ACTTTCACCAGCCTGAGGCCATCGAGGAGTTCCCTGTGCCAGCCCTGCACCACCCAGTGTTCCAGCAGG
AGAGCTTTACCCGCCAGGTGCTGTGGAAACTGCTGAAAGTGGTGAAGTTCGGAGAGGTCATCAGCTA
CAGCCACCTGGCCGCCCTGGCCGGCAATCCCGCCGCCACCGCCGCCGTGAAAACCGCCCTGAGCGGAA
ATCCCGTGCCCATTCTGATCCCCTGCCACCGGGTGGTGCAGGGCGACCTGGACGTGGGGGGCTACGAG
GGCGGGCTCGCCGTGAAAGAGTGGCTGCTGGCCCACGAGGGCCACAGACTGGGCAAGCCTGGGCTG
GGTtaaTATATAACTGTCTAGAAATAAAGAGTATCATCTTTCAAAGGTCACCCGGCCAGCGACATGGA
GGCCCAGAATACCCTCCTTGACAGTCTTGACGTGCGCAGCTCAGGGGCATGATGTGACTGTCGCCCGT
ACATTTAGCCCATACATCCCCATGTATAATCATTTGCATCCATACATTTTGATGGCCGCACGGCGCGAA
GCAAAAATTACGGCTCCTCGCTGCAGACCTGCGAGCAGGGAAACGCTCCCCTCACAGACGCGTTGAAT
TGTCCCCACGCCGCGCCCCTGTAGAGAAATATAAAAGGTTAGGATTTGCCACTGAGGTTCTTCTTTCAT
ATACTTCCTTTTAAAATCTTGCTAGGATACAGTTCTCACATCACATCCGAACATAAACAACCATGGGTA
CCACTCTTGACGACACGGCTTACCGGTACCGCACCAGTGTCCCGGGGGACGCCGAGGCCATCGAGGCA
CTGGATGGGTCCTTCACCACCGACACCGTCTTCCGCGTCACCGCCACCGGGGACGGCTTCACCCTGCGG
GAGGTGCCGGTGGACCCGCCCCTGACCAAGGTGTTCCCCGACGACGAATCGGACGACGAATCGGACG
ACGGGGAGGACGGCGACCCGGACTCCCGGACGTTCGTCGCGTACGGGGACGACGGCGACCTGGCGG
GCTTCGTGGTCGTCTCGTACTCCGGCTGGAACCGCCGGCTGACCGTCGAGGACATCGAGGTCGCCCCG
GAGCACCGGGGGCACGGGGTCGGGCGCGCGTTGATGGGGCTCGCGACGGAGTTCGCCCGCGAGCGG
GGCGCCGGGCACCTCTGGCTGGAGGTCACCAACGTCAACGCACCGGCGATCCACGCGTACCGGCGGA
TGGGGTTCACCCTCTGCGGCCTGGACACCGCCCTGTACGACGGCACCGCCTCGGACGGCGAGCAGGCG
CTCTACATGAGCATGCCCTGCCCCTAATCAGTACTGACAATAAAAAGATTCTTGTTTTCAAGAACTTGT
CATTTGTATAGTTTTTTTATATTGTAGTTGTTCTATTTTAATCAAATGTTAGCGTGATTTATATTTTTTTTC
GCCTCGACATCATCTGCCCAGATGCGAAGTTAAGTGCGCAGAAAGTAATATCATGCGTCAATCGTATG
TGAATGCTGGTCGCTATACTGCTGTCGATTCGATACTAACGCCGCcactagttctagagcggccgccaccgcggtg
gagctccagcttttgttccctttagtgagggttaattgcgcgcttggcgtaatcatggtcatagctgtttcctgtgtgaaattgttatccgct
cacaattccacacaacatacgagccggaagcataaagtgtaaagcctggggtgcctaatgagtgagctaactcacattaattgcgttg
cgctcactgcccgctttccagtcgggaaacctgtcgtgccagctgcattaatgaatcggccaacgcgcggggagaggcggtttgcgtatt
gggcgctcttccgcttcctcgctcactgactcgctgcgctcggtcgttcggctgcggcgagcggtatcagctcactcaaaggcggtaatac
ggttatccacagaatcaggggataacgcaggaaagaacatgtgagcaaaaggccagcaaaaggccaggaaccgtaaaaaggccgc
gttgctggcgtttttccataggctccgcccccctgacgagcatcacaaaaatcgacgctcaagtcagaggtggcgaaacccgacagga**

ctataaagataccaggcgtttccccctggaagctccctcgtgcgctctcctgttccgaccctgccgcttaccggatacctgtccgcctttct
cccttcgggaagcgtggcgctttctcatagctcacgctgtaggtatctcagttcggtgtaggtcgttcgctccaagctgggctgtgtgcac
gaaccccccgttcagcccgaccgctgcgccttatccggtaactatcgtcttgagtccaacccggtaagacacgacttatcgccactggca
gcagccactggtaacaggattagcagagcgaggtatgtaggcggtgctacagagttcttgaagtggtggcctaactacggctacacta
gaaggacagtatttggtatctgcgctctgctgaagccagttaccttcggaaaaagagttggtagctcttgatccggcaaacaaaccacc
gctggtagcggtggtttttttgtttgcaagcagcagattacgcgcagaaaaaaaggatctcaagaagatcctttgatcttttctacgggg
tctgacgctcagtggaacgaaaactcacgttaagggattttggtcatgagattatcaaaaaggatcttcacctagatccttttaaattaa
aaatgaagttttaaatcaatctaaagtatatatgagtaaacttggtctgacagttaccaatgcttaatcagtgaggcacctatctcagc
gatctgtctatttcgttcatccatagttgcctgactccccgtcgtgtagataactacgatacgggagggcttaccatctggccccagtgct
gcaatgataccgcgagacccacgctcaccggctccagatttatcagcaataaaccagccagccggaagggccgagcgcagaagtggt
cctgcaactttatccgcctccatccagtctattaattgttgccgggaagctagagtaagtagttcgccagttaatagtttgcgcaacgttg
ttgccattgctacaggcatcgtggtgtcacgctcgtcgtttggtatggcttcattcagctccggttcccaacgatcaaggcgagttacatg
atcccccatgttgtgcaaaaaagcggttagctccttcggtcctccgatcgttgtcagaagtaagttggccgcagtgttatcactcatggtt
atggcagcactgcataattctcttactgtcatgccatccgtaagatgcttttctgtgactggtgagtactcaaccaagtcattctgagaat
agtgtatgcggcgaccgagttgctcttgcccggcgtcaatacgggataataccgcgccacatagcagaactttaaaagtgctcatcatt
ggaaaacgttcttcggggcgaaaactctcaaggatcttaccgctgttgagatccagttcgatgtaacccactcgtgcacccaactgatct
tcagcatcttttactttcaccagcgtttctgggtgagcaaaaacaggaaggcaaaatgccgcaaaaaagggaataagggcgacacgg
aaatgttgaatactcatactcttcctttttcaatattattgaagcatttatcagggttattgtctcatgagcggatacatatttgaatgtatt
tagaaaaataaacaaataggggttccgcgcacatttccccgaaaagtgccac

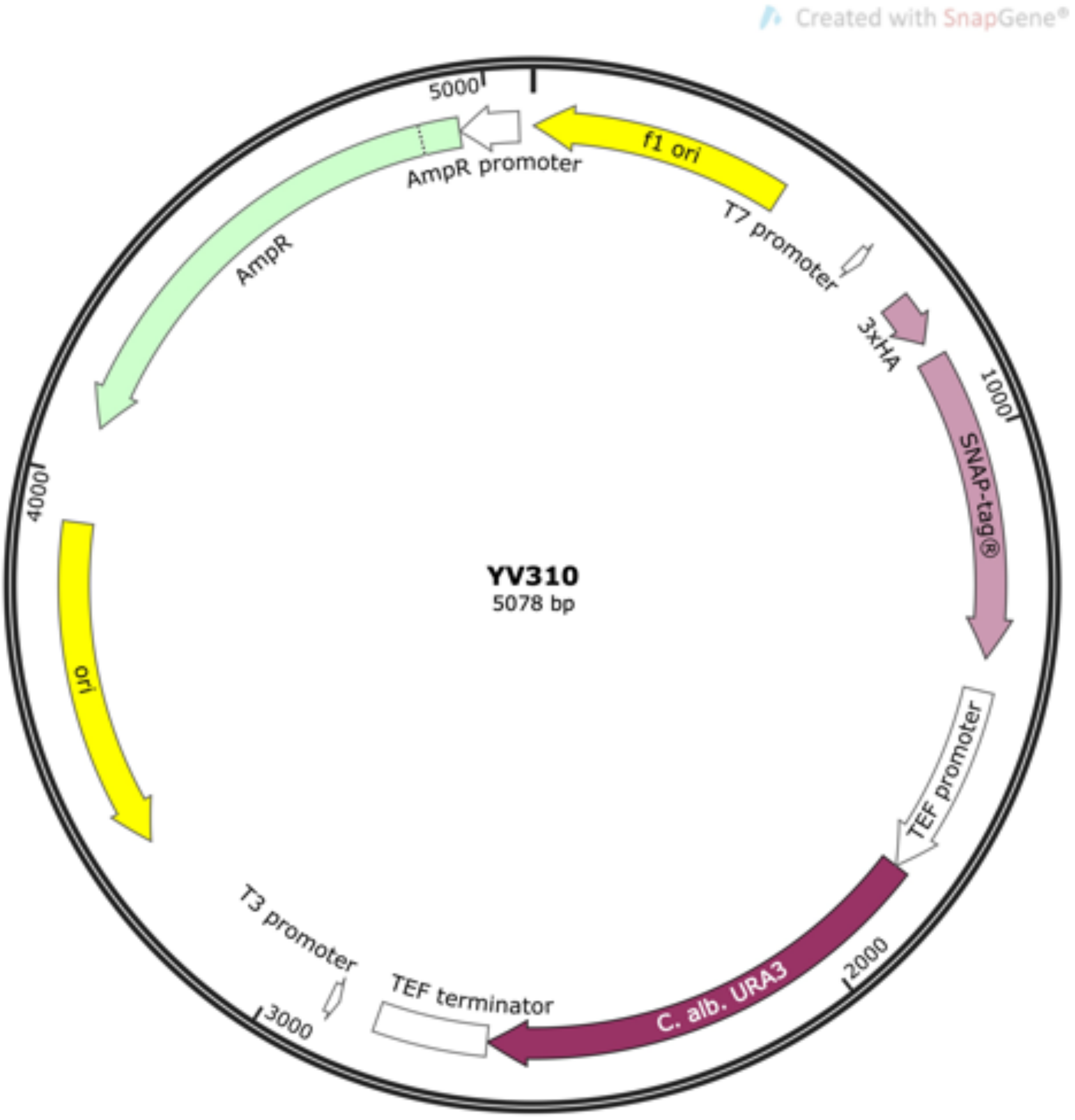

Created with SnapGene®
YV310
5078 bp
f1 ori
T7 promoter
3xHA
SNAP-tag®
TEF promoter
C. alb. URA3
TEF terminator
T3 promoter
ori
AmpR
AmpR promoter
1000
2000
3000
4000
5000

**YV310: pBS-SKII-3XHA-fSNAP-URA**

**ctaaattgtaagcgttaatattttgttaaaattcgcgttaaatttttgttaaatcagctcattttttaaccaataggccgaaatcggcaaa
atcccttataaatcaaaagaatagaccgagatagggttgagtgttgttccagtttggaacaagagtccactattaaagaacgtggact
ccaacgtcaaagggcgaaaaaccgtctatcagggcgatggcccactacgtgaaccatcaccctaatcaagttttttggggtcgaggtg
ccgtaaagcactaaatcggaaccctaaagggagcccccgatttagagcttgacggggaaagccggcgaacgtggcgagaaaggaag
ggaagaaagcgaaaggagcgggcgctagggcgctggcaagtgtagcggtcacgctgcgcgtaaccaccacacccgccgcgcttaatg
cgccgctacagggcgcgtcccattcgccattcaggctgcgcaactgttgggaagggcgatcggtgcgggcctcttcgctattacgccagc
tggcgaaagggggatgtgctgcaaggcgattaagttgggtaacgccagggttttcccagtcacgacgttgtaaaacgacggccagtga
gcgcgcgtaatacgactcactatagggcgaattgggtaccgggccccccctcgaggtcgacggtatcgataagcttgatatcgggtcga
cggatccccgggttaattaacatctttTACCCATACGATGTTCCTGACTATGCGGGCTATCCCTATGACGTCCCGG
ACTATGCGGGATCCTATCCATATGACGTTCCAGATTACGCTGCTCAGTGCGGTTCAGGTGGATCTGGTT
CTATGGACAAAGACTGCGAAATGAAGCGCACCACCCTGGATAGCCCTCTGGGCAAGCTGGAACTGTCT
GGGTGCGAACAGGGCCTGCACCGTATCATCTTCCTGGGCAAAGGAACATCTGCCGCCGACGCCGTGG
AAGTGCCTGCCCCAGCCGCCGTGCTGGGCGGACCAGAGCCACTGATGCAGGCCACCGCCTGGCTCAAC
GCCTACTTTCACCAGCCTGAGGCCATCGAGGAGTTCCCTGTGCCAGCCCTGCACCACCCAGTGTTCCAG
CAGGAGAGCTTTACCCGCCAGGTGCTGTGGAAACTGCTGAAAGTGGTGAAGTTCGGAGAGGTCATCA
GCTACAGCCACCTGGCCGCCCTGGCCGGCAATCCCGCCGCCACCGCCGCCGTGAAAACCGCCCTGAGC
GGAAATCCCGTGCCCATTCTGATCCCCTGCCACCGGGTGGTGCAGGGCGACCTGGACGTGGGGGGCT
ACGAGGGCGGGCTCGCCGTGAAAGAGTGGCTGCTGGCCCACGAGGGCCACAGACTGGGCAAGCCTG
GGCTGGGTTAATATATAACTGTCTAGAAATAAAGAGTATCATCTTTCAAAGGTCACCCGGCCAGCGAC
ATGGAGGCCCAGAATACCCTCCTTGACAGTCTTGACGTGCGCAGCTCAGGGGCATGATGTGACTGTCG
CCCGTACATTTAGCCCATACATCCCCATGTATAATCATTTGCATCCATACATTTTGATGGCCGCACGGCG
CGAAGCAAAAATTACGGCTCCTCGCTGCAGACCTGCGAGCAGGGAAACGCTCCCCTCACAGACGCGTT
GAATTGTCCCCACGCCGCGCCCCTGTAGAGAAATATAAAAGGTTAGGATTTGCCACTGAGGTTCTTCTT
TCATATACTTCCTTTTAAAATCTTGCTAGGATACAGTTCTCACATCACATCCGAACATAAACAACCATGA
CAGTCAACACTAAGACCTATAGTGAGAGAGCAGAAACTCATGCCTCACCAGTAGCACAACGATTATTT
CGATTAATGGAACTGAAGAAAACCAATTTATGTGCATCAATTGATGTTGATACCACTAAGGAATTCCT
TGAATTAATTGATAAATTGGGTCCTTATGTATGCTTAATCAAGACTCATATTGATATAATCAATGATTTT
TCCTATGAATCCACTATTGAACCATTATTAGAACTTTCACGTAAACATCAATTTATGATTTTTGAAGATA
GAAAATTTGCTGATATTGGTAATACCGTGAAGAAACAATATATTGGTGGAGTTTATAAAATTAGTAGT
TGGGCAGATATTACTAATGCTCATGGTGTCACTGGGAATGGAGTAGTTGAAGGATTAAAACAGGGAG
CTAAAGAAACCACCACCAACCAAGAGCCAAGAGGGTTATTGATGTTAGCTGAATTATCATCAGTGGG
ATCATTAGCATATGGAGAATATTCTCAAAAAACTGTTGAAATTGCTAAATCCGATAAGGAATTTGTTAT
TGGATTTATTGCCCAACGTGATATGGGTGGACAAGAAGAAGGATTTGATTGGCTTATTATGACACCTG
GAGTTGGATTAGATGATAAAGGTGATGGATTAGGACAACAATATAGAACTGTTGATGAAGTTGTTAG
CACTGGAACTGATATTATCATTGTTGGTAGAGGATTGTTTGGTAAAGGAAGAGATCCAGATATTGAA
GGTAAAAGGTATAGAGATGCTGGTTGGAATGCTTATTTGAAAAAGACTGGCCAATTATAATCAGTACT
GACAATAAAAAGATTCTTGTTTTCAAGAACTTGTCATTTGTATAGTTTTTTTATATTGTAGTTGTTCTATT
TTAATCAAATGTTAGCGTGATTTATATTTTTTTTCGCCTCGACATCATCTGCCCAGATGCGAAGTTAAGT
GCGCAGAAAGTAATATCATGCGTCAATCGTATGTGAATGCTGGTCGCTATACTGCTGTCGATTCGATA
CTAACGCGCcactagttctagagcggccgccaccgcggtggagctccagcttttgttccctttagtgagggttaattgcgcgcttggc
gtaatcatggtcatagctgtttcctgtgtgaaattgttatccgctcacaattccacacaacatacgagccggaagcataaagtgtaaag
cctggggtgcctaatgagtgagctaactcacattaattgcgttgcgctcactgcccgctttccagtcgggaaacctgtcgtgccagctgc**

**attaatgaatcggccaacgcgcggggagaggcggtttgcgtattgggcgctcttccgcttcctcgctcactgactcgctgcgctcggtcgt
tcggctgcggcgagcggtatcagctcactcaaaggcggtaatacggttatccacagaatcaggggataacgcaggaaagaacatgtg
agcaaaaggccagcaaaaggccaggaaccgtaaaaaggccgcgttgctggcgtttttccataggctccgcccccctgacgagcatcac
aaaaatcgacgctcaagtcagaggtggcgaaacccgacaggactataaagataccaggcgtttccccctggaagctccctcgtgcgct
ctcctgttccgaccctgccgcttaccggatacctgtccgcctttctcccttcgggaagcgtggcgctttctcatagctcacgctgtaggtatc
tcagttcggtgtaggtcgttcgctccaagctgggctgtgtgcacgaaccccccgttcagcccgaccgctgcgccttatccggtaactatcg
tcttgagtccaacccggtaagacacgacttatcgccactggcagcagccactggtaacaggattagcagagcgaggtatgtaggcggt
gctacagagttcttgaagtggtggcctaactacggctacactagaaggacagtatttggtatctgcgctctgctgaagccagttaccttc
ggaaaaagagttggtagctcttgatccggcaaacaaaccaccgctggtagcggtggtttttttgtttgcaagcagcagattacgcgcag
aaaaaaaggatctcaagaagatcctttgatcttttctacggggtctgacgctcagtggaacgaaaactcacgttaagggattttggtca
tgagattatcaaaaaggatcttcacctagatccttttaaattaaaaatgaagttttaaatcaatctaaagtatatatgagtaaacttggt
ctgacagttaccaatgcttaatcagtgaggcacctatctcagcgatctgtctatttcgttcatccatagttgcctgactccccgtcgtgtag
ataactacgatacgggagggcttaccatctggccccagtgctgcaatgataccgcgagacccacgctcaccggctccagatttatcagc
aataaaccagccagccggaagggccgagcgcagaagtggtcctgcaactttatccgcctccatccagtctattaattgttgccgggaag
ctagagtaagtagttcgccagttaatagtttgcgcaacgttgttgccattgctacaggcatcgtggtgtcacgctcgtcgtttggtatggc
ttcattcagctccggttcccaacgatcaaggcgagttacatgatcccccatgttgtgcaaaaaagcggttagctccttcggtcctccgatc
gttgtcagaagtaagttggccgcagtgttatcactcatggttatggcagcactgcataattctcttactgtcatgccatccgtaagatgct
tttctgtgactggtgagtactcaaccaagtcattctgagaatagtgtatgcggcgaccgagttgctcttgcccggcgtcaatacgggata
ataccgcgccacatagcagaactttaaaagtgctcatcattggaaaacgttcttcggggcgaaaactctcaaggatcttaccgctgttg
agatccagttcgatgtaacccactcgtgcacccaactgatcttcagcatcttttactttcaccagcgtttctgggtgagcaaaaacagga
aggcaaaatgccgcaaaaaagggaataagggcgacacggaaatgttgaatactcatactcttcctttttcaatattattgaagcattta
tcagggttattgtctcatgagcggatacatatttgaatgtatttagaaaaataaacaaataggggttccgcgcacatttccccgaaaag
tgccac**

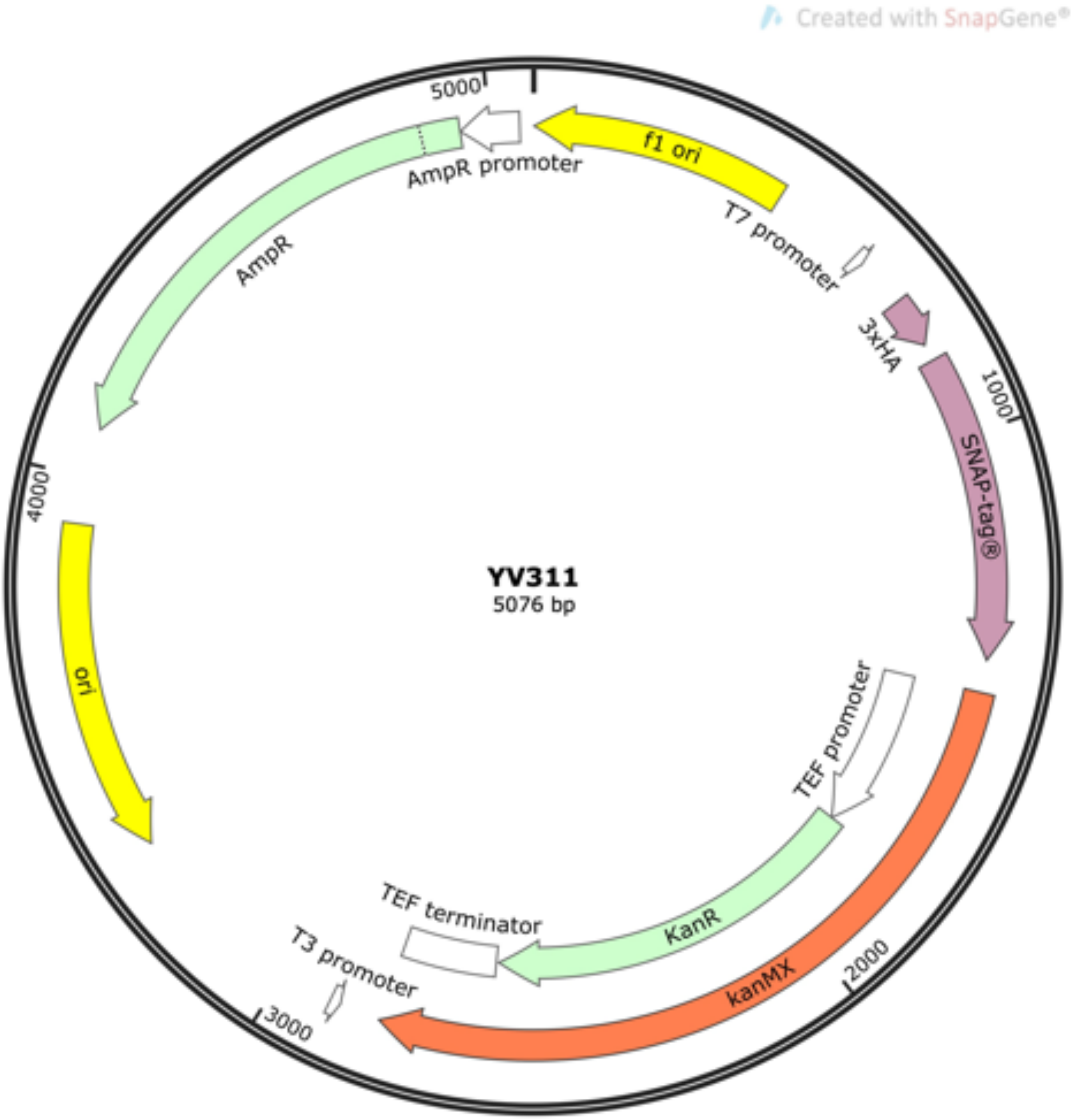

Created with SnapGene®
YV311
5076 bp
f1 ori
AmpR promoter
AmpR
T7 promoter
3xHA
SNAP-tag®
1000
5000
4000
ori
TEF promoter
KanR
kanMX
TEF terminator
T3 promoter
2000
3000

**YV311: pBS-SKII-3XHA-fSNAP-Kan**

ctaaattgtaagcgttaatattttgttaaaattcgcgttaaatttttgttaaatcagctcattttttaaccaataggccgaaatcggcaaa
atcccttataaatcaaaagaatagaccgagatagggttgagtgttgttccagtttggaacaagagtccactattaaagaacgtggact
ccaacgtcaaagggcgaaaaaccgtctatcagggcgatggcccactacgtgaaccatcaccctaatcaagttttttggggtcgaggtg
ccgtaaagcactaaatcggaaccctaaagggagcccccgatttagagcttgacggggaaagccggcgaacgtggcgagaaaggaag
ggaagaaagcgaaaggagcgggcgctagggcgctggcaagtgtagcggtcacgctgcgcgtaaccaccacacccgccgcgcttaatg
cgccgctacagggcgcgtcccattcgccattcaggctgcgcaactgttgggaagggcgatcggtgcgggcctcttcgctattacgccagc
tggcgaaagggggatgtgctgcaaggcgattaagttgggtaacgccagggttttcccagtcacgacgttgtaaaacgacggccagtga
gcgcgcgtaatacgactcactatagggcgaattgggtaccgggccccccctcgaggtcgacggtatcgataagcttgatatcgggtcga
cggatccccgggttaattaacatctttTACCCATACGATGTTCCTGACTATGCGGGCTATCCCTATGACGTCCCGG
ACTATGCAGGATCCTATCCATATGACGTTCCAGATTACGCTGCTCAGTGCggttcaggtggatctggttctATG
GACAAAGACTGCGAAATGAAGCGCACCACCCTGGATAGCCCTCTGGGCAAGCTGGAACTGTCTGGGT
GCGAACAGGGCCTGCACCGTATCATCTTCCTGGGCAAAGGAACATCTGCCGCCGACGCCGTGGAAGT
GCCTGCCCCAGCCGCCGTGCTGGGCGGACCAGAGCCACTGATGCAGGCCACCGCCTGGCTCAACGCCT
ACTTTCACCAGCCTGAGGCCATCGAGGAGTTCCCTGTGCCAGCCCTGCACCACCCAGTGTTCCAGCAGG
AGAGCTTTACCCGCCAGGTGCTGTGGAAACTGCTGAAAGTGGTGAAGTTCGGAGAGGTCATCAGCTA
CAGCCACCTGGCCGCCCTGGCCGGCAATCCCGCCGCCACCGCCGCCGTGAAAACCGCCCTGAGCGGAA
ATCCCGTGCCCATTCTGATCCCCTGCCACCGGGTGGTGCAGGGCGACCTGGACGTGGGGGGCTACGAG
GGCGGGCTCGCCGTGAAAGAGTGGCTGCTGGCCCACGAGGGCCACAGACTGGGCAAGCCTGGGCTG
GGTtaaTATATAACTGTCTAGAAATAAAGAGTATCATCTTTCAAAGGTCACCCGGCCAGCGACATGGA
GGCCCAGAATACCCTCCTTGACAGTCTTGACGTGCGCAGCTCAGGGGCATGATGTGACTGTCGCCCGT
ACATTTAGCCCATACATCCCCATGTATAATCATTTGCATCCATACATTTTGATGGCCGCACGGCGCGAA
GCAAAAATTACGGCTCCTCGCTGCAGACCTGCGAGCAGGGAAACGCTCCCCTCACAGACGCGTTGAAT
TGTCCCCACGCCGCGCCCCTGTAGAGAAATATAAAAAGTTAGGATTTGCCACTGAGGTTCTTCTTTCAT
ATACTTCCTTTTAAAATCTTGCTAGGATACAGTTCTCACATCACATCCGAACATAAACAACCATGGGTA
AGGAAAAGACTCACGTTTCGAGGCCGCGATTAAATTCCAACATGGATGCTGATTTATATGGGTATAAA
TGGGCTCGCGATAATGTCGGGCAATCAGGTGCGACAATCTATCGATTGTATGGGAAGCCCGATGCGC
CAGAGTTGTTTCTGAAACATGGCAAAGGTAGCGTTGCCAATGATGTTACAGATGAGATGGTCAGACT
AAACTGGCTGACGGAATTTATGCCTCTTCCGACCATCAAGCATTTTATCCGTACTCCTGATGATGCATG
GTTACTCACCACTGCGATCCCCGGCAAAACAGCATTCCAGGTATTAGAAGAATATCCTGATTCAGGTG
AAAATATTGTTGATGCGCTGGCAGTGTTCCTGCGCCGGTTGCATTCGATTCCTGTTTGTAATTGTCCTTT
TAACAGCGATCGCGTATTTCGTCTCGCTCAGGCGCAATCACGAATGAATAACGGTTTGGTTGATGCGA
GTGATTTTGATGACGAGCGTAATGGCTGGCCTGTTGAACAAGTCTGGAAAGAAATGCATAAGCTTTTG
CCATTCTCACCGGATTCAGTCGTCACTCATGGTGATTTCTCACTTGATAACCTTATTTTTGACGAGGGGA
AATTAATAGGTTGTATTGATGTTGGACGAGTCGGAATCGCAGACCGATACCAGGATCTTGCCATCCTA
TGGAACTGCCTCGGTGAGTTTTCTCCTTCATTACAGAAACGGCTTTTTCAAAAATATGGTATTGATAAT
CCTGATATGAATAAATTGCAGTTTCATTTGATGCTCGATGAGTTTTTCTAATCAGTACTGACAATAAAA
AGATTCTTGTTTTCAAGAACTTGTCATTTGTATAGTTTTTTTATATTGTAGTTGCTCTATTTTAATCAAAT
GTTAGCGTGATTTATATTTTTTTTCGCCTCGACATCATCTGCCCAGATGCGAAGTTAAGTGCGCAGAAA
GTAATATCATGCGTCAATCGTATGTGAATGCTGGTCGCTATACTGCTGTCGATTCGATACTAACGCCGC
cactagttctagagcggccgccaccgcggtggagctccagcttttgttccctttagtgagggttaattgcgcgcttggcgtaatcatggtc
atagctgtttcctgtgtgaaattgttatccgctcacaattccacacaacatacgagccggaagcataaagtgtaaagcctggggtgcct
aatgagtgagctaactcacattaattgcgttgcgctcactgcccgctttccagtcgggaaacctgtcgtgccagctgcattaatgaatcg

**gccaacgcgcggggagaggcggtttgcgtattgggcgctcttccgcttcctcgctcactgactcgctgcgctcggtcgttcggctgcggcg
agcggtatcagctcactcaaaggcggtaatacggttatccacagaatcaggggataacgcaggaaagaacatgtgagcaaaaggcc
agcaaaaggccaggaaccgtaaaaaggccgcgttgctggcgtttttccataggctccgcccccctgacgagcatcacaaaaatcgacg
ctcaagtcagaggtggcgaaacccgacaggactataaagataccaggcgtttccccctggaagctccctcgtgcgctctcctgttccga
ccctgccgcttaccggatacctgtccgcctttctcccttcgggaagcgtggcgctttctcatagctcacgctgtaggtatctcagttcggtg
taggtcgttcgctccaagctgggctgtgtgcacgaaccccccgttcagcccgaccgctgcgccttatccggtaactatcgtcttgagtcca
acccggtaagacacgacttatcgccactggcagcagccactggtaacaggattagcagagcgaggtatgtaggcggtgctacagagtt
cttgaagtggtggcctaactacggctacactagaaggacagtatttggtatctgcgctctgctgaagccagttaccttcggaaaaagag
ttggtagctcttgatccggcaaacaaaccaccgctggtagcggtggtttttttgtttgcaagcagcagattacgcgcagaaaaaaagga
tctcaagaagatcctttgatcttttctacggggtctgacgctcagtggaacgaaaactcacgttaagggattttggtcatgagattatca
aaaaggatcttcacctagatccttttaaattaaaaatgaagttttaaatcaatctaaagtatatatgagtaaacttggtctgacagttac
caatgcttaatcagtgaggcacctatctcagcgatctgtctatttcgttcatccatagttgcctgactccccgtcgtgtagataactacgat
acgggagggcttaccatctggccccagtgctgcaatgataccgcgagacccacgctcaccggctccagatttatcagcaataaaccag
ccagccggaagggccgagcgcagaagtggtcctgcaactttatccgcctccatccagtctattaattgttgccgggaagctagagtaag
tagttcgccagttaatagtttgcgcaacgttgttgccattgctacaggcatcgtggtgtcacgctcgtcgtttggtatggcttcattcagct
ccggttcccaacgatcaaggcgagttacatgatcccccatgttgtgcaaaaaagcggttagctccttcggtcctccgatcgttgtcagaa
gtaagttggccgcagtgttatcactcatggttatggcagcactgcataattctcttactgtcatgccatccgtaagatgcttttctgtgact
ggtgagtactcaaccaagtcattctgagaatagtgtatgcggcgaccgagttgctcttgcccggcgtcaatacgggataataccgcgcc
acatagcagaactttaaaagtgctcatcattggaaaacgttcttcggggcgaaaactctcaaggatcttaccgctgttgagatccagtt
cgatgtaacccactcgtgcacccaactgatcttcagcatcttttactttcaccagcgtttctgggtgagcaaaaacaggaaggcaaaat
gccgcaaaaaagggaataagggcgacacggaaatgttgaatactcatactcttcctttttcaatattattgaagcatttatcagggttat
tgtctcatgagcggatacatatttgaatgtatttagaaaaataaacaaataggggttccgcgcacatttccccgaaaagtgccac**

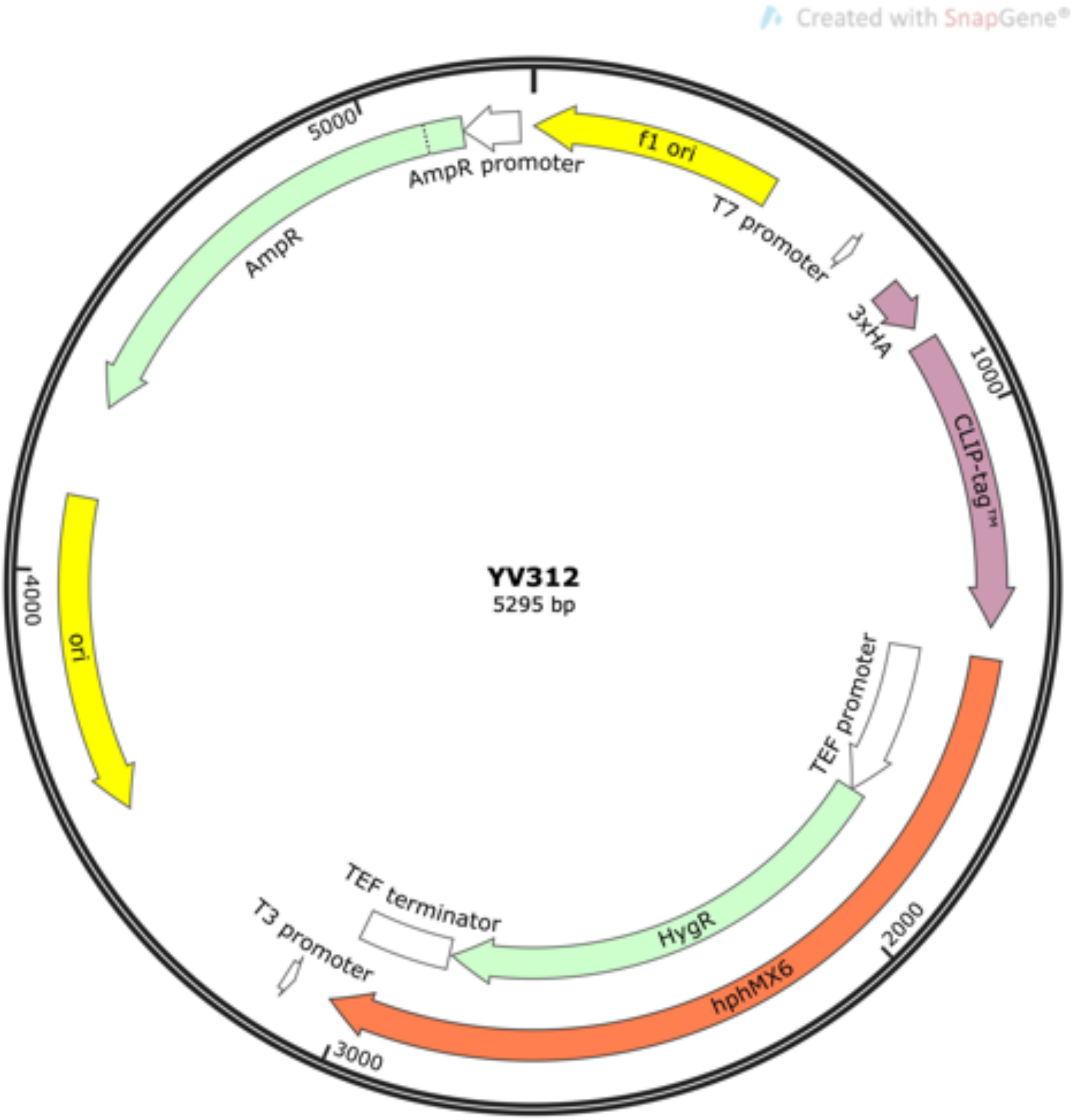
Created with SnapGene®
YV312
5295 bp
f1 ori
AmpR promoter
T7 promoter
AmpR
3xHA
1000
CLIP-tag™
5000
4000
ori
TEF promoter
HygR
TEF terminator
T3 promoter
hphMX6
2000
3000

**YV312: pBS-SKII-3XHA-fCLIP-Hygromycin**

**ctaaattgtaagcgttaatattttgttaaaattcgcgttaaatttttgttaaatcagctcattttttaaccaataggccgaaatcggcaaa
atcccttataaatcaaaagaatagaccgagatagggttgagtgttgttccagtttggaacaagagtccactattaaagaacgtggact
ccaacgtcaaagggcgaaaaaccgtctatcagggcgatggcccactacgtgaaccatcaccctaatcaagttttttggggtcgaggtg
ccgtaaagcactaaatcggaaccctaaagggagcccccgatttagagcttgacggggaaagccggcgaacgtggcgagaaaggaag
ggaagaaagcgaaaggagcgggcgctagggcgctggcaagtgtagcggtcacgctgcgcgtaaccaccacacccgccgcgcttaatg
cgccgctacagggcgcgtcccattcgccattcaggctgcgcaactgttgggaagggcgatcggtgcgggcctcttcgctattacgccagc
tggcgaaagggggatgtgctgcaaggcgattaagttgggtaacgccagggttttcccagtcacgacgttgtaaaacgacggccagtga
gcgcgcgtaatacgactcactatagggcgaattgggtaccgggccccccctcgaggtcgacggtatcgataagcttgatatcgggtcga
cggatccccgggttaattaacatctttTACCCATACGATGTTCCTGACTATGCGGGCTATCCCTATGACGTCCCGG
ACTATGCAGGATCCTATCCATATGACGTTCCAGATTACGCTGCTCAGTGCggttcaggtggatctggttctATG
GACAAAGACTGCGAAATGAAGCGCACCACCCTGGATAGCCCTCTGGGCAAGCTGGAACTGTCTGGGT
GCGAACAGGGCCTGCACCGTATCATCTTCCTGGGCAAAGGAACATCTGCCGCCGACGCCGTGGAAGT
GCCTGCCCCAGCCGCCGTGCTGGGCGGACCAGAGCCACTGATCCAGGCCACCGCCTGGCTCAACGCCT
ACTTTCACCAGCCTGAGGCCATCGAGGAGTTCCCTGTGCCAGCCCTGCACCACCCAGTGTTCCAGCAGG
AGAGCTTTACCCGCCAGGTGCTGTGGAAACTGCTGAAAGTGGTGAAGTTCGGAGAGGTCATCAGCGA
GAGCCACCTGGCCGCCCTGGTGGGCAATCCCGCCGCCACCGCCGCCGTGAACACCGCCCTGGACGGAA
ATCCCGTGCCCATTCTGATCCCCTGCCACCGGGTGGTGCAGGGCGACAGCGACGTGGGGCCCTACCTG
GGCGGGCTCGCCGTGAAAGAGTGGCTGCTGGCCCACGAGGGCCACAGACTGGGCAAGCCTGGGCTG
GGTtaaTATATAACTGTCTAGAAATAAAGAGTATCATCTTTCAAAGGTCACCCGGCCAGCGACATGGA
GGCCCAGAATACCCTCCTTGACAGTCTTGACGTGCGCAGCTCAGGGGCATGATGTGACTGTCGCCCGT
ACATTTAGCCCATACATCCCCATGTATAATCATTTGCATCCATACATTTTGATGGCCGCACGGCGCGAA
GCAAAAATTACGGCTCCTCGCTGCGGACCTGCGAGCAGGGAAACGCTCCCCTCACAGACGCGTTGAAT
TGTCCCCACGCCGCGCCCCTGTAGAGAAATATAAAAGGTTAGGATTTGCCACTGAGGTTCTTCTTTCAT
ATACTTCCTTTTAAAATCTTGCTAGGATACAGTTCTCACATCACATCCGAACATAAACAACCATGGGTA
AAAAGCCTGAACTCACCGCGACGTCTGTCGAGAAGTTTCTGATCGAAAAGTTCGACAGCGTCTCCGAC
CTGATGCAGCTCTCGGAGGGCGAAGAATCTCGTGCTTTCAGCTTCGATGTAGGAGGGCGTGGATATGT
CCTGCGGGTAAATAGCTGCGCCGATGGTTTCTACAAAGATCGTTATGTTTATCGGCACTTTGCATCGGC
CGCGCTCCCGATTCCGGAAGTGCTTGACATTGGGGAATTCAGCGAGAGCCTGACCTATTGCATCTCCC
GCCGTGCACAGGGTGTCACGTTGCAAGACCTGCCTGAAACCGAACTGCCCGCTGTTCTGCAGCCGGTC
GCGGAGGCCATGGATGCGATCGCTGCGGCCGATCTTAGCCAGACGAGCGGGTTCGGCCCATTCGGAC
CGCAAGGAATCGGTCAATACACTACATGGCGTGATTTCATATGCGCGATTGCTGATCCCCATGTGTATC
ACTGGCAAACTGTGATGGACGACACCGTCAGTGCGTCCGTCGCGCAGGCTCTCGATGAGCTGATGCTT
TGGGCCGAGGACTGCCCCGAAGTCCGGCACCTCGTGCACGCGGATTTCGGCTCCAACAATGTCCTGAC
GGACAATGGCCGCATAACAGCGGTCATTGACTGGAGCGAGGCGATGTTCGGGGATTCCCAATACGAG
GTCGCCAACATCTTCTTCTGGAGGCCGTGGTTGGCTTGTATGGAGCAGCAGACGCGCTACTTCGAGCG
GAGGCATCCGGAGCTTGCAGGATCGCCGCGGCTCCGGGCGTATATGCTCCGCATTGGTCTTGACCAAC
TCTATCAGAGCTTGGTTGACGGCAATTTCGATGATGCAGCTTGGGCGCAGGGTCGATGCGACGCAATC
GTCCGATCCGGAGCCGGGACTGTCGGGCGTACACAAATCGCCCGCAGAAGCGCGGCCGTCTGGACCG
ATGGCTGTGTAGAAGTACTCGCCGATAGTGGAAACCGACGCCCCAGCACTCGTCCGAGGGCAAAGGA
ATAATCAGTACTGACAATAAAAAGATTCTTGTTTTCAAGAACTTGTCATTTGTATAGTTTTTTTATATTG
TAGTTGTTCTATTTTAATCAAATGTTAGCGTGATTTATATTTTTTTTCGCCTCGACATCATCTGCCCAGAT
GCGAAGTTAAGTGCGCAGAAAGTAATATCATGCGTCAATCGTATGTGAATGCTGGTCGCTATACTGCT**

**GTCGATTCGATACTAACGCCGCcactagttctagagcggccgccaccgcggtggagctccagcttttgttccctttagtgaggg
ttaattgcgcgcttggcgtaatcatggtcatagctgtttcctgtgtgaaattgttatccgctcacaattccacacaacatacgagccggaa
gcataaagtgtaaagcctggggtgcctaatgagtgagctaactcacattaattgcgttgcgctcactgcccgctttccagtcgggaaac
ctgtcgtgccagctgcattaatgaatcggccaacgcgcggggagaggcggtttgcgtattgggcgctcttccgcttcctcgctcactgact
cgctgcgctcggtcgttcggctgcggcgagcggtatcagctcactcaaaggcggtaatacggttatccacagaatcaggggataacgc
aggaaagaacatgtgagcaaaaggccagcaaaaggccaggaaccgtaaaaaggccgcgttgctggcgtttttccataggctccgccc
ccctgacgagcatcacaaaaatcgacgctcaagtcagaggtggcgaaacccgacaggactataaagataccaggcgtttccccctgg
aagctccctcgtgcgctctcctgttccgaccctgccgcttaccggatacctgtccgcctttctcccttcgggaagcgtggcgctttctcatag
ctcacgctgtaggtatctcagttcggtgtaggtcgttcgctccaagctgggctgtgtgcacgaaccccccgttcagcccgaccgctgcgcc
ttatccggtaactatcgtcttgagtccaacccggtaagacacgacttatcgccactggcagcagccactggtaacaggattagcagagc
gaggtatgtaggcggtgctacagagttcttgaagtggtggcctaactacggctacactagaaggacagtatttggtatctgcgctctgct
gaagccagttaccttcggaaaaagagttggtagctcttgatccggcaaacaaaccaccgctggtagcggtggtttttttgtttgcaagc
agcagattacgcgcagaaaaaaaggatctcaagaagatcctttgatcttttctacggggtctgacgctcagtggaacgaaaactcacg
ttaagggattttggtcatgagattatcaaaaaggatcttcacctagatccttttaaattaaaaatgaagttttaaatcaatctaaagtat
atatgagtaaacttggtctgacagttaccaatgcttaatcagtgaggcacctatctcagcgatctgtctatttcgttcatccatagttgcct
gactccccgtcgtgtagataactacgatacgggagggcttaccatctggccccagtgctgcaatgataccgcgagacccacgctcaccg
gctccagatttatcagcaataaaccagccagccggaagggccgagcgcagaagtggtcctgcaactttatccgcctccatccagtctat
taattgttgccgggaagctagagtaagtagttcgccagttaatagtttgcgcaacgttgttgccattgctacaggcatcgtggtgtcacg
ctcgtcgtttggtatggcttcattcagctccggttcccaacgatcaaggcgagttacatgatcccccatgttgtgcaaaaaagcggttag
ctccttcggtcctccgatcgttgtcagaagtaagttggccgcagtgttatcactcatggttatggcagcactgcataattctcttactgtca
tgccatccgtaagatgcttttctgtgactggtgagtactcaaccaagtcattctgagaatagtgtatgcggcgaccgagttgctcttgccc
ggcgtcaatacgggataataccgcgccacatagcagaactttaaaagtgctcatcattggaaaacgttcttcggggcgaaaactctca
aggatcttaccgctgttgagatccagttcgatgtaacccactcgtgcacccaactgatcttcagcatcttttactttcaccagcgtttctgg
gtgagcaaaaacaggaaggcaaaatgccgcaaaaaagggaataagggcgacacggaaatgttgaatactcatactcttcctttttca
atattattgaagcatttatcagggttattgtctcatgagcggatacatatttgaatgtatttagaaaaataaacaaataggggttccgcg
cacatttccccgaaaagtgccac**

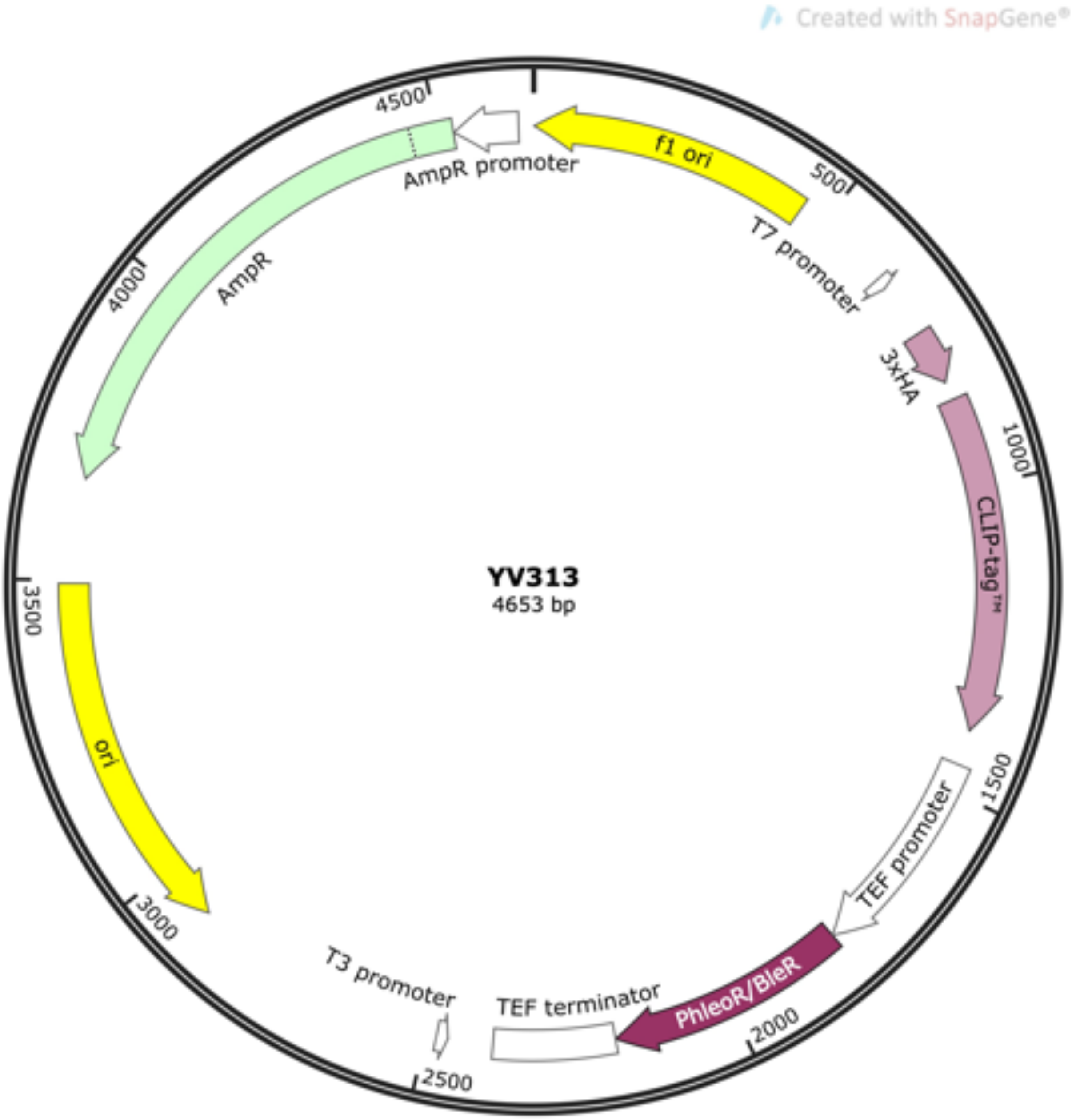
Created with SnapGene®
YV313
4653 bp
f1 ori
500
T7 promoter
3xHA
1000
CLIP-tag™
1500
TEF promoter
PhleoR/BleR
2000
TEF terminator
T3 promoter
2500
3000
ori
3500
4000
AmpR
AmpR promoter
4500

**YV313: pBS-SKII-3XHA-fCLIP-Phleomycin**

**ctaaattgtaagcgttaatattttgttaaaattcgcgttaaatttttgttaaatcagctcattttttaaccaataggccgaaatcggcaaa
atcccttataaatcaaaagaatagaccgagatagggttgagtgttgttccagtttggaacaagagtccactattaaagaacgtggact
ccaacgtcaaagggcgaaaaaccgtctatcagggcgatggcccactacgtgaaccatcaccctaatcaagttttttggggtcgaggtg
ccgtaaagcactaaatcggaaccctaaagggagcccccgatttagagcttgacggggaaagccggcgaacgtggcgagaaaggaag
ggaagaaagcgaaaggagcgggcgctagggcgctggcaagtgtagcggtcacgctgcgcgtaaccaccacacccgccgcgcttaatg
cgccgctacagggcgcgtcccattcgccattcaggctgcgcaactgttgggaagggcgatcggtgcgggcctcttcgctattacgccagc
tggcgaaagggggatgtgctgcaaggcgattaagttgggtaacgccagggttttcccagtcacgacgttgtaaaacgacggccagtga
gcgcgcgtaatacgactcactatagggcgaattgggtaccgggccccccctcgaggtcgacggtatcgataagcttgatatcgggtcga
cggatccccgggttaattaacatctttTACCCATACGATGTTCCTGACTATGCGGGCTATCCCTATGACGTCCCGG
ACTATGCAGGATCCTATCCATATGACGTTCCAGATTACGCTGCTCAGTGCggttcaggtggatctggttctATG
GACAAAGACTGCGAAATGAAGCGCACCACCCTGGATAGCCCTCTGGGCAAGCTGGAACTGTCTGGGT
GCGAACAGGGCCTGCACCGTATCATCTTCCTGGGCAAAGGAACATCTGCCGCCGACGCCGTGGAAGT
GCCTGCCCCAGCCGCCGTGCTGGGCGGACCAGAGCCACTGATCCAGGCCACCGCCTGGCTCAACGCCT
ACTTTCACCAGCCTGAGGCCATCGAGGAGTTCCCTGTGCCAGCCCTGCACCACCCAGTGTTCCAGCAGG
AGAGCTTTACCCGCCAGGTGCTGTGGAAACTGCTGAAAGTGGTGAAGTTCGGAGAGGTCATCAGCGA
GAGCCACCTGGCCGCCCTGGTGGGCAATCCCGCCGCCACCGCCGCCGTGAACACCGCCCTGGACGGAA
ATCCCGTGCCCATTCTGATCCCCTGCCACCGGGTGGTGCAGGGCGACAGCGACGTGGGGCCCTACCTG
GGCGGGCTCGCCGTGAAAGAGTGGCTGCTGGCCCACGAGGGCCACAGACTGGGCAAGCCTGGGCTG
GGTtaaTATATAACTGTCTAGAAATAAAGAGTATCATCTTTCAAAGGTCACCCGGCCAGCGACATGGA
GGCCCAGAATACCCTCCTTGACAGTCTTGACGTGCGCAGCTCAGGGGCATGATGTGACTGTCGCCCGT
ACATTTAGCCCATACATCCCCATGTATAATCATTTGCATCCATACATTTTGATGGCCGCACGGCGCGAA
GCAAAAATTACGGCTCCTCGCTGCAGACCTGCGAGCAGGGAAACGCTCCCCTCACAGACGCGTTGAAT
TGTCCCCACGCCGCGCCCCTGTAGAGAAATATAAAAGGTTAGGATTTGCCACTGAGGTTCTTCTTTCAT
ATACTTCCTTTTAAAATCTTGCTAGGATACAGTTCTCACATCACATCCGAACATAAACAACCATGGGTA
TGACCGACCAAGCGACGCCCAACCTGCCATCACGAGATTTCGATcCCACcGCCGCCTTCTATGAAAGGT
TGGGCTTCGGAATCGTTTTCCGGGACGCCGGCTGGATGATCCTCCAGCGCGGGGATCTCAtGCTGGAG
TTCTTCGCCCACCCCGGGCTCGATCCCCTCGCGAGTTGGTTCAGCTGCTGCCTGAGGCTGGACGACCTC
GCGGAGTTCTACCGGCAGTGCAAATCCGTCGGCATCCAGGAAACCAGCAGCGGCTATCCGCGCATCCA
TGCCCCCGAACTGCAGGAGTGGGGAGGCACGATGGCCGCTTTGGTCGACCCGGACGGGACGCTCCTG
CGCCTGATACAGAACGAATTGCTTGCAGGCATCTCATGATCAGTACTGACAATAAAAAGATTCTTGTTT
TCAAGAACTTGTCATTTGTATAGTTTTTTTATATTGTAGTTGTTCTATTTTAATCAAATGTTAGCGTGATT
TATATTTTTTTTCGCCTCGACATCATCTGCCCAGATGCGAAGTTAAGTGCGCAGAAAGTAATATCATGC
GTCAATCGTATGTGAATGCTGGTCGCTATACTGCTGTCGATTCGATACTAACGCCGCcactagttctagagcg
gccgccaccgcggtggagctccagcttttgttccctttagtgagggttaattgcgcgcttggcgtaatcatggtcatagctgtttcctgtgt
gaaattgttatccgctcacaattccacacaacatacgagccggaagcataaagtgtaaagcctggggtgcctaatgagtgagctaact
cacattaattgcgttgcgctcactgcccgctttccagtcgggaaacctgtcgtgccagctgcattaatgaatcggccaacgcgcggggag
aggcggtttgcgtattgggcgctcttccgcttcctcgctcactgactcgctgcgctcggtcgttcggctgcggcgagcggtatcagctcact
caaaggcggtaatacggttatccacagaatcaggggataacgcaggaaagaacatgtgagcaaaaggccagcaaaaggccaggaa
ccgtaaaaaggccgcgttgctggcgtttttccataggctccgcccccctgacgagcatcacaaaaatcgacgctcaagtcagaggtggc
gaaacccgacaggactataaagataccaggcgtttccccctggaagctccctcgtgcgctctcctgttccgaccctgccgcttaccggat
acctgtccgcctttctcccttcgggaagcgtggcgctttctcatagctcacgctgtaggtatctcagttcggtgtaggtcgttcgctccaag
ctgggctgtgtgcacgaaccccccgttcagcccgaccgctgcgccttatccggtaactatcgtcttgagtccaacccggtaagacacgac**

ttatcgccactggcagcagccactggtaacaggattagcagagcgaggtatgtaggcggtgctacagagttcttgaagtggtggccta
actacggctacactagaaggacagtatttggtatctgcgctctgctgaagccagttaccttcggaaaaagagttggtagctcttgatccg
gcaaacaaaccaccgctggtagcggtggtttttttgtttgcaagcagcagattacgcgcagaaaaaaaggatctcaagaagatccttt
gatcttttctacggggtctgacgctcagtggaacgaaaactcacgttaagggattttggtcatgagattatcaaaaaggatcttcaccta
gatccttttaaattaaaaatgaagttttaaatcaatctaaagtatatatgagtaaacttggtctgacagttaccaatgcttaatcagtga
ggcacctatctcagcgatctgtctatttcgttcatccatagttgcctgactccccgtcgtgtagataactacgatacgggagggcttacca
tctggccccagtgctgcaatgataccgcgagacccacgctcaccggctccagatttatcagcaataaaccagccagccggaagggccg
agcgcagaagtggtcctgcaactttatccgcctccatccagtctattaattgttgccgggaagctagagtaagtagttcgccagttaata
gtttgcgcaacgttgttgccattgctacaggcatcgtggtgtcacgctcgtcgtttggtatggcttcattcagctccggttcccaacgatca
aggcgagttacatgatcccccatgttgtgcaaaaaagcggttagctccttcggtcctccgatcgttgtcagaagtaagttggccgcagtg
ttatcactcatggttatggcagcactgcataattctcttactgtcatgccatccgtaagatgcttttctgtgactggtgagtactcaaccaa
gtcattctgagaatagtgtatgcggcgaccgagttgctcttgcccggcgtcaatacgggataataccgcgccacatagcagaactttaa
aagtgctcatcattggaaaacgttcttcggggcgaaaactctcaaggatcttaccgctgttgagatccagttcgatgtaacccactcgtg
cacccaactgatcttcagcatcttttactttcaccagcgtttctgggtgagcaaaaacaggaaggcaaaatgccgcaaaaaagggaat
aagggcgacacggaaatgttgaatactcatactcttcctttttcaatattattgaagcatttatcagggttattgtctcatgagcggatac
atatttgaatgtatttagaaaaataaacaaataggggttccgcgcacatttccccgaaaagtgccac

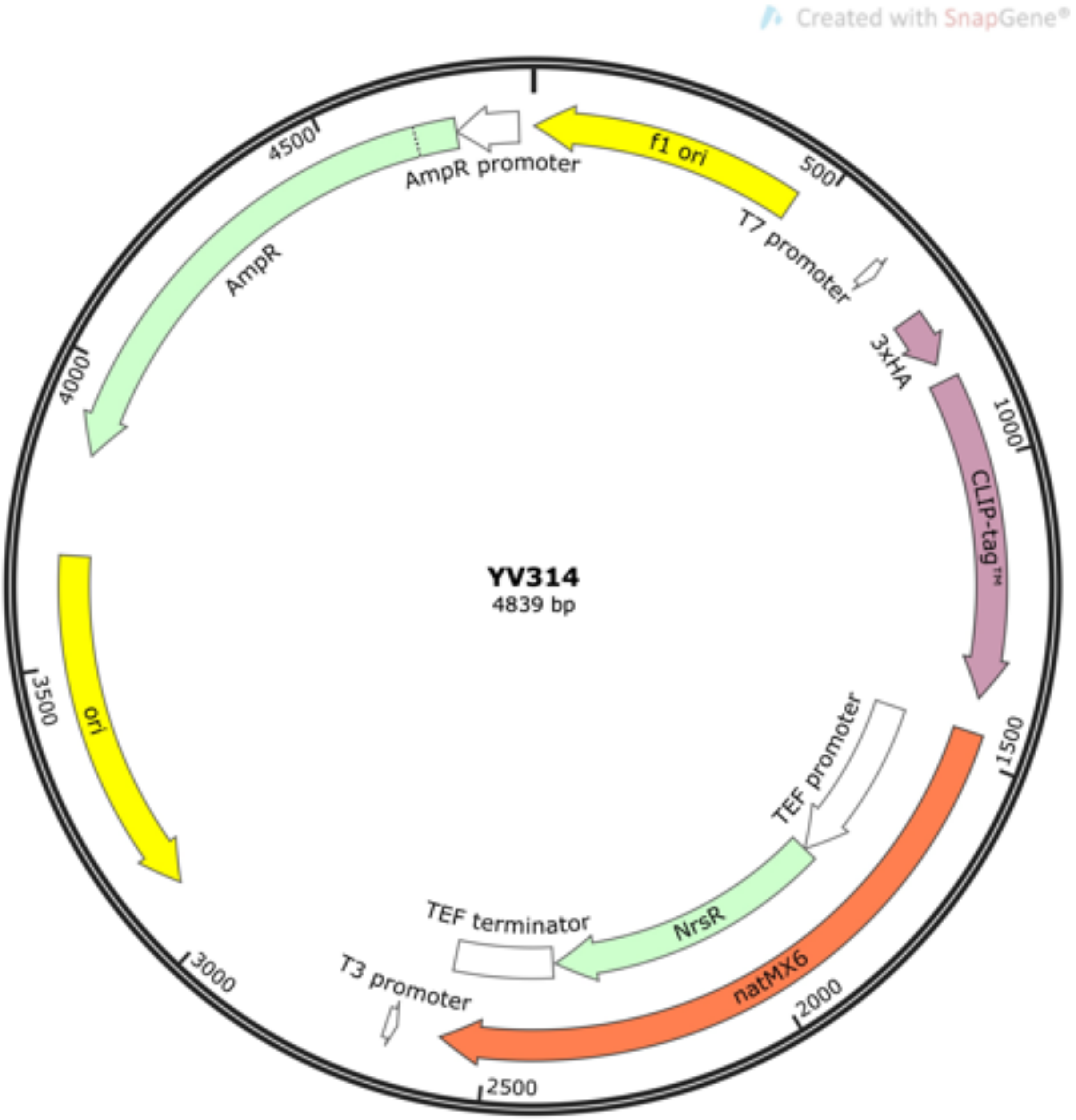
Created with SnapGene®
YV314
4839 bp
f1 ori
500
T7 promoter
3xHA
1000
CLIP-tag™
1500
TEF promoter
NrsR
natMX6
2000
TEF terminator
T3 promoter
2500
3000
ori
3500
4000
AmpR
4500
AmpR promoter

**YV314: pBS-SKII-3XHA-fCLIP-NAT**

**ctaaattgtaagcgttaatattttgttaaaattcgcgttaaatttttgttaaatcagctcattttttaaccaataggccgaaatcggcaaa
atcccttataaatcaaaagaatagaccgagatagggttgagtgttgttccagtttggaacaagagtccactattaaagaacgtggact
ccaacgtcaaagggcgaaaaaccgtctatcagggcgatggcccactacgtgaaccatcaccctaatcaagttttttggggtcgaggtg
ccgtaaagcactaaatcggaaccctaaagggagcccccgatttagagcttgacggggaaagccggcgaacgtggcgagaaaggaag
ggaagaaagcgaaaggagcgggcgctagggcgctggcaagtgtagcggtcacgctgcgcgtaaccaccacacccgccgcgcttaatg
cgccgctacagggcgcgtcccattcgccattcaggctgcgcaactgttgggaagggcgatcggtgcgggcctcttcgctattacgccagc
tggcgaaagggggatgtgctgcaaggcgattaagttgggtaacgccagggttttcccagtcacgacgttgtaaaacgacggccagtga
gcgcgcgtaatacgactcactatagggcgaattgggtaccgggccccccctcgaggtcgacggtatcgataagcttgatatcgggtcga
cggatccccgggttaattaacatctttTACCCATACGATGTTCCTGACTATGCGGGCTATCCCTATGACGTCCCGG
ACTATGCAGGATCCTATCCATATGACGTTCCAGATTACGCTGCTCAGTGCggttcaggtggatctggttctATG
GACAAAGACTGCGAAATGAAGCGCACCACCCTGGATAGCCCTCTGGGCAAGCTGGAACTGTCTGGGT
GCGAACAGGGCCTGCACCGTATCATCTTCCTGGGCAAAGGAACATCTGCCGCCGACGCCGTGGAAGT
GCCTGCCCCAGCCGCCGTGCTGGGCGGACCAGAGCCACTGATCCAGGCCACCGCCTGGCTCAACGCCT
ACTTTCACCAGCCTGAGGCCATCGAGGAGTTCCCTGTGCCAGCCCTGCACCACCCAGTGTTCCAGCAGG
AGAGCTTTACCCGCCAGGTGCTGTGGAAACTGCTGAAAGTGGTGAAGTTCGGAGAGGTCATCAGCGA
GAGCCACCTGGCCGCCCTGGTGGGCAATCCCGCCGCCACCGCCGCCGTGAACACCGCCCTGGACGGAA
ATCCCGTGCCCATTCTGATCCCCTGCCACCGGGTGGTGCAGGGCGACAGCGACGTGGGGCCCTACCTG
GGCGGGCTCGCCGTGAAAGAGTGGCTGCTGGCCCACGAGGGCCACAGACTGGGCAAGCCTGGGCTG
GGTtaaTATATAACTGTCTAGAAATAAAGAGTATCATCTTTCAAAGGTCACCCGGCCAGCGACATGGA
GGCCCAGAATACCCTCCTTGACAGTCTTGACGTGCGCAGCTCAGGGGCATGATGTGACTGTCGCCCGT
ACATTTAGCCCATACATCCCCATGTATAATCATTTGCATCCATACATTTTGATGGCCGCACGGCGCGAA
GCAAAAATTACGGCTCCTCGCTGCAGACCTGCGAGCAGGGAAACGCTCCCCTCACAGACGCGTTGAAT
TGTCCCCACGCCGCGCCCCTGTAGAGAAATATAAAAGGTTAGGATTTGCCACTGAGGTTCTTCTTTCAT
ATACTTCCTTTTAAAATCTTGCTAGGATACAGTTCTCACATCACATCCGAACATAAACAACCATGGGTA
CCACTCTTGACGACACGGCTTACCGGTACCGCACCAGTGTCCCGGGGGACGCCGAGGCCATCGAGGCA
CTGGATGGGTCCTTCACCACCGACACCGTCTTCCGCGTCACCGCCACCGGGGACGGCTTCACCCTGCGG
GAGGTGCCGGTGGACCCGCCCCTGACCAAGGTGTTCCCCGACGACGAATCGGACGACGAATCGGACG
ACGGGGAGGACGGCGACCCGGACTCCCGGACGTTCGTCGCGTACGGGGACGACGGCGACCTGGCGG
GCTTCGTGGTCGTCTCGTACTCCGGCTGGAACCGCCGGCTGACCGTCGAGGACATCGAGGTCGCCCCG
GAGCACCGGGGGCACGGGGTCGGGCGCGCGTTGATGGGGCTCGCGACGGAGTTCGCCCGCGAGCGG
GGCGCCGGGCACCTCTGGCTGGAGGTCACCAACGTCAACGCACCGGCGATCCACGCGTACCGGCGGA
TGGGGTTCACCCTCTGCGGCCTGGACACCGCCCTGTACGACGGCACCGCCTCGGACGGCGAGCAGGCG
CTCTACATGAGCATGCCCTGCCCCTAATCAGTACTGACAATAAAAAGATTCTTGTTTTCAAGAACTTGT
CATTTGTATAGTTTTTTTATATTGTAGTTGTTCTATTTTAATCAAATGTTAGCGTGATTTATATTTTTTTTC
GCCTCGACATCATCTGCCCAGATGCGAAGTTAAGTGCGCAGAAAGTAATATCATGCGTCAATCGTATG
TGAATGCTGGTCGCTATACTGCTGTCGATTCGATACTAACGCCGCcactagttctagagcggccgccaccgcggtg
gagctccagcttttgttccctttagtgagggttaattgcgcgcttggcgtaatcatggtcatagctgtttcctgtgtgaaattgttatccgct
cacaattccacacaacatacgagccggaagcataaagtgtaaagcctggggtgcctaatgagtgagctaactcacattaattgcgttg
cgctcactgcccgctttccagtcgggaaacctgtcgtgccagctgcattaatgaatcggccaacgcgcggggagaggcggtttgcgtatt
gggcgctcttccgcttcctcgctcactgactcgctgcgctcggtcgttcggctgcggcgagcggtatcagctcactcaaaggcggtaatac
ggttatccacagaatcaggggataacgcaggaaagaacatgtgagcaaaaggccagcaaaaggccaggaaccgtaaaaaggccgc
gttgctggcgtttttccataggctccgcccccctgacgagcatcacaaaaatcgacgctcaagtcagaggtggcgaaacccgacagga**

ctataaagataccaggcgtttccccctggaagctccctcgtgcgctctcctgttccgaccctgccgcttaccggatacctgtccgcctttct
cccttcgggaagcgtggcgctttctcatagctcacgctgtaggtatctcagttcggtgtaggtcgttcgctccaagctgggctgtgtgcac
gaaccccccgttcagcccgaccgctgcgccttatccggtaactatcgtcttgagtccaacccggtaagacacgacttatcgccactggca
gcagccactggtaacaggattagcagagcgaggtatgtaggcggtgctacagagttcttgaagtggtggcctaactacggctacacta
gaaggacagtatttggtatctgcgctctgctgaagccagttaccttcggaaaaagagttggtagctcttgatccggcaaacaaaccacc
gctggtagcggtggtttttttgtttgcaagcagcagattacgcgcagaaaaaaaggatctcaagaagatcctttgatcttttctacgggg
tctgacgctcagtggaacgaaaactcacgttaagggattttggtcatgagattatcaaaaaggatcttcacctagatccttttaaattaa
aaatgaagttttaaatcaatctaaagtatatatgagtaaacttggtctgacagttaccaatgcttaatcagtgaggcacctatctcagc
gatctgtctatttcgttcatccatagttgcctgactccccgtcgtgtagataactacgatacgggagggcttaccatctggccccagtgct
gcaatgataccgcgagacccacgctcaccggctccagatttatcagcaataaaccagccagccggaagggccgagcgcagaagtggt
cctgcaactttatccgcctccatccagtctattaattgttgccgggaagctagagtaagtagttcgccagttaatagtttgcgcaacgttg
ttgccattgctacaggcatcgtggtgtcacgctcgtcgtttggtatggcttcattcagctccggttcccaacgatcaaggcgagttacatg
atcccccatgttgtgcaaaaaagcggttagctccttcggtcctccgatcgttgtcagaagtaagttggccgcagtgttatcactcatggtt
atggcagcactgcataattctcttactgtcatgccatccgtaagatgcttttctgtgactggtgagtactcaaccaagtcattctgagaat
agtgtatgcggcgaccgagttgctcttgcccggcgtcaatacgggataataccgcgccacatagcagaactttaaaagtgctcatcatt
ggaaaacgttcttcggggcgaaaactctcaaggatcttaccgctgttgagatccagttcgatgtaacccactcgtgcacccaactgatct
tcagcatcttttactttcaccagcgtttctgggtgagcaaaaacaggaaggcaaaatgccgcaaaaaagggaataagggcgacacgg
aaatgttgaatactcatactcttcctttttcaatattattgaagcatttatcagggttattgtctcatgagcggatacatatttgaatgtatt
tagaaaaataaacaaataggggttccgcgcacatttccccgaaaagtgccac

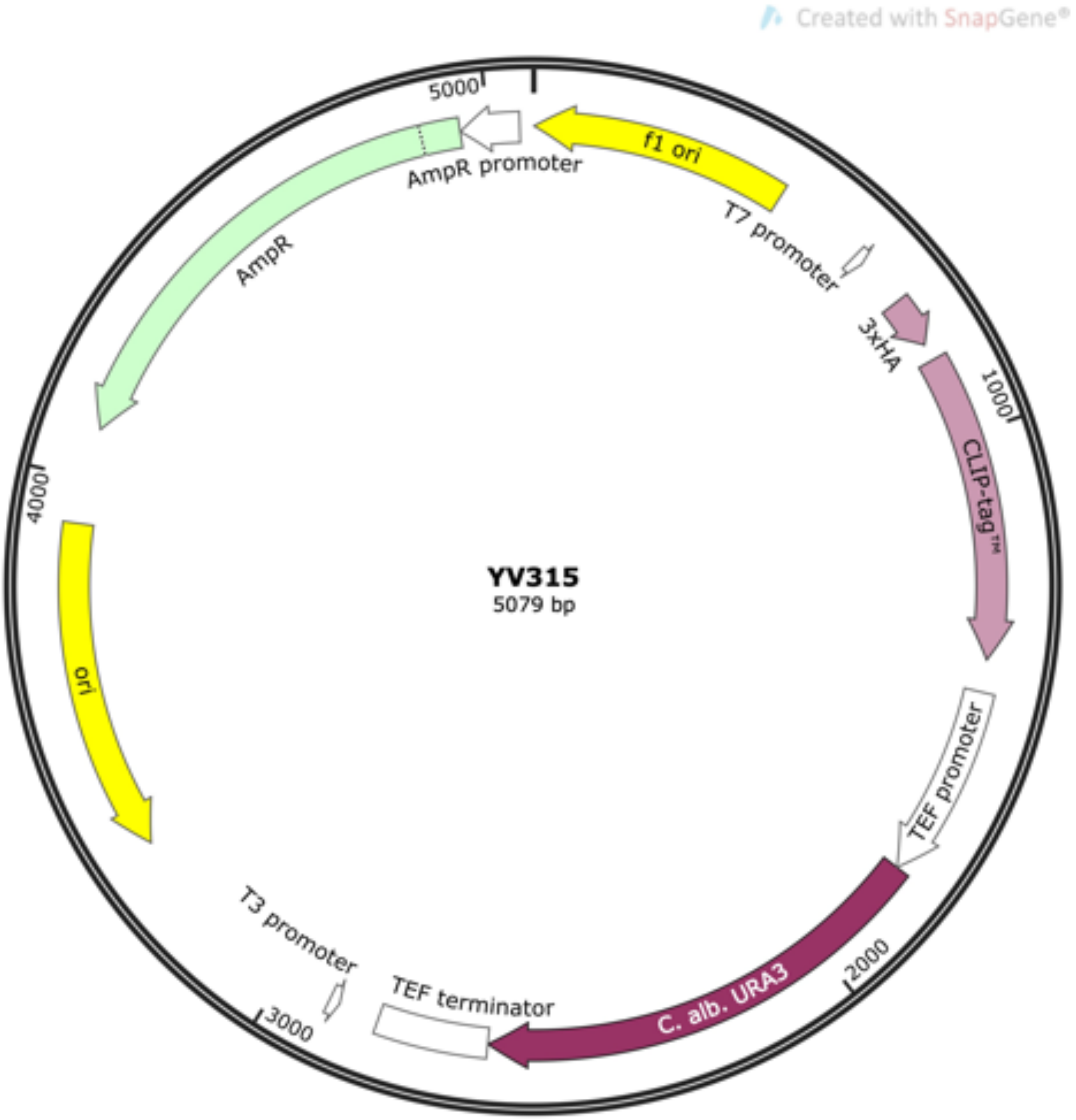
Created with SnapGene®
YV315
5079 bp
f1 ori
T7 promoter
3xHA
CLIP-tag™
TEF promoter
C. alb. URA3
TEF terminator
T3 promoter
ori
AmpR
AmpR promoter
1000
2000
3000
4000
5000

**YV315: pBS-SKII-3XHA-fCLIP-URA**

**ctaaattgtaagcgttaatattttgttaaaattcgcgttaaatttttgttaaatcagctcattttttaaccaataggccgaaatcggcaaa
atcccttataaatcaaaagaatagaccgagatagggttgagtgttgttccagtttggaacaagagtccactattaaagaacgtggact
ccaacgtcaaagggcgaaaaaccgtctatcagggcgatggcccactacgtgaaccatcaccctaatcaagttttttggggtcgaggtg
ccgtaaagcactaaatcggaaccctaaagggagcccccgatttagagcttgacggggaaagccggcgaacgtggcgagaaaggaag
ggaagaaagcgaaaggagcgggcgctagggcgctggcaagtgtagcggtcacgctgcgcgtaaccaccacacccgccgcgcttaatg
cgccgctacagggcgcgtcccattcgccattcaggctgcgcaactgttgggaagggcgatcggtgcgggcctcttcgctattacgccagc
tggcgaaagggggatgtgctgcaaggcgattaagttgggtaacgccagggttttcccagtcacgacgttgtaaaacgacggccagtga
gcgcgcgtaatacgactcactatagggcgaattgggtaccgggccccccctcgaggtcgacggtatcgataagcttgatatcgggtcga
cggatccccgggttaattaacatctttTACCCATACGATGTTCCTGACTATGCGGGCTATCCCTATGACGTCCCGG
ACTATGCAGGATCCTATCCATATGACGTTCCAGATTACGCTGCTCAGTGCggttcaggtggatctggttctATG
GACAAAGACTGCGAAATGAAGCGCACCACCCTGGATAGCCCTCTGGGCAAGCTGGAACTGTCTGGGT
GCGAACAGGGCCTGCACCGTATCATCTTCCTGGGCAAAGGAACATCTGCCGCCGACGCCGTGGAAGT
GCCTGCCCCAGCCGCCGTGCTGGGCGGACCAGAGCCACTGATCCAGGCCACCGCCTGGCTCAACGCCT
ACTTTCACCAGCCTGAGGCCATCGAGGAGTTCCCTGTGCCAGCCCTGCACCACCCAGTGTTCCAGCAGG
AGAGCTTTACCCGCCAGGTGCTGTGGAAACTGCTGAAAGTGGTGAAGTTCGGAGAGGTCATCAGCGA
GAGCCACCTGGCCGCCCTGGTGGGCAATCCCGCCGCCACCGCCGCCGTGAACACCGCCCTGGACGGAA
ATCCCGTGCCCATTCTGATCCCCTGCCACCGGGTGGTGCAGGGCGACAGCGACGTGGGGCCCTACCTG
GGCGGGCTCGCCGTGAAAGAGTGGCTGCTGGCCCACGAGGGCCACAGACTGGGCAAGCCTGGGCTG
GGTtaaTATATAACTGTCTAGAAATAAAGAGTATCATCTTTCAAAGGTCACCCGGCCAGCGACATGGA
GGCCCAGAATACCCTCCTTGACAGTCTTGACGTGCGCAGCTCAGGGGCATGATGTGACTGTCGCCCGT
ACATTTAGCCCATACATCCCCATGTATAATCATTTGCATCCATACATTTTGATGGCCGCACGGCGCGAA
GCAAAAATTACGGCTCCTCGCTGTAGACCTGCGAGCAGGGAAACGCTCCCCTCACAGACGCGTTGAAT
TGTCCCCACGCCGCGCCCCTGTAGAGAAATATAAAAGGTTAGGATTTGCCACTGAGGTTCTTCTTTCAT
ATACTTCCTTTTAAAATCTTGCTAGGATACAGTTCTCACATCACATCCGAACATAAACAACCATGACAG
TCAACACTAAGACCTATAGTGAGAGAGCAGAAACTCATGCCTCACCAGTAGCACAACGATTATTTCGA
TTAATGGAACTGAAGAAAACCAATTTATGTGCATCAATTGATGTTGATACCACTAAGGAATTCCTTGA
ATTAATTGATAAATTGGGTCCTTATGTATGCTTAATCAAGACTCATATTGATATAATCAATGATTTTTCC
TATGAATCCACTATTGAACCATTATTAGAACTTTCACGTAAACATCAATTTATGATTTTTGAAGATAGA
AAATTTGCTGATATTGGTAATACCGTGAAGAAACAATATATTGGTGGAGTTTATAAAATTAGTAGTTG
GGCAGATATTACTAATGCTCATGGTGTCACTGGGAATGGAGTAGTTGAAGGATTAAAACAGGGAGCT
AAAGAAACCACCACCAACCAAGAGCCAAGAGGGTTATTGATGTTAGCTGAATTATCATCAGTGGGAT
CATTAGCATATGGAGAATATTCTCAAAAAACTGTTGAAATTGCTAAATCCGATAAGGAATTTGTTATT
GGATTTATTGCCCAACGTGATATGGGTGGACAAGAAGAAGGATTTGATTGGCTTATTATGACACCTGG
AGTTGGATTAGATGATAAAGGTGATGGATTAGGACAACAATATAGAACTGTTGATGAAGTTGTTAGC
ACTGGAACTGATATTATCATTGTTGGTAGAGGATTGTTTGGTAAAGGAAGAGATCCAGATATTGAAG
GTAAAAGGTATAGAGATGCTGGTTGGAATGCTTATTTGAAAAAGACTGGCCAATTATAATCAGTACTG
ACAATAAAAAGATTCTTGTTTTCAAGAACTTGTCATTTGTATAGTTTTTTTATATTGTAGTTGTTCTATTT
TAATCAAATGTTAGCGTGATTTATATTTTTTTTCGCCTCGACATCATCTGCCCAGATGCGAAGTTAAGTG
CGCAGAAAGTAATATCATGCGTCAATCGTATGTGAATGCTGGTCGCTATACTGCTGTCGATTCGATACT
AACGCCGCcactagttctagagcggccgccaccgcggtggagctccagcttttgttccctttagtgagggttaattgcgcgcttggcg
taatcatggtcatagctgtttcctgtgtgaaattgttatccgctcacaattccacacaacatacgagccggaagcataaagtgtaaagcc
tggggtgcctaatgagtgagctaactcacattaattgcgttgcgctcactgcccgctttccagtcgggaaacctgtcgtgccagctgcatt**

aatgaatcggccaacgcgcggggagaggcggtttgcgtattgggcgctcttccgcttcctcgctcactgactcgctgcgctcggtcgttcg
gctgcggcgagcggtatcagctcactcaaaggcggtaatacggttatccacagaatcaggggataacgcaggaaagaacatgtgagc
aaaaggccagcaaaaggccaggaaccgtaaaaaggccgcgttgctggcgtttttccataggctccgcccccctgacgagcatcacaa
aaatcgacgctcaagtcagaggtggcgaaacccgacaggactataaagataccaggcgtttccccctggaagctccctcgtgcgctct
cctgttccgaccctgccgcttaccggatacctgtccgcctttctcccttcgggaagcgtggcgctttctcatagctcacgctgtaggtatctc
agttcggtgtaggtcgttcgctccaagctgggctgtgtgcacgaaccccccgttcagcccgaccgctgcgccttatccggtaactatcgtc
ttgagtccaacccggtaagacacgacttatcgccactggcagcagccactggtaacaggattagcagagcgaggtatgtaggcggtgc
tacagagttcttgaagtggtggcctaactacggctacactagaaggacagtatttggtatctgcgctctgctgaagccagttaccttcgg
aaaaagagttggtagctcttgatccggcaaacaaaccaccgctggtagcggtggtttttttgtttgcaagcagcagattacgcgcagaa
aaaaaggatctcaagaagatcctttgatcttttctacggggtctgacgctcagtggaacgaaaactcacgttaagggattttggtcatg
agattatcaaaaaggatcttcacctagatccttttaaattaaaaatgaagttttaaatcaatctaaagtatatatgagtaaacttggtct
gacagttaccaatgcttaatcagtgaggcacctatctcagcgatctgtctatttcgttcatccatagttgcctgactccccgtcgtgtagat
aactacgatacgggagggcttaccatctggccccagtgctgcaatgataccgcgagacccacgctcaccggctccagatttatcagcaa
taaaccagccagccggaagggccgagcgcagaagtggtcctgcaactttatccgcctccatccagtctattaattgttgccgggaagct
agagtaagtagttcgccagttaatagtttgcgcaacgttgttgccattgctacaggcatcgtggtgtcacgctcgtcgtttggtatggctt
cattcagctccggttcccaacgatcaaggcgagttacatgatcccccatgttgtgcaaaaaagcggttagctccttcggtcctccgatcg
ttgtcagaagtaagttggccgcagtgttatcactcatggttatggcagcactgcataattctcttactgtcatgccatccgtaagatgcttt
tctgtgactggtgagtactcaaccaagtcattctgagaatagtgtatgcggcgaccgagttgctcttgcccggcgtcaatacgggataat
accgcgccacatagcagaactttaaaagtgctcatcattggaaaacgttcttcggggcgaaaactctcaaggatcttaccgctgttgag
atccagttcgatgtaacccactcgtgcacccaactgatcttcagcatcttttactttcaccagcgtttctgggtgagcaaaaacaggaag
gcaaaatgccgcaaaaaagggaataagggcgacacggaaatgttgaatactcatactcttcctttttcaatattattgaagcatttatc
agggttattgtctcatgagcggatacatatttgaatgtatttagaaaaataaacaaataggggttccgcgcacatttccccgaaaagtg
ccac

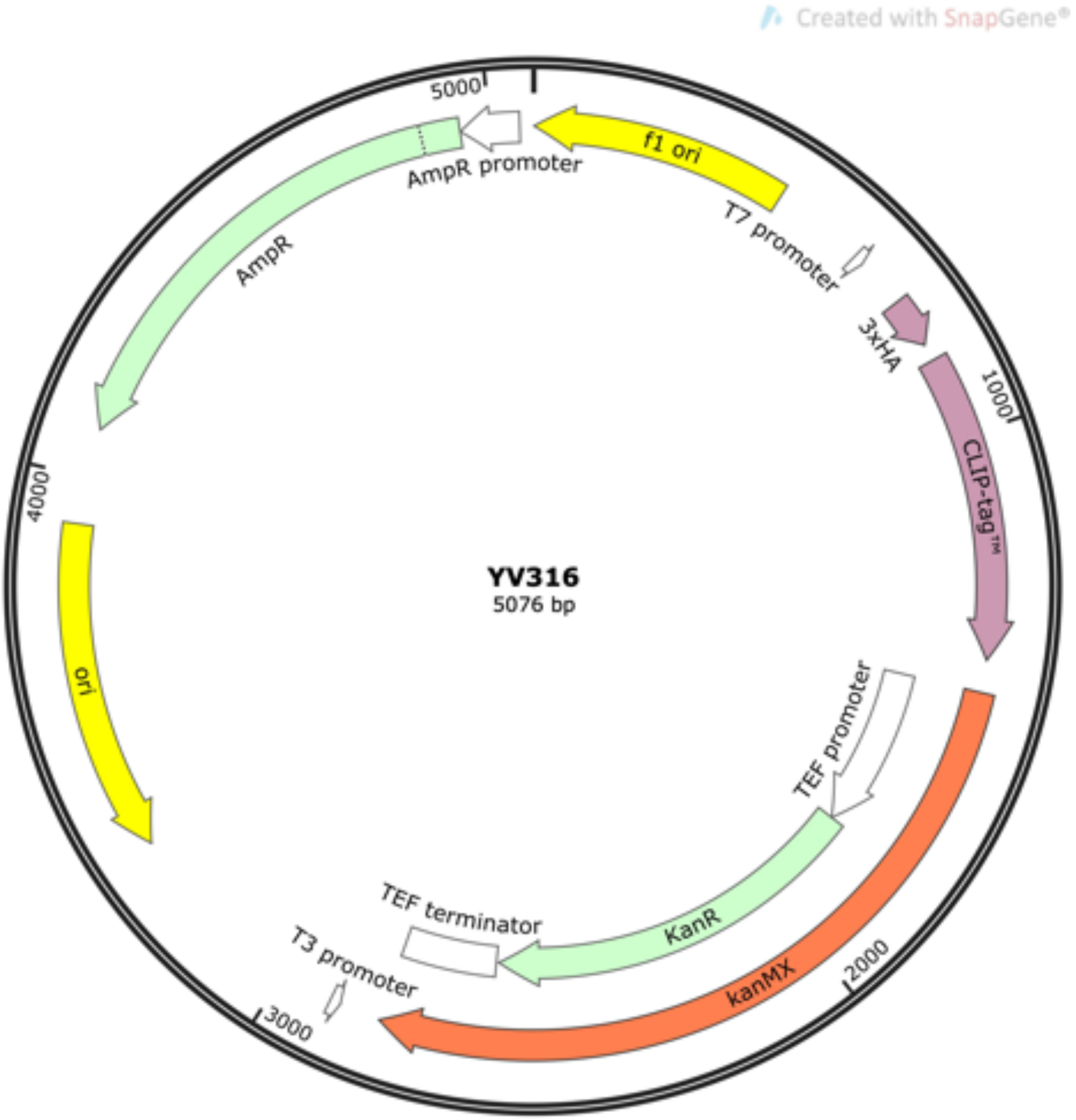
Created with SnapGene®
YV316
5076 bp
f1 ori
T7 promoter
3xHA
CLIP-tag™
1000
TEF promoter
kanMX
KanR
2000
TEF terminator
T3 promoter
3000
ori
4000
AmpR
AmpR promoter
5000

**YV316: pBS-SKII-3XHA-fCLIP-Kan**

**ctaaattgtaagcgttaatattttgttaaaattcgcgttaaatttttgttaaatcagctcattttttaaccaataggccgaaatcggcaaa
atcccttataaatcaaaagaatagaccgagatagggttgagtgttgttccagtttggaacaagagtccactattaaagaacgtggact
ccaacgtcaaagggcgaaaaaccgtctatcagggcgatggcccactacgtgaaccatcaccctaatcaagttttttggggtcgaggtg
ccgtaaagcactaaatcggaaccctaaagggagcccccgatttagagcttgacggggaaagccggcgaacgtggcgagaaaggaag
ggaagaaagcgaaaggagcgggcgctagggcgctggcaagtgtagcggtcacgctgcgcgtaaccaccacacccgccgcgcttaatg
cgccgctacagggcgcgtcccattcgccattcaggctgcgcaactgttgggaagggcgatcggtgcgggcctcttcgctattacgccagc
tggcgaaagggggatgtgctgcaaggcgattaagttgggtaacgccagggttttcccagtcacgacgttgtaaaacgacggccagtga
gcgcgcgtaatacgactcactatagggcgaattgggtaccgggccccccctcgaggtcgacggtatcgataagcttgatatcgggtcga
cggatccccgggttaattaacatctttTACCCATACGATGTTCCTGACTATGCGGGCTATCCCTATGACGTCCCGG
ACTATGCAGGATCCTATCCATATGACGTTCCAGATTACGCTGCTCAGTGCggttcaggtggatctggttctATG
GACAAAGACTGCGAAATGAAGCGCACCACCCTGGATAGCCCTCTGGGCAAGCTGGAACTGTCTGGGT
GCGAACAGGGCCTGCACCGTATCATCTTCCTGGGCAAAGGAACATCTGCCGCCGACGCCGTGGAAGT
GCCTGCCCCAGCCGCCGTGCTGGGCGGACCAGAGCCACTGATCCAGGCCACCGCCTGGCTCAACGCCT
ACTTTCACCAGCCTGAGGCCATCGAGGAGTTCCCTGTGCCAGCCCTGCACCACCCAGTGTTCCAGCAGG
AGAGCTTTACCCGCCAGGTGCTGTGGAAACTGCTGAAAGTGGTGAAGTTCGGAGAGGTCATCAGCGA
GAGCCACCTGGCCGCCCTGGTGGGCAATCCCGCCGCCACCGCCGCCGTGAACACCGCCCTGGACGGAA
ATCCCGTGCCCATTCTGATCCCCTGCCACCGGGTGGTGCAGGGCGACAGCGACGTGGGGCCCTACCTG
GGCGGGCTCGCCGTGAAAGAGTGGCTGCTGGCCCACGAGGGCCACAGACTGGGCAAGCCTGGGCTG
GGTtaaTATATAACTGTCTAGAAATAAAGAGTATCATCTTTCAAAGGTCACCCGGCCAGCGACATGGA
GGCCCAGAATACCCTCCTTGACAGTCTTGACGTGCGCAGCTCAGGGGCATGATGTGACTGTCGCCCGT
ACATTTAGCCCATACATCCCCATGTATAATCATTTGCATCCATACATTTTGATGGCCGCACGGCGCGAA
GCAAAAATTACGGCTCCTCGCTGCAGACCTGCGAGCAGGGAAACGCTCCCCTCACAGACGCGTTGAAT
TGTCCCCACGCCGCGCCCCTGTAGAGAAATATAAAAGGTTAGGATTTGCCACTGAGGTTCTTCTTTCAT
ATACTTCCTTTTAAAATCTTGCTAGGATACAGTTCTCACATCACATCCGAACATAAACAACCATGGGTA
AGGAAAAGACTCACGTTTCGAGGCCGCGATTAAATTCCAACATGGATGCTGATTTATATGGGTATAAA
TGGGCTCGCGATAATGTCGGGCAATCAGGTGCGACAATCTATCGATTGTATGGGAAGCCCGATGCGC
CAGAGTTGTTTCTGAAACATGGCAAAGGTAGCGTTGCCAATGATGTTACAGATGAGATGGTCAGACT
AAACTGGCTGACGGAATTTATGCCTCTTCCGACCATCAAGCATTTTATCCGTACTCCTGATGATGCATG
GTTACTCACCACTGCGATCCCCGGCAAAACAGCATTCCAGGTATTAGAAGAATATCCTGATTCAGGTG
AAAATATTGTTGATGCGCTGGCAGTGTTCCTGCGCCGGTTGCATTCGATTCCTGTTTGTAATTGTCCTTT
TAACAGCGATCGCGTATTTCGTCTCGCTCAGGCGCAATCACGAATGAATAACGGTTTGGTTGATGCGA
GTGATTTTGATGACGAGCGTAATGGCTGGCCTGTTGAACAAGTCTGGAAAGAAATGCATAAGCTTTTG
CCATTCTCACCGGATTCAGTCGTCACTCATGGTGATTTCTCACTTGATAACCTTATTTTTGACGAGGGGA
AATTAATAGGTTGTATTGATGTTGGACGAGTCGGAATCGCAGACCGATACCAGGATCTTGCCATCCTA
TGGAACTGCCTCGGTGAGTTTTCTCCTTCATTACAGAAACGGCTTTTTCAAAAATATGGTATTGATAAT
CCTGATATGAATAAATTGCAGTTTCATTTGATGCTCGATGAGTTTTTCTAATCAGTACTGACAATAAAA
AGATTCTTGTTTTCAAGAACTTGTCATTTGTATAGTTTTTTTATATTGTAGTTGTTCTATTTTAATCAAAT
GTTAGCGTGATTTATATTTTTTTTCGCCTCGACATCATCTGCCCAGATGCGAAGTTAAGTGCGCAGAAA
GTAATATCATGCGTCAATCGTATGTGAATGCTGGTCGCTATACTGCTGTCGATTCGATACTAACGCCGC
cactagttctagagcggccgccaccgcggtggagctccagcttttgttccctttagtgagggttaattgcgcgcttggcgtaatcatggtc
atagctgtttcctgtgtgaaattgttatccgctcacaattccacacaacatacgagccggaagcataaagtgtaaagcctggggtgcct
aatgagtgagctaactcacattaattgcgttgcgctcactgcccgctttccagtcgggaaacctgtcgtgccagctgcattaatgaatcg**

**gccaacgcgcggggagaggcggtttgcgtattgggcgctcttccgcttcctcgctcactgactcgctgcgctcggtcgttcggctgcggcg**
**agcggtatcagctcactcaaaggcggtaatacggttatccacagaatcaggggataacgcaggaaagaacatgtgagcaaaaggcc**
**agcaaaaggccaggaaccgtaaaaaggccgcgttgctggcgtttttccataggctccgcccccctgacgagcatcacaaaaatcgacg**
**ctcaagtcagaggtggcgaaacccgacaggactataaagataccaggcgtttccccctggaagctccctcgtgcgctctcctgttccga**
**ccctgccgcttaccggatacctgtccgcctttctcccttcgggaagcgtggcgctttctcatagctcacgctgtaggtatctcagttcggtg**
**taggtcgttcgctccaagctgggctgtgtgcacgaaccccccgttcagcccgaccgctgcgccttatccggtaactatcgtcttgagtcca**
**acccggtaagacacgacttatcgccactggcagcagccactggtaacaggattagcagagcgaggtatgtaggcggtgctacagagtt**
**cttgaagtggtggcctaactacggctacactagaaggacagtatttggtatctgcgctctgctgaagccagttaccttcggaaaaagag**
**ttggtagctcttgatccggcaaacaaaccaccgctggtagcggtggtttttttgtttgcaagcagcagattacgcgcagaaaaaaagga**
**tctcaagaagatcctttgatcttttctacggggtctgacgctcagtggaacgaaaactcacgttaagggattttggtcatgagattatca**
**aaaaggatcttcacctagatccttttaaattaaaaatgaagttttaaatcaatctaaagtatatatgagtaaacttggtctgacagttac**
**caatgcttaatcagtgaggcacctatctcagcgatctgtctatttcgttcatccatagttgcctgactccccgtcgtgtagataactacgat**
**acgggagggcttaccatctggccccagtgctgcaatgataccgcgagacccacgctcaccggctccagatttatcagcaataaaccag**
**ccagccggaagggccgagcgcagaagtggtcctgcaactttatccgcctccatccagtctattaattgttgccgggaagctagagtaag**
**tagttcgccagttaatagtttgcgcaacgttgttgccattgctacaggcatcgtggtgtcacgctcgtcgtttggtatggcttcattcagct**
**ccggttcccaacgatcaaggcgagttacatgatcccccatgttgtgcaaaaaagcggttagctccttcggtcctccgatcgttgtcagaa**
**gtaagttggccgcagtgttatcactcatggttatggcagcactgcataattctcttactgtcatgccatccgtaagatgcttttctgtgact**
**ggtgagtactcaaccaagtcattctgagaatagtgtatgcggcgaccgagttgctcttgcccggcgtcaatacgggataataccgcgcc**
**acatagcagaactttaaaagtgctcatcattggaaaacgttcttcggggcgaaaactctcaaggatcttaccgctgttgagatccagtt**
**cgatgtaacccactcgtgcacccaactgatcttcagcatcttttactttcaccagcgtttctgggtgagcaaaaacaggaaggcaaaat**
**gccgcaaaaaagggaataagggcgacacggaaatgttgaatactcatactcttcctttttcaatattattgaagcatttatcagggttat**
**tgtctcatgagcggatacatatttgaatgtatttagaaaaataaacaaataggggttccgcgcacatttccccgaaaagtgccac**

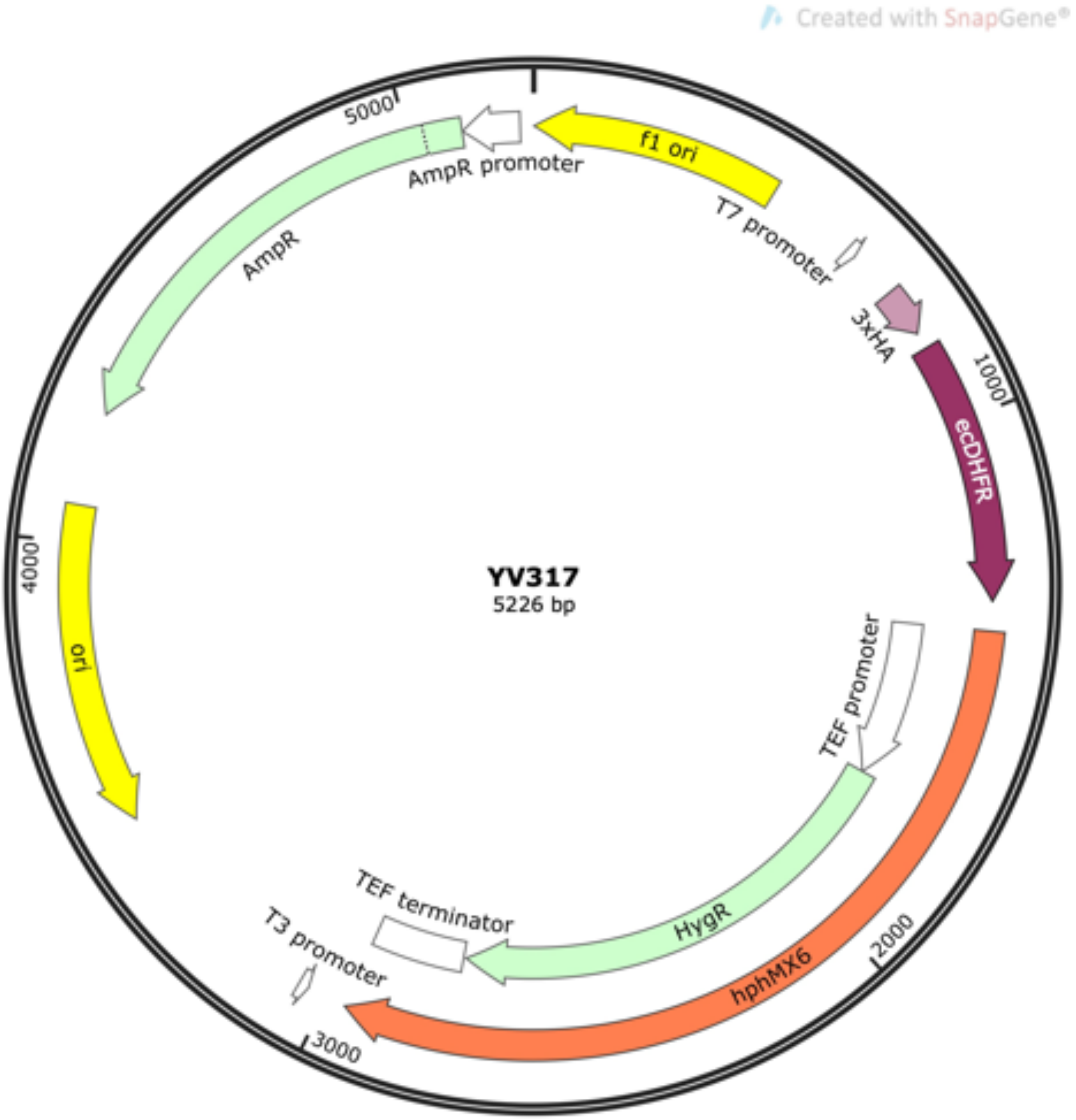
Created with SnapGene®
YV317
5226 bp
f1 ori
AmpR promoter
AmpR
T7 promoter
3xHA
ecDHFR
TEF promoter
HygR
hphMX6
TEF terminator
T3 promoter
ori
1000
2000
3000
4000
5000

**YV317: pBS-SKII-3XHA-eDHFR-Hygromycin**

**ctaaattgtaagcgttaatattttgttaaaattcgcgttaaatttttgttaaatcagctcattttttaaccaataggccgaaatcggcaaa
atcccttataaatcaaaagaatagaccgagatagggttgagtgttgttccagtttggaacaagagtccactattaaagaacgtggact
ccaacgtcaaagggcgaaaaaccgtctatcagggcgatggcccactacgtgaaccatcaccctaatcaagttttttggggtcgaggtg
ccgtaaagcactaaatcggaaccctaaagggagcccccgatttagagcttgacggggaaagccggcgaacgtggcgagaaaggaag
ggaagaaagcgaaaggagcgggcgctagggcgctggcaagtgtagcggtcacgctgcgcgtaaccaccacacccgccgcgcttaatg
cgccgctacagggcgcgtcccattcgccattcaggctgcgcaactgttgggaagggcgatcggtgcgggcctcttcgctattacgccagc
tggcgaaagggggatgtgctgcaaggcgattaagttgggtaacgccagggttttcccagtcacgacgttgtaaaacgacggccagtga
gcgcgcgtaatacgactcactatagggcgaattgggtaccgggccccccctcgaggtcgacggtatcgataagcttgatatcgggtcga
cggatccccgggttaattaacatctttTACCCATACGATGTTCCTGACTATGCGGGCTATCCCTATGACGTCCCGG
ACTATGCAGGATCCTATCCATATGACGTTCCAGATTACGCTGCTCAGTGCggttcaggtggatctggttctATG
ATCAGTCTGATTGCGGCGTTAGCGGTAGATCGCGTTATCGGCATGGAAAACGCCATGCCGTGGAACCT
GCCTGCCGATCTCGCCTGGTTTAAACGCAACACCTTAAATAAACCCGTGATTATGGGCCGCCATACCTG
GGAATCAATCGGTCGTCCGTTGCCAGGACGCAAAAATATTATCCTCAGCAGTCAACCGGGTACGGACG
ATCGCGTAACGTGGGTGAAGTCGGTGGATGAAGCCATCGCGGCGTGTGGTGACGTACCAGAAATCAT
GGTGATTGGCGGCGGTCGCGTTTATGAACAGTTCTTGCCAAAAGCGCAAAAACTGTATCTGACGCATA
TCGACGCAGAAGTGGAAGGCGACACCCATTTCCCGGATTACGAGCCGGATGACTGGGAATCGGTATT
CAGCGAATTCCACGATGCTGATGCGCAGAACTCTCACAGCTATTGCTTTGAGATTCTGGAGCGGCGGT
AATATATAACTGTCTAGAAATAAAGAGTATCATCTTTCAAAGGTCACCCGGCCAGCGACATGGAGGCC
CAGAATACCCTCCTTGACAGTCTTGACGTGCGCAGCTCAGGGGCATGATGTGACTGTCGCCCGTACATT
TAGCCCATACATCCCCATGTATAATCATTTGCATCCATACATTTTGATGGCCGCACGGCGCGAAGCATA
AATTACGGCTCCTCGCTGCgGACCTGCGAGCAGGGAAACGCTCCCCTCACAGACGCGTTGAATTGTCCC
CACGCCGCGCCCCTGTAGAGAAATATAAAAGGTTAGGATTTGCCACTGAGGTTCTTCTTTCATATACTT
CCTTTTAAAATCTTGCTAGGATACAGTTCTCACATCACATCCGAACATAAACAACCATGGGTAAAAAGC
CTGAACTCACCGCGACGTCTGTCGAGAAGTTTCTGATCGAAAAGTTCGACAGCGTCTCCGACCTGATG
CAGCTCTCGGAGGGCGAAGAATCTCGTGCTTTCAGCTTCGATGTAGGAGGGCGTGGATATGTCCTGCG
GGTAAATAGCTGCGCCGATGGTTTCTACAAAGATCGTTATGTTTATCGGCACTTTGCATCGGCCGCGCT
CCCGATTCCGGAAGTGCTTGACATTGGGGAATTCAGCGAGAGCCTGACCTATTGCATCTCCCGCCGTG
CACAGGGTGTCACGTTGCAAGACCTGCCTGAAACCGAACTGCCCGCTGTTCTGCAGCCGGTCGCGGAG
GCCATGGATGCGATCGCTGCGGCCGATCTTAGCCAGACGAGCGGGTTCGGCCCATTCGGACCGCAAG
GAATCGGTCAATACACTACATGGCGTGATTTCATATGCGCGATTGCTGATCCCCATGTGTATCACTGGC
AAACTGTGATGGACGACACCGTCAGTGCGTCCGTCGCGCAGGCTCTCGATGAGCTGATGCTTTGGGCC
GAGGACTGCCCCGAAGTCCGGCACCTCGTGCACGCGGATTTCGGCTCCAACAATGTCCTGACGGACAA
TGGCCGCATAACAGCGGTCATTGACTGGAGCGAGGCGATGTTCGGGGATTCCCAATACGAGGTCGCC
AACATCTTCTTCTGGAGGCCGTGGTTGGCTTGTATGGAGCAGCAGACGCGCTACTTCGAGCGGAGGCA
TCCGGAGCTTGCAGGATCGCCGCGGCTCCGGGCGTATATGCTCCGCATTGGTCTTGACCAACTCTATCA
GAGCTTGGTTGACGGCAATTTCGATGATGCAGCTTGGGCGCAGGGTCGATGCGACGCAATCGTCCGA
TCCGGAGCCGGGACTGTCGGGCGTACACAAATCGCCCGCAGAAGCGCGGCCGTCTGGACCGATGGCT
GTGTAGAAGTACTCGCCGATAGTGGAAACCGACGCCCCAGCACTCGTCCGAGGGCAAAGGAATAATC
AGTACTGACAATAAAAAGATTCTTGTTTTCAAGAACTTGTCATTTGTATAGTTTTTTTATATTGTAGTTG
TTCTATTTTAATCAAATGTTAGCGTGATTTATATTTTTTTTCGCCTCGACATCATCTGCCCAGATGCGAA
GTTAAGTGCGCAGAAAGTAATATCATGCGTCAATCGTATGTGAATGCTGGTCGCTATACTGCTGTCGA
TTCGATACTAACGCCGCcactagttctagagcggccgccaccgcggtggagctccagcttttgttccctttagtgagggttaattg**

**cgcgcttggcgtaatcatggtcatagctgtttcctgtgtgaaattgttatccgctcacaattccacacaacatacgagccggaagcataa
agtgtaaagcctggggtgcctaatgagtgagctaactcacattaattgcgttgcgctcactgcccgctttccagtcgggaaacctgtcgt
gccagctgcattaatgaatcggccaacgcgcggggagaggcggtttgcgtattgggcgctcttccgcttcctcgctcactgactcgctgc
gctcggtcgttcggctgcggcgagcggtatcagctcactcaaaggcggtaatacggttatccacagaatcaggggataacgcaggaaa
gaacatgtgagcaaaaggccagcaaaaggccaggaaccgtaaaaaggccgcgttgctggcgtttttccataggctccgcccccctgac
gagcatcacaaaaatcgacgctcaagtcagaggtggcgaaacccgacaggactataaagataccaggcgtttccccctggaagctcc
ctcgtgcgctctcctgttccgaccctgccgcttaccggatacctgtccgcctttctcccttcgggaagcgtggcgctttctcatagctcacgc
tgtaggtatctcagttcggtgtaggtcgttcgctccaagctgggctgtgtgcacgaaccccccgttcagcccgaccgctgcgccttatccg
gtaactatcgtcttgagtccaacccggtaagacacgacttatcgccactggcagcagccactggtaacaggattagcagagcgaggta
tgtaggcggtgctacagagttcttgaagtggtggcctaactacggctacactagaaggacagtatttggtatctgcgctctgctgaagcc
agttaccttcggaaaaagagttggtagctcttgatccggcaaacaaaccaccgctggtagcggtggtttttttgtttgcaagcagcagat
tacgcgcagaaaaaaaggatctcaagaagatcctttgatcttttctacggggtctgacgctcagtggaacgaaaactcacgttaaggg
attttggtcatgagattatcaaaaaggatcttcacctagatccttttaaattaaaaatgaagttttaaatcaatctaaagtatatatgag
taaacttggtctgacagttaccaatgcttaatcagtgaggcacctatctcagcgatctgtctatttcgttcatccatagttgcctgactccc
cgtcgtgtagataactacgatacgggagggcttaccatctggccccagtgctgcaatgataccgcgagacccacgctcaccggctccag
atttatcagcaataaaccagccagccggaagggccgagcgcagaagtggtcctgcaactttatccgcctccatccagtctattaattgtt
gccgggaagctagagtaagtagttcgccagttaatagtttgcgcaacgttgttgccattgctacaggcatcgtggtgtcacgctcgtcgt
ttggtatggcttcattcagctccggttcccaacgatcaaggcgagttacatgatcccccatgttgtgcaaaaaagcggttagctccttcg
gtcctccgatcgttgtcagaagtaagttggccgcagtgttatcactcatggttatggcagcactgcataattctcttactgtcatgccatcc
gtaagatgcttttctgtgactggtgagtactcaaccaagtcattctgagaatagtgtatgcggcgaccgagttgctcttgcccggcgtca
atacgggataataccgcgccacatagcagaactttaaaagtgctcatcattggaaaacgttcttcggggcgaaaactctcaaggatctt
accgctgttgagatccagttcgatgtaacccactcgtgcacccaactgatcttcagcatcttttactttcaccagcgtttctgggtgagca
aaaacaggaaggcaaaatgccgcaaaaaagggaataagggcgacacggaaatgttgaatactcatactcttcctttttcaatattatt
gaagcatttatcagggttattgtctcatgagcggatacatatttgaatgtatttagaaaaataaacaaataggggttccgcgcacatttc
cccgaaaagtgccac**

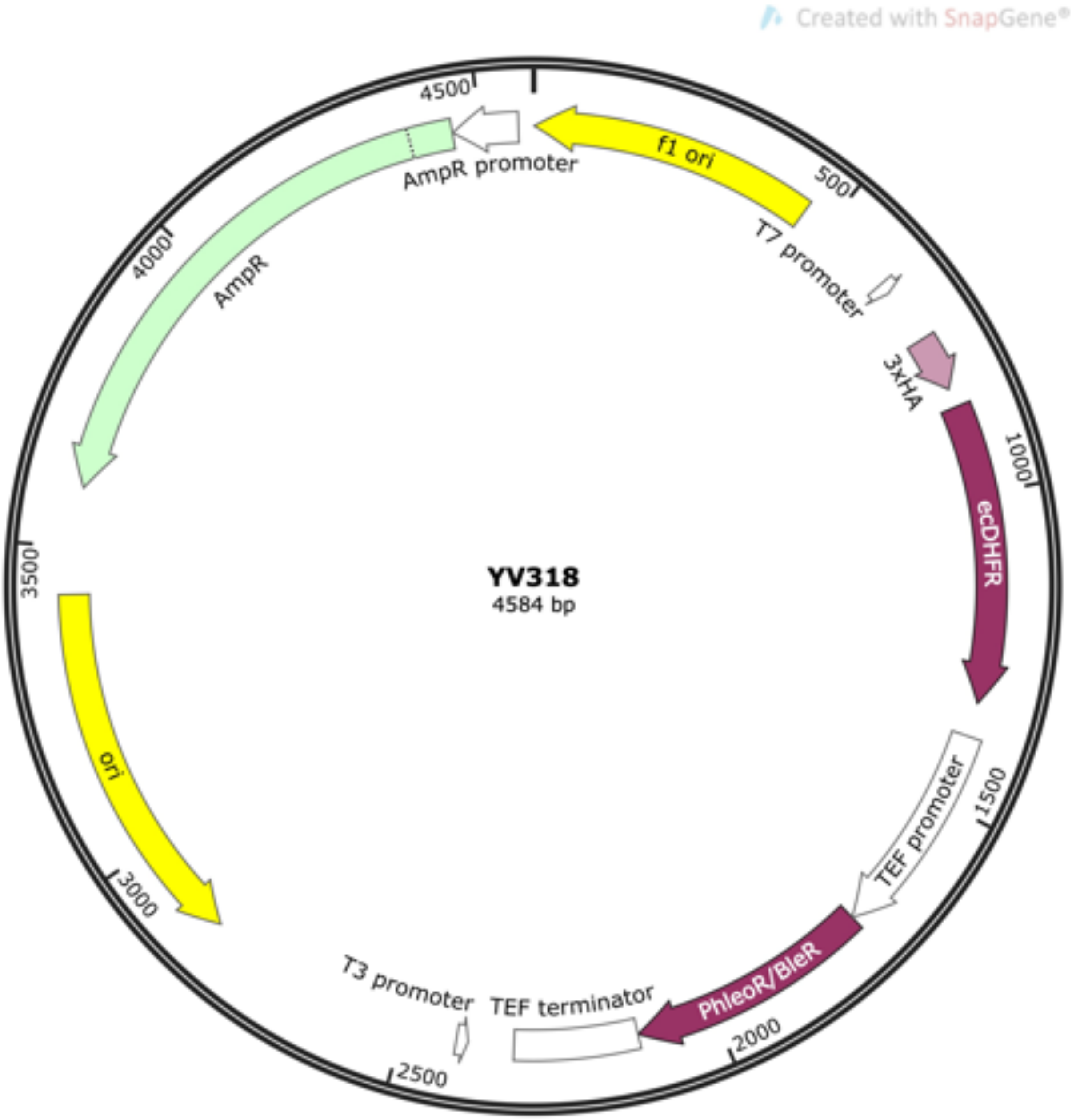
Created with SnapGene®
YV318
4584 bp
f1 ori
500
T7 promoter
3xHA
1000
ecDHFR
1500
TEF promoter
PhleoR/BleR
2000
TEF terminator
T3 promoter
2500
3000
ori
3500
4000
AmpR
AmpR promoter
4500

**YV318: pBS-SKII-3XHA-eDHFR-Phleomycin**

**ctaaattgtaagcgttaatattttgttaaaattcgcgttaaatttttgttaaatcagctcattttttaaccaataggccgaaatcggcaaa
atcccttataaatcaaaagaatagaccgagatagggttgagtgttgttccagtttggaacaagagtccactattaaagaacgtggact
ccaacgtcaaagggcgaaaaaccgtctatcagggcgatggcccactacgtgaaccatcaccctaatcaagttttttggggtcgaggtg
ccgtaaagcactaaatcggaaccctaaagggagcccccgatttagagcttgacggggaaagccggcgaacgtggcgagaaaggaag
ggaagaaagcgaaaggagcgggcgctagggcgctggcaagtgtagcggtcacgctgcgcgtaaccaccacacccgccgcgcttaatg
cgccgctacagggcgcgtcccattcgccattcaggctgcgcaactgttgggaagggcgatcggtgcgggcctcttcgctattacgccagc
tggcgaaagggggatgtgctgcaaggcgattaagttgggtaacgccagggttttcccagtcacgacgttgtaaaacgacggccagtga
gcgcgcgtaatacgactcactatagggcgaattgggtaccgggccccccctcgaggtcgacggtatcgataagcttgatatcgggtcga
cggatccccgggttaattaacatctttTACCCATACGATGTTCCTGACTATGCGGGCTATCCCTATGACGTCCCGG
ACTATGCAGGATCCTATCCATATGACGTTCCAGATTACGCTGCTCAGTGCggttcaggtggatctggttctATG
ATCAGTCTGATTGCGGCGTTAGCGGTAGATCGCGTTATCGGCATGGAAAACGCCATGCCGTGGAACCT
GCCTGCCGATCTCGCCTGGTTTAAACGCAACACCTTAAATAAACCCGTGATTATGGGCCGCCATACCTG
GGAATCAATCGGTCGTCCGTTGCCGGGACGCAAAAATATTATCCTCAGCAGTCAACCGGGTACGGACG
ATCGCGTAACGTGGGTGAAGTCGGTGGATGAAGCCATCGCGGCGTGTGGTGACGTACCAGAAATCAT
GGTGATTGGCGGCGGTCGCGTTTATGAACAGTTCTTGCCAAAAGCGCAAAAACTGTATCTGACGCATA
TCGACGCAGAAGTGGAAGGCGACACCCATTTCCCGGATTACGAGCCGGATGACTGGGAATCGGTATT
CAGCGAATTCCACGATGCTGATGCGCAGAACTCTCACAGCTATTGCTTTGAGATTCTGGAGCGGCGGT
AATATATAACTGTCTAGAAATAAAGAGTATCATCTTTCAAAGGTCACCCGGCCAGCGACATGGAGGCC
CAGAATACCCTCCTTGACAGTCTTGACGTGCGCAGCTCAGGGGCATGATGTGACTGTCGCCCGTACATT
TAGCCCATACATCCCCATGTATAATCATTTGCATCCATACATTTTGATGGCCGCACGGCGCGAAGCAAA
AATTACGGCTCCTCGCTGCAGACCTGCGAGCAGGGAAACGCTCCCCTCACAGACGCGTTGAATTGTCC
CCACGCCGCGCCCCTGTAGAGAAATATAAAAGGTTAGGATTTGCCACTGAGGTTCTTCTTTCATATACT
TCCTTTTAAAATCTTGCTAGGATACAGTTCTCACATCACATCCGAACATAAACAACCATGGGTATGACC
GACCAAGCGACGCCCAACCTGCCATCACGAGATTTCGATcCCACcGCCGCCTTCTATGAAAGGTTGGGC
TTCGGAATCGTTTTCCGGGACGCCGGCTGGATGATCCTCCAGCGCGGGGATCTCAtGCTGGAGTTCTTC
GCCCACCCCGGGCTCGATCCCCTCGCGAGTTGGTTCAGCTGCTGCCTGAGGCTGGACGACCTCGCGGA
GTTCTACCGGCAGTGCAAATCCGTCGGCATCCAGGAAACCAGCAGCGGCTATCCGCGCATCCATGCCC
CCGAACTGCAGGAGTGGGGAGGCACGATGGCCGCTTTGGTCGACCCGGACGGGACGCTCCTGCGCCT
GATACAGAACGAATTGCTTGCAGGCATCTCATGATCAGTACTGACAATAAAAAGATTCTTGTTTTCAA
GAACTTGTCATTTGTATAGTTTTTTTATATTGTAGTTGTTCTATTTTAATCAAATGTTAGCGTGATTTATA
TTTTTTTTCGCCTCGACATCATCTGCCCAGATGCGAAGTTAAGTGCGCAGAAAGTAATATCATGCGTCA
ATCGTATGTGAATGCTGGTCGCTATACTGCTGTCGATTCGATACTAACGCCGCcactagttctagagcggccgc
caccgcggtggagctccagcttttgttccctttagtgagggttaattgcgcgcttggcgtaatcatggtcatagctgtttcctgtgtgaaat
tgttatccgctcacaattccacacaacatacgagccggaagcataaagtgtaaagcctggggtgcctaatgagtgagctaactcacatt
aattgcgttgcgctcactgcccgctttccagtcgggaaacctgtcgtgccagctgcattaatgaatcggccaacgcgcggggagaggcg
gtttgcgtattgggcgctcttccgcttcctcgctcactgactcgctgcgctcggtcgttcggctgcggcgagcggtatcagctcactcaaag
gcggtaatacggttatccacagaatcaggggataacgcaggaaagaacatgtgagcaaaaggccagcaaaaggccaggaaccgta
aaaaggccgcgttgctggcgtttttccataggctccgcccccctgacgagcatcacaaaaatcgacgctcaagtcagaggtggcgaaa
cccgacaggactataaagataccaggcgtttccccctggaagctccctcgtgcgctctcctgttccgaccctgccgcttaccggatacctg
tccgcctttctcccttcgggaagcgtggcgctttctcatagctcacgctgtaggtatctcagttcggtgtaggtcgttcgctccaagctggg
ctgtgtgcacgaaccccccgttcagcccgaccgctgcgccttatccggtaactatcgtcttgagtccaacccggtaagacacgacttatc
gccactggcagcagccactggtaacaggattagcagagcaggtatgtaggcggtgctacagagttcttgaagtggtggcctaactac**

**ggctacactagaaggacagtatttggtatctgcgctctgctgaagccagttaccttcggaaaaagagttggtagctcttgatccggcaa
acaaaccaccgctggtagcggtggtttttttgtttgcaagcagcagattacgcgcagaaaaaaaggatctcaagaagatcctttgatct
tttctacggggtctgacgctcagtggaacgaaaactcacgttaagggattttggtcatgagattatcaaaaaggatcttcacctagatcc
ttttaaattaaaaatgaagttttaaatcaatctaaagtatatatgagtaaacttggtctgacagttaccaatgcttaatcagtgaggcac
ctatctcagcgatctgtctatttcgttcatccatagttgcctgactccccgtcgtgtagataactacgatacgggagggcttaccatctggc
cccagtgctgcaatgataccgcgagacccacgctcaccggctccagatttatcagcaataaaccagccagccggaagggccgagcgc
agaagtggtcctgcaactttatccgcctccatccagtctattaattgttgccgggaagctagagtaagtagttcgccagttaatagtttgc
gcaacgttgttgccattgctacaggcatcgtggtgtcacgctcgtcgtttggtatggcttcattcagctccggttcccaacgatcaaggcg
agttacatgatcccccatgttgtgcaaaaaagcggttagctccttcggtcctccgatcgttgtcagaagtaagttggccgcagtgttatc
actcatggttatggcagcactgcataattctcttactgtcatgccatccgtaagatgcttttctgtgactggtgagtactcaaccaagtca
ttctgagaatagtgtatgcggcgaccgagttgctcttgcccggcgtcaatacgggataataccgcgccacatagcagaactttaaaagt
gctcatcattggaaaacgttcttcggggcgaaaactctcaaggatcttaccgctgttgagatccagttcgatgtaacccactcgtgcacc
caactgatcttcagcatcttttactttcaccagcgtttctgggtgagcaaaaacaggaaggcaaaatgccgcaaaaaagggaataagg
gcgacacggaaatgttgaatactcatactcttcctttttcaatattattgaagcatttatcagggttattgtctcatgagcggatacatatt
tgaatgtatttagaaaaataaacaaataggggttccgcgcacatttccccgaaaagtgccac**

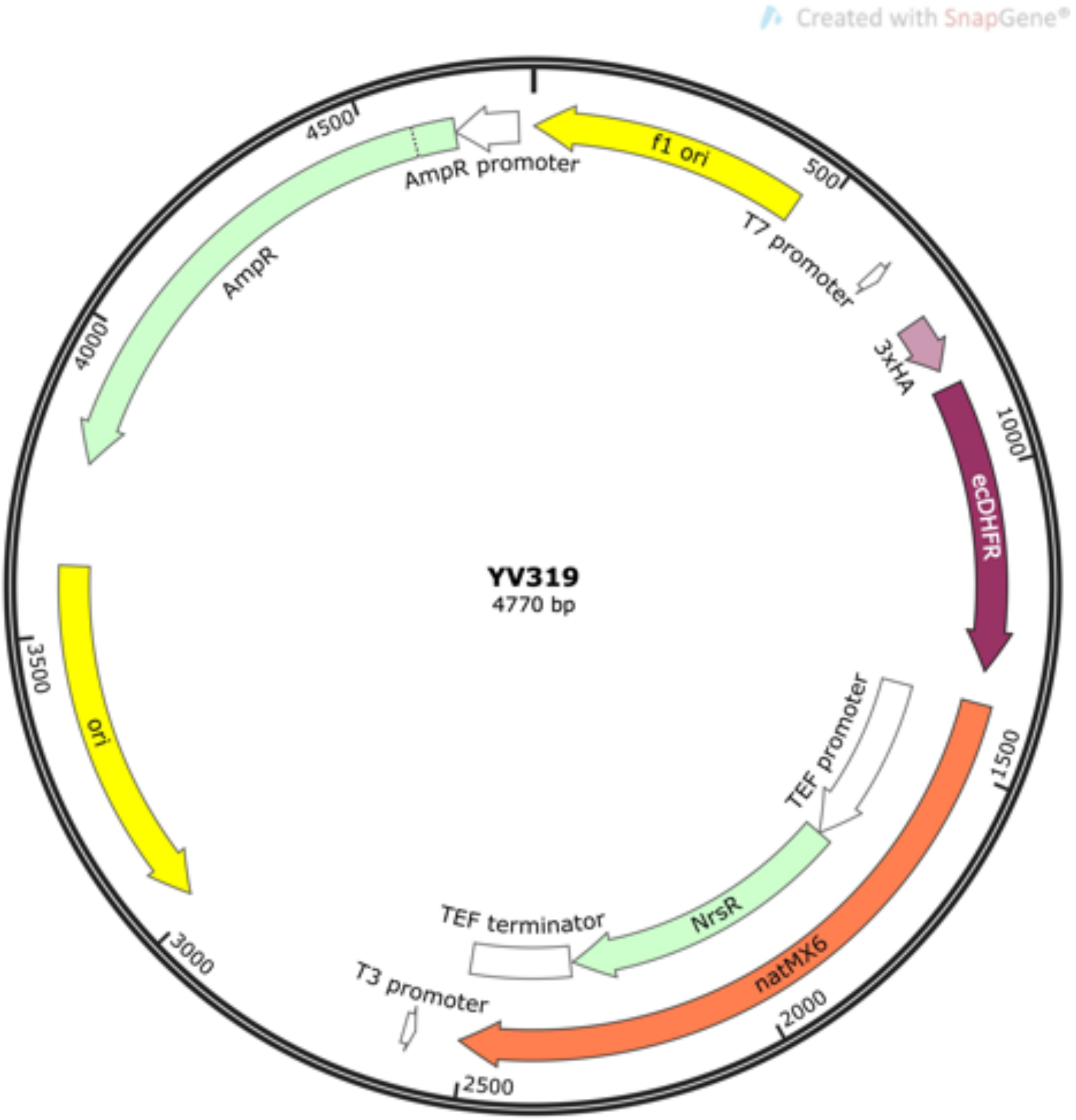

Created with SnapGene®
YV319
4770 bp
f1 ori
500
T7 promoter
3xHA
1000
ecDHFR
TEF promoter
1500
NrsR
natMX6
2000
TEF terminator
T3 promoter
2500
3000
ori
3500
4000
AmpR
4500
AmpR promoter

**YV319: pBS-SKII-3XHA-eDHFR-NAT**

**ctaaattgtaagcgttaatattttgttaaaattcgcgttaaatttttgttaaatcagctcattttttaaccaataggccgaaatcggcaaa
atcccttataaatcaaaagaatagaccgagatagggttgagtgttgttccagtttggaacaagagtccactattaaagaacgtggact
ccaacgtcaaagggcgaaaaaccgtctatcagggcgatggcccactacgtgaaccatcaccctaatcaagttttttggggtcgaggtg
ccgtaaagcactaaatcggaaccctaaagggagcccccgatttagagcttgacggggaaagccggcgaacgtggcgagaaaggaag
ggaagaaagcgaaaggagcgggcgctagggcgctggcaagtgtagcggtcacgctgcgcgtaaccaccacacccgccgcgcttaatg
cgccgctacagggcgcgtcccattcgccattcaggctgcgcaactgttgggaagggcgatcggtgcgggcctcttcgctattacgccagc
tggcgaaagggggatgtgctgcaaggcgattaagttgggtaacgccagggttttcccagtcacgacgttgtaaaacgacggccagtga
gcgcgcgtaatacgactcactatagggcgaattgggtaccgggccccccctcgaggtcgacggtatcgataagcttgatatcgggtcga
cggatccccgggttaattaacatctttTACCCATACGATGTTCCTGACTATGCGGGCTATCCCTATGACGTCCCGG
ACTATGCAGGATCCTATCCATATGACGTTCCAGATTACGCTGCTCAGTGCggttcaggtggatctggttctATG
ATCAGTCTGATTGCGGCGTTAGCGGTAGATCGCGTTATCGGCATGGAAAACGCCATGCCGTGGAACCT
GCCTGCCGATCTCGCCTGGTTTAAACGCAACACCTTAAATAAACCCGTGATTATGGGCCGCCATACCTG
GGAATCAATCGGTCGTCCGTTGCCAGGACGCAAAAATATTATCCTCAGCAGTCAACCGGGTACGGACG
ATCGCGTAACGTGGGTGAAGTCGGTGGATGAAGCCATCGCGGCGTGTGGTGACGTACCAGAAATCAT
GGTGATTGGCGGCGGTCGCGTTTATGAACAGTTCTTGCCAAAAGCGCAAAAACTGTATCTGACGCATA
TCGACGCAGAAGTGGAAGGCGACACCCATTTCCCGGATTACGAGCCGGATGACTGGGAATCGGTATT
CAGCGAATTCCACGATGCTGATGCGCAGAACTCTCACAGCTATTGCTTTGAGATTCTGGAGCGGCGGT
AATATATAACTGTCTAGAAATAAAGAGTATCATCTTTCAAAGGTCACCCGGCCAGCGACATGGAGGCC
CAGAATACCCTCCTTGACAGTCTTGACGTGCGCAGCTCAGGGGCATGATGTGACTGTCGCCCGTACATT
TAGCCCATACATCCCCATGTATAATCATTTGCATCCATACATTTTGATGGCCGCACGGCGCGAAGCAAA
AATTACGGCTCCTCGCTGCAGACCTGCGAGCAGGGAAACGCTCCCCTCACAGACGCGTTGAATTGTCC
CCACGCCGCGCCCCTGTAGAGAAATATAAAAGGTTAGGATTTGCCACTGAGGTTCTTCTTTCATATACT
TCCTTTTAAAATCTTGCTAGGATACAGTTCTCACATCACATCCGAACATAAACAACCATGGGTACCACT
CTTGACGACACGGCTTACCGGTACCGCACCAGTGTCCCGGGGGACGCCGAGGCCATCGAGGCACTGG
ATGGGTCCTTCACCACCGACACCGTCTTCCGCGTCACCGCCACCGGGGACGGCTTCACCCTGCGGGAG
GTGCCGGTGGACCCGCCCCTGACCAAGGTGTTCCCCGACGACGAATCGGACGACGAATCGGACGACG
GGGAGGACGGCGACCCGGACTCCCGGACGTTCGTCGCGTACGGGGACGACGGCGACCTGGCGGGCT
TCGTGGTCGTCTCGTACTCCGGCTGGAACCGCCGGCTGACCGTCGAGGACATCGAGGTCGCCCCGGAG
CACCGGGGGCACGGGGTCGGGCGCGCGTTGATGGGGCTCGCGACGGAGTTCGCCCGCGAGCGGGGC
GCCGGGCACCTCTGGCTGGAGGTCACCAACGTCAACGCACCGGCGATCCACGCGTACCGGCGGATGG
GGTTCACCCTCTGCGGCCTGGACACCGCCCTGTACGACGGCACCGCCTCGGACGGCGAGCAGGCGCTC
TACATGAGCATGCCCTGCCCCTAATCAGTACTGACAATAAAAAGATTCTTGTTTTCAAGAACTTGTCAT
TTGTATAGTTTTTTTATATTGTAGTTGTTCTATTTTAATCAAATGTTAGCGTGATTTATATTTTTTTTCGCC
TCGACATCATCTGCCCAGATGCGAAGTTAAGTGCGCAGAAAGTAATATCATGCGTCAATCGTATGTGA
ATGCTGGTCGCTATACTGCTGTCGATTCGATACTAACGCCGCcactagttctagagcggccgccaccgcggtggagc
tccagcttttgttccctttagtgagggttaattgcgcgcttggcgtaatcatggtcatagctgtttcctgtgtgaaattgttatccgctcaca
attccacacaacatacgagccggaagcataaagtgtaaagcctggggtgcctaatgagtgagctaactcacattaattgcgttgcgct
cactgcccgctttccagtcgggaaacctgtcgtgccagctgcattaatgaatcggccaacgcgcggggagaggcggtttgcgtattggg
cgctcttccgcttcctcgctcactgactcgctgcgctcggtcgttcggctgcggcgagcggtatcagctcactcaaaggcggtaatacggt
tatccacagaatcaggggataacgcaggaaagaacatgtgagcaaaaggccagcaaaaggccaggaaccgtaaaaaggccgcgtt
gctggcgtttttccataggctccgcccccctgacgagcatcacaaaaatcgacgctcaagtcagaggtggcgaaacccgacaggacta
taaagataccaggcgtttccccctggaagctccctcgtgcgctctcctgttccgaccctgccgcttaccggatacctgtccgcctttctccc**

ttcgggaagcgtggcgctttctcatagctcacgctgtaggtatctcagttcggtgtaggtcgttcgctccaagctgggctgtgtgcacgaa
ccccccgttcagcccgaccgctgcgccttatccggtaactatcgtcttgagtccaacccggtaagacacgacttatcgccactggcagca
gccactggtaacaggattagcagagcgaggtatgtaggcggtgctacagagttcttgaagtggtggcctaactacggctacactagaa
ggacagtatttggtatctgcgctctgctgaagccagttaccttcggaaaaagagttggtagctcttgatccggcaaacaaaccaccgct
ggtagcggtggtttttttgtttgcaagcagcagattacgcgcagaaaaaaaggatctcaagaagatcctttgatcttttctacggggtct
gacgctcagtggaacgaaaactcacgttaagggattttggtcatgagattatcaaaaaggatcttcacctagatccttttaaattaaaa
atgaagttttaaatcaatctaaagtatatatgagtaaacttggtctgacagttaccaatgcttaatcagtgaggcacctatctcagcgat
ctgtctatttcgttcatccatagttgcctgactccccgtcgtgtagataactacgatacgggagggcttaccatctggccccagtgctgca
atgataccgcgagacccacgctcaccggctccagatttatcagcaataaaccagccagccggaagggccgagcgcagaagtggtcct
gcaactttatccgcctccatccagtctattaattgttgccgggaagctagagtaagtagttcgccagttaatagtttgcgcaacgttgttg
ccattgctacaggcatcgtggtgtcacgctcgtcgtttggtatggcttcattcagctccggttcccaacgatcaaggcgagttacatgatc
ccccatgttgtgcaaaaaagcggttagctccttcggtcctccgatcgttgtcagaagtaagttggccgcagtgttatcactcatggttatg
gcagcactgcataattctcttactgtcatgccatccgtaagatgcttttctgtgactggtgagtactcaaccaagtcattctgagaatagt
gtatgcggcgaccgagttgctcttgcccggcgtcaatacgggataataccgcgccacatagcagaactttaaaagtgctcatcattgga
aaacgttcttcggggcgaaaactctcaaggatcttaccgctgttgagatccagttcgatgtaacccactcgtgcacccaactgatcttca
gcatcttttactttcaccagcgtttctgggtgagcaaaaacaggaaggcaaaatgccgcaaaaaagggaataagggcgacacggaa
atgttgaatactcatactcttcctttttcaatattattgaagcatttatcagggttattgtctcatgagcggatacatatttgaatgtattta
gaaaaataaacaaataggggttccgcgcacatttccccgaaaagtgccac

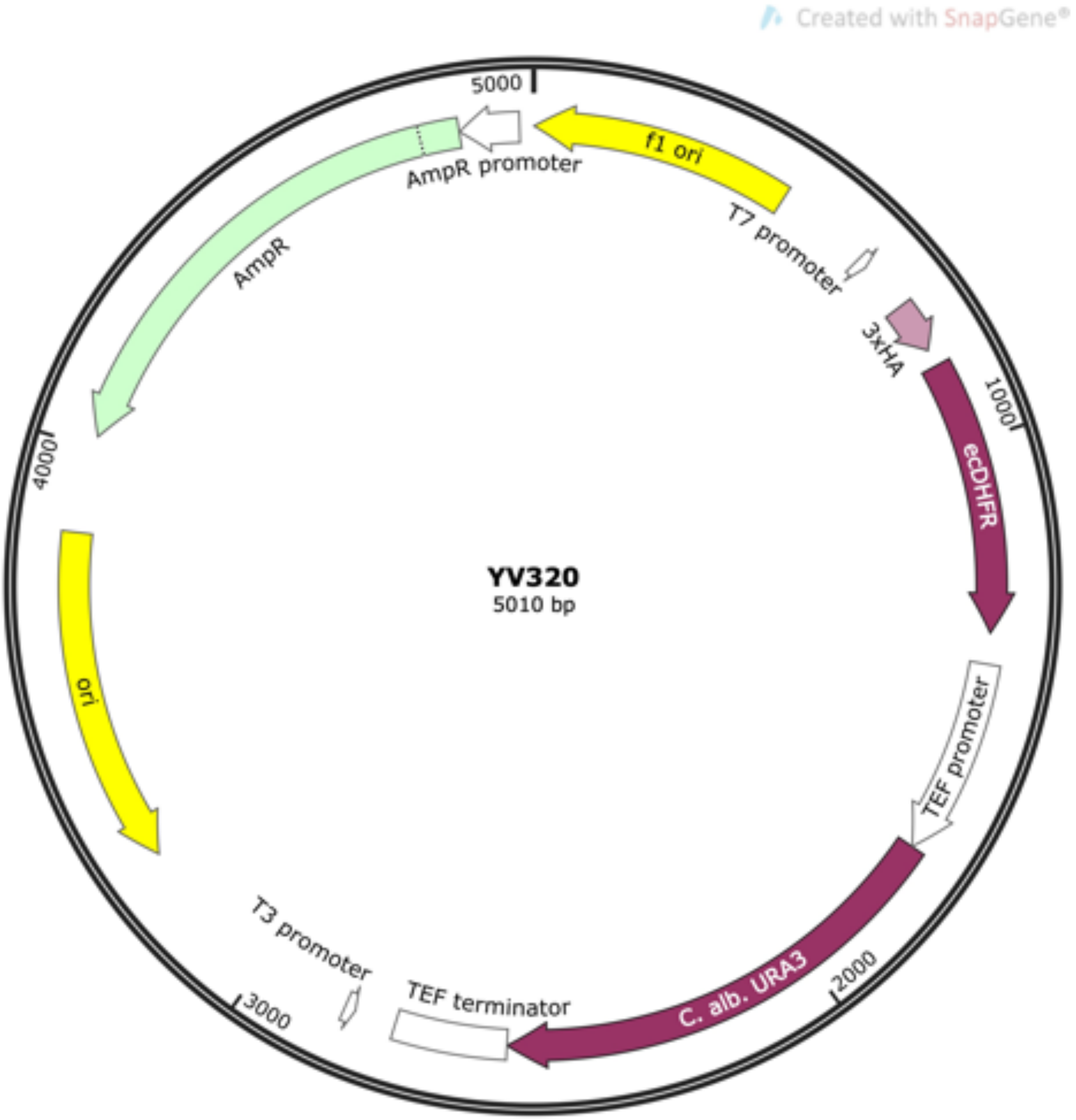

Created with SnapGene®
5000
f1 ori
AmpR promoter
T7 promoter
AmpR
3xHA
1000
4000
ecDHFR
YV320
5010 bp
ori
TEF promoter
T3 promoter
2000
C. alb. URA3
3000
TEF terminator

**YV320: pBS-SKII-3XHA-eDHFR-URA**

**ctaaattgtaagcgttaatattttgttaaaattcgcgttaaatttttgttaaatcagctcattttttaaccaataggccgaaatcggcaaa
atcccttataaatcaaaagaatagaccgagatagggttgagtgttgttccagtttggaacaagagtccactattaaagaacgtggact
ccaacgtcaaagggcgaaaaaccgtctatcagggcgatggcccactacgtgaaccatcaccctaatcaagttttttggggtcgaggtg
ccgtaaagcactaaatcggaaccctaaagggagcccccgatttagagcttgacggggaaagccggcgaacgtggcgagaaaggaag
ggaagaaagcgaaaggagcgggcgctagggcgctggcaagtgtagcggtcacgctgcgcgtaaccaccacacccgccgcgcttaatg
cgccgctacagggcgcgtcccattcgccattcaggctgcgcaactgttgggaagggcgatcggtgcgggcctcttcgctattacgccagc
tggcgaaagggggatgtgctgcaaggcgattaagttgggtaacgccagggttttcccagtcacgacgttgtaaaacgacggccagtga
gcgcgcgtaatacgactcactatagggcgaattgggtaccgggccccccctcgaggtcgacggtatcgataagcttgatatcgggtcga
cggatccccgggttaattaacatctttTACCCATACGATGTTCCTGACTATGCGGGCTATCCCTATGACGTCCCGG
ACTATGCAGGATCCTATCCATATGACGTTCCAGATTACGCTGCTCAGTGCggttcaggtggatctggttctATG
ATCAGTCTGATTGCGGCGTTAGCGGTAGATCGCGTTATCGGCATGGAAAACGCCATGCCGTGGAACCT
GCCTGCCGATCTCGCCTGGTTTAAACGCAACACCTTAAATAAACCCGTGATTATGGGCCGCCATACCTG
GGAATCAATCGGTCGTCCGTTGCCAGGACGCAAAAATATTATCCTCAGCAGTCAACCGGGTACGGACG
ATCGCGTAACGTGGGTGAAGTCGGTGGATGAAGCCATCGCGGCGTGTGGTGACGTACCAGAAATCAT
GGTGATTGGCGGCGGTCGCGTTTATGAACAGTTCTTGCCAAAAGCGCAAAAACTGTATCTGACGCATA
TCGACGCAGAAGTGGAAGGCGACACCCATTTCCCGGATTACGAGCCGGATGACTGGGAATCGGTATT
CAGCGAATTCCACGATGCTGATGCGCAGAACTCTCACAGCTATTGCTTTGAGATTCTGGAGCGGCGGT
AATATATAACTGTCTAGAAATAAAGAGTATCATCTTTCAAAGGTCACCCGGCCAGCGACATGGAGGCC
CAGAATACCCTCCTTGACAGTCTTGACGTGCGCAGCTCAGGGGCATGATGTGACTGTCGCCCGTACATT
TAGCCCATACATCCCCATGTATAATCATTTGCATCCATACATTTTGATGGCCGCACGGCGCGAAGCAAA
AATTACGGCTCCTCGCTGCAGACCTGCGAGCAGGGAAACGCTCCCCTCACAGACGCGTTGAATTGTCC
CCACGCCGCGCCCCTGTAGAGAAATATAAAAGGTTAGGATTTGCCACTGAGGTTCTTCTTTCATATACT
TCCTTTTAAAATCTTGTTAGGATACAGTTCTCACATCACATCCGAACATAAACAACCATGACAGTCAAC
ACTAAGACCTATAGTGAGAGAGCAGAAACTCATGCCTCACCAGTAGCACAACGATTATTTCGATTAAT
GGAACTGAAGAAAACCAATTTATGTGCATCAATTGATGTTGATACCACTAAGGAATTCCTTGAATTAA
TTGATAAATTGGGTCCTTATGTATGCTTAATCAAGACTCATATTGATATAATCAATGATTTTTCCTATGA
ATCCACTATTGAACCATTATTAGAACTTTCACGTAAACATCAATTTATGATTTTTGAAGATAGAAAATTT
GCTGATATTGGTAATACCGTGAAGAAACAATATATTGGTGGAGTTTATAAAATTAGTAGTTGGGCAG
ATATTACTAATGCTCATGGTGTCACTGGGAATGGAGTAGTTGAAGGATTAAAACAGGGAGCTAAAGA
AACCACCACCAACCAAGAGCCAAGAGGGTTATTGATGTTAGCTGAATTATCATCAGTGGGATCATTAG
CATATGGAGAATATTCTCAAAAAACTGTTGAAATTGCTAAATCCGATAAGGAATTTGTTATTGGATTTA
TTGCCCAACGTGATATGGGTGGACAAGAAGAAGGATTTGATTGGCTTATTATGACACCTGGAGTTGG
ATTAGATGATAAAGGTGATGGATTAGGACAACAATATAGAACTGTTGATGAAGTTGTTAGCACTGGA
ACTGATATTATCATTGTTGGTAGAGGATTGTTTGGTAAAGGAAGAGATCCAGATATTGAAGGTAAAA
GGTATAGAGATGCTGGTTGGAATGCTTATTTGAAAAAGACTGGCCAATTATAATCAGTACTGACAATA
AAAAGATTCTTGTTTTCAAGAACTTGTCATTTGTATAGTTTTTTTATATTGTAGTTGTTCTATTTTAATCA
AATGTTAGCGTGATTTATATTTTTTTTCGCCTCGACATCATCTGCCCAGATGCGAAGTTAAGTGCGCAG
AAAGTAATATCATGCGTCAATCGTATGTGAATGCTGGTCGCTATACTGCTGTCGATTCGATACTAACGC
CGCcactagttctagagcggccgccaccgcggtggagctccagcttttgttccctttagtgagggttaattgcgcgcttggcgtaatcat
ggtcatagctgtttcctgtgtgaaattgttatccgctcacaattccacacaacatacgagccggaagcataaagtgtaaagcctggggt
gcctaatgagtgagctaactcacattaattgcgttgcgctcactgcccgctttccagtcgggaaacctgtcgtgccagctgcattaatga
atcggccaacgcgcggggagaggcggtttgcgtattgggcgctcttccgcttcctcgctcactgactcgctgcgctcggtcgttcggctgc**

**ggcgagcggtatcagctcactcaaaggcggtaatacggttatccacagaatcaggggataacgcaggaaagaacatgtgagcaaaa
ggccagcaaaaggccaggaaccgtaaaaaggccgcgttgctggcgtttttccataggctccgcccccctgacgagcatcacaaaaatc
gacgctcaagtcagaggtggcgaaacccgacaggactataaagataccaggcgtttccccctggaagctccctcgtgcgctctcctgtt
ccgaccctgccgcttaccggatacctgtccgcctttctcccttcgggaagcgtggcgctttctcatagctcacgctgtaggtatctcagttc
ggtgtaggtcgttcgctccaagctgggctgtgtgcacgaaccccccgttcagcccgaccgctgcgccttatccggtaactatcgtcttgag
tccaacccggtaagacacgacttatcgccactggcagcagccactggtaacaggattagcagagcgaggtatgtaggcggtgctacag
agttcttgaagtggtggcctaactacggctacactagaaggacagtatttggtatctgcgctctgctgaagccagttaccttcggaaaa
agagttggtagctcttgatccggcaaacaaaccaccgctggtagcggtggtttttttgtttgcaagcagcagattacgcgcagaaaaaa
aggatctcaagaagatcctttgatcttttctacggggtctgacgctcagtggaacgaaaactcacgttaagggattttggtcatgagatt
atcaaaaaggatcttcacctagatccttttaaattaaaaatgaagttttaaatcaatctaaagtatatatgagtaaacttggtctgaca
gttaccaatgcttaatcagtgaggcacctatctcagcgatctgtctatttcgttcatccatagttgcctgactccccgtcgtgtagataact
acgatacgggagggcttaccatctggccccagtgctgcaatgataccgcgagacccacgctcaccggctccagatttatcagcaataa
accagccagccggaagggccgagcgcagaagtggtcctgcaactttatccgcctccatccagtctattaattgttgccgggaagctaga
gtaagtagttcgccagttaatagtttgcgcaacgttgttgccattgctacaggcatcgtggtgtcacgctcgtcgtttggtatggcttcatt
cagctccggttcccaacgatcaaggcgagttacatgatcccccatgttgtgcaaaaaagcggttagctccttcggtcctccgatcgttgt
cagaagtaagttggccgcagtgttatcactcatggttatggcagcactgcataattctcttactgtcatgccatccgtaagatgcttttct
gtgactggtgagtactcaaccaagtcattctgagaatagtgtatgcggcgaccgagttgctcttgcccggcgtcaatacgggataatac
cgcgccacatagcagaactttaaaagtgctcatcattggaaaacgttcttcggggcgaaaactctcaaggatcttaccgctgttgagat
ccagttcgatgtaacccactcgtgcacccaactgatcttcagcatcttttactttcaccagcgtttctgggtgagcaaaaacaggaaggc
aaaatgccgcaaaaaagggaataagggcgacacggaaatgttgaatactcatactcttcctttttcaatattattgaagcatttatcag
ggttattgtctcatgagcggatacatatttgaatgtatttagaaaaataaacaaataggggttccgcgcacatttccccgaaaagtgcc
ac**

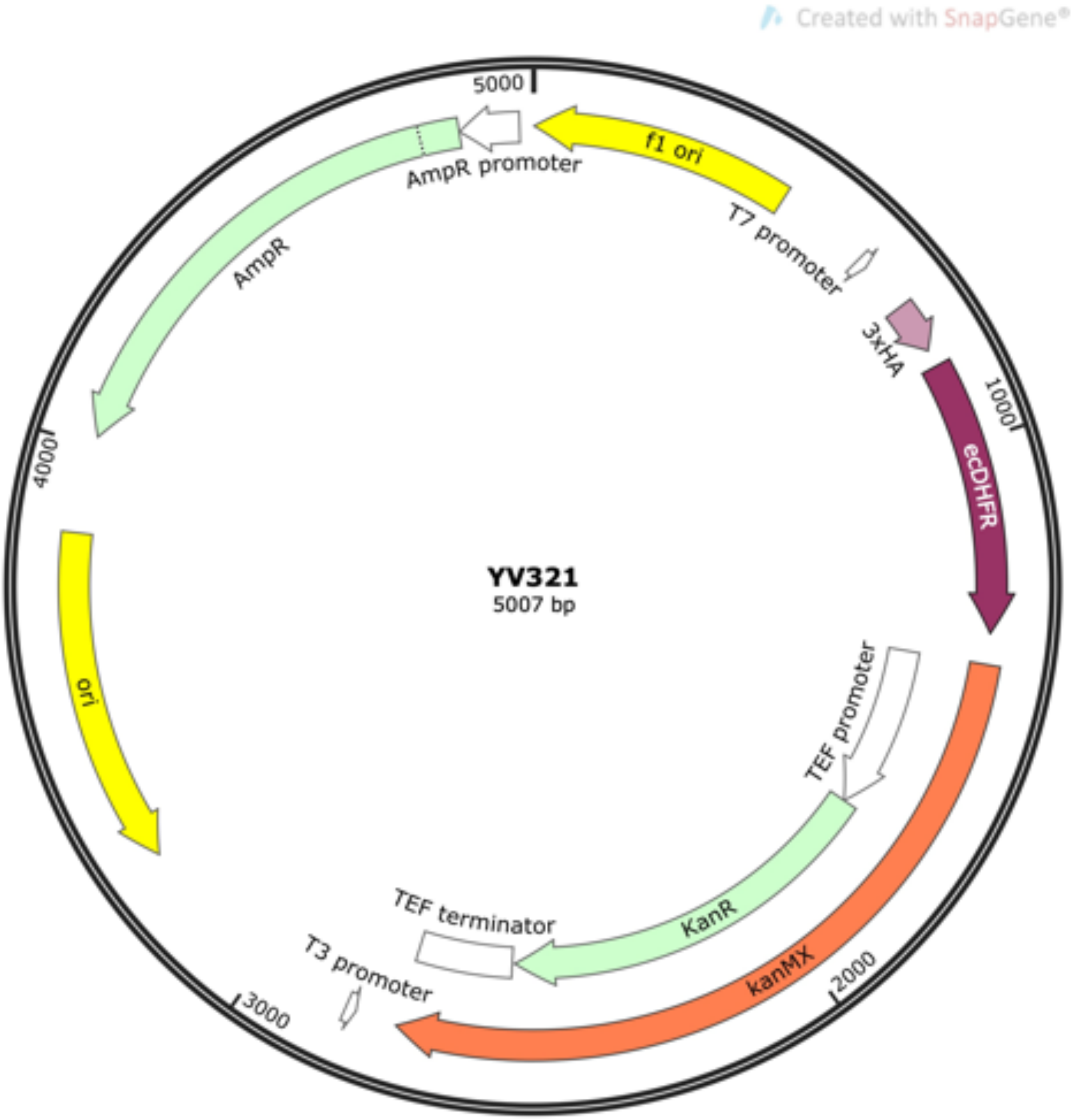
Created with SnapGene®
YV321
5007 bp
5000
1000
2000
3000
4000
f1 ori
AmpR promoter
AmpR
T7 promoter
3xHA
ecDHFR
TEF promoter
kanMX
KanR
TEF terminator
T3 promoter
ori

**YV321: pBS-SKII-3XHA-eDHFR-Kan**

**ctaaattgtaagcgttaatattttgttaaaattcgcgttaaatttttgttaaatcagctcattttttaaccaataggccgaaatcggcaaa
atcccttataaatcaaaagaatagaccgagatagggttgagtgttgttccagtttggaacaagagtccactattaaagaacgtggact
ccaacgtcaaagggcgaaaaaccgtctatcagggcgatggcccactacgtgaaccatcaccctaatcaagttttttggggtcgaggtg
ccgtaaagcactaaatcggaaccctaaagggagcccccgatttagagcttgacggggaaagccggcgaacgtggcgagaaaggaag
ggaagaaagcgaaaggagcgggcgctagggcgctggcaagtgtagcggtcacgctgcgcgtaaccaccacacccgccgcgcttaatg
cgccgctacagggcgcgtcccattcgccattcaggctgcgcaactgttgggaagggcgatcggtgcgggcctcttcgctattacgccagc
tggcgaaagggggatgtgctgcaaggcgattaagttgggtaacgccagggttttcccagtcacgacgttgtaaaacgacggccagtga
gcgcgcgtaatacgactcactatagggcgaattgggtaccgggccccccctcgaggtcgacggtatcgataagcttgatatcgggtcga
cggatccccgggttaattaacatctttTACCCATACGATGTTCCTGACTATGCGGGCTATCCCTATGACGTCCCGG
ACTATGCAGGATCCTATCCATATGACGTTCCAGATTACGCTGCTCAGTGCGGTTCAGGTGGATCTGGTT
CTATGATCAGTCTGATTGCGGCGTTAGCGGTAGATCGCGTTATCGGCATGGAAAACGCCATGCCGTGG
AACCTGCCTGCCGATCTCGCCTGGTTTAAACGCAACACCTTAAATAAACCCGTGATTATGGGCCGCCAT
ACCTGGGAATCAATCGGTCGTCCGTTGCCGGGACGCAAAAATATTATCCTCAGCAGTCAACCGGGTAC
GGACGATCGCGTAACGTGGGTGAAGTCGGTGGATGAAGCCATCGCGGCGTGTGGTGACGTACCAGA
AATCATGGTGATTGGCGGCGGTCGCGTTTATGAACAGTTCTTGCCAAAAGCGCAAAAACTGTATCTGA
CGCATATCGACGCAGAAGTGGAAGGCGACACCCATTTCCCGGATTACGAGCCGGATGACTGGGAATC
GGTATTCAGCGAATTCCACGATGCTGATGCGCAGAACTCTCACAGCTATTGCTTTGAGATTCTGGAGC
GGCGGTAATATATAACTGTCTAGAAATAAAGAGTATCATCTTTCAAAGGTCACCCGGCCAGCGACATG
GAGGCCCAGAATACCCTCCTTGACAGTCTTGACGTGCGCAGCTCAGGGGCATGATGTGACTGTCGCCC
GTACATTTAGCCCATACATCCCCATGTATAATCATTTGCATCCATACATTTTGATGGCCGCACGGCGCG
AAGCAAAAATTACGGCTCCTCGCTGCAGACCTGCGAGCAGGGAAACGCTCCCCTCACAGACGCGTTGA
ATTGTCCCCACGCCGCGCCCCTGTAGAGAAATATAAAAGGTTAGGATTTGCCACTGAGGTTCTTCTTTC
ATATACTTCCTTTTAAAATCTTGCTAGGATACAGTTCTCACATCACATCCGAACATAAACAACCATGGG
TAAGGAAAAGACTCACGTTTCGAGGCCGCGATTAAATTCCAACATGGATGCTGATTTATATGGGTATA
AATGGGCTCGCGATAATGTCGGGCAATCAGGTGCGACAATCTATCGATTGTATGGGAAGCCCGATGC
GCCAGAGTTGTTTCTGAAACATGGCAAAGGTAGCGTTGCCAATGATGTTACAGATGAGATGGTCAGA
CTAAACTGGCTGACGGAATTTATGCCTCTTCCGACCATCAAGCATTTTATCCGTACTCCTGATGATGCAT
GGTTACTCACCACTGCGATCCCCGGCAAAACAGCATTCCAGGTATTAGAAGAATATCCTGATTCAGGT
GAAAATATTGTTGATGCGCTGGCAGTGTTCCTGCGCCGGTTGCATTCGATTCCTGTTTGTAATTGTCCTT
TTAACAGCGATCGCGTATTTCGTCTCGCTCAGGCGCAATCACGAATGAATAACGGTTTGGTTGATGCG
AGTGATTTTGATGACGAGCGTAATGGCTGGCCTGTTGAACAAGTCTGGAAAGAAATGCATAAGCTTTT
GCCATTCTCACCGGATTCAGTCGTCACTCATGGTGATTTCTCACTTGATAACCTTATTTTTGACGAGGGG
AAATTAATAGGTTGTATTGATGTTGGACGAGTCGGAATCGCAGACCGATACCAGGATCTTGCCATCCT
ATGGAACTGCCTCGGTGAGTTTTCTCCTTCATTACAGAAACGGCTTTTTCAAAAATATGGTATTGATAA
TCCTGATATGAATAAATTGCAGTTTCATTTGATGCTCGATGAGTTTTTCTAATCAGTACTGACAATAAA
AAGATTCTTGTTTTCAAGAACTTGTCATTTGTATAGTTTTTTTATATTGTAGTTGTTCTATTTTAATCAAA
TGTTAGCGTGATTTATATTTTTTTTCGCCTCGACATCATCTGCCCAGATGCGAAGTTAAGTGCGCAGAA
AGTAATATCATGCGTCAATCGTATGTGAATGCTGGTCGCTATACTGCTGTCGATTCGATACTAACGCCG
Ccactagttctagagcggccgccaccgcggtggagctccagcttttgttccctttagtgagggttaattgcgcgcttggcgtaatcatggt
catagctgtttcctgtgtgaaattgttatccgctcacaattccacacaacatacgagccggaagcataaagtgtaaagcctggggtgcc
taatgagtgagctaactcacattaattgcgttgcgctcactgcccgctttccagtcgggaaacctgtcgtgccagctgcattaatgaatc
ggccaacgcgcggggagaggcggtttgcgtattgggcgctcttccgcttcctcgctcactgactcgctgcgctcggtcgttcggctgcggc**

gagcggtatcagctcactcaaaggcggtaatacggttatccacagaatcaggggataacgcaggaaagaacatgtgagcaaaaggc
cagcaaaaggccaggaaccgtaaaaaggccgcgttgctggcgtttttccataggctccgcccccctgacgagcatcacaaaaatcgac
gctcaagtcagaggtggcgaaacccgacaggactataaagataccaggcgtttccccctggaagctccctcgtgcgctctcctgttccg
accctgccgcttaccggatacctgtccgcctttctcccttcgggaagcgtggcgctttctcatagctcacgctgtaggtatctcagttcggt
gtaggtcgttcgctccaagctgggctgtgtgcacgaaccccccgttcagcccgaccgctgcgccttatccggtaactatcgtcttgagtcc
aacccggtaagacacgacttatcgccactggcagcagccactggtaacaggattagcagagcgaggtatgtaggcggtgctacagag
ttcttgaagtggtggcctaactacggctacactagaaggacagtatttggtatctgcgctctgctgaagccagttaccttcggaaaaag
agttggtagctcttgatccggcaaacaaaccaccgctggtagcggtggtttttttgtttgcaagcagcagattacgcgcagaaaaaaag
gatctcaagaagatcctttgatcttttctacggggtctgacgctcagtggaacgaaaactcacgttaagggattttggtcatgagattat
caaaaaggatcttcacctagatccttttaaattaaaaatgaagttttaaatcaatctaaagtatatatgagtaaacttggtctgacagtt
accaatgcttaatcagtgaggcacctatctcagcgatctgtctatttcgttcatccatagttgcctgactccccgtcgtgtagataactac
gatacgggagggcttaccatctggccccagtgctgcaatgataccgcgagacccacgctcaccggctccagatttatcagcaataaacc
agccagccggaagggccgagcgcagaagtggtcctgcaactttatccgcctccatccagtctattaattgttgccgggaagctagagta
agtagttcgccagttaatagtttgcgcaacgttgttgccattgctacaggcatcgtggtgtcacgctcgtcgtttggtatggcttcattcag
ctccggttcccaacgatcaaggcgagttacatgatcccccatgttgtgcaaaaaagcggttagctccttcggtcctccgatcgttgtcag
aagtaagttggccgcagtgttatcactcatggttatggcagcactgcataattctcttactgtcatgccatccgtaagatgcttttctgtga
ctggtgagtactcaaccaagtcattctgagaatagtgtatgcggcgaccgagttgctcttgcccggcgtcaatacgggataataccgcg
ccacatagcagaactttaaaagtgctcatcattggaaaacgttcttcggggcgaaaactctcaaggatcttaccgctgttgagatccag
ttcgatgtaacccactcgtgcacccaactgatcttcagcatcttttactttcaccagcgtttctgggtgagcaaaaacaggaaggcaaaa
tgccgcaaaaaagggaataagggcgacacggaaatgttgaatactcatactcttcctttttcaatattattgaagcatttatcagggtta
ttgtctcatgagcggatacatatttgaatgtatttagaaaaataaacaaataggggttccgcgcacatttccccgaaaagtgccac

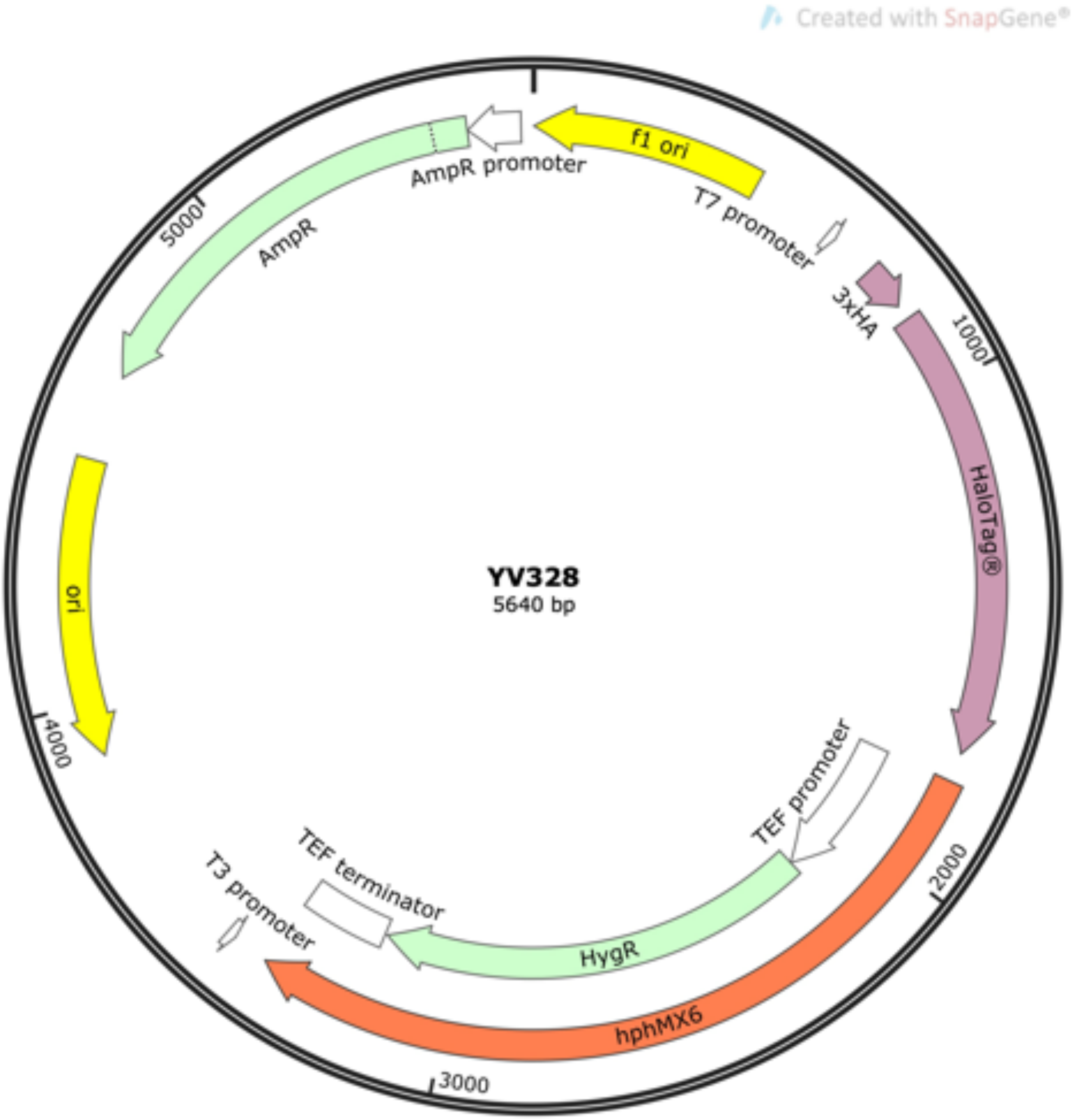
Created with SnapGene®
YV328
5640 bp
f1 ori
AmpR promoter
T7 promoter
3xHA
1000
HaloTag®
TEF promoter
2000
hphMX6
HygR
TEF terminator
T3 promoter
3000
4000
ori
AmpR
5000

**YV328: pBS-SKII-3XHA-HALO-Hygromycin**

**ctaaattgtaagcgttaatattttgttaaaattcgcgttaaatttttgttaaatcagctcattttttaaccaataggccgaaatcggcaaa
atcccttataaatcaaaagaatagaccgagatagggttgagtgttgttccagtttggaacaagagtccactattaaagaacgtggact
ccaacgtcaaagggcgaaaaaccgtctatcagggcgatggcccactacgtgaaccatcaccctaatcaagttttttggggtcgaggtg
ccgtaaagcactaaatcggaaccctaaagggagcccccgatttagagcttgacggggaaagccggcgaacgtggcgagaaaggaag
ggaagaaagcgaaaggagcgggcgctagggcgctggcaagtgtagcggtcacgctgcgcgtaaccaccacacccgccgcgcttaatg
cgccgctacagggcgcgtcccattcgccattcaggctgcgcaactgttgggaagggcgatcggtgcgggcctcttcgctattacgccagc
tggcgaaagggggatgtgctgcaaggcgattaagttgggtaacgccagggttttcccagtcacgacgttgtaaaacgacggccagtga
gcgcgcgtaatacgactcactatagggcgaattgggtaccgggccccccctcgaggtcgacggtatcgataagcttgatatcgggtcga
cggatccccgggttaattaacatctttTACCCATACGATGTTCCTGACTATGCGGGCTATCCGTATGACGTCCCGG
ACTATGCAGGATCCTATCCATATGACGTTCCAGATTACGCTGCTCAGTGCggttcaggtggatctggttctATG
GCAGAAATCGGTACTGGCTTTCCATTCGACCCGCATTATGTGGAAGTCCTGGGCGAGAGAATGCATTA
CGTTGACGTGGGTCCGAGAGATGGAACTCCGGTCCTTTTTCTGCACGGGAATCCTACAAGCTCTTATGT
TTGGCGCAATATCATCCCTCATGTAGCTCCGACGCATCGCTGTATTGCGCCGGACCTGATTGGTATGGG
AAAATCTGATAAACCAGACCTGGGTTACTTTTTCGATGATCATGTGCGTTTCATGGATGCCTTCATTGA
GGCATTAGGGCTTGAAGAAGTCGTCTTGGTGATTCATGATTGGGGCTCAGCTCTGGGATTTCACTGGG
CTAAAAGAAATCCTGAACGCGTAAAAGGCATCGCGTTTATGGAGTTCATTCGTCCAATTCCGACTTGG
GATGAATGGCCTGAGTTCGCGAGAGAAACATTTCAAGCATTTCGCACGACCGATGTAGGCCGGAAGT
TAATCATCGATCAGAATGTCTTTATCGAAGGGACATTGCCGATGGGAGTCGTTCGTCCGTTAACAGAA
GTCGAAATGGATCACTATAGAGAACCTTTTCTTAATCCTGTGGACAGAGAGCCGCTGTGGCGGTTTCC
GAACGAACTGCCGATTGCAGGCGAGCCTGCTAACATTGTAGCGCTGGTTGAAGAGTATATGGATTGG
CTTCATCAGTCTCCAGTTCCGAAGTTATTGTTTTGGGGTACGCCTGGCGTGCTTATTCCACCGGCCGAA
GCGGCACGTTTGGCAAAAAGCCTGCCAAATTGCAAAGCCGTTGACATTGGCCCTGGACTTAACTTGCT
TCAAGAGGATAACCCGGACTTAATCGGGAGCGAAATTGCCCGGTGGCTTTCTACCTTAGAAATCAGCG
GCtaaTATATAACTGTCTAGAAATAAAGAGTATCATCTTTCAAAGGTCACCCGGCCAGCGACATGGAG
GCCCAGAATACCCTCCTTGACAGTCTTGACGTGCGCAGCTCAGGGGCATGATGTGACTGTCGCCCGTA
CATTTAGCCCATACATCCCCATGTATAATCATTTGCATCCATACATTTTGATGGCCGCACGGCGCGAAG
CAAAAATTACGGCTCCTCGCTGCgGACCTGCGAGCAGGGAAACGCTCCCCTCACAGACGCGTTGAATT
GTCCCCACGCCGCGCCCCTGTAGAGAAATATAAAAGGTTAGGATTTGCCACTGAGGTTCTTCTTTCATA
TACTTCCTTTTAAAATCTTGCTAGGATACAGTTCTCACATCACATCCGAACATAAACAACCATGGGTAA
AAAGCCTGAACTCACCGCGACGTCTGTCGAGAAGTTTCTGATCGAAAAGTTCGACAGCGTCTCCGACC
TGATGCAGCTCTCGGAGGGCGAAGAATCTCGTGCTTTCAGCTTCGATGTAGGAGGGCGTGGATATGTC
CTGCGGGTAAATAGCTGCGCCGATGGTTTCTACAAAGATCGTTATGTTTATCGGCACTTTGCATCGGCC
GCGCTCCCGATTCCGGAAGTGCTTGACATTGGGGAATTCAGCGAGAGCCTGACCTATTGCATCTCCCG
CCGTGCACAGGGTGTCACGTTGCAAGACCTGCCTGAAACCGAACTGCCCGCTGTTCTGCAGCCGGTCG
CGGAGGCCATGGATGCGATCGCTGCGGCCGATCTTAGCCAGACGAGCGGGTTCGGCCCATTCGGACC
GCAAGGAATCGGTCAATACACTACATGGCGTGATTTCATATGCGCGATTGCTGATCCCCATGTGTATCA
CTGGCAAACTGTGATGGACGACACCGTCAGTGCGTCCGTCGCGCAGGCTCTCGATGAGCTGATGCTTT
GGGCCGAGGACTGCCCCGAAGTCCGGCACCTCGTGCACGCGGATTTCGGCTCCAACAATGTCCTGACG
GACAATGGCCGCATAACAGCGGTCATTGACTGGAGCGAGGCGATGTTCGGGGATTCCCAATACGAGG
TCGCCAACATCTTCTTCTGGAGGCCGTGGTTGGCTTGTATGGAGCAGCAGACGCGCTACTTCGAGCGG
AGGCATCCGGAGCTTGCAGGATCGCCGCGGCTCCGGGCGTATATGCTCCGCATTGGTCTTGACCAACT
CTATCAGAGCTTGGTTGACGGCAATTTCGATGATGCAGCTTGGGCGCAGGGTCGATGCGACGCAATC**

**GTCCGATCCGGAGCCGGGACTGTCGGGCGTACACAAATCGCCCGCAGAAGCGCGGCCGTCTGGACCG
ATGGCTGTGTAGAAGTACTCGCCGATAGTGGAAACCGACGCCCCAGCACTCGTCCGAGGGCAAAGGA
ATAATCAGTACTGACAATAAAAAGATTCTTGTTTTCAAGAACTTGTCATTTGTATAGTTTTTTTATATTG
TAGTTGTTCTATTTTAATCAAATGTTAGCGTGATTTATATTTTTTTTCGCCTCGACATCATCTGCCCAGAT
GCGAAGTTAAGTGCGCAGAAAGTAATATCATGCGTCAATCGTATGTGAATGCTGGTCGCTATACTGCT
GTCGATTCGATACTAACGCCGCcactagttctagagcggccgccaccgcggtggagctccagcttttgttccctttagtgaggg
ttaattgcgcgcttggcgtaatcatggtcatagctgtttcctgtgtgaaattgttatccgctcacaattccacacaacatacgagccggaa
gcataaagtgtaaagcctggggtgcctaatgagtgagctaactcacattaattgcgttgcgctcactgcccgctttccagtcgggaaac
ctgtcgtgccagctgcattaatgaatcggccaacgcgcggggagaggcggtttgcgtattgggcgctcttccgcttcctcgctcactgact
cgctgcgctcggtcgttcggctgcggcgagcggtatcagctcactcaaaggcggtaatacggttatccacagaatcaggggataacgc
aggaaagaacatgtgagcaaaaggccagcaaaaggccaggaaccgtaaaaaggccgcgttgctggcgtttttccataggctccgccc
ccctgacgagcatcacaaaaatcgacgctcaagtcagaggtggcgaaacccgacaggactataaagataccaggcgtttccccctgg
aagctccctcgtgcgctctcctgttccgaccctgccgcttaccggatacctgtccgcctttctcccttcgggaagcgtggcgctttctcatag
ctcacgctgtaggtatctcagttcggtgtaggtcgttcgctccaagctgggctgtgtgcacgaaccccccgttcagcccgaccgctgcgcc
ttatccggtaactatcgtcttgagtccaacccggtaagacacgacttatcgccactggcagcagccactggtaacaggattagcagagc
gaggtatgtaggcggtgctacagagttcttgaagtggtggcctaactacggctacactagaaggacagtatttggtatctgcgctctgct
gaagccagttaccttcggaaaaagagttggtagctcttgatccggcaaacaaaccaccgctggtagcggtggtttttttgtttgcaagc
agcagattacgcgcagaaaaaaaggatctcaagaagatcctttgatcttttctacggggtctgacgctcagtggaacgaaaactcacg
ttaagggattttggtcatgagattatcaaaaaggatcttcacctagatccttttaaattaaaaatgaagttttaaatcaatctaaagtat
atatgagtaaacttggtctgacagttaccaatgcttaatcagtgaggcacctatctcagcgatctgtctatttcgttcatccatagttgcct
gactccccgtcgtgtagataactacgatacgggagggcttaccatctggccccagtgctgcaatgataccgcgagacccacgctcaccg
gctccagatttatcagcaataaaccagccagccggaagggccgagcgcagaagtggtcctgcaactttatccgcctccatccagtctat
taattgttgccgggaagctagagtaagtagttcgccagttaatagtttgcgcaacgttgttgccattgctacaggcatcgtggtgtcacg
ctcgtcgtttggtatggcttcattcagctccggttcccaacgatcaaggcgagttacatgatcccccatgttgtgcaaaaaagcggttag
ctccttcggtcctccgatcgttgtcagaagtaagttggccgcagtgttatcactcatggttatggcagcactgcataattctcttactgtca
tgccatccgtaagatgcttttctgtgactggtgagtactcaaccaagtcattctgagaatagtgtatgcggcgaccgagttgctcttgccc
ggcgtcaatacgggataataccgcgccacatagcagaactttaaaagtgctcatcattggaaaacgttcttcggggcgaaaactctca
aggatcttaccgctgttgagatccagttcgatgtaacccactcgtgcacccaactgatcttcagcatcttttactttcaccagcgtttctgg
gtgagcaaaaacaggaaggcaaaatgccgcaaaaaagggaataagggcgacacggaaatgttgaatactcatactcttcctttttca
atattattgaagcatttatcagggttattgtctcatgagcggatacatatttgaatgtatttagaaaaataaacaaataggggttccgcg
cacatttccccgaaaagtgccac**

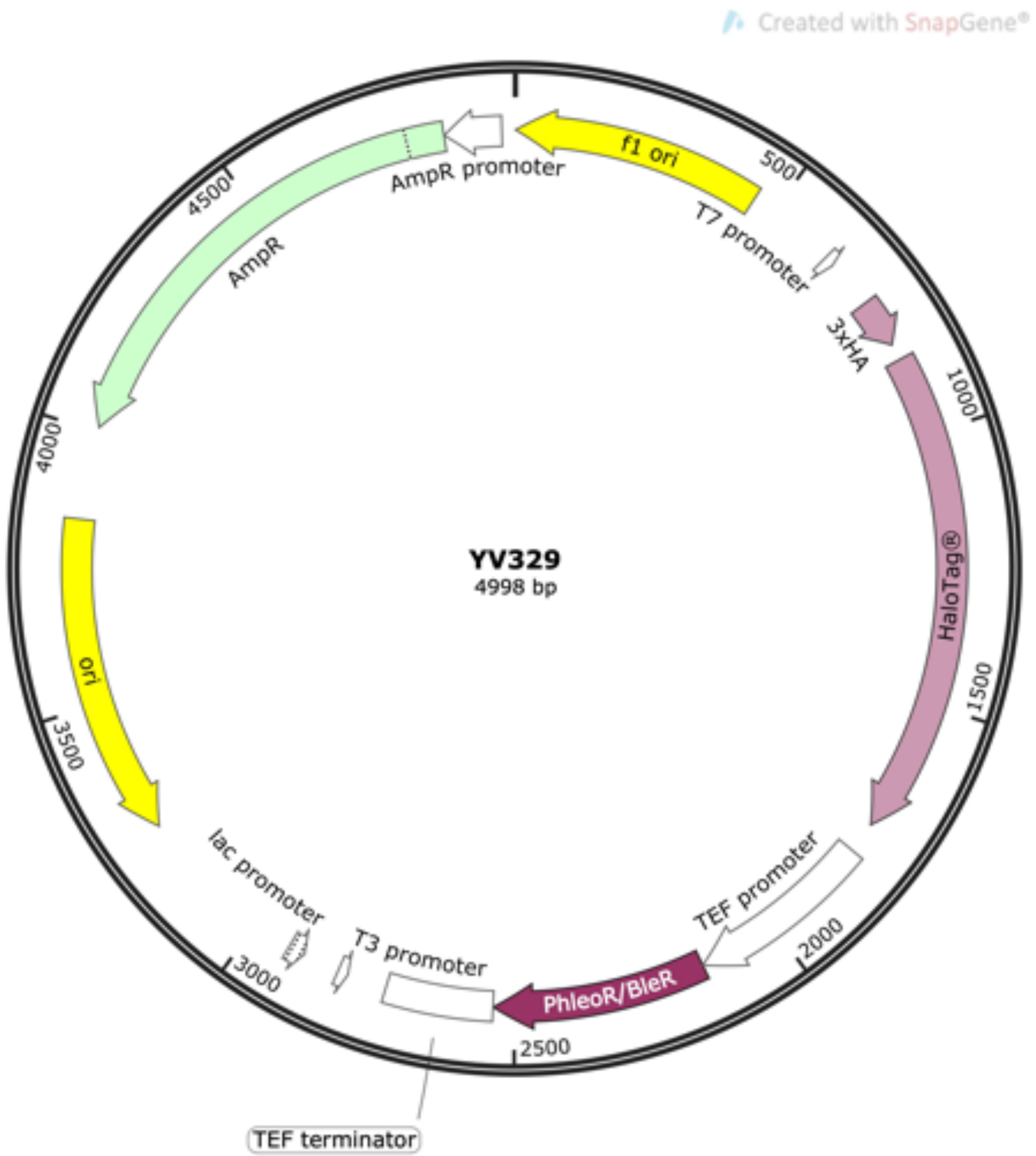

Created with SnapGene®
YV329
4998 bp
f1 ori
500
T7 promoter
3xHA
1000
HaloTag®
1500
TEF promoter
2000
PhleoR/BleR
2500
TEF terminator
T3 promoter
3000
lac promoter
3500
ori
4000
AmpR
4500
AmpR promoter

**YV329: pBS-SKII-3XHA-HALO-Phleomycin**

**ctaaattgtaagcgttaatattttgttaaaattcgcgttaaatttttgttaaatcagctcattttttaaccaataggccgaaatcggcaaa
atcccttataaatcaaaagaatagaccgagatagggttgagtgttgttccagtttggaacaagagtccactattaaagaacgtggact
ccaacgtcaaagggcgaaaaaccgtctatcagggcgatggcccactacgtgaaccatcaccctaatcaagttttttggggtcgaggtg
ccgtaaagcactaaatcggaaccctaaagggagcccccgatttagagcttgacggggaaagccggcgaacgtggcgagaaaggaag
ggaagaaagcgaaaggagcgggcgctagggcgctggcaagtgtagcggtcacgctgcgcgtaaccaccacacccgccgcgcttaatg
cgccgctacagggcgcgtcccattcgccattcaggctgcgcaactgttgggaagggcgatcggtgcgggcctcttcgctattacgccagc
tggcgaaagggggatgtgctgcaaggcgattaagttgggtaacgccagggttttcccagtcacgacgttgtaaaacgacggccagtga
gcgcgcgtaatacgactcactatagggcgaattgggtaccgggccccccctcgaggtcgacggtatcgataagcttgatatcgggtcga
cggatccccgggttaattaacatctttTACCCATACGATGTTCCTGACTATGCGGGCTATCCCTATGACGTCCCGG
ACTATGCAGGATCCTATCCATATGACGTTCCAGATTACGCTGCTCAGTGCggttcaggtggatctggttctATG
GCAGAAATCGGTACTGGCTTTCCATTCGACCCGCATTATGTGGAAGTCCTGGGCGAGAGAATGCATTA
CGTTGACGTGGGTCCGAGAGATGGAACTCCGGTCCTTTTTCTGCACGGGAATCCTACAAGCTCTTATGT
TTGGCGCAATATCATCCCTCATGTAGCTCCGACGCATCGCTGTATTGCGCCGGACCTGATTGGTATGGG
AAAATCTGATAAACCAGACCTGGGTTACTTTTTCGATGATCATGTGCGTTTCATGGATGCCTTCATTGA
GGCATTAGGGCTTGAAGAAGTCGTCTTGGTGATTCATGATTGGGGCTCAGCTCTGGGATTTCACTGGG
CTAAAAGAAATCCTGAACGCGTAAAAGGCATCGCGTTTATGGAGTTCATTCGTCCAATTCCGACTTGG
GATGAATGGCCTGAGTTCGCGAGAGAAACATTTCAAGCATTTCGCACGACCGATGTAGGCCGGAAGT
TAATCATCGATCAGAATGTCTTTATCGAAGGGACATTGCCGATGGGAGTCGTTCGTCCGTTAACAGAA
GTCGAAATGGATCACTATAGAGAACCTTTTCTTAATCCTGTGGACAGAGAGCCGCTGTGGCGGTTTCC
GAACGAACTGCCGATTGCAGGCGAGCCTGCTAACATTGTAGCGCTGGTTGAAGAGTATATGGATTGG
CTTCATCAGTCTCCAGTTCCGAAGTTATTGTTTTGGGGTACGCCTGGCGTGCTTATTCCACCGGCCGAA
GCGGCACGTTTGGCAAAAAGCCTGCCAAATTGCAAAGCCGTTGACATTGGCCCTGGACTTAACTTGCT
TCAAGAGGATAACCCGGACTTAATCGGGAGCGAAATTGCCCGGTGGCTTTCTACCTTAGAAATCAGCG
GCtaaTATATAACTGTCTAGAAATAAAGAGTATCATCTTTCAAAGGTCACCCGGCCAGCGACATGGAG
GCCCAGAATACCCTCCTTGACAGTCTTGACGTGCGCAGCTCAGGGGCATGATGTGACTGTCGCCCGTA
CATTTAGCCCATACATCCCCATGTATAATCATTTGCATCCATACATTTTGATGGCCGCACGGCGCGAAG
CAAAAATTACGGCTCCTCGCTGCAGACCTGCGAGCAGGGAAACGCTCCCCTCACAGACGCGTTGAATT
GTCCCCACGCCGCGCCCCTGTAGAGAAATATAAAAGGTTAGGATTTGCCACTGAGGTTCTTCTTTCATA
TACTTCCTTTTAAAATCTTGCTAGGATACAGTTCTCACATCACATCCGAACATAAACAACCATGGGTAT
GACCGACCAAGCGACGCCCAACCTGCCATCACGAGATTTCGATcCCACcGCCGCCTTCTATGAAAGGTT
GGGCTTCGGAATCGTTTTCCGGGACGCCGGCTGGATGATCCTCCAGCGCGGGGATCTCAtGCTGGAGT
TCTTCGCCCACCCCGGGCTCGATCCCCTCGCGAGTTGGTTCAGCTGCTGCCTGAGGCTGGACGACCTCG
CGGAGTTCTACCGGCAGTGCAAATCCGTCGGCATCCAGGAAACCAGCAGCGGCTATCCGCGCATCCAT
GCCCCCGAACTGCAGGAGTGGGGAGGCACGATGGCCGCTTTGGTCGACCCGGACGGGACGCTCCTGC
GCCTGATACAGAACGAATTGCTTGCAGGCATCTCATGATCAGTACTGACAATAAAAAGATTCTTGTTTT
CAAGAACTTGTCATTTGTATAGTTTTTTTATATTGTAGTTGTTCTATTTTAATCAAATGTTAGCGTGATTT
ATATTTTTTTTCGCCTCGACATCATCTGCCCAGATGCGAAGTTAAGTGCGCAGAAAGTAATATCATGCG
TCAATCGTATGTGAATGCTGGTCGCTATACTGCTGTCGATTCGATACTAACGCCGCcactagttctagagcgg
ccgccaccgcggtggagctccagcttttgttccctttagtgagggttaattgcgcgcttggcgtaatcatggtcatagctgtttcctgtgtg
aaattgttatccgctcacaattccacacaacatacgagccggaagcataaagtgtaaagcctggggtgcctaatgagtgagctaactc
acattaattgcgttgcgctcactgcccgctttccagtcgggaaacctgtcgtgccagctgcattaatgaatcggccaacgcgcggggag
aggcggtttgcgtattgggcgctcttccgcttcctcgctcactgactcgctgcgctcggtcgttcggctgcggcgagcggtatcagctcact**

caaaggcggtaatacggttatccacagaatcaggggataacgcaggaaagaacatgtgagcaaaaggccagcaaaaggccaggaa
ccgtaaaaaggccgcgttgctggcgtttttccataggctccgcccccctgacgagcatcacaaaaatcgacgctcaagtcagaggtggc
gaaacccgacaggactataaagataccaggcgtttccccctggaagctccctcgtgcgctctcctgttccgaccctgccgcttaccggat
acctgtccgcctttctcccttcgggaagcgtggcgctttctcatagctcacgctgtaggtatctcagttcggtgtaggtcgttcgctccaag
ctgggctgtgtgcacgaaccccccgttcagcccgaccgctgcgccttatccggtaactatcgtcttgagtccaacccggtaagacacgac
ttatcgccactggcagcagccactggtaacaggattagcagagcgaggtatgtaggcggtgctacagagttcttgaagtggtggccta
actacggctacactagaaggacagtatttggtatctgcgctctgctgaagccagttaccttcggaaaaagagttggtagctcttgatccg
gcaaacaaaccaccgctggtagcggtggtttttttgtttgcaagcagcagattacgcgcagaaaaaaaggatctcaagaagatccttt
gatcttttctacggggtctgacgctcagtggaacgaaaactcacgttaagggattttggtcatgagattatcaaaaaggatcttcacctа
gatccttttaaattaaaaatgaagttttaaatcaatctaaagtatatatgagtaaacttggtctgacagttaccaatgcttaatcagtga
ggcacctatctcagcgatctgtctatttcgttcatccatagttgcctgactccccgtcgtgtagataactacgatacgggagggcttacca
tctggccccagtgctgcaatgataccgcgagacccacgctcaccggctccagatttatcagcaataaaccagccagccggaagggccg
agcgcagaagtggtcctgcaactttatccgcctccatccagtctattaattgttgccgggaagctagagtaagtagttcgccagttaata
gtttgcgcaacgttgttgccattgctacaggcatcgtggtgtcacgctcgtcgtttggtatggcttcattcagctccggttcccaacgatca
aggcgagttacatgatcccccatgttgtgcaaaaaagcggttagctccttcggtcctccgatcgttgtcagaagtaagttggccgcagtg
ttatcactcatggttatggcagcactgcataattctcttactgtcatgccatccgtaagatgcttttctgtgactggtgagtactcaaccaa
gtcattctgagaatagtgtatgcggcgaccgagttgctcttgcccggcgtcaatacgggataataccgcgccacatagcagaactttaa
aagtgctcatcattggaaaacgttcttcggggcgaaaactctcaaggatcttaccgctgttgagatccagttcgatgtaacccactcgtg
cacccaactgatcttcagcatcttttactttcaccagcgtttctgggtgagcaaaaacaggaaggcaaaatgccgcaaaaaagggaat
aagggcgacacggaaatgttgaatactcatactcttcctttttcaatattattgaagcatttatcagggttattgtctcatgagcggatac
atatttgaatgtatttagaaaaataaacaaataggggttccgcgcacatttccccgaaaagtgccac

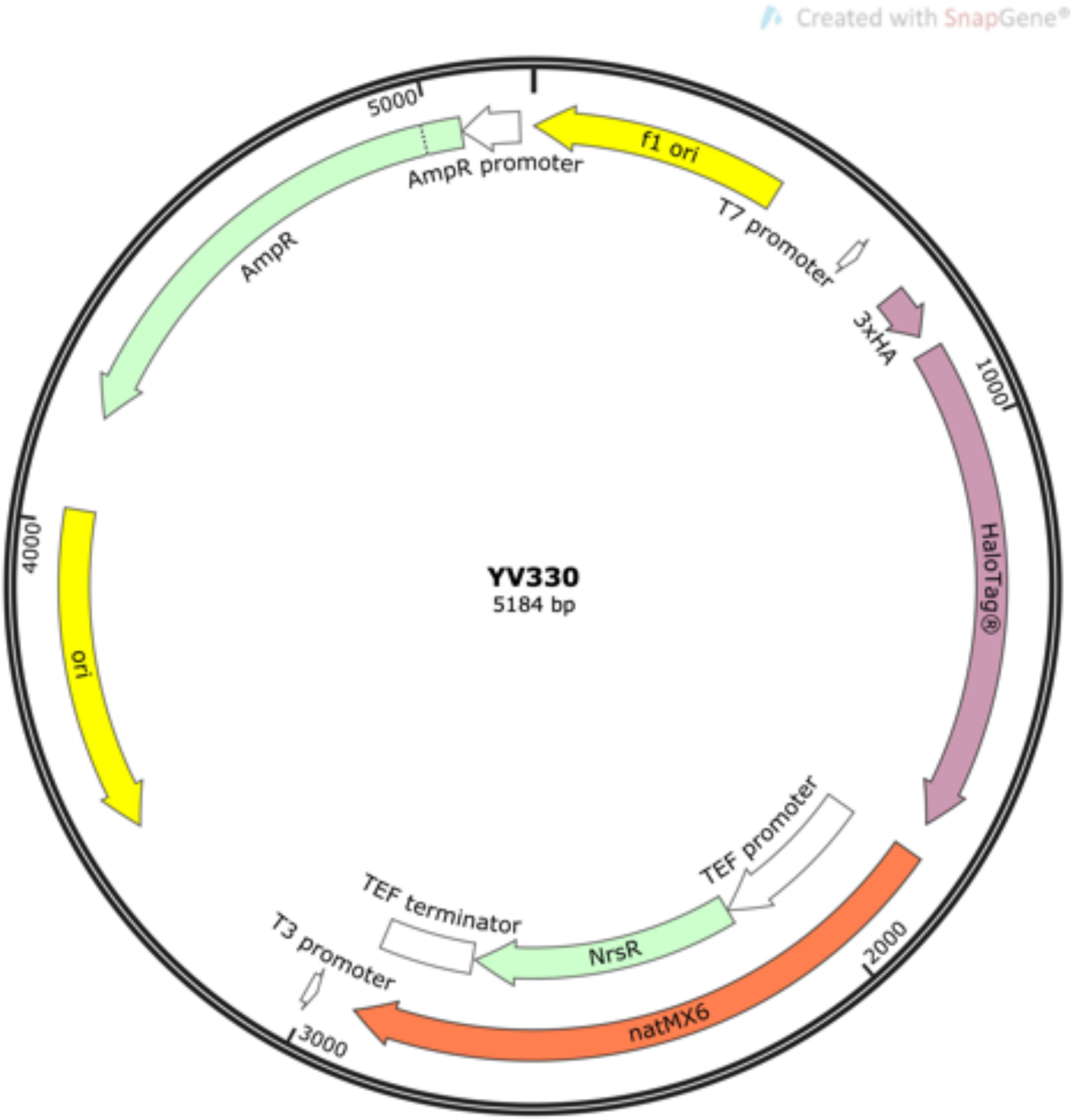
Created with SnapGene®
YV330
5184 bp
f1 ori
T7 promoter
3xHA
HaloTag®
TEF promoter
NrsR
TEF terminator
T3 promoter
natMX6
ori
AmpR
AmpR promoter
1000
2000
3000
4000
5000

**YV330: pBS-SKII-3XHA-HALO-NAT**

**ctaaattgtaagcgttaatattttgttaaaattcgcgttaaatttttgttaaatcagctcattttttaaccaataggccgaaatcggcaaa
atcccttataaatcaaaagaatagaccgagatagggttgagtgttgttccagtttggaacaagagtccactattaaagaacgtggact
ccaacgtcaaagggcgaaaaaccgtctatcagggcgatggcccactacgtgaaccatcaccctaatcaagttttttggggtcgaggtg
ccgtaaagcactaaatcggaaccctaaagggagcccccgatttagagcttgacggggaaagccggcgaacgtggcgagaaaggaag
ggaagaaagcgaaaggagcgggcgctagggcgctggcaagtgtagcggtcacgctgcgcgtaaccaccacacccgccgcgcttaatg
cgccgctacagggcgcgtcccattcgccattcaggctgcgcaactgttgggaagggcgatcggtgcgggcctcttcgctattacgccagc
tggcgaaagggggatgtgctgcaaggcgattaagttgggtaacgccagggttttcccagtcacgacgttgtaaaacgacggccagtga
gcgcgcgtaatacgactcactatagggcgaattgggtaccgggccccccctcgaggtcgacggtatcgataagcttgatatcgggtcga
cggatccccgggttaattaacatctttTACCCATACGATGTTCCTGACTATGCGGGCTATCCGTATGACGTCCCGG
ACTATGCAGGATCCTATCCATATGACGTTCCAGATTACGCTGCTCAGTGCggttcaggtggatctggttctATG
GCAGAAATCGGTACTGGCTTTCCATTCGACCCGCATTATGTGGAAGTCCTGGGCGAGAGAATGCATTA
CGTTGACGTGGGTCCGAGAGATGGAACTCCGGTCCTTTTTCTGCACGGGAATCCTACAAGCTCTTATGT
TTGGCGCAATATCATCCCTCATGTAGCTCCGACGCATCGCTGTATTGCGCCGGACCTGATTGGTATGGG
AAAATCTGATAAACCAGACCTGGGTTACTTTTTCGATGATCATGTGCGTTTCATGGATGCCTTCATTGA
GGCATTAGGGCTTGAAGAAGTCGTCTTGGTGATTCATGATTGGGGCTCAGCTCTGGGATTTCACTGGG
CTAAAAGAAATCCTGAACGCGTAAAAGGCATCGCGTTTATGGAGTTCATTCGTCCAATTCCGACTTGG
GATGAATGGCCTGAGTTCGCGAGAGAAACATTTCAAGCATTTCGCACGACCGATGTAGGCCGGAAGT
TAATCATCGATCAGAATGTCTTTATCGAAGGGACATTGCCGATGGGAGTCGTTCGTCCGTTAACAGAA
GTCGAAATGGATCACTATAGAGAACCTTTTCTTAATCCTGTGGACAGAGAGCCGCTGTGGCGGTTTCC
GAACGAACTGCCGATTGCAGGCGAGCCTGCTAACATTGTAGCGCTGGTTGAAGAGTATATGGATTGG
CTTCATCAGTCTCCAGTTCCGAAGTTATTGTTTTGGGGTACGCCTGGCGTGCTTATTCCACCGGCCGAA
GCGGCACGTTTGGCAAAAAGCCTGCCAAATTGCAAAGCCGTTGACATTGGCCCTGGACTTAACTTGCT
TCAAGAGGATAACCCGGACTTAATCGGGAGCGAAATTGCCCGGTGGCTTTCTACCTTAGAAATCAGCG
GCtaaTATATAACTGTCTAGAAATAAAGAGTATCATCTTTCAAAGGTCACCCGGCCAGCGACATGGAG
GCCCAGAATACCCTCCTTGACAGTCTTGACGTGCGCAGCTCAGGGGCATGATGTGACTGTCGCCCGTA
CATTTAGCCCATACATCCCCATGTATAATCATTTGCATCCATACATTTTGATGGCCGCACGGCGCGAAG
CAAAAATTACGGCTCCTCGCTGCAGACCTGCGAGCAGGGAAACGCTCCCCTCACAGACGCGTTGAATT
GTCCCCACGCCGCGCCCCTGTAGAGAAATATAAAAGGTTAGGATTTGCCACTGAGGTTCTTCTTTCATA
TACTTCCTTTTAAAATCTTGCTAGGATACAGTTCTCACATCACATCCGAACATAAACAACCATGGGTACC
ACTCTTGACGACACGGCTTACCGGTACCGCACCAGTGTCCCGGGGGACGCCGAGGCCATCGAGGCACT
GGATGGGTCCTTCACCACCGACACCGTCTTCCGCGTCACCGCCACCGGGGACGGCTTCACCCTGCGGG
AGGTGCCGGTGGACCCGCCCCTGACCAAGGTGTTCCCCGACGACGAATCGGACGACGAATCGGACGA
CGGGGAGGACGGCGACCCGGACTCCCGGACGTTCGTCGCGTACGGGGACGACGGCGACCTGGCGGG
CTTCGTGGTCGTCTCGTACTCCGGCTGGAACCGCCGGCTGACCGTCGAGGACATCGAGGTCGCCCCGG
AGCACCGGGGGCACGGGGTCGGGCGCGCGTTGATGGGGCTCGCGACGGAGTTCGCCCGCGAGCGGG
GCGCCGGGCACCTCTGGCTGGAGGTCACCAACGTCAACGCACCGGCGATCCACGCGTACCGGCGGAT
GGGGTTCACCCTCTGCGGCCTGGACACCGCCCTGTACGACGGCACCGCCTCGGACGGCGAGCAGGCGC
TCTACATGAGCATGCCCTGCCCCTAATCAGTACTGACAATAAAAAGATTCTTGTTTTCAAGAACTTGTC
ATTTGTATAGTTTTTTTATATTGTAGTTGTTCTATTTTAATCAAATGTTAGCGTGATTTATATTTTTTTTCG
CCTCGACATCATCTGCCCAGATGCGAAGTTAAGTGCGCAGAAAGTAATATCATGCGTCAATCGTATGT
GAATGCTGGTCGCTATACTGCTGTCGATTCGATACTAACGCCGCcactagttctagagcggccgccaccgcggtgg
agctccagcttttgttccctttagtgagggttaattgcgcgcttggcgtaatcatggtcatagctgtttcctgtgtgaaattgttatccgctc**

acaattccacacaacatacgagccggaagcataaagtgtaaagcctggggtgcctaatgagtgagctaactcacattaattgcgttgc
gctcactgcccgctttccagtcgggaaacctgtcgtgccagctgcattaatgaatcggccaacgcgcggggagaggcggtttgcgtatt
gggcgctcttccgcttcctcgctcactgactcgctgcgctcggtcgttcggctgcggcgagcggtatcagctcactcaaaggcggtaatac
ggttatccacagaatcaggggataacgcaggaaagaacatgtgagcaaaaggccagcaaaaggccaggaaccgtaaaaaggccgc
gttgctggcgtttttccataggctccgcccccctgacgagcatcacaaaaatcgacgctcaagtcagaggtggcgaaacccgacagga
ctataaagataccaggcgtttccccctggaagctccctcgtgcgctctcctgttccgaccctgccgcttaccggatacctgtccgcctttct
cccttcgggaagcgtggcgctttctcatagctcacgctgtaggtatctcagttcggtgtaggtcgttcgctccaagctgggctgtgtgcac
gaaccccccgttcagcccgaccgctgcgccttatccggtaactatcgtcttgagtccaacccggtaagacacgacttatcgccactggca
gcagccactggtaacaggattagcagagcgaggtatgtaggcggtgctacagagttcttgaagtggtggcctaactacggctacacta
gaaggacagtatttggtatctgcgctctgctgaagccagttaccttcggaaaaagagttggtagctcttgatccggcaaacaaaccacc
gctggtagcggtggtttttttgtttgcaagcagcagattacgcgcagaaaaaaaggatctcaagaagatcctttgatcttttctacgggg
tctgacgctcagtggaacgaaaactcacgttaagggattttggtcatgagattatcaaaaaggatcttcacctagatccttttaaattaa
aaatgaagttttaaatcaatctaaagtatatatgagtaaacttggtctgacagttaccaatgcttaatcagtgaggcacctatctcagc
gatctgtctatttcgttcatccatagttgcctgactccccgtcgtgtagataactacgatacgggagggcttaccatctggccccagtgct
gcaatgataccgcgagacccacgctcaccggctccagatttatcagcaataaaccagccagccggaagggccgagcgcagaagtggt
cctgcaactttatccgcctccatccagtctattaattgttgccgggaagctagagtaagtagttcgccagttaatagtttgcgcaacgttg
ttgccattgctacaggcatcgtggtgtcacgctcgtcgtttggtatggcttcattcagctccggttcccaacgatcaaggcgagttacatg
atcccccatgttgtgcaaaaaagcggttagctccttcggtcctccgatcgttgtcagaagtaagttggccgcagtgttatcactcatggtt
atggcagcactgcataattctcttactgtcatgccatccgtaagatgcttttctgtgactggtgagtactcaaccaagtcattctgagaat
agtgtatgcggcgaccgagttgctcttgcccggcgtcaatacgggataataccgcgccacatagcagaactttaaaagtgctcatcatt
ggaaaacgttcttcggggcgaaaactctcaaggatcttaccgctgttgagatccagttcgatgtaacccactcgtgcacccaactgatct
tcagcatcttttactttcaccagcgtttctgggtgagcaaaaacaggaaggcaaaatgccgcaaaaaagggaataagggcgacacgg
aaatgttgaatactcatactcttcctttttcaatattattgaagcatttatcagggttattgtctcatgagcggatacatatttgaatgtatt
tagaaaaataaacaaataggggttccgcgcacatttccccgaaaagtgccac

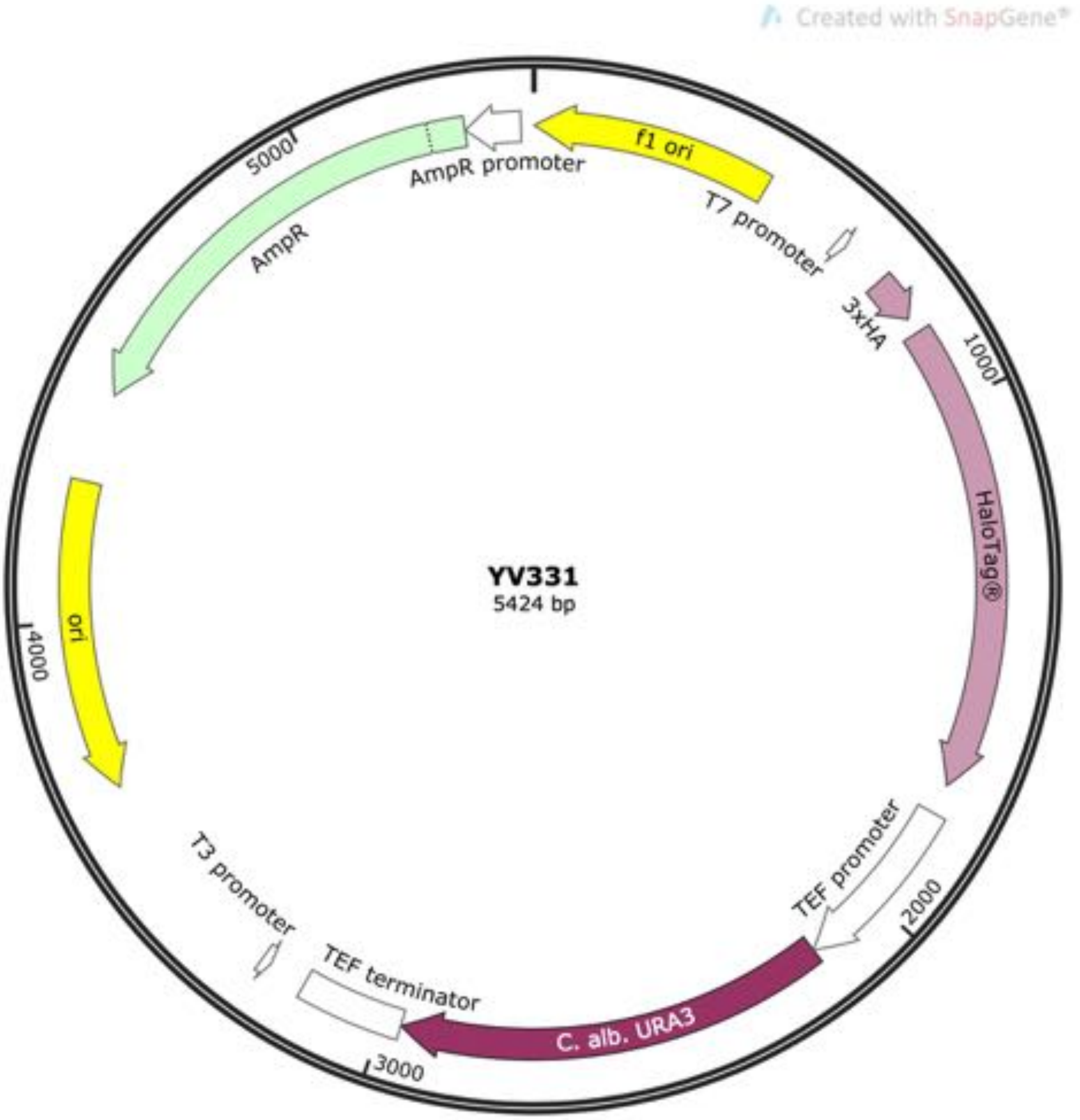
Created with SnapGene®
YV331
5424 bp
f1 ori
AmpR promoter
AmpR
T7 promoter
3xHA
HaloTag®
TEF promoter
C. alb. URA3
TEF terminator
T3 promoter
ori
1000
2000
3000
4000
5000

**YV331: pBS-SKII-3XHA-HALO-URA**

**ctaaattgtaagcgttaatattttgttaaaattcgcgttaaatttttgttaaatcagctcattttttaaccaataggccgaaatcggcaaa
atcccttataaatcaaaagaatagaccgagatagggttgagtgttgttccagtttggaacaagagtccactattaaagaacgtggact
ccaacgtcaaagggcgaaaaaccgtctatcagggcgatggcccactacgtgaaccatcaccctaatcaagttttttggggtcgaggtg
ccgtaaagcactaaatcggaaccctaaagggagcccccgatttagagcttgacggggaaagccggcgaacgtggcgagaaaggaag
ggaagaaagcgaaaggagcgggcgctagggcgctggcaagtgtagcggtcacgctgcgcgtaaccaccacacccgccgcgcttaatg
cgccgctacagggcgcgtcccattcgccattcaggctgcgcaactgttgggaagggcgatcggtgcgggcctcttcgctattacgccagc
tggcgaaagggggatgtgctgcaaggcgattaagttgggtaacgccagggttttcccagtcacgacgttgtaaaacgacggccagtga
gcgcgcgtaatacgactcactatagggcgaattgggtaccgggccccccctcgaggtcgacggtatcgataagcttgatatcgggtcga
cggatccccgggttaattaacatctttTACCCATACGATGTTCCTGACTATGCGGGCTATCCCTATGACGTCCCGG
ACTATGCAGGATCCTATCCATATGACGTTCCAGATTACGCTGCTCAGTGCggttcaggtggatctggttctATG
GCAGAAATCGGTACTGGCTTTCCATTCGACCCGCATTATGTGGAAGTCCTGGGCGAGAGAATGCATTA
CGTTGACGTGGGTCCGAGAGATGGAACTCCGGTCCTTTTTCTGCACGGGAATCCTACAAGCTCTTATGT
TTGGCGCAATATCATCCCTCATGTAGCTCCGACGCATCGCTGTATTGCGCCGGACCTGATTGGTATGGG
AAAATCTGATAAACCAGACCTGGGTTACTTTTTCGATGATCATGTGCGTTTCATGGATGCCTTCATTGA
GGCATTAGGGCTTGAAGAAGTCGTCTTGGTGATTCATGATTGGGGCTCAGCTCTGGGATTTCACTGGG
CTAAAAGAAATCCTGAACGCGTAAAAGGCATCGCGTTTATGGAGTTCATTCGTCCAATTCCGACTTGG
GATGAATGGCCTGAGTTCGCGAGAGAAACATTTCAAGCATTTCGCACGACCGATGTAGGCCGGAAGT
TAATCATCGATCAGAATGTCTTTATCGAAGGGACATTGCCGATGGGAGTCGTTCGTCCGTTAACAGAA
GTCGAAATGGATCACTATAGAGAACCTTTTCTTAATCCTGTGGACAGAGAGCCGCTGTGGCGGTTTCC
GAACGAACTGCCGATTGCAGGCGAGCCTGCTAACATTGTAGCGCTGGTTGAAGAGTATATGGATTGG
CTTCATCAGTCTCCAGTTCCGAAGTTATTGTTTTGGGGTACGCCTGGCGTGCTTATTCCACCGGCCGAA
GCGGCACGTTTGGCAAAAAGCCTGCCAAATTGCAAAGCCGTTGACATTGGCCCTGGACTTAACTTGCT
TCAAGAGGATAACCCGGACTTAATCGGGAGCGAAATTGCCCGGTGGCTTTCTACCTTAGAAATCAGCG
GCtaaTATATAACTGTCTAGAAATAAAGAGTATCATCTTTCAAAGGTCACCCGGCCAGCGACATGGAG
GCCCAGAATACCCTCCTTGACAGTCTTGACGTGCGCAGCTCAGGGGCATGATGTGACTGTCGCCCGTA
CATTTAGCCCATACATCCCCATGTATAATCATTTGCATCCATACATTTTGATGGCCGCACGGCGCGAAG
CAAAAATTACGGCTCCTCGCTGCAGACCTGCGAGCAGGGAAACGCTCCCCTCACAGACGCGTTGAATT
GTCCCCACGCCGCGCCCCTGTAGAGAAATATAAAAGGTTAGGATTTGCCACTGAGGTTCTTCTTTCATA
TACTTCCTTTTAAAATCTTGTTAGGATACAGTTCTCACATCACATCCGAACATAAACAACCATGACAGTC
AACACTAAGACCTATAGTGAGAGAGCAGAAACTCATGCCTCACCAGTAGCACAACGATTATTTCGATT
AATGGAACTGAAGAAAACCAATTTATGTGCATCAATTGATGTTGATACCACTAAGGAATTCCTTGAAT
TAATTGATAAATTGGGTCCTTATGTATGCTTAATCAAGACTCATATTGATATAATCAATGATTTTTCCTA
TGAATCCACTATTGAACCATTATTAGAACTTTCACGTAAACATCAATTTATGATTTTTGAAGATAGAAA
ATTTGCTGATATTGGTAATACCGTGAAGAAACAATATATTGGTGGAGTTTATAAAATTAGTAGTTGGG
CAGATATTACTAATGCTCATGGTGTCACTGGGAATGGAGTAGTTGAAGGATTAAAACAGGGAGCTAA
AGAAACCACCACCAACCAAGAGCCAAGAGGGTTATTGATGTTAGCTGAATTATCATCAGTGGGATCAT
TAGCATATGGAGAATATTCTCAAAAAACTGTTGAAATTGCTAAATCCGATAAGGAATTTGTTATTGGA
TTTATTGCCCAACGTGATATGGGTGGACAAGAAGAAGGATTTGATTGGCTTATTATGACACCTGGAGT
TGGATTAGATGATAAAGGTGATGGATTAGGACAACAATATAGAACTGTTGATGAAGTTGTTAGCACT
GGAACTGATATTATCATTGTTGGTAGAGGATTGTTTGGTAAAGGAAGAGATCCAGATATTGAAGGTA
AAAGGTATAGAGATGCTGGTTGGAATGCTTATTTGAAAAAGACTGGCCAATTATAATCAGTACTGACA
ATAAAAAGATTCTTGTTTTCAAGAACTTGTCATTTGTATAGTTTTTTTATATTGTAGTTGTTCTATTTTAA**

**TCAAATGTTAGCGTGATTTATATTTTTTTTCGCCTCGACATCATCTGCCCAGATGCGAAGTTAAGTGCGC
AGAAAGTAATATCATGCGTCAATCGTATGTGAATGCTGGTCGCTATACTGCTGTCGATTCGATACTAAC
GCCGCcactagttctagagcggccgccaccgcggtggagctccagcttttgttccctttagtgagggttaattgcgcgcttggcgtaat
catggtcatagctgtttcctgtgtgaaattgttatccgctcacaattccacacaacatacgagccggaagcataaagtgtaaagcctgg
ggtgcctaatgagtgagctaactcacattaattgcgttgcgctcactgcccgctttccagtcgggaaacctgtcgtgccagctgcattaat
gaatcggccaacgcgcggggagaggcggtttgcgtattgggcgctcttccgcttcctcgctcactgactcgctgcgctcggtcgttcggct
gcggcgagcggtatcagctcactcaaaggcggtaatacggttatccacagaatcaggggataacgcaggaaagaacatgtgagcaa
aaggccagcaaaaggccaggaaccgtaaaaaggccgcgttgctggcgtttttccataggctccgcccccctgacgagcatcacaaaa
atcgacgctcaagtcagaggtggcgaaacccgacaggactataaagataccaggcgtttccccctggaagctccctcgtgcgctctcct
gttccgaccctgccgcttaccggatacctgtccgcctttctcccttcgggaagcgtggcgctttctcatagctcacgctgtaggtatctcag
ttcggtgtaggtcgttcgctccaagctgggctgtgtgcacgaaccccccgttcagcccgaccgctgcgccttatccggtaactatcgtctt
gagtccaacccggtaagacacgacttatcgccactggcagcagccactggtaacaggattagcagagcgaggtatgtaggcggtgct
acagagttcttgaagtggtggcctaactacggctacactagaaggacagtatttggtatctgcgctctgctgaagccagttaccttcgga
aaaagagttggtagctcttgatccggcaaacaaaccaccgctggtagcggtggtttttttgtttgcaagcagcagattacgcgcagaaa
aaaaggatctcaagaagatcctttgatcttttctacggggtctgacgctcagtggaacgaaaactcacgttaagggattttggtcatga
gattatcaaaaaggatcttcacctagatccttttaaattaaaaatgaagttttaaatcaatctaaagtatatatgagtaaacttggtctg
acagttaccaatgcttaatcagtgaggcacctatctcagcgatctgtctatttcgttcatccatagttgcctgactccccgtcgtgtagata
actacgatacgggagggcttaccatctggccccagtgctgcaatgataccgcgagacccacgctcaccggctccagatttatcagcaat
aaaccagccagccggaagggccgagcgcagaagtggtcctgcaactttatccgcctccatccagtctattaattgttgccgggaagcta
gagtaagtagttcgccagttaatagtttgcgcaacgttgttgccattgctacaggcatcgtggtgtcacgctcgtcgtttggtatggcttc
attcagctccggttcccaacgatcaaggcgagttacatgatcccccatgttgtgcaaaaaagcggttagctccttcggtcctccgatcgtt
gtcagaagtaagttggccgcagtgttatcactcatggttatggcagcactgcataattctcttactgtcatgccatccgtaagatgctttt
ctgtgactggtgagtactcaaccaagtcattctgagaatagtgtatgcggcgaccgagttgctcttgcccggcgtcaatacgggataat
accgcgccacatagcagaactttaaaagtgctcatcattggaaaacgttcttcggggcgaaaactctcaaggatcttaccgctgttgag
atccagttcgatgtaacccactcgtgcacccaactgatcttcagcatcttttactttcaccagcgtttctgggtgagcaaaaacaggaag
gcaaaatgccgcaaaaaagggaataagggcgacacggaaatgttgaatactcatactcttcctttttcaatattattgaagcatttatc
agggttattgtctcatgagcggatacatatttgaatgtatttagaaaaataaacaaataggggttccgcgcacatttccccgaaaagtg
ccac**

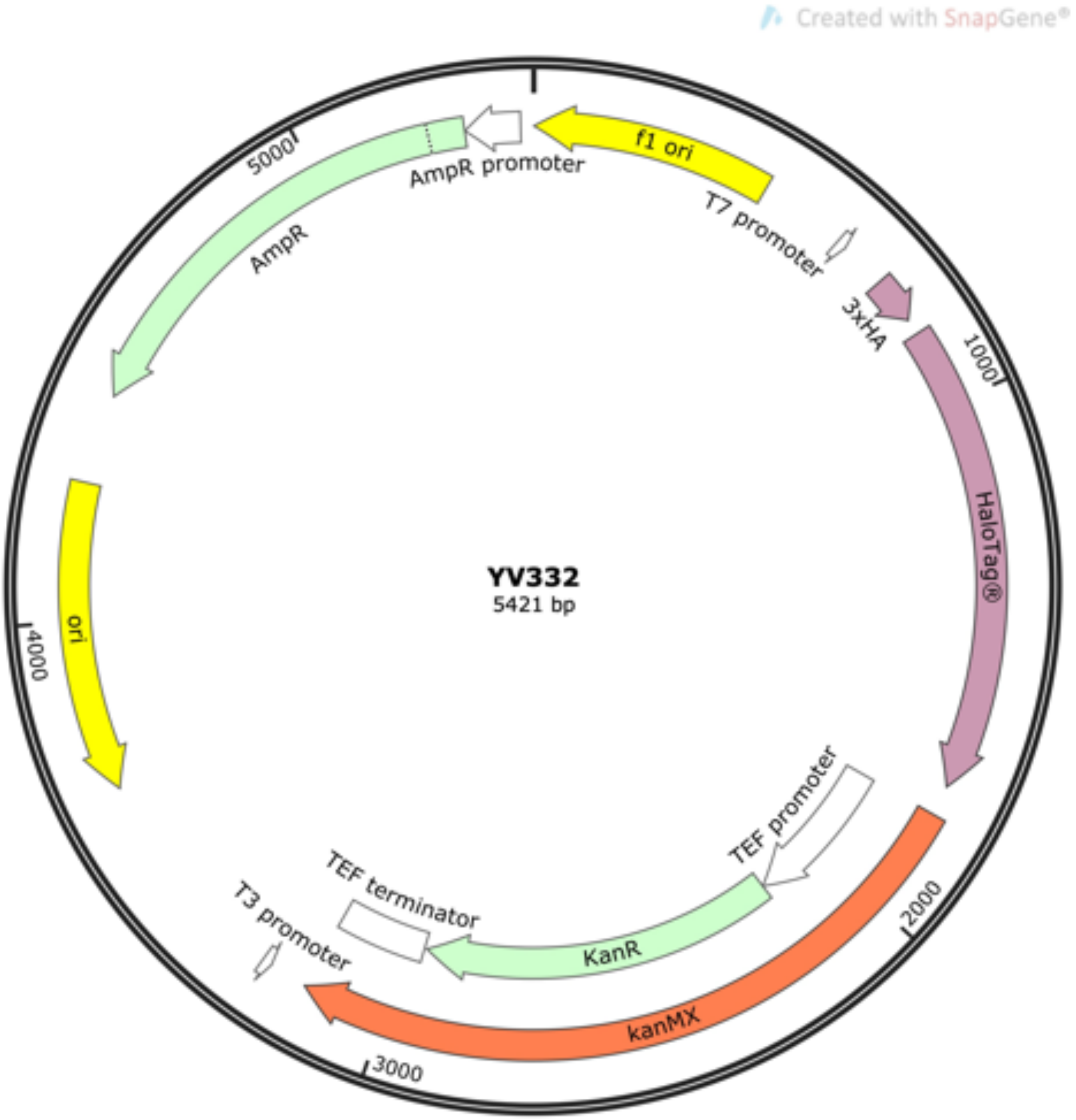
Created with SnapGene®
YV332
5421 bp
f1 ori
T7 promoter
3xHA
1000
HaloTag®
TEF promoter
2000
kanMX
KanR
TEF terminator
T3 promoter
3000
ori
4000
AmpR
5000
AmpR promoter

**YV332: pBS-SKII-3XHA-HALO-Kan**

**ctaaattgtaagcgttaatattttgttaaaattcgcgttaaatttttgttaaatcagctcattttttaaccaataggccgaaatcggcaaa
atcccttataaatcaaaagaatagaccgagatagggttgagtgttgttccagtttggaacaagagtccactattaaagaacgtggact
ccaacgtcaaagggcgaaaaaccgtctatcagggcgatggcccactacgtgaaccatcaccctaatcaagttttttggggtcgaggtg
ccgtaaagcactaaatcggaaccctaaagggagcccccgatttagagcttgacggggaaagccggcgaacgtggcgagaaaggaag
ggaagaaagcgaaaggagcgggcgctagggcgctggcaagtgtagcggtcacgctgcgcgtaaccaccacacccgccgcgcttaatg
cgccgctacagggcgcgtcccattcgccattcaggctgcgcaactgttgggaagggcgatcggtgcgggcctcttcgctattacgccagc
tggcgaaagggggatgtgctgcaaggcgattaagttgggtaacgccagggttttcccagtcacgacgttgtaaaacgacggccagtga
gcgcgcgtaatacgactcactatagggcgaattgggtaccgggccccccctcgaggtcgacggtatcgataagcttgatatcgggtcga
cggatccccgggttaattaacatctttTACCCATACGATGTTCCTGACTATGCGGGCTATCCGTATGACGTCCCGG
ACTATGCAGGATCCTATCCATATGACGTTCCAGATTACGCTGCTCAGTGCggttcaggtggatctggttctATG
GCAGAAATCGGTACTGGCTTTCCATTCGACCCGCATTATGTGGAAGTCCTGGGCGAGAGAATGCATTA
CGTTGACGTGGGTCCGAGAGATGGAACTCCGGTCCTTTTTCTGCACGGGAATCCTACAAGCTCTTATGT
TTGGCGCAATATCATCCCTCATGTAGCTCCGACGCATCGCTGTATTGCGCCGGACCTGATTGGTATGGG
AAAATCTGATAAACCAGACCTGGGTTACTTTTTCGATGATCATGTGCGTTTCATGGATGCCTTCATTGA
GGCATTAGGGCTTGAAGAAGTCGTCTTGGTGATTCATGATTGGGGCTCAGCTCTGGGATTTCACTGGG
CTAAAAGAAATCCTGAACGCGTAAAAGGCATCGCGTTTATGGAGTTCATTCGTCCAATTCCGACTTGG
GATGAATGGCCTGAGTTCGCGAGAGAAACATTTCAAGCATTTCGCACGACCGATGTAGGCCGGAAGT
TAATCATCGATCAGAATGTCTTTATCGAAGGGACATTGCCGATGGGAGTCGTTCGTCCGTTAACAGAA
GTCGAAATGGATCACTATAGAGAACCTTTTCTTAATCCTGTGGACAGAGAGCCGCTGTGGCGGTTTCC
GAACGAACTGCCGATTGCAGGCGAGCCTGCTAACATTGTAGCGCTGGTTGAAGAGTATATGGATTGG
CTTCATCAGTCTCCAGTTCCGAAGTTATTGTTTTGGGGTACGCCTGGCGTGCTTATTCCACCGGCCGAA
GCGGCACGTTTGGCAAAAAGCCTGCCAAATTGCAAAGCCGTTGACATTGGCCCTGGACTTAACTTGCT
TCAAGAGGATAACCCGGACTTAATCGGGAGCGAAATTGCCCGGTGGCTTTCTACCTTAGAAATCAGCG
GCtaaTATATAACTGTCTAGAAATAAAGAGTATCATCTTTCAAAGGTCACCCGGCCAGCGACATGGAG
GCCCAGAATACCCTCCTTGACAGTCTTGACGTGCGCAGCTCAGGGGCATGATGTGACTGTCGCCCGTA
CATTTAGCCCATACATCCCCATGTATAATCATTTGCATCCATACATTTTGATGGCCGCACGGCGCGAAG
CAAAAATTACGGCTCCTCGCTGCAGACCTGCGAGCAGGGAAACGCTCCCCTCACAGACGCGTTGAATT
GTCCCCACGCCGCGCCCCTGTAGAGAAATATAAAAGGTTAGGATTTGCCACTGAGGTTCTTCTTTCATA
TACTTCCTTTTAAAATCTTGCTAGGATACAGTTCTCACATCACATCCGAACATAAACAACCATGGGTAA
GGAAAAGACTCACGTTTCGAGGCCGCGATTAAATTCCAACATGGATGCTGATTTATATGGGTATAAAT
GGGCTCGCGATAATGTCGGGCAATCAGGTGCGACAATCTATCGATTGTATGGGAAGCCCGATGCGCC
AGAGTTGTTTCTGAAACATGGCAAAGGTAGCGTTGCCAATGATGTTACAGATGAGATGGTCAGACTA
AACTGGCTGACGGAATTTATGCCTCTTCCGACCATCAAGCATTTTATCCGTACTCCTGATGATGCATGG
TTACTCACCACTGCGATCCCCGGCAAAACAGCATTCCAGGTATTAGAAGAATATCCTGATTCAGGTGA
AAATATTGTTGATGCGCTGGCAGTGTTCCTGCGCCGGTTGCATTCGATTCCTGTTTGTAATTGTCCTTTT
AACAGCGATCGCGTATTTCGTCTCGCTCAGGCGCAATCACGAATGAATAACGGTTTGGTTGATGCGAG
TGATTTTGATGACGAGCGTAATGGCTGGCCTGTTGAACAAGTCTGGAAAGAAATGCATAAGCTTTTGC
CATTCTCACCGGATTCAGTCGTCACTCATGGTGATTTCTCACTTGATAACCTTATTTTTGACGAGGGGAA
ATTAATAGGTTGTATTGATGTTGGACGAGTCGGAATCGCAGACCGATACCAGGATCTTGCCATCCTAT
GGAACTGCCTCGGTGAGTTTTCTCCTTCATTACAGAAACGGCTTTTTCAAAAATATGGTATTGATAATC
CTGATATGAATAAATTGCAGTTTCATTTGATGCTCGATGAGTTTTTCTAATCAGTACTGACAATAAAAA
GATTCTTGTTTTCAAGAACTTGTCATTTGTATAGTTTTTTATATTGTAGTTGTTCTATTTTAATCAAATG**

**TTAGCGTGATTTATATTTTTTTTCGCCTCGACATCATCTGCCCAGATGCGAAGTTAAGTGCGCAGAAAG**
**TAATATCATGCGTCAATCGTATGTGAATGCTGGTCGCTATACTGCTGTCGATTCGATACTAACGCCGCc**
**actagttctagagcggccgccaccgcggtggagctccagcttttgttccctttagtgagggttaattgcgcgcttggcgtaatcatggtca**
**tagctgtttcctgtgtgaaattgttatccgctcacaattccacacaacatacgagccggaagcataaagtgtaaagcctggggtgccta**
**atgagtgagctaactcacattaattgcgttgcgctcactgcccgctttccagtcgggaaacctgtcgtgccagctgcattaatgaatcgg**
**ccaacgcgcggggagaggcggtttgcgtattgggcgctcttccgcttcctcgctcactgactcgctgcgctcggtcgttcggctgcggcga**
**gcggtatcagctcactcaaaggcggtaatacggttatccacagaatcaggggataacgcaggaaagaacatgtgagcaaaaggcca**
**gcaaaaggccaggaaccgtaaaaaggccgcgttgctggcgtttttccataggctccgcccccctgacgagcatcacaaaaatcgacgc**
**tcaagtcagaggtggcgaaacccgacaggactataaagataccaggcgtttccccctggaagctccctcgtgcgctctcctgttccgac**
**cctgccgcttaccggatacctgtccgcctttctcccttcgggaagcgtggcgctttctcatagctcacgctgtaggtatctcagttcggtgt**
**aggtcgttcgctccaagctgggctgtgtgcacgaaccccccgttcagcccgaccgctgcgccttatccggtaactatcgtcttgagtccaa**
**cccggtaagacacgacttatcgccactggcagcagccactggtaacaggattagcagagcgaggtatgtaggcggtgctacagagttc**
**ttgaagtggtggcctaactacggctacactagaaggacagtatttggtatctgcgctctgctgaagccagttaccttcggaaaaagagt**
**tggtagctcttgatccggcaaacaaaccaccgctggtagcggtggtttttttgtttgcaagcagcagattacgcgcagaaaaaaaggat**
**ctcaagaagatcctttgatcttttctacggggtctgacgctcagtggaacgaaaactcacgttaagggattttggtcatgagattatcaa**
**aaaggatcttcacctagatccttttaaattaaaaatgaagttttaaatcaatctaaagtatatatgagtaaacttggtctgacagttacc**
**aatgcttaatcagtgaggcacctatctcagcgatctgtctatttcgttcatccatagttgcctgactccccgtcgtgtagataactacgat**
**acgggagggcttaccatctggccccagtgctgcaatgataccgcgagacccacgctcaccggctccagatttatcagcaataaaccag**
**ccagccggaagggccgagcgcagaagtggtcctgcaactttatccgcctccatccagtctattaattgttgccgggaagctagagtaag**
**tagttcgccagttaatagtttgcgcaacgttgttgccattgctacaggcatcgtggtgtcacgctcgtcgtttggtatggcttcattcagct**
**ccggttcccaacgatcaaggcgagttacatgatcccccatgttgtgcaaaaaagcggttagctccttcggtcctccgatcgttgtcagaa**
**gtaagttggccgcagtgttatcactcatggttatggcagcactgcataattctcttactgtcatgccatccgtaagatgcttttctgtgact**
**ggtgagtactcaaccaagtcattctgagaatagtgtatgcggcgaccgagttgctcttgcccggcgtcaatacgggataataccgcgcc**
**acatagcagaactttaaaagtgctcatcattggaaaacgttcttcggggcgaaaactctcaaggatcttaccgctgttgagatccagtt**
**cgatgtaacccactcgtgcacccaactgatcttcagcatcttttactttcaccagcgtttctgggtgagcaaaaacaggaaggcaaaat**
**gccgcaaaaaagggaataagggcgacacggaaatgttgaatactcatactcttcctttttcaatattattgaagcatttatcagggttat**
**tgtctcatgagcggatacatatttgaatgtatttagaaaaataaacaaataggggttccgcgcacatttccccgaaaagtgccac**

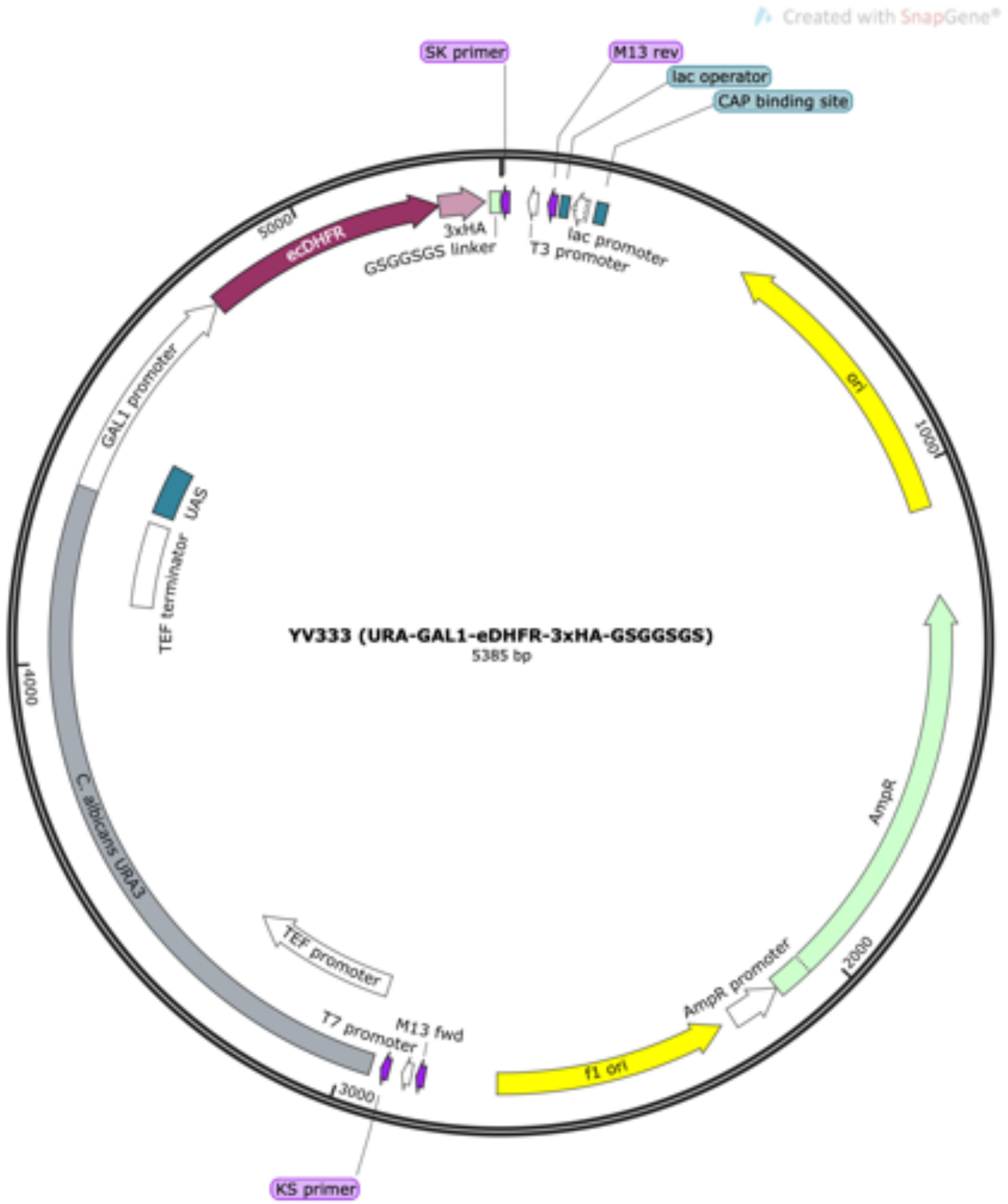
Created with SnapGene®
SK primer
M13 rev
lac operator
CAP binding site
3xHA
GSGGSGS linker
T3 promoter
lac promoter
5000
ecDHFR
GAL1 promoter
UAS
TEF terminator
ori
1000
YV333 (URA-GAL1-eDHFR-3xHA-GSGGSGS)
5385 bp
4000
C. albicans URA3
AmpR
TEF promoter
AmpR promoter
2000
T7 promoter
M13 fwd
f1 ori
3000
KS primer

**Supplemental Data:** Maps and sequences of tagging plasmids

## YV333: pBS-SKII-URA-GAL1-DHFR-3xHA

**gatccactagttctagagcggccgccaccgcggtggagctccagcttttgttccctttagtgagggttaattgcgcgcttggcgtaatcat
ggtcatagctgtttcctgtgtgaaattgttatccgctcacaattccacacaacatacgagccggaagcataaagtgtaaagcctggggt
gcctaatgagtgagctaactcacattaattgcgttgcgctcactgcccgctttccagtcgggaaacctgtcgtgccagctgcattaatga
atcggccaacgcgcggggagaggcggtttgcgtattgggcgctcttccgcttcctcgctcactgactcgctgcgctcggtcgttcggctgc
ggcgagcggtatcagctcactcaaaggcggtaatacggttatccacagaatcaggggataacgcaggaaagaacatgtgagcaaaa
ggccagcaaaaggccaggaaccgtaaaaaggccgcgttgctggcgtttttccataggctccgcccccctgacgagcatcacaaaaatc
gacgctcaagtcagaggtggcgaaacccgacaggactataaagataccaggcgtttccccctggaagctccctcgtgcgctctcctgtt
ccgaccctgccgcttaccggatacctgtccgcctttctcccttcgggaagcgtggcgctttctcatagctcacgctgtaggtatctcagttc
ggtgtaggtcgttcgctccaagctgggctgtgtgcacgaaccccccgttcagcccgaccgctgcgccttatccggtaactatcgtcttgag
tccaacccggtaagacacgacttatcgccactggcagcagccactggtaacaggattagcagagcgaggtatgtaggcggtgctacag
agttcttgaagtggtggcctaactacggctacactagaaggacagtatttggtatctgcgctctgctgaagccagttaccttcggaaaa
agagttggtagctcttgatccggcaaacaaaccaccgctggtagcggtggtttttttgtttgcaagcagcagattacgcgcagaaaaaa
aggatctcaagaagatcctttgatcttttctacggggtctgacgctcagtggaacgaaaactcacgttaagggattttggtcatgagatt
atcaaaaaggatcttcacctagatccttttaaattaaaaatgaagttttaaatcaatctaaagtatatatgagtaaacttggtctgaca
gttaccaatgcttaatcagtgaggcacctatctcagcgatctgtctatttcgttcatccatagttgcctgactccccgtcgtgtagataact
acgatacgggagggcttaccatctggccccagtgctgcaatgataccgcgagacccacgctcaccggctccagatttatcagcaataa
accagccagccggaagggccgagcgcagaagtggtcctgcaactttatccgcctccatccagtctattaattgttgccgggaagctaga
gtaagtagttcgccagttaatagtttgcgcaacgttgttgccattgctacaggcatcgtggtgtcacgctcgtcgtttggtatggcttcatt
cagctccggttcccaacgatcaaggcgagttacatgatcccccatgttgtgcaaaaaagcggttagctccttcggtcctccgatcgttgt
cagaagtaagttggccgcagtgttatcactcatggttatggcagcactgcataattctcttactgtcatgccatccgtaagatgcttttct
gtgactggtgagtactcaaccaagtcattctgagaatagtgtatgcggcgaccgagttgctcttgcccggcgtcaatacgggataatac
cgcgccacatagcagaactttaaaagtgctcatcattggaaaacgttcttcggggcgaaaactctcaaggatcttaccgctgttgagat
ccagttcgatgtaacccactcgtgcacccaactgatcttcagcatcttttactttcaccagcgtttctgggtgagcaaaaacaggaaggc
aaaatgccgcaaaaaagggaataagggcgacacggaaatgttgaatactcatactcttcctttttcaatattattgaagcatttatcag
ggttattgtctcatgagcggatacatatttgaatgtatttagaaaaataaacaaataggggttccgcgcacatttccccgaaaagtgcc
acctaaattgtaagcgttaatattttgttaaaattcgcgttaaatttttgttaaatcagctcattttttaaccaataggccgaaatcggca
aaatcccttataaatcaaaagaatagaccgagatagggttgagtgttgttccagtttggaacaagagtccactattaaagaacgtgga
ctccaacgtcaaagggcgaaaaaccgtctatcagggcgatggcccactacgtgaaccatcaccctaatcaagttttttggggtcgaggt
gccgtaaagcactaaatcggaaccctaaagggagcccccgatttagagcttgacggggaaagccggcgaacgtggcgagaaaggaa
gggaagaaagcgaaaggagcgggcgctagggcgctggcaagtgtagcggtcacgctgcgcgtaaccaccacacccgccgcgcttaat
gcgccgctacagggcgcgtcccattcgccattcaggctgcgcaactgttgggaagggcgatcggtgcgggcctcttcgctattacgcca
gctggcgaaagggggatgtgctgcaaggcgattaagttgggtaacgccagggttttcccagtcacgacgttgtaaaacgacggccagt
gagcgcgcgtaatacgactcactatagggcgaattgggtaccgggccccccctcgaggtcgacggtatcgataagcttgatatcgaatt
GGTCACCCGGCCAGCGACATGGAGGCCCAGAATACCCTCCTTGACAGTCTTGACGTGCGCAGCTCAGG
GGCATGATGTGACTGTCGCCCGTACATTTAGCCCATACATCCCCATGTATAATCATTTGCATCCATACAT
TTTGATGGCCGCACGGCGCGAAGCAAAAATTACGGCTCCTCGCTGCAGACCTGCGAGCAGGGAAACG
CTCCCCTCACAGACGCGTTGAATTGTCCCCACGCCGCGCCCCTGTAGAGAAATATAAAAGGTTAGGAT
TTGCCACTGAGGTTCTTCTTTCATATACTTCCTTTTAAAATCTTGCTAGGATACAGTTCTCACATCACATC
CGAACATAAACAACCATGACAGTCAACACTAAGACCTATAGTGAGAGAGCAGAAACTCATGCCTCACC
AGTAGCACAACGATTATTTCGATTAATGGAACTGAAGAAAACCAATTTATGTGCATCAATTGATGTTG**

ATACCACTAAGGAATTCCTTGAATTAATTGATAAATTGGGTCCTTATGTATGCTTAATCAAGACTCATA
TTGATATAATCAATGATTTTTCCTATGAATCCACTATTGAACCATTATTAGAACTTTCACGTAAACATCA
ATTTATGATTTTTGAAGATAGAAAATTTGCTGATATTGGTAATACCGTGAAGAAACAATATATTGGTG
GAGTTTATAAAATTAGTAGTTGGGCAGATATTACTAATGCTCATGGTGTCACTGGGAATGGAGTAGTT
GAAGGATTAAAACAGGGAGCTAAAGAAACCACCACCAACCAAGAGCCAAGAGGGTTATTGATGTTA
GCTGAATTATCATCAGTGGGATCATTAGCATATGGAGAATATTCTCAAAAAACTGTTGAAATTGCTAA
ATCCGATAAGGAATTTGTTATTGGATTTATTGCCCAACGTGATATGGGTGGACAAGAAGAAGGATTTG
ATTGGCTTATTATGACACCTGGAGTTGGATTAGATGATAAAGGTGATGGATTAGGACAACAATATAG
AACTGTTGATGAAGTTGTTAGCACTGGAACTGATATTATCATTGTTGGTAGAGGATTGTTTGGTAAAG
GAAGAGATCCAGATATTGAAGGTAAAAGGTATAGAGATGCTGGTTGGAATGCTTATTTGAAAAAGAC
TGGCCAATTATAATCAGTACTGACAATAAAAAGATTCTTGTTTTCAAGAACTTGTCATTTGTATAGTTTT
TTTATATTGTAGTTGTTCTATTTTAATCAAATGTTAGCGTGATTTATATTTTTTTTCGCCTCGACATCATC
TGCCCAGATGCGAAGTTAAGTGCGCAGAAAGTAATATCATGCGTCAATCGTATGTGAATGCTGGTCGC
TATACTGCTGTCGATTCGATACTAACGCCGCCGGATTAGAAGCCGCCGAGCGGGTGACAGCCCTCCGA
AGGAAGACTCTCCTCCGTGCGTCCTCGTCTTCACCGGTCGCGTTCCTGAAACGCAGATGTGCCTCGCGC
CGCACTGCTCCGAACAATAAAGATTCTACAATACTAGCTTTTATGGTTATGAAGAGGAAAAATTGGCA
GTAACCTGGCCCCACAAACCTTCAAATGAACGAATCAAATTAACAACCATAGGATGATAATGCGATTA
GTTTTTTAGCCTTATTTCTGGGGTAATTAATCAGCGAAGCGATGATTTTTGATCTATTAACAGATATATA
AATGCAAAAACTGCATAACCACTTTAACTAATACTTTCAACATTTTCGGTTTGTATTACTTCTTATTCAA
ATGTAATAAAAGTATCAACAAAAAAATTGTTAATATACCTCTATACTTTAACGTCAAGGAGATGATCAG
TCTGATTGCGGCGTTAGCGGTAGATCGCGTTATCGGCATGGAAAACGCCATGCCGTGGAACCTGCCTG
CCGATCTCGCCTGGTTTAAACGCAACACCTTAAATAAACCCGTGATTATGGGCCGCCATACCTGGGAAT
CAATCGGTCGTCCGTTGCCAGGACGCAAAAATATTATCCTCAGCAGTCAACCGGGTACGGACGATCGC
GTAACGTGGGTGAAGTCGGTGGATGAAGCCATCGCGGCGTGTGGTGACGTACCAGAAATCATGGTG
ATTGGCGGCGGTCGCGTTTATGAACAGTTCTTGCCAAAAGCGCAAAAACTGTATCTGACGCATATCGA
CGCAGAAGTGGAAGGCGACACCCATTTCCCGGATTACGAGCCGGATGACTGGGAATCGGTATTCAGC
GAATTCCACGATGCTGATGCGCAGAACTCTCACAGCTATTGCTTTGAGATTCTGGAGCGGCGGTACCC
ATACGATGTTCCTGACTATGCGGGCTATCCCTATGACGTCCCGGACTATGCGGGATCCTATCCATATGA
CGTTCCAGATTACGCTGCTCAGTGCGGTTCAGGTGGATCTGGTTCT

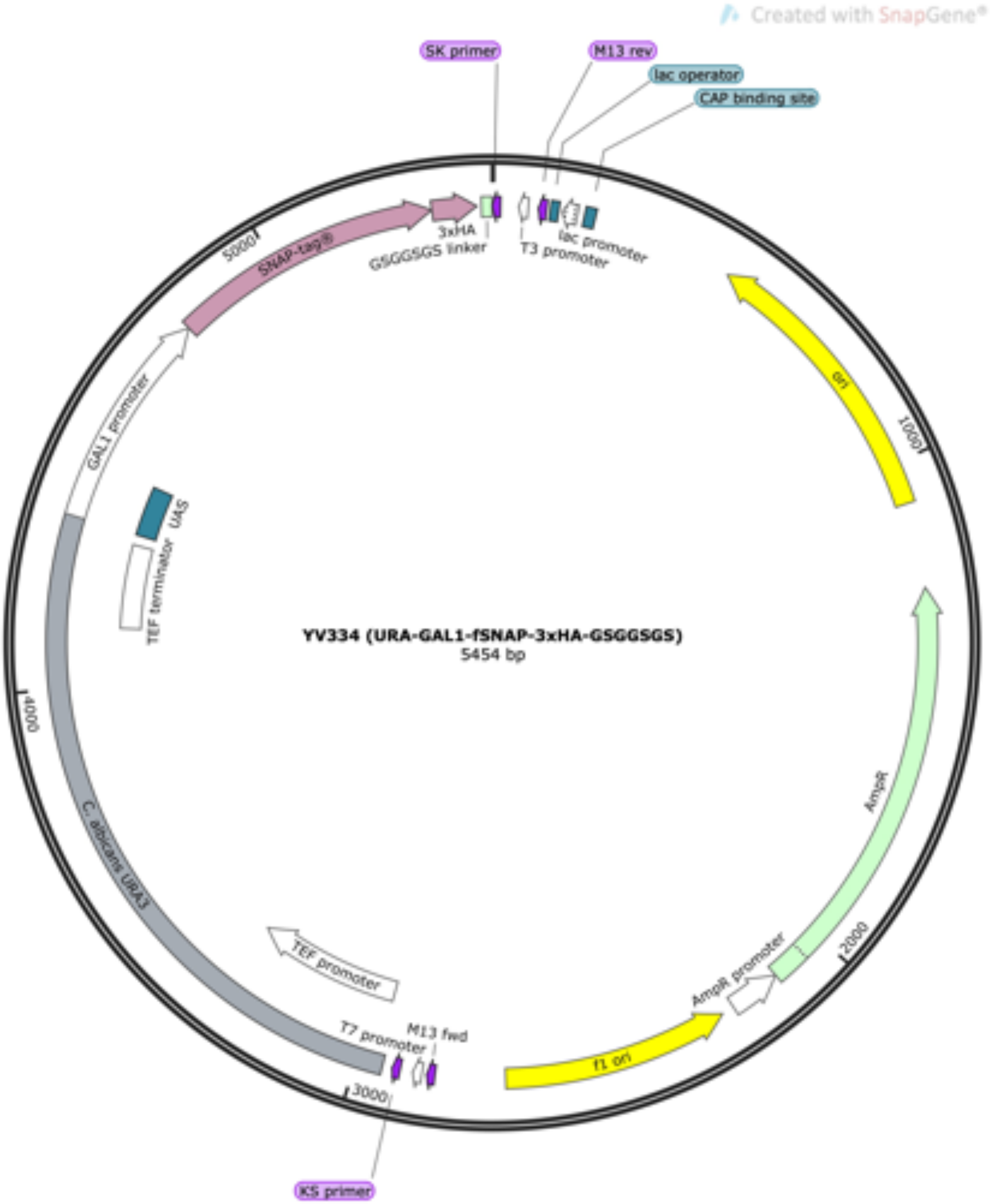
Created with SnapGene®
SK primer
M13 rev
lac operator
CAP binding site
3xHA
GSGGSGS linker
T3 promoter
lac promoter
5000
SNAP-tag®
ori
1000
GAL1 promoter
UAS
TEF terminator
YV334 (URA-GAL1-fSNAP-3xHA-GSGGSGS)
5454 bp
4000
AmpR
C. albicans URA3
TEF promoter
AmpR promoter
2000
T7 promoter
M13 fwd
f1 ori
3000
KS primer

**YV334: pBS-SKII-URA-GAL1-fSNAP-3xHA**

**gatccactagttctagagcggccgccaccgcggtggagctccagcttttgttccctttagtgagggttaattgcgcgcttggcgtaatcat
ggtcatagctgtttcctgtgtgaaattgttatccgctcacaattccacacaacatacgagccggaagcataaagtgtaaagcctggggt
gcctaatgagtgagctaactcacattaattgcgttgcgctcactgcccgctttccagtcgggaaacctgtcgtgccagctgcattaatga
atcggccaacgcgcggggagaggcggtttgcgtattgggcgctcttccgcttcctcgctcactgactcgctgcgctcggtcgttcggctgc
ggcgagcggtatcagctcactcaaaggcggtaatacggttatccacagaatcaggggataacgcaggaaagaacatgtgagcaaaa
ggccagcaaaaggccaggaaccgtaaaaaggccgcgttgctggcgtttttccataggctccgcccccctgacgagcatcacaaaaatc
gacgctcaagtcagaggtggcgaaacccgacaggactataaagataccaggcgtttccccctggaagctccctcgtgcgctctcctgtt
ccgaccctgccgcttaccggatacctgtccgcctttctcccttcgggaagcgtggcgctttctcatagctcacgctgtaggtatctcagttc
ggtgtaggtcgttcgctccaagctgggctgtgtgcacgaaccccccgttcagcccgaccgctgcgccttatccggtaactatcgtcttgag
tccaacccggtaagacacgacttatcgccactggcagcagccactggtaacaggattagcagagcgaggtatgtaggcggtgctacag
agttcttgaagtggtggcctaactacggctacactagaaggacagtatttggtatctgcgctctgctgaagccagttaccttcggaaaa
agagttggtagctcttgatccggcaaacaaaccaccgctggtagcggtggtttttttgtttgcaagcagcagattacgcgcagaaaaaa
aggatctcaagaagatcctttgatcttttctacggggtctgacgctcagtggaacgaaaactcacgttaagggattttggtcatgagatt
atcaaaaaggatcttcacctagatccttttaaattaaaaatgaagttttaaatcaatctaaagtatatatgagtaaacttggtctgaca
gttaccaatgcttaatcagtgaggcacctatctcagcgatctgtctatttcgttcatccatagttgcctgactccccgtcgtgtagataact
acgatacgggagggcttaccatctggccccagtgctgcaatgataccgcgagacccacgctcaccggctccagatttatcagcaataa
accagccagccggaagggccgagcgcagaagtggtcctgcaactttatccgcctccatccagtctattaattgttgccgggaagctaga
gtaagtagttcgccagttaatagtttgcgcaacgttgttgccattgctacaggcatcgtggtgtcacgctcgtcgtttggtatggcttcatt
cagctccggttcccaacgatcaaggcgagttacatgatcccccatgttgtgcaaaaaagcggttagctccttcggtcctccgatcgttgt
cagaagtaagttggccgcagtgttatcactcatggttatggcagcactgcataattctcttactgtcatgccatccgtaagatgcttttct
gtgactggtgagtactcaaccaagtcattctgagaatagtgtatgcggcgaccgagttgctcttgcccggcgtcaatacgggataatac
cgcgccacatagcagaactttaaaagtgctcatcattggaaaacgttcttcggggcgaaaactctcaaggatcttaccgctgttgagat
ccagttcgatgtaacccactcgtgcacccaactgatcttcagcatcttttactttcaccagcgtttctgggtgagcaaaaacaggaaggc
aaaatgccgcaaaaaagggaataagggcgacacggaaatgttgaatactcatactcttcctttttcaatattattgaagcatttatcag
ggttattgtctcatgagcggatacatatttgaatgtatttagaaaaataaacaaataggggttccgcgcacatttccccgaaaagtgcc
acctaaattgtaagcgttaatattttgttaaaattcgcgttaaatttttgttaaatcagctcattttttaaccaataggccgaaatcggca
aaatcccttataaatcaaaagaatagaccgagatagggttgagtgttgttccagtttggaacaagagtccactattaaagaacgtgga
ctccaacgtcaaagggcgaaaaaccgtctatcagggcgatggcccactacgtgaaccatcaccctaatcaagttttttggggtcgaggt
gccgtaaagcactaaatcggaaccctaaagggagcccccgatttagagcttgacggggaaagccggcgaacgtggcgagaaaggaa
gggaagaaagcgaaaggagcgggcgctagggcgctggcaagtgtagcggtcacgctgcgcgtaaccaccacacccgccgcgcttaat
gcgccgctacagggcgcgtcccattcgccattcaggctgcgcaactgttgggaagggcgatcggtgcgggcctcttcgctattacgcca
gctggcgaaagggggatgtgctgcaaggcgattaagttgggtaacgccagggttttcccagtcacgacgttgtaaaacgacggccagt
gagcgcgcgtaatacgactcactatagggcgaattgggtaccgggccccccctcgaggtcgacggtatcgataagcttgatatcgaatt
GGTCACCCGGCCAGCGACATGGAGGCCCAGAATACCCTCCTTGACAGTCTTGACGTGCGCAGCTCAGG
GGCATGATGTGACTGTCGCCCGTACATTTAGCCCATACATCCCCATGTATAATCATTTGCATCCATACAT
TTTGATGGCCGCACGGCGCGAAGCAAAAATTACGGCTCCTCGCTGCAGACCTGCGAGCAGGGAAACG
CTCCCCTCACAGACGCGTTGAATTGTCCCCACGCCGCGCCCCTGTAGAGAAATATAAAAGGTTAGGAT
TTGCCACTGAGGTTCTTCTTTCATATACTTCCTTTTAAAATCTTGCTAGGATACAGTTCTCACATCACATC
CGAACATAAACAACCATGACAGTCAACACTAAGACCTATAGTGAGAGAGCAGAAACTCATGCCTCACC
AGTAGCACAACGATTATTTCGATTAATGGAACTGAAGAAAACCAATTTATGTGCATCAATTGATGTTG
ATACCACTAAGGAATTCCTTGAATTAATTGATAAATTGGGTCCTTATGTATGCTTAATCAAGACTCATA
TTGATATAATCAATGATTTTTCCTATGAATCCACTATTGAACCATTATTAGAACTTTCACGTAAACATCA**

**ATTTATGATTTTTGAAGATAGAAAATTTGCTGATATTGGTAATACCGTGAAGAAACAATATATTGGTG
GAGTTTATAAAATTAGTAGTTGGGCAGATATTACTAATGCTCATGGTGTCACTGGGAATGGAGTAGTT
GAAGGATTAAAACAGGGAGCTAAAGAAACCACCACCAACCAAGAGCCAAGAGGGTTATTGATGTTA
GCTGAATTATCATCAGTGGGATCATTAGCATATGGAGAATATTCTCAAAAAACTGTTGAAATTGCTAA
ATCCGATAAGGAATTTGTTATTGGATTTATTGCCCAACGTGATATGGGTGGACAAGAAGAAGGATTTG
ATTGGCTTATTATGACACCTGGAGTTGGATTAGATGATAAAGGTGATGGATTAGGACAACAATATAG
AACTGTTGATGAAGTTGTTAGCACTGGAACTGATATTATCATTGTTGGTAGAGGATTGTTTGGTAAAG
GAAGAGATCCAGATATTGAAGGTAAAAGGTATAGAGATGCTGGTTGGAATGCTTATTTGAAAAAGAC
TGGCCAATTATAATCAGTACTGACAATAAAAAGATTCTTGTTTTCAAGAACTTGTCATTTGTATAGTTTT
TTTATATTGTAGTTGTTCTATTTTAATCAAATGTTAGCGTGATTTATATTTTTTTTCGCCTCGACATCATC
TGCCCAGATGCGAAGTTAAGTGCGCAGAAAGTAATATCATGCGTCAATCGTATGTGAATGCTGGTCGC
TATACTGCTGTCGATTCGATACTAACGCCGCCGGATTAGAAGCCGCCGAGCGGGTGACAGCCCTCCGA
AGGAAGACTCTCCTCCGTGCGTCCTCGTCTTCACCGGTCGCGTTCCTGAAACGCAGATGTGCCTCGCGC
CGCACTGCTCCGAACAATAAAGATTCTACAATACTAGCTTTTATGGTTATGAAGAGGAAAAATTGGCA
GTAACCTGGCCCCACAAACCTTCAAATGAACGAATCAAATTAACAACCATAGGATGATAATGCGATTA
GTTTTTTAGCCTTATTTCTGGGGTAATTAATCAGCGAAGCGATGATTTTTGATCTATTAACAGATATATA
AATGCAAAAACTGCATAACCACTTTAACTAATACTTTCAACATTTTCGGTTTGTATTACTTCTTATTCAA
ATGTAATAAAAGTATCAACAAAAAATTGTTAATATACCTCTATACTTTAACGTCAAGGAGATGGACAA
AGACTGCGAAATGAAGCGCACCACCCTGGATAGCCCTCTGGGCAAGCTGGAACTGTCTGGGTGCGAA
CAGGGCCTGCACCGTATCATCTTCCTGGGCAAAGGAACATCTGCCGCCGACGCCGTGGAAGTGCCTGC
CCCAGCCGCCGTGCTGGGCGGACCAGAGCCACTGATGCAGGCCACCGCCTGGCTCAACGCCTACTTTC
ACCAGCCTGAGGCCATCGAGGAGTTCCCTGTGCCAGCCCTGCACCACCCAGTGTTCCAGCAGGAGAGC
TTTACCCGCCAGGTGCTGTGGAAACTGCTGAAAGTGGTGAAGTTCGGAGAGGTCATCAGCTACAGCC
ACCTGGCCGCCCTGGCCGGCAATCCCGCCGCCACCGCCGCCGTGAAAACCGCCCTGAGCGGAAATCCC
GTGCCCATTCTGATCCCCTGCCACCGGGTGGTGCAGGGCGACCTGGACGTGGGGGGCTACGAGGGCG
GGCTCGCCGTGAAAGAGTGGCTGCTGGCCCACGAGGGCCACAGACTGGGCAAGCCTGGGCTGGGTTA
CCCATACGATGTTCCTGACTATGCGGGCTATCCCTATGACGTCCCGGACTATGCGGGATCCTATCCATA
TGACGTTCCAGATTACGCTGCTCAGTGCGGTTCAGGTGGATCTGGTTCT**

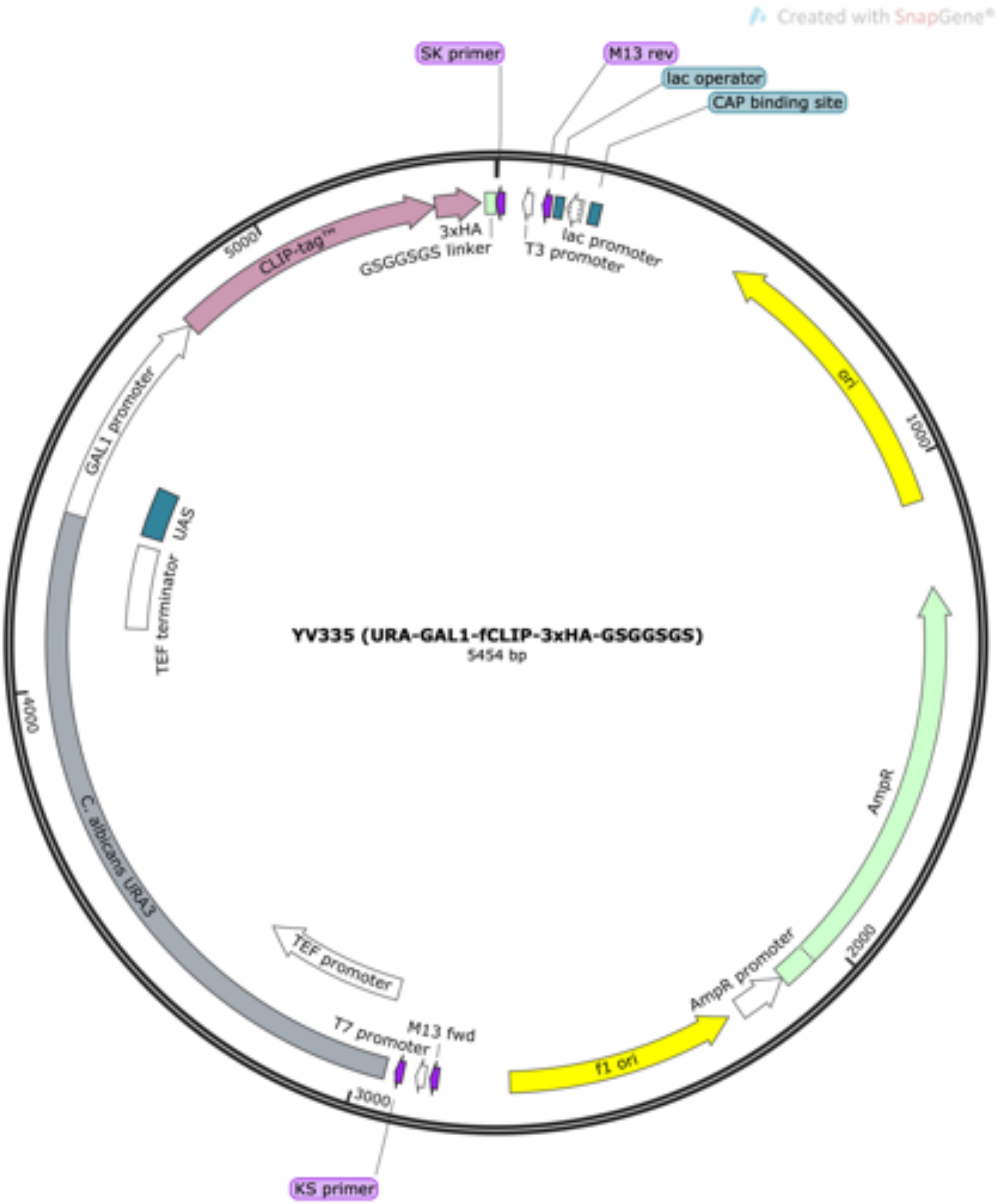

Created with SnapGene®
SK primer
M13 rev
lac operator
CAP binding site
3xHA
GSGGSGS linker
T3 promoter
lac promoter
5000
CLIP-tag™
ori
1000
GAL1 promoter
UAS
TEF terminator
YV335 (URA-GAL1-fCLIP-3xHA-GSGGSGS)
5454 bp
4000
C. albicans URA3
AmpR
TEF promoter
AmpR promoter
2000
T7 promoter
M13 fwd
f1 ori
3000
KS primer

**YV335: pBS-SKII-URA-GAL1-fCLIP-3xHA**

**gatccactagttctagagcggccgccaccgcggtggagctccagcttttgttccctttagtgagggttaattgcgcgcttggcgtaatcat
ggtcatagctgtttcctgtgtgaaattgttatccgctcacaattccacacaacatacgagccggaagcataaagtgtaaagcctggggt
gcctaatgagtgagctaactcacattaattgcgttgcgctcactgcccgctttccagtcgggaaacctgtcgtgccagctgcattaatga
atcggccaacgcgcggggagaggcggtttgcgtattgggcgctcttccgcttcctcgctcactgactcgctgcgctcggtcgttcggctgc
ggcgagcggtatcagctcactcaaaggcggtaatacggttatccacagaatcaggggataacgcaggaaagaacatgtgagcaaaa
ggccagcaaaaggccaggaaccgtaaaaaggccgcgttgctggcgtttttccataggctccgcccccctgacgagcatcacaaaaatc
gacgctcaagtcagaggtggcgaaacccgacaggactataaagataccaggcgtttccccctggaagctccctcgtgcgctctcctgtt
ccgaccctgccgcttaccggatacctgtccgcctttctcccttcgggaagcgtggcgctttctcatagctcacgctgtaggtatctcagttc
ggtgtaggtcgttcgctccaagctgggctgtgtgcacgaaccccccgttcagcccgaccgctgcgccttatccggtaactatcgtcttgag
tccaacccggtaagacacgacttatcgccactggcagcagccactggtaacaggattagcagagcgaggtatgtaggcggtgctacag
agttcttgaagtggtggcctaactacggctacactagaaggacagtatttggtatctgcgctctgctgaagccagttaccttcggaaaa
agagttggtagctcttgatccggcaaacaaaccaccgctggtagcggtggtttttttgtttgcaagcagcagattacgcgcagaaaaaa
aggatctcaagaagatcctttgatcttttctacggggtctgacgctcagtggaacgaaaactcacgttaagggattttggtcatgagatt
atcaaaaaggatcttcacctagatccttttaaattaaaaatgaagttttaaatcaatctaaagtatatatgagtaaacttggtctgaca
gttaccaatgcttaatcagtgaggcacctatctcagcgatctgtctatttcgttcatccatagttgcctgactccccgtcgtgtagataact
acgatacgggagggcttaccatctggccccagtgctgcaatgataccgcgagacccacgctcaccggctccagatttatcagcaataa
accagccagccggaagggccgagcgcagaagtggtcctgcaactttatccgcctccatccagtctattaattgttgccgggaagctaga
gtaagtagttcgccagttaatagtttgcgcaacgttgttgccattgctacaggcatcgtggtgtcacgctcgtcgtttggtatggcttcatt
cagctccggttcccaacgatcaaggcgagttacatgatcccccatgttgtgcaaaaaagcggttagctccttcggtcctccgatcgttgt
cagaagtaagttggccgcagtgttatcactcatggttatggcagcactgcataattctcttactgtcatgccatccgtaagatgcttttct
gtgactggtgagtactcaaccaagtcattctgagaatagtgtatgcggcgaccgagttgctcttgcccggcgtcaatacgggataatac
cgcgccacatagcagaactttaaaagtgctcatcattggaaaacgttcttcggggcgaaaactctcaaggatcttaccgctgttgagat
ccagttcgatgtaacccactcgtgcacccaactgatcttcagcatcttttactttcaccagcgtttctgggtgagcaaaaacaggaaggc
aaaatgccgcaaaaaagggaataagggcgacacggaaatgttgaatactcatactcttcctttttcaatattattgaagcatttatcag
ggttattgtctcatgagcggatacatatttgaatgtatttagaaaaataaacaaataggggttccgcgcacatttccccgaaaagtgcc
acctaaattgtaagcgttaatattttgttaaaattcgcgttaaatttttgttaaatcagctcattttttaaccaataggccgaaatcggca
aaatcccttataaatcaaaagaatagaccgagatagggttgagtgttgttccagtttggaacaagagtccactattaaagaacgtgga
ctccaacgtcaaagggcgaaaaaccgtctatcagggcgatggcccactacgtgaaccatcaccctaatcaagttttttggggtcgaggt
gccgtaaagcactaaatcggaaccctaaagggagcccccgatttagagcttgacggggaaagccggcgaacgtggcgagaaaggaa
gggaagaaagcgaaaggagcgggcgctagggcgctggcaagtgtagcggtcacgctgcgcgtaaccaccacacccgccgcgcttaat
gcgccgctacagggcgcgtcccattcgccattcaggctgcgcaactgttgggaagggcgatcggtgcgggcctcttcgctattacgcca
gctggcgaaagggggatgtgctgcaaggcgattaagttgggtaacgccagggttttcccagtcacgacgttgtaaaacgacggccagt
gagcgcgcgtaatacgactcactatagggcgaattgggtaccgggccccccctcgaggtcgacggtatcgataagcttgatatcgaatt
GGTCACCCGGCCAGCGACATGGAGGCCCAGAATACCCTCCTTGACAGTCTTGACGTGCGCAGCTCAGG
GGCATGATGTGACTGTCGCCCGTACATTTAGCCCATACATCCCCATGTATAATCATTTGCATCCATACAT
TTTGATGGCCGCACGGCGCGAAGCAAAAATTACGGCTCCTCGCTGCAGACCTGCGAGCAGGGAAACG
CTCCCCTCACAGACGCGTTGAATTGTCCCCACGCCGCGCCCCTGTAGAGAAATATAAAAGGTTAGGAT
TTGCCACTGAGGTTCTTCTTTCATATACTTCCTTTTAAAATCTTGCTAGGATACAGTTCTCACATCACATC
CGAACATAAACAACCATGACAGTCAACACTAAGACCTATAGTGAGAGAGCAGAAACTCATGCCTCACC
AGTAGCACAACGATTATTTCGATTAATGGAACTGAAGAAAACCAATTTATGTGCATCAATTGATGTTG
ATACCACTAAGGAATTCCTTGAATTAATTGATAAATTGGGTCCTTATGTATGCTTAATCAAGACTCATA
TTGATATAATCAATGATTTTTCCTATGAATCCACTATTGAACCATTATTAGAACTTTCACGTAAACATCA**

**ATTTATGATTTTTGAAGATAGAAAATTTGCTGATATTGGTAATACCGTGAAGAAACAATATATTGGTG
GAGTTTATAAAATTAGTAGTTGGGCAGATATTACTAATGCTCATGGTGTCACTGGGAATGGAGTAGTT
GAAGGATTAAAACAGGGAGCTAAAGAAACCACCACCAACCAAGAGCCAAGAGGGTTATTGATGTTA
GCTGAATTATCATCAGTGGGATCATTAGCATATGGAGAATATTCTCAAAAAACTGTTGAAATTGCTAA
ATCCGATAAGGAATTTGTTATTGGATTTATTGCCCAACGTGATATGGGTGGACAAGAAGAAGGATTTG
ATTGGCTTATTATGACACCTGGAGTTGGATTAGATGATAAAGGTGATGGATTAGGACAACAATATAG
AACTGTTGATGAAGTTGTTAGCACTGGAACTGATATTATCATTGTTGGTAGAGGATTGTTTGGTAAAG
GAAGAGATCCAGATATTGAAGGTAAAAGGTATAGAGATGCTGGTTGGAATGCTTATTTGAAAAAGAC
TGGCCAATTATAATCAGTACTGACAATAAAAAGATTCTTGTTTTCAAGAACTTGTCATTTGTATAGTTTT
TTTATATTGTAGTTGTTCTATTTTAATCAAATGTTAGCGTGATTTATATTTTTTTTCGCCTCGACATCATC
TGCCCAGATGCGAAGTTAAGTGCGCAGAAAGTAATATCATGCGTCAATCGTATGTGAATGCTGGTCGC
TATACTGCTGTCGATTCGATACTAACGCCGCCGGATTAGAAGCCGCCGAGCGGGTGACAGCCCTCCGA
AGGAAGACTCTCCTCCGTGCGTCCTCGTCTTCACCGGTCGCGTTCCTGAAACGCAGATGTGCCTCGCGC
CGCACTGCTCCGAACAATAAAGATTCTACAATACTAGCTTTTATGGTTATGAAGAGGAAAAATTGGCA
GTAACCTGGCCCCACAAACCTTCAAATGAACGAATCAAATTAACAACCATAGGATGATAATGCGATTA
GTTTTTTAGCCTTATTTCTGGGGTAATTAATCAGCGAAGCGATGATTTTTGATCTATTAACAGATATATA
AATGCAAAAACTGCATAACCACTTTAACTAATACTTTCAACATTTTCGGTTTGTATTACTTCTTATTCAA
ATGTAATAAAAGTATCAACAAAAAAATTGTTAATATACCTCTATACTTTAACGTCAAGGAGATGGACAA
AGACTGCGAAATGAAGCGCACCACCCTGGATAGCCCTCTGGGCAAGCTGGAACTGTCTGGGTGCGAA
CAGGGCCTGCACCGTATCATCTTCCTGGGCAAAGGAACATCTGCCGCCGACGCCGTGGAAGTGCCTGC
CCCAGCCGCCGTGCTGGGCGGACCAGAGCCACTGATCCAGGCCACCGCCTGGCTCAACGCCTACTTTC
ACCAGCCTGAGGCCATCGAGGAGTTCCCTGTGCCAGCCCTGCACCACCCAGTGTTCCAGCAGGAGAGC
TTTACCCGCCAGGTGCTGTGGAAACTGCTGAAAGTGGTGAAGTTCGGAGAGGTCATCAGCGAGAGCC
ACCTGGCCGCCCTGGTGGGCAATCCCGCCGCCACCGCCGCCGTGAACACCGCCCTGGACGGAAATCCC
GTGCCCATTCTGATCCCCTGCCACCGGGTGGTGCAGGGCGACAGCGACGTGGGGCCCTACCTGGGCG
GGCTCGCCGTGAAAGAGTGGCTGCTGGCCCACGAGGGCCACAGACTGGGCAAGCCTGGGCTGGGTTA
CCCATACGATGTTCCTGACTATGCGGGCTATCCCTATGACGTCCCGGACTATGCGGGATCCTATCCATA
TGACGTTCCAGATTACGCTGCTCAGTGCGGTTCAGGTGGATCTGGTTCT**

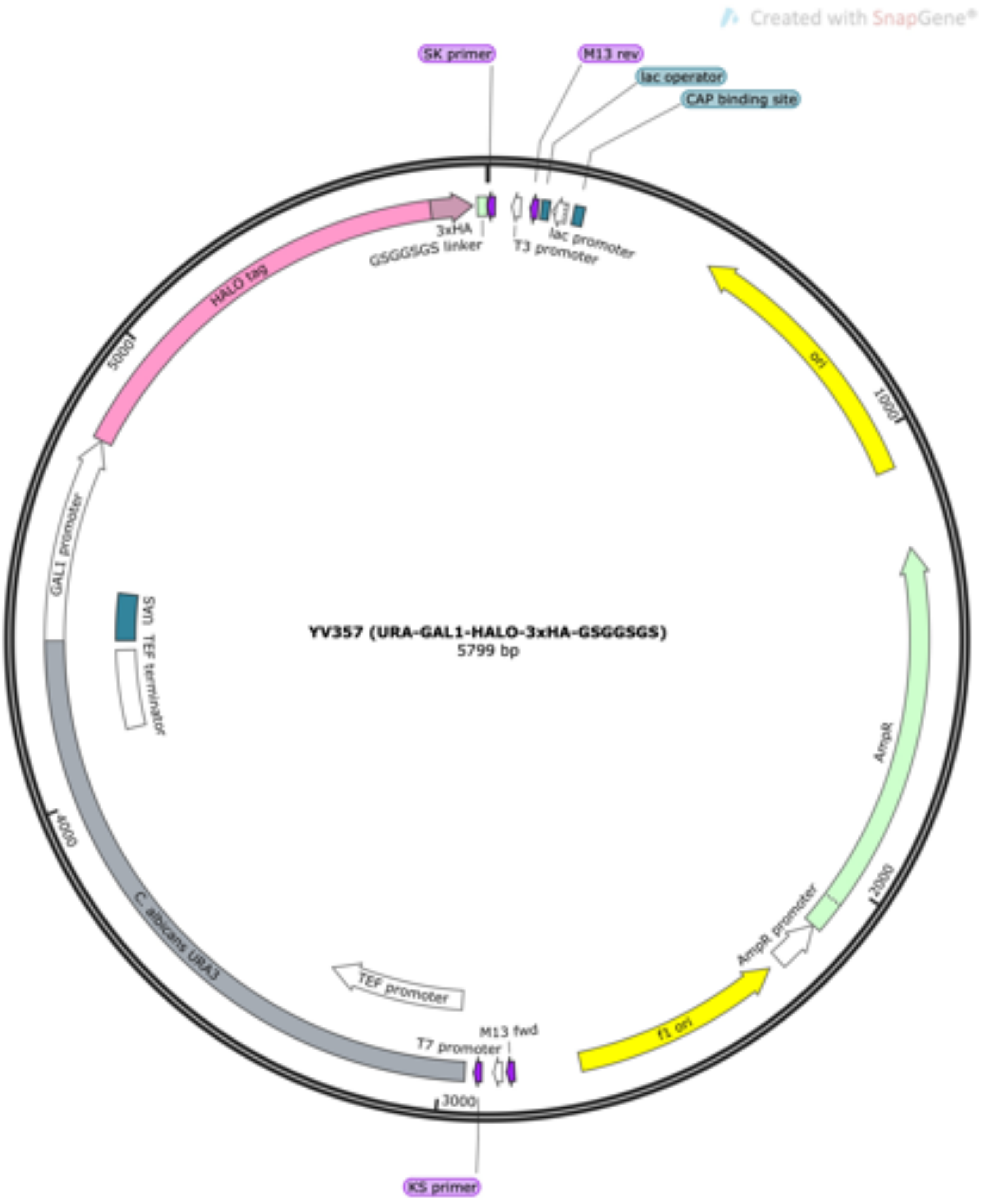

Created with SnapGene®
SK primer
M13 rev
lac operator
CAP binding site
3xHA
GSGGSGS linker
T3 promoter
lac promoter
HALO tag
ori
5000
1000
GAL1 promoter
SV40 NLS
TEF terminator
YV357 (URA-GAL1-HALO-3xHA-GSGGSGS)
5799 bp
AmpR
4000
2000
AmpR promoter
C. albicans URA3
TEF promoter
f1 ori
M13 fwd
T7 promoter
3000
KS primer

**YV357: pBS-SKII-URA-GAL1-HALO-3xHA**

**gatccactagttctagagcggccgccaccgcggtggagctccagcttttgttccctttagtgagggttaattgcgcgcttggcgtaatcat
ggtcatagctgtttcctgtgtgaaattgttatccgctcacaattccacacaacatacgagccggaagcataaagtgtaaagcctggggt
gcctaatgagtgagctaactcacattaattgcgttgcgctcactgcccgctttccagtcgggaaacctgtcgtgccagctgcattaatga
atcggccaacgcgcggggagaggcggtttgcgtattgggcgctcttccgcttcctcgctcactgactcgctgcgctcggtcgttcggctgc
ggcgagcggtatcagctcactcaaaggcggtaatacggttatccacagaatcaggggataacgcaggaaagaacatgtgagcaaaa
ggccagcaaaaggccaggaaccgtaaaaaggccgcgttgctggcgtttttccataggctccgcccccctgacgagcatcacaaaaatc
gacgctcaagtcagaggtggcgaaacccgacaggactataaagataccaggcgtttccccctggaagctccctcgtgcgctctcctgtt
ccgaccctgccgcttaccggatacctgtccgcctttctcccttcgggaagcgtggcgctttctcatagctcacgctgtaggtatctcagttc
ggtgtaggtcgttcgctccaagctgggctgtgtgcacgaaccccccgttcagcccgaccgctgcgccttatccggtaactatcgtcttgag
tccaacccggtaagacacgacttatcgccactggcagcagccactggtaacaggattagcagagcgaggtatgtaggcggtgctacag
agttcttgaagtggtggcctaactacggctacactagaaggacagtatttggtatctgcgctctgctgaagccagttaccttcggaaaa
agagttggtagctcttgatccggcaaacaaaccaccgctggtagcggtggtttttttgtttgcaagcagcagattacgcgcagaaaaaa
aggatctcaagaagatcctttgatcttttctacggggtctgacgctcagtggaacgaaaactcacgttaagggattttggtcatgagatt
atcaaaaaggatcttcacctagatccttttaaattaaaaatgaagttttaaatcaatctaaagtatatatgagtaaacttggtctgaca
gttaccaatgcttaatcagtgaggcacctatctcagcgatctgtctatttcgttcatccatagttgcctgactccccgtcgtgtagataact
acgatacgggagggcttaccatctggccccagtgctgcaatgataccgcgagacccacgctcaccggctccagatttatcagcaataa
accagccagccggaagggccgagcgcagaagtggtcctgcaactttatccgcctccatccagtctattaattgttgccgggaagctaga
gtaagtagttcgccagttaatagtttgcgcaacgttgttgccattgctacaggcatcgtggtgtcacgctcgtcgtttggtatggcttcatt
cagctccggttcccaacgatcaaggcgagttacatgatcccccatgttgtgcaaaaaagcggttagctccttcggtcctccgatcgttgt
cagaagtaagttggccgcagtgttatcactcatggttatggcagcactgcataattctcttactgtcatgccatccgtaagatgcttttct
gtgactggtgagtactcaaccaagtcattctgagaatagtgtatgcggcgaccgagttgctcttgcccggcgtcaatacgggataatac
cgcgccacatagcagaactttaaaagtgctcatcattggaaaacgttcttcggggcgaaaactctcaaggatcttaccgctgttgagat
ccagttcgatgtaacccactcgtgcacccaactgatcttcagcatcttttactttcaccagcgtttctgggtgagcaaaaacaggaaggc
aaaatgccgcaaaaaagggaataagggcgacacggaaatgttgaatactcatactcttcctttttcaatattattgaagcatttatcag
ggttattgtctcatgagcggatacatatttgaatgtatttagaaaaataaacaaataggggttccgcgcacatttccccgaaaagtgcc
acctaaattgtaagcgttaatattttgttaaaattcgcgttaaatttttgttaaatcagctcattttttaaccaataggccgaaatcggca
aaatcccttataaatcaaaagaatagaccgagatagggttgagtgttgttccagtttggaacaagagtccactattaaagaacgtgga
ctccaacgtcaaagggcgaaaaaccgtctatcagggcgatggcccactacgtgaaccatcaccctaatcaagttttttggggtcgaggt
gccgtaaagcactaaatcggaaccctaaagggagcccccgatttagagcttgacggggaaagccggcgaacgtggcgagaaaggaa
gggaagaaagcgaaaggagcgggcgctagggcgctggcaagtgtagcggtcacgctgcgcgtaaccaccacacccgccgcgcttaat
gcgccgctacagggcgcgtcccattcgccattcaggctgcgcaactgttgggaagggcgatcggtgcgggcctcttcgctattacgcca
gctggcgaaagggggatgtgctgcaaggcgattaagttgggtaacgccagggttttcccagtcacgacgttgtaaaacgacggccagt
gagcgcgcgtaatacgactcactatagggcgaattgggtaccgggccccccctcgaggtcgacggtatcgataagcttgatatcgaatt
GGTCACCCGGCCAGCGACATGGAGGCCCAGAATACCCTCCTTGACAGTCTTGACGTGCGCAGCTCAGG
GGCATGATGTGACTGTCGCCCGTACATTTAGCCCATACATCCCCATGTATAATCATTTGCATCCATACAT
TTTGATGGCCGCACGGCGCGAAGCAAAAATTACGGCTCCTCGCTGCAGACCTGCGAGCAGGGAAACG
CTCCCCTCACAGACGCGTTGAATTGTCCCCACGCCGCGCCCCTGTAGAGAAATATAAAAGGTTAGGAT
TTGCCACTGAGGTTCTTCTTTCATATACTTCCTTTTAAAATCTTGCTAGGATACAGTTCTCACATCACATC
CGAACATAAACAACCATGACAGTCAACACTAAGACCTATAGTGAGAGAGCAGAAACTCATGCCTCACC
AGTAGCACAACGATTATTTCGATTAATGGAACTGAAGAAAACCAATTTATGTGCATCAATTGATGTTG
ATACCACTAAGGAATTCCTTGAATTAATTGATAAATTGGGTCCTTATGTATGCTTAATCAAGACTCATA
TTGATATAATCAATGATTTTTCCTATGAATCCACTATTGAACCATTATTAGAACTTTCACGTAAACATCA**

**ATTTATGATTTTTGAAGATAGAAAATTTGCTGATATTGGTAATACCGTGAAGAAACAATATATTGGTG
GAGTTTATAAAATTAGTAGTTGGGCAGATATTACTAATGCTCATGGTGTCACTGGGAATGGAGTAGTT
GAAGGATTAAAACAGGGAGCTAAAGAAACCACCACCAACCAAGAGCCAAGAGGGTTATTGATGTTA
GCTGAATTATCATCAGTGGGATCATTAGCATATGGAGAATATTCTCAAAAAACTGTTGAAATTGCTAA
ATCCGATAAGGAATTTGTTATTGGATTTATTGCCCAACGTGATATGGGTGGACAAGAAGAAGGATTTG
ATTGGCTTATTATGACACCTGGAGTTGGATTAGATGATAAAGGTGATGGATTAGGACAACAATATAG
AACTGTTGATGAAGTTGTTAGCACTGGAACTGATATTATCATTGTTGGTAGAGGATTGTTTGGTAAAG
GAAGAGATCCAGATATTGAAGGTAAAAGGTATAGAGATGCTGGTTGGAATGCTTATTTGAAAAAGAC
TGGCCAATTATAATCAGTACTGACAATAAAAAGATTCTTGTTTTCAAGAACTTGTCATTTGTATAGTTTT
TTTATATTGTAGTTGTTCTATTTTAATCAAATGTTAGCGTGATTTATATTTTTTTTCGCCTCGACATCATC
TGCCCAGATGCGAAGTTAAGTGCGCAGAAAGTAATATCATGCGTCAATCGTATGTGAATGCTGGTCGC
TATACTGCTGTCGATTCGATACTAACGCCGCCGGATTAGAAGCCGCCGAGCGGGTGACAGCCCTCCGA
AGGAAGACTCTCCTCCGTGCGTCCTCGTCTTCACCGGTCGCGTTCCTGAAACGCAGATGTGCCTCGCGC
CGCACTGCTCCGAACAATAAAGATTCTACAATACTAGCTTTTATGGTTATGAAGAGGAAAAATTGGCA
GTAACCTGGCCCCACAAACCTTCAAATGAACGAATCAAATTAACAACCATAGGATGATAATGCGATTA
GTTTTTTAGCCTTATTTCTGGGGTAATTAATCAGCGAAGCGATGATTTTTGATCTATTAACAGATATATA
AATGCAAAAACTGCATAACCACTTTAACTAATACTTTCAACATTTTCGGTTTGTATTACTTCTTATTCAA
ATGTAATAAAAGTATCAACAAAAAAATTGTTAATATACCTCTATACTTTAACGTCAAGGAGATGGCAGA
AATCGGTACTGGCTTTCCATTCGACCCGCATTATGTGGAAGTCCTGGGCGAGAGAATGCATTACGTTG
ACGTGGGTCCGAGAGATGGAACTCCGGTCCTTTTTCTGCACGGGAATCCTACAAGCTCTTATGTTTGGC
GCAATATCATCCCTCATGTAGCTCCGACGCATCGCTGTATTGCGCCGGACCTGATTGGTATGGGAAAAT
CTGATAAACCAGACCTGGGTTACTTTTTCGATGATCATGTGCGTTTCATGGATGCCTTCATTGAGGCAT
TAGGGCTTGAAGAAGTCGTCTTGGTGATTCATGATTGGGGCTCAGCTCTGGGATTTCACTGGGCTAAA
AGAAATCCTGAACGCGTAAAAGGCATCGCGTTTATGGAGTTCATTCGTCCAATTCCGACTTGGGATGA
ATGGCCTGAGTTCGCGAGAGAAACATTTCAAGCATTTCGCACGACCGATGTAGGCCGGAAGTTAATCA
TCGATCAGAATGTCTTTATCGAAGGGACATTGCCGATGGGAGTCGTTCGTCCGTTAACAGAAGTCGAA
ATGGATCACTATAGAGAACCTTTTCTTAATCCTGTGGACAGAGAGCCGCTGTGGCGGTTTCCGAACGA
ACTGCCGATTGCAGGCGAGCCTGCTAACATTGTAGCGCTGGTTGAAGAGTATATGGATTGGCTTCATC
AGTCTCCAGTTCCGAAGTTATTGTTTTGGGGTACGCCTGGCGTGCTTATTCCACCGGCCGAAGCGGCAC
GTTTGGCAAAAAGCCTGCCAAATTGCAAAGCCGTTGACATTGGCCCTGGACTTAACTTGCTTCAAGAG
GATAACCCGGACTTAATCGGGAGCGAAATTGCCCGGTGGCTTTCTACCTTAGAAATCAGCGGCTACCC
ATACGATGTTCCTGACTATGCGGGCTATCCCTATGACGTCCCGGACTATGCGGGATCCTATCCATATGA
CGTTCCAGATTACGCTGCTCAGTGCGGTTCAGGTGGATCTGGTTCT**

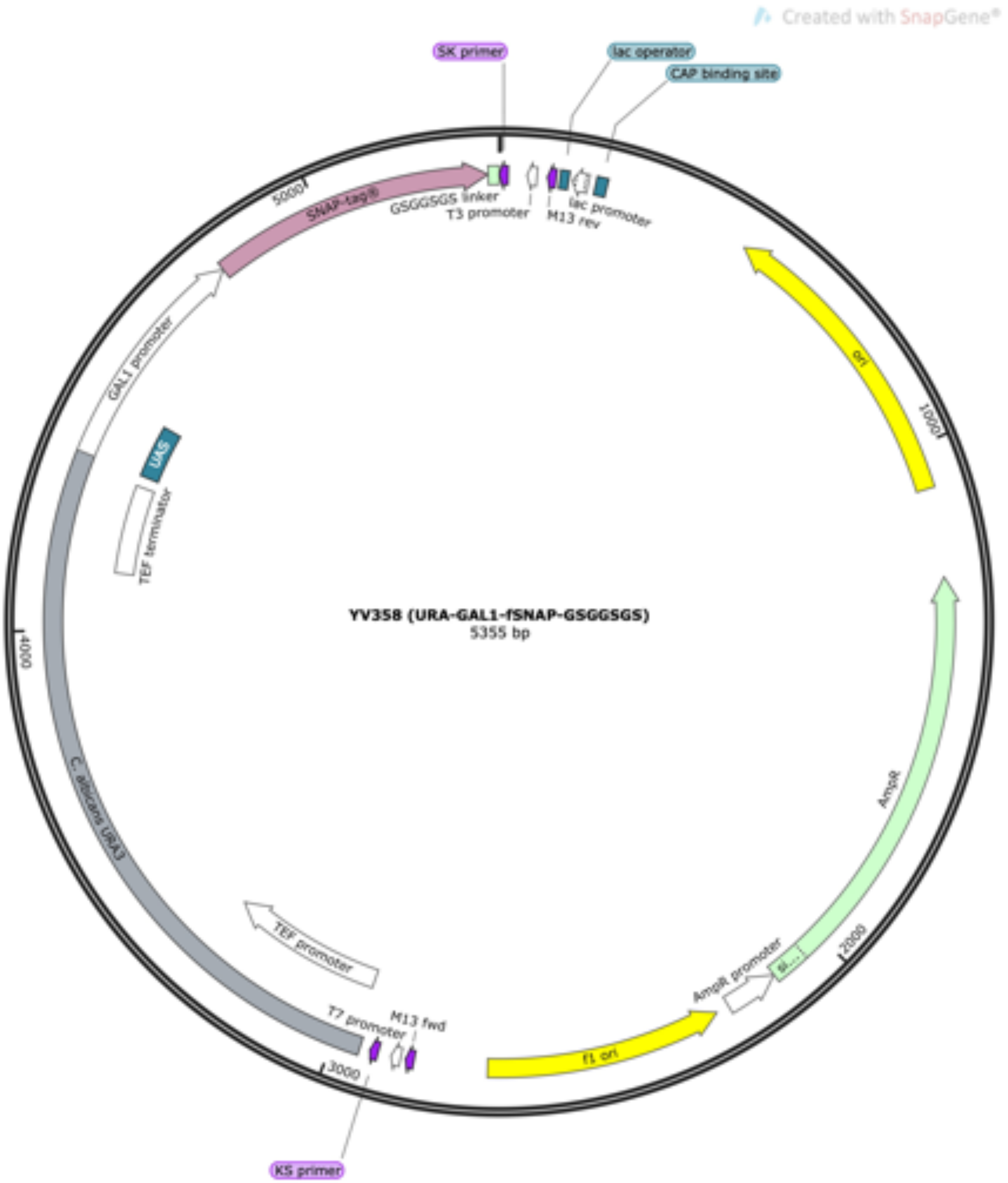
Created with SnapGene®
SK primer
lac operator
CAP binding site
SNAP-tag®
GSGGSGS linker
T3 promoter
M13 rev
lac promoter
5000
ori
1000
GAL1 promoter
UAS
TEF terminator
YV358 (URA-GAL1-fSNAP-GSGGSGS)
5355 bp
4000
C. albicans URA3
AmpR
TEF promoter
2000
AmpR promoter
T7 promoter
M13 fwd
f1 ori
3000
KS primer

**YV358: pBS-SKII-URA-GAL1-fSNAP-GS**

gatccactagttctagagcggccgccaccgcggtggagctccagcttttgttccctttagtgagggttaattgcgcgcttggcgtaatcat
ggtcatagctgtttcctgtgtgaaattgttatccgctcacaattccacacaacatacgagccggaagcataaagtgtaaagcctggggt
gcctaatgagtgagctaactcacattaattgcgttgcgctcactgcccgctttccagtcgggaaacctgtcgtgccagctgcattaatga
atcggccaacgcgcggggagaggcggtttgcgtattgggcgctcttccgcttcctcgctcactgactcgctgcgctcggtcgttcggctgc
ggcgagcggtatcagctcactcaaaggcggtaatacggttatccacagaatcaggggataacgcaggaaagaacatgtgagcaaaa
ggccagcaaaaggccaggaaccgtaaaaaggccgcgttgctggcgtttttccataggctccgcccccctgacgagcatcacaaaaatc
gacgctcaagtcagaggtggcgaaacccgacaggactataaagataccaggcgtttccccctggaagctccctcgtgcgctctcctgtt
ccgaccctgccgcttaccggatacctgtccgcctttctcccttcgggaagcgtggcgctttctcatagctcacgctgtaggtatctcagttc
ggtgtaggtcgttcgctccaagctgggctgtgtgcacgaaccccccgttcagcccgaccgctgcgccttatccggtaactatcgtcttgag
tccaacccggtaagacacgacttatcgccactggcagcagccactggtaacaggattagcagagcgaggtatgtaggcggtgctacag
agttcttgaagtggtggcctaactacggctacactagaaggacagtatttggtatctgcgctctgctgaagccagttaccttcggaaaa
agagttggtagctcttgatccggcaaacaaaccaccgctggtagcggtggtttttttgtttgcaagcagcagattacgcgcagaaaaaa
aggatctcaagaagatcctttgatcttttctacggggtctgacgctcagtggaacgaaaactcacgttaagggattttggtcatgagatt
atcaaaaaggatcttcacctagatccttttaaattaaaaatgaagttttaaatcaatctaaagtatatatgagtaaacttggtctgaca
gttaccaatgcttaatcagtgaggcacctatctcagcgatctgtctatttcgttcatccatagttgcctgactccccgtcgtgtagataact
acgatacgggagggcttaccatctggccccagtgctgcaatgataccgcgagacccacgctcaccggctccagatttatcagcaataa
accagccagccggaagggccgagcgcagaagtggtcctgcaactttatccgcctccatccagtctattaattgttgccgggaagctaga
gtaagtagttcgccagttaatagtttgcgcaacgttgttgccattgctacaggcatcgtggtgtcacgctcgtcgtttggtatggcttcatt
cagctccggttcccaacgatcaaggcgagttacatgatcccccatgttgtgcaaaaaagcggttagctccttcggtcctccgatcgttgt
cagaagtaagttggccgcagtgttatcactcatggttatggcagcactgcataattctcttactgtcatgccatccgtaagatgcttttct
gtgactggtgagtactcaaccaagtcattctgagaatagtgtatgcggcgaccgagttgctcttgcccggcgtcaatacgggataatac
cgcgccacatagcagaactttaaaagtgctcatcattggaaaacgttcttcggggcgaaaactctcaaggatcttaccgctgttgagat
ccagttcgatgtaacccactcgtgcacccaactgatcttcagcatcttttactttcaccagcgtttctgggtgagcaaaaacaggaaggc
aaaatgccgcaaaaaagggaataagggcgacacggaaatgttgaatactcatactcttcctttttcaatattattgaagcatttatcag
ggttattgtctcatgagcggatacatatttgaatgtatttagaaaaataaacaaataggggttccgcgcacatttccccgaaaagtgcc
acctaaattgtaagcgttaatattttgttaaaattcgcgttaaatttttgttaaatcagctcattttttaaccaataggccgaaatcggca
aaatcccttataaatcaaaagaatagaccgagatagggttgagtgttgttccagtttggaacaagagtccactattaaagaacgtgga
ctccaacgtcaaagggcgaaaaaccgtctatcagggcgatggcccactacgtgaaccatcaccctaatcaagttttttggggtcgaggt
gccgtaaagcactaaatcggaaccctaaagggagcccccgatttagagcttgacggggaaagccggcgaacgtggcgagaaaggaa
gggaagaaagcgaaaggagcgggcgctagggcgctggcaagtgtagcggtcacgctgcgcgtaaccaccacacccgccgcgcttaat
gcgccgctacagggcgcgtcccattcgccattcaggctgcgcaactgttgggaagggcgatcggtgcgggcctcttcgctattacgcca
gctggcgaaagggggatgtgctgcaaggcgattaagttgggtaacgccagggttttcccagtcacgacgttgtaaaacgacggccagt
gagcgcgcgtaatacgactcactatagggcgaattgggtaccgggccccccctcgaggtcgacggtatcgataagcttgatatcgaatt
GGTCACCCGGCCAGCGACATGGAGGCCCAGAATACCCTCCTTGACAGTCTTGACGTGCGCAGCTCAGG
GGCATGATGTGACTGTCGCCCGTACATTTAGCCCATACATCCCCATGTATAATCATTTGCATCCATACAT
TTTGATGGCCGCACGGCGCGAAGCAAAAATTACGGCTCCTCGCTGCAGACCTGCGAGCAGGGAAACG
CTCCCCTCACAGACGCGTTGAATTGTCCCCACGCCGCGCCCCTGTAGAGAAATATAAAAGGTTAGGAT
TTGCCACTGAGGTTCTTCTTTCATATACTTCCTTTTAAAATCTTGCTAGGATACAGTTCTCACATCACATC
CGAACATAAACAACCATGACAGTCAACACTAAGACCTATAGTGAGAGAGCAGAAACTCATGCCTCACC
AGTAGCACAACGATTATTTCGATTAATGGAACTGAAGAAAACCAATTTATGTGCATCAATTGATGTTG
ATACCACTAAGGAATTCCTTGAATTAATTGATAAATTGGGTCCTTATGTATGCTTAATCAAGACTCATA
TTGATATAATCAATGATTTTTCCTATGAATCCACTATTGAACCATTATTAGAACTTTCACGTAAACATCA

**ATTTATGATTTTTGAAGATAGAAAATTTGCTGATATTGGTAATACCGTGAAGAAACAATATATTGGTG
GAGTTTATAAAATTAGTAGTTGGGCAGATATTACTAATGCTCATGGTGTCACTGGGAATGGAGTAGTT
GAAGGATTAAAACAGGGAGCTAAAGAAACCACCACCAACCAAGAGCCAAGAGGGTTATTGATGTTA
GCTGAATTATCATCAGTGGGATCATTAGCATATGGAGAATATTCTCAAAAAACTGTTGAAATTGCTAA
ATCCGATAAGGAATTTGTTATTGGATTTATTGCCCAACGTGATATGGGTGGACAAGAAGAAGGATTTG
ATTGGCTTATTATGACACCTGGAGTTGGATTAGATGATAAAGGTGATGGATTAGGACAACAATATAG
AACTGTTGATGAAGTTGTTAGCACTGGAACTGATATTATCATTGTTGGTAGAGGATTGTTTGGTAAAG
GAAGAGATCCAGATATTGAAGGTAAAAGGTATAGAGATGCTGGTTGGAATGCTTATTTGAAAAAGAC
TGGCCAATTATAATCAGTACTGACAATAAAAAGATTCTTGTTTTCAAGAACTTGTCATTTGTATAGTTTT
TTTATATTGTAGTTGTTCTATTTTAATCAAATGTTAGCGTGATTTATATTTTTTTTCGCCTCGACATCATC
TGCCCAGATGCGAAGTTAAGTGCGCAGAAAGTAATATCATGCGTCAATCGTATGTGAATGCTGGTCGC
TATACTGCTGTCGATTCGATACTAACGCCGCCGGATTAGAAGCCGCCGAGCGGGTGACAGCCCTCCGA
AGGAAGACTCTCCTCCGTGCGTCCTCGTCTTCACCGGTCGCGTTCCTGAAACGCAGATGTGCCTCGCGC
CGCACTGCTCCGAACAATAAAGATTCTACAATACTAGCTTTTATGGTTATGAAGAGGAAAAATTGGCA
GTAACCTGGCCCCACAAACCTTCAAATGAACGAATCAAATTAACAACCATAGGATGATAATGCGATTA
GTTTTTTAGCCTTATTTCTGGGGTAATTAATCAGCGAAGCGATGATTTTTGATCTATTAACAGATATATA
AATGCAAAAACTGCATAACCACTTTAACTAATACTTTCAACATTTTCGGTTTGTATTACTTCTTATTCAA
ATGTAATAAAAGTATCAACAAAAAAATTGTTAATATACCTCTATACTTTAACGTCAAGGAGATGGACAA
AGACTGCGAAATGAAGCGCACCACCCTGGATAGCCCTCTGGGCAAGCTGGAACTGTCTGGGTGCGAA
CAGGGCCTGCACCGTATCATCTTCCTGGGCAAAGGAACATCTGCCGCCGACGCCGTGGAAGTGCCTGC
CCCAGCCGCCGTGCTGGGCGGACCAGAGCCACTGATGCAGGCCACCGCCTGGCTCAACGCCTACTTTC
ACCAGCCTGAGGCCATCGAGGAGTTCCCTGTGCCAGCCCTGCACCACCCAGTGTTCCAGCAGGAGAGC
TTTACCCGCCAGGTGCTGTGGAAACTGCTGAAAGTGGTGAAGTTCGGAGAGGTCATCAGCTACAGCC
ACCTGGCCGCCCTGGCCGGCAATCCCGCCGCCACCGCCGCCGTGAAAACCGCCCTGAGCGGAAATCCC
GTGCCCATTCTGATCCCCTGCCACCGGGTGGTGCAGGGCGACCTGGACGTGGGGGGCTACGAGGGCG
GGCTCGCCGTGAAAGAGTGGCTGCTGGCCCACGAGGGCCACAGACTGGGCAAGCCTGGGCTGGGTG
GTTCAGGTGGATCTGGTTCT**

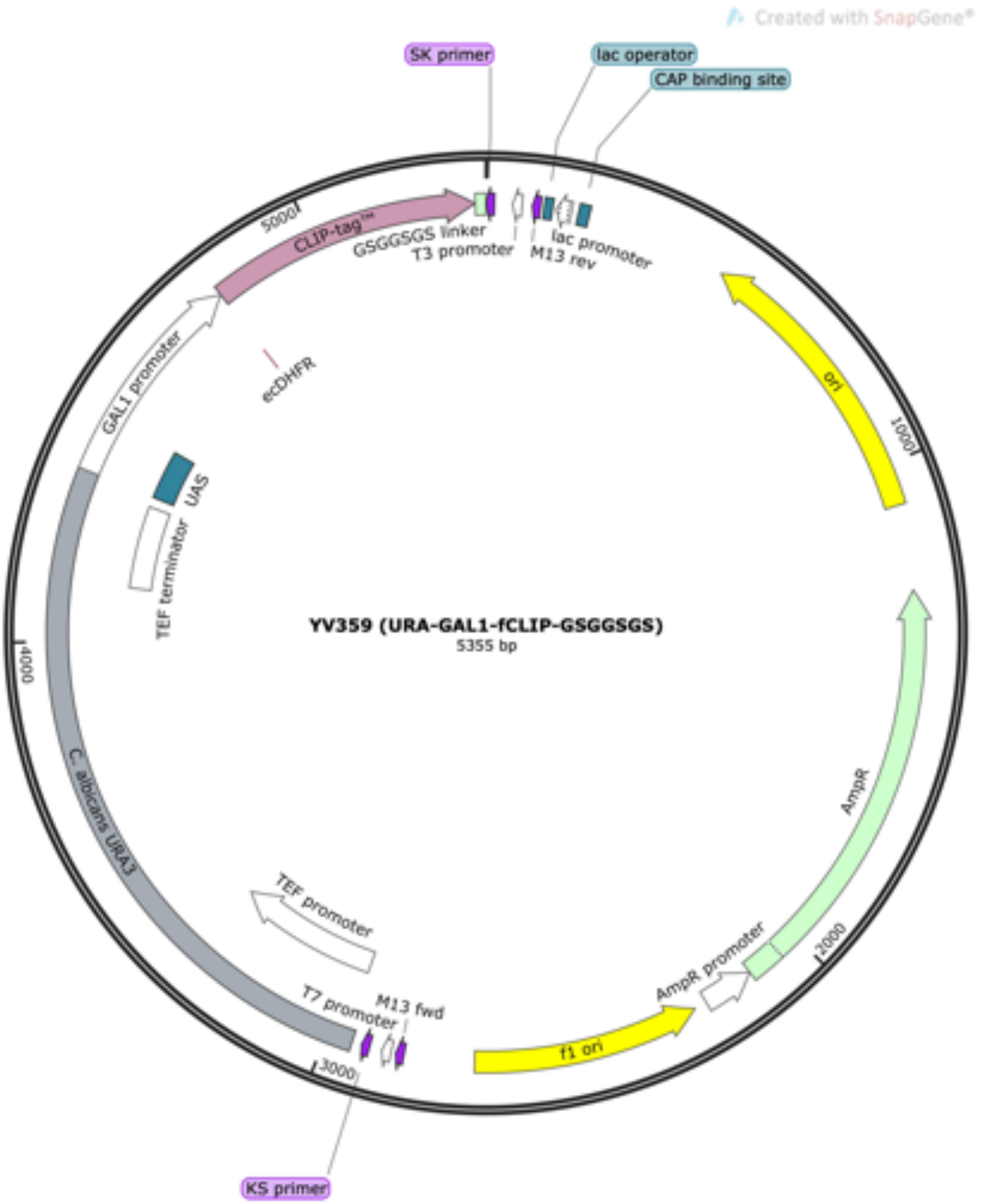
Created with SnapGene®
SK primer
lac operator
CAP binding site
5000
CLIP-tag™
GSGGSGS linker
T3 promoter
M13 rev
lac promoter
ori
1000
GAL1 promoter
ecDHFR
UAS
TEF terminator
YV359 (URA-GAL1-fCLIP-GSGGSGS)
5355 bp
4000
C. albicans URA3
AmpR
TEF promoter
AmpR promoter
2000
T7 promoter
M13 fwd
f1 ori
3000
KS primer

**YV359: pBS-SKII-URA-GAL1-fCLIP-GS**

**gatccactagttctagagcggccgccaccgcggtggagctccagcttttgttccctttagtgagggttaattgcgcgcttggcgtaatcat
ggtcatagctgtttcctgtgtgaaattgttatccgctcacaattccacacaacatacgagccggaagcataaagtgtaaagcctggggt
gcctaatgagtgagctaactcacattaattgcgttgcgctcactgcccgctttccagtcgggaaacctgtcgtgccagctgcattaatga
atcggccaacgcgcggggagaggcggtttgcgtattgggcgctcttccgcttcctcgctcactgactcgctgcgctcggtcgttcggctgc
ggcgagcggtatcagctcactcaaaggcggtaatacggttatccacagaatcaggggataacgcaggaaagaacatgtgagcaaaa
ggccagcaaaaggccaggaaccgtaaaaaggccgcgttgctggcgtttttccataggctccgcccccctgacgagcatcacaaaaatc
gacgctcaagtcagaggtggcgaaacccgacaggactataaagataccaggcgtttccccctggaagctccctcgtgcgctctcctgtt
ccgaccctgccgcttaccggatacctgtccgcctttctcccttcgggaagcgtggcgctttctcatagctcacgctgtaggtatctcagttc
ggtgtaggtcgttcgctccaagctgggctgtgtgcacgaaccccccgttcagcccgaccgctgcgccttatccggtaactatcgtcttgag
tccaacccggtaagacacgacttatcgccactggcagcagccactggtaacaggattagcagagcgaggtatgtaggcggtgctacag
agttcttgaagtggtggcctaactacggctacactagaaggacagtatttggtatctgcgctctgctgaagccagttaccttcggaaaa
agagttggtagctcttgatccggcaaacaaaccaccgctggtagcggtggtttttttgtttgcaagcagcagattacgcgcagaaaaaa
aggatctcaagaagatcctttgatcttttctacggggtctgacgctcagtggaacgaaaactcacgttaagggattttggtcatgagatt
atcaaaaaggatcttcacctagatccttttaaattaaaaatgaagttttaaatcaatctaaagtatatatgagtaaacttggtctgaca
gttaccaatgcttaatcagtgaggcacctatctcagcgatctgtctatttcgttcatccatagttgcctgactccccgtcgtgtagataact
acgatacgggagggcttaccatctggccccagtgctgcaatgataccgcgagacccacgctcaccggctccagatttatcagcaataa
accagccagccggaagggccgagcgcagaagtggtcctgcaactttatccgcctccatccagtctattaattgttgccgggaagctaga
gtaagtagttcgccagttaatagtttgcgcaacgttgttgccattgctacaggcatcgtggtgtcacgctcgtcgtttggtatggcttcatt
cagctccggttcccaacgatcaaggcgagttacatgatcccccatgttgtgcaaaaaagcggttagctccttcggtcctccgatcgttgt
cagaagtaagttggccgcagtgttatcactcatggttatggcagcactgcataattctcttactgtcatgccatccgtaagatgcttttct
gtgactggtgagtactcaaccaagtcattctgagaatagtgtatgcggcgaccgagttgctcttgcccggcgtcaatacgggataatac
cgcgccacatagcagaactttaaaagtgctcatcattggaaaacgttcttcggggcgaaaactctcaaggatcttaccgctgttgagat
ccagttcgatgtaacccactcgtgcacccaactgatcttcagcatcttttactttcaccagcgtttctgggtgagcaaaaacaggaaggc
aaaatgccgcaaaaaagggaataagggcgacacggaaatgttgaatactcatactcttcctttttcaatattattgaagcatttatcag
ggttattgtctcatgagcggatacatatttgaatgtatttagaaaaataaacaaataggggttccgcgcacatttccccgaaaagtgcc
acctaaattgtaagcgttaatattttgttaaaattcgcgttaaatttttgttaaatcagctcattttttaaccaataggccgaaatcggca
aaatcccttataaatcaaaagaatagaccgagatagggttgagtgttgttccagtttggaacaagagtccactattaaagaacgtgga
ctccaacgtcaaagggcgaaaaaccgtctatcagggcgatggcccactacgtgaaccatcaccctaatcaagttttttggggtcgaggt
gccgtaaagcactaaatcggaaccctaaagggagcccccgatttagagcttgacggggaaagccggcgaacgtggcgagaaaggaa
gggaagaaagcgaaaggagcgggcgctagggcgctggcaagtgtagcggtcacgctgcgcgtaaccaccacacccgccgcgcttaat
gcgccgctacagggcgcgtcccattcgccattcaggctgcgcaactgttgggaagggcgatcggtgcgggcctcttcgctattacgcca
gctggcgaaagggggatgtgctgcaaggcgattaagttgggtaacgccagggttttcccagtcacgacgttgtaaaacgacggccagt
gagcgcgcgtaatacgactcactatagggcgaattgggtaccgggccccccctcgaggtcgacggtatcgataagcttgatatcgaatt
GGTCACCCGGCCAGCGACATGGAGGCCCAGAATACCCTCCTTGACAGTCTTGACGTGCGCAGCTCAGG
GGCATGATGTGACTGTCGCCCGTACATTTAGCCCATACATCCCCATGTATAATCATTTGCATCCATACAT
TTTGATGGCCGCACGGCGCGAAGCAAAAATTACGGCTCCTCGCTGCAGACCTGCGAGCAGGGAAACG
CTCCCCTCACAGACGCGTTGAATTGTCCCCACGCCGCGCCCCTGTAGAGAAATATAAAAGGTTAGGAT
TTGCCACTGAGGTTCTTCTTTCATATACTTCCTTTTAAAATCTTGCTAGGATACAGTTCTCACATCACATC
CGAACATAAACAACCATGACAGTCAACACTAAGACCTATAGTGAGAGAGCAGAAACTCATGCCTCACC
AGTAGCACAACGATTATTTCGATTAATGGAACTGAAGAAAACCAATTTATGTGCATCAATTGATGTTG
ATACCACTAAGGAATTCCTTGAATTAATTGATAAATTGGGTCCTTATGTATGCTTAATCAAGACTCATA
TTGATATAATCAATGATTTTTCCTATGAATCCACTATTGAACCATTATTAGAACTTTCACGTAAACATCA**

**ATTTATGATTTTTGAAGATAGAAAATTTGCTGATATTGGTAATACCGTGAAGAAACAATATATTGGTG
GAGTTTATAAAATTAGTAGTTGGGCAGATATTACTAATGCTCATGGTGTCACTGGGAATGGAGTAGTT
GAAGGATTAAAACAGGGAGCTAAAGAAACCACCACCAACCAAGAGCCAAGAGGGTTATTGATGTTA
GCTGAATTATCATCAGTGGGATCATTAGCATATGGAGAATATTCTCAAAAAACTGTTGAAATTGCTAA
ATCCGATAAGGAATTTGTTATTGGATTTATTGCCCAACGTGATATGGGTGGACAAGAAGAAGGATTTG
ATTGGCTTATTATGACACCTGGAGTTGGATTAGATGATAAAGGTGATGGATTAGGACAACAATATAG
AACTGTTGATGAAGTTGTTAGCACTGGAACTGATATTATCATTGTTGGTAGAGGATTGTTTGGTAAAG
GAAGAGATCCAGATATTGAAGGTAAAAGGTATAGAGATGCTGGTTGGAATGCTTATTTGAAAAAGAC
TGGCCAATTATAATCAGTACTGACAATAAAAAGATTCTTGTTTTCAAGAACTTGTCATTTGTATAGTTTT
TTTATATTGTAGTTGTTCTATTTTAATCAAATGTTAGCGTGATTTATATTTTTTTTCGCCTCGACATCATC
TGCCCAGATGCGAAGTTAAGTGCGCAGAAAGTAATATCATGCGTCAATCGTATGTGAATGCTGGTCGC
TATACTGCTGTCGATTCGATACTAACGCCGCCGGATTAGAAGCCGCCGAGCGGGTGACAGCCCTCCGA
AGGAAGACTCTCCTCCGTGCGTCCTCGTCTTCACCGGTCGCGTTCCTGAAACGCAGATGTGCCTCGCGC
CGCACTGCTCCGAACAATAAAGATTCTACAATACTAGCTTTTATGGTTATGAAGAGGAAAAATTGGCA
GTAACCTGGCCCCACAAACCTTCAAATGAACGAATCAAATTAACAACCATAGGATGATAATGCGATTA
GTTTTTTAGCCTTATTTCTGGGGTAATTAATCAGCGAAGCGATGATTTTTGATCTATTAACAGATATATA
AATGCAAAAACTGCATAACCACTTTAACTAATACTTTCAACATTTTCGGTTTGTATTACTTCTTATTCAA
ATGTAATAAAAGTATCAACAAAAAATTGTTAATATACCTCTATACTTTAACGTCAAGGAGATGGACAA
AGACTGCGAAATGAAGCGCACCACCCTGGATAGCCCTCTGGGCAAGCTGGAACTGTCTGGGTGCGAA
CAGGGCCTGCACCGTATCATCTTCCTGGGCAAAGGAACATCTGCCGCCGACGCCGTGGAAGTGCCTGC
CCCAGCCGCCGTGCTGGGCGGACCAGAGCCACTGATCCAGGCCACCGCCTGGCTCAACGCCTACTTTC
ACCAGCCTGAGGCCATCGAGGAGTTCCCTGTGCCAGCCCTGCACCACCCAGTGTTCCAGCAGGAGAGC
TTTACCCGCCAGGTGCTGTGGAAACTGCTGAAAGTGGTGAAGTTCGGAGAGGTCATCAGCGAGAGCC
ACCTGGCCGCCCTGGTGGGCAATCCCGCCGCCACCGCCGCCGTGAACACCGCCCTGGACGGAAATCCC
GTGCCCATTCTGATCCCCTGCCACCGGGTGGTGCAGGGCGACAGCGACGTGGGGCCCTACCTGGGCG
GGCTCGCCGTGAAAGAGTGGCTGCTGGCCCACGAGGGCCACAGACTGGGCAAGCCTGGGCTGGGTG
GTTCAGGTGGATCTGGTTCT**

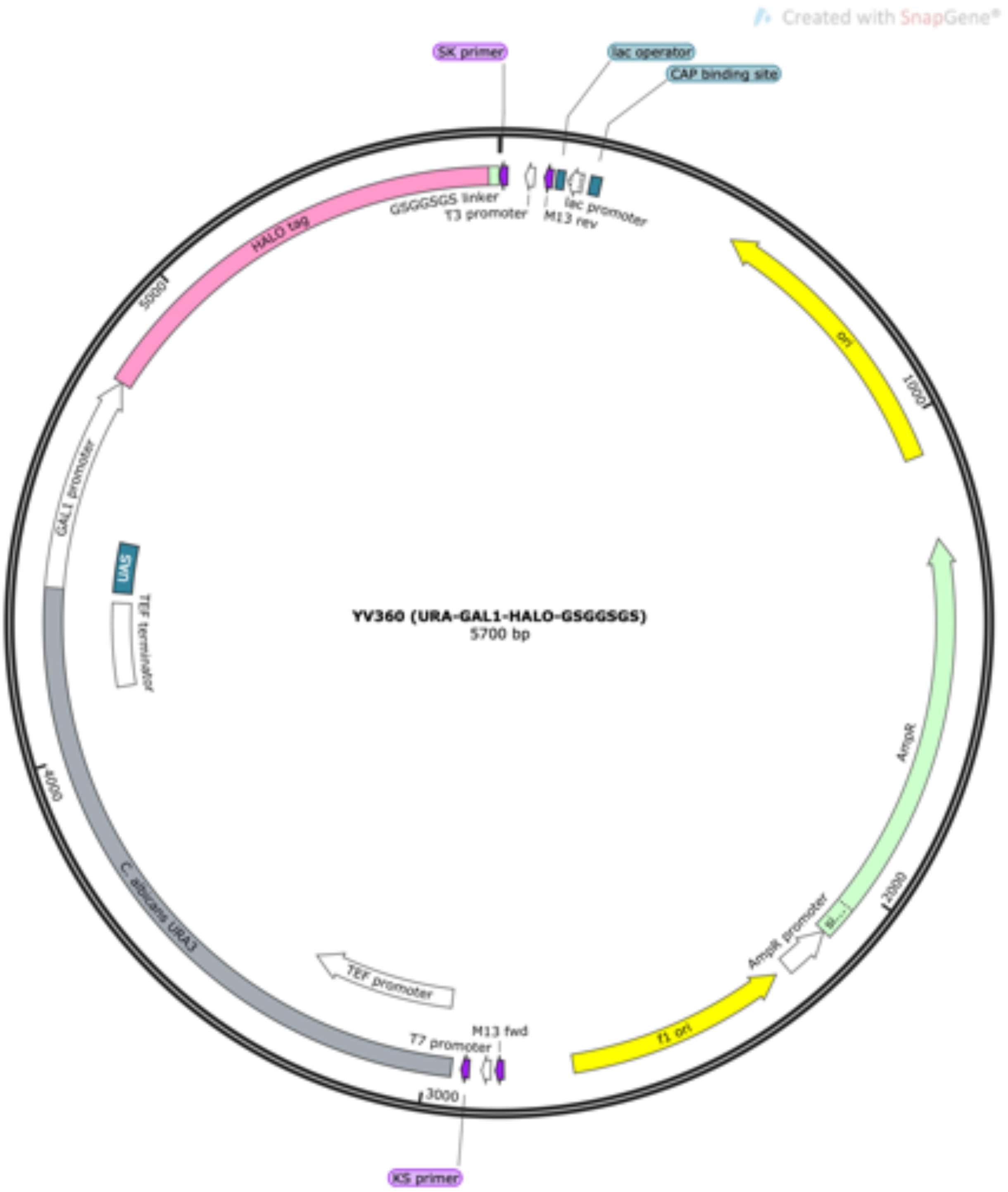


**YV360: pBS-SKII-URA-GAL1-HALO-GS**

**gatccactagttctagagcggccgccaccgcggtggagctccagcttttgttccctttagtgagggttaattgcgcgcttggcgtaatcat**
**ggtcatagctgtttcctgtgtgaaattgttatccgctcacaattccacacaacatacgagccggaagcataaagtgtaaagcctggggt**

gcctaatgagtgagctaactcacattaattgcgttgcgctcactgcccgctttccagtcgggaaacctgtcgtgccagctgcattaatga
atcggccaacgcgcggggagaggcggtttgcgtattgggcgctcttccgcttcctcgctcactgactcgctgcgctcggtcgttcggctgc
ggcgagcggtatcagctcactcaaaggcggtaatacggttatccacagaatcaggggataacgcaggaaagaacatgtgagcaaaa
ggccagcaaaaggccaggaaccgtaaaaaggccgcgttgctggcgtttttccataggctccgcccccctgacgagcatcacaaaaatc
gacgctcaagtcagaggtggcgaaacccgacaggactataaagataccaggcgtttccccctggaagctccctcgtgcgctctcctgtt
ccgaccctgccgcttaccggatacctgtccgcctttctcccttcgggaagcgtggcgctttctcatagctcacgctgtaggtatctcagttc
ggtgtaggtcgttcgctccaagctgggctgtgtgcacgaaccccccgttcagcccgaccgctgcgccttatccggtaactatcgtcttgag
tccaacccggtaagacacgacttatcgccactggcagcagccactggtaacaggattagcagagcgaggtatgtaggcggtgctacag
agttcttgaagtggtggcctaactacggctacactagaaggacagtatttggtatctgcgctctgctgaagccagttaccttcggaaaa
agagttggtagctcttgatccggcaaacaaaccaccgctggtagcggtggtttttttgtttgcaagcagcagattacgcgcagaaaaaa
aggatctcaagaagatcctttgatcttttctacggggtctgacgctcagtggaacgaaaactcacgttaagggattttggtcatgagatt
atcaaaaaggatcttcacctagatccttttaaattaaaaatgaagttttaaatcaatctaaagtatatatgagtaaacttggtctgaca
gttaccaatgcttaatcagtgaggcacctatctcagcgatctgtctatttcgttcatccatagttgcctgactccccgtcgtgtagataact
acgatacgggagggcttaccatctggccccagtgctgcaatgataccgcgagacccacgctcaccggctccagatttatcagcaataa
accagccagccggaagggccgagcgcagaagtggtcctgcaactttatccgcctccatccagtctattaattgttgccgggaagctaga
gtaagtagttcgccagttaatagtttgcgcaacgttgttgccattgctacaggcatcgtggtgtcacgctcgtcgtttggtatggcttcatt
cagctccggttcccaacgatcaaggcgagttacatgatcccccatgttgtgcaaaaaagcggttagctccttcggtcctccgatcgttgt
cagaagtaagttggccgcagtgttatcactcatggttatggcagcactgcataattctcttactgtcatgccatccgtaagatgcttttct
gtgactggtgagtactcaaccaagtcattctgagaatagtgtatgcggcgaccgagttgctcttgcccggcgtcaatacgggataatac
cgcgccacatagcagaactttaaaagtgctcatcattggaaaacgttcttcggggcgaaaactctcaaggatcttaccgctgttgagat
ccagttcgatgtaacccactcgtgcacccaactgatcttcagcatcttttactttcaccagcgtttctgggtgagcaaaaacaggaaggc
aaaatgccgcaaaaaagggaataagggcgacacggaaatgttgaatactcatactcttcctttttcaatattattgaagcatttatcag
ggttattgtctcatgagcggatacatatttgaatgtatttagaaaaataaacaaataggggttccgcgcacatttccccgaaaagtgcc
acctaaattgtaagcgttaatattttgttaaaattcgcgttaaatttttgttaaatcagctcattttttaaccaataggccgaaatcggca
aaatcccttataaatcaaaagaatagaccgagatagggttgagtgttgttccagtttggaacaagagtccactattaaagaacgtgga
ctccaacgtcaaagggcgaaaaaccgtctatcagggcgatggcccactacgtgaaccatcaccctaatcaagttttttggggtcgaggt
gccgtaaagcactaaatcggaaccctaaagggagcccccgatttagagcttgacggggaaagccggcgaacgtggcgagaaaggaa
gggaagaaagcgaaaggagcgggcgctagggcgctggcaagtgtagcggtcacgctgcgcgtaaccaccacacccgccgcgcttaat
gcgccgctacagggcgcgtcccattcgccattcaggctgcgcaactgttgggaagggcgatcggtgcgggcctcttcgctattacgcca
gctggcgaaagggggatgtgctgcaaggcgattaagttgggtaacgccagggttttcccagtcacgacgttgtaaaacgacggccagt
gagcgcgcgtaatacgactcactatagggcgaattgggtaccgggccccccctcgaggtcgacggtatcgataagcttgatatcgaatt
GGTCACCCGGCCAGCGACATGGAGGCCCAGAATACCCTCCTTGACAGTCTTGACGTGCGCAGCTCAGG
GGCATGATGTGACTGTCGCCCGTACATTTAGCCCATACATCCCCATGTATAATCATTTGCATCCATACAT
TTTGATGGCCGCACGGCGCGAAGCAAAAATTACGGCTCCTCGCTGCAGACCTGCGAGCAGGGAAACG
CTCCCCTCACAGACGCGTTGAATTGTCCCCACGCCGCGCCCCTGTAGAGAAATATAAAAGGTTAGGAT
TTGCCACTGAGGTTCTTCTTTCATATACTTCCTTTTAAAATCTTGCTAGGATACAGTTCTCACATCACATC
CGAACATAAACAACCATGACAGTCAACACTAAGACCTATAGTGAGAGAGCAGAAACTCATGCCTCACC
AGTAGCACAACGATTATTTCGATTAATGGAACTGAAGAAAACCAATTTATGTGCATCAATTGATGTTG
ATACCACTAAGGAATTCCTTGAATTAATTGATAAATTGGGTCCTTATGTATGCTTAATCAAGACTCATA
TTGATATAATCAATGATTTTTCCTATGAATCCACTATTGAACCATTATTAGAACTTTCACGTAAACATCA
ATTTATGATTTTTGAAGATAGAAAATTTGCTGATATTGGTAATACCGTGAAGAAACAATATATTGGTG
GAGTTTATAAAATTAGTAGTTGGGCAGATATTACTAATGCTCATGGTGTCACTGGGAATGGAGTAGTT
GAAGGATTAAAACAGGGAGCTAAAGAAACCACCACCAACCAAGAGCCAAGAGGGTTATTGATGTTA
GCTGAATTATCATCAGTGGGATCATTAGCATATGGAGAATATTCTCAAAAAACTGTTGAAATTGCTAA

**ATCCGATAAGGAATTTGTTATTGGATTTATTGCCCAACGTGATATGGGTGGACAAGAAGAAGGATTTG**
**ATTGGCTTATTATGACACCTGGAGTTGGATTAGATGATAAAGGTGATGGATTAGGACAACAATATAG**
**AACTGTTGATGAAGTTGTTAGCACTGGAACTGATATTATCATTGTTGGTAGAGGATTGTTTGGTAAAG**
**GAAGAGATCCAGATATTGAAGGTAAAAGGTATAGAGATGCTGGTTGGAATGCTTATTTGAAAAAGAC**
**TGGCCAATTATAATCAGTACTGACAATAAAAAGATTCTTGTTTTCAAGAACTTGTCATTTGTATAGTTTT**
**TTTATATTGTAGTTGTTCTATTTTAATCAAATGTTAGCGTGATTTATATTTTTTTTCGCCTCGACATCATC**
**TGCCCAGATGCGAAGTTAAGTGCGCAGAAAGTAATATCATGCGTCAATCGTATGTGAATGCTGGTCGC**
**TATACTGCTGTCGATTCGATACTAACGCCGCCGGATTAGAAGCCGCCGAGCGGGTGACAGCCCTCCGA**
**AGGAAGACTCTCCTCCGTGCGTCCTCGTCTTCACCGGTCGCGTTCCTGAAACGCAGATGTGCCTCGCGC**
**CGCACTGCTCCGAACAATAAAGATTCTACAATACTAGCTTTTATGGTTATGAAGAGGAAAAATTGGCA**
**GTAACCTGGCCCCACAAACCTTCAAATGAACGAATCAAATTAACAACCATAGGATGATAATGCGATTA**
**GTTTTTTAGCCTTATTTCTGGGGTAATTAATCAGCGAAGCGATGATTTTTGATCTATTAACAGATATATA**
**AATGCAAAAACTGCATAACCACTTTAACTAATACTTTCAACATTTTCGGTTTGTATTACTTCTTATTCAA**
**ATGTAATAAAAGTATCAACAAAAAATTGTTAATATACCTCTATACTTTAACGTCAAGGAGATGGCAGA**
**AATCGGTACTGGCTTTCCATTCGACCCGCATTATGTGGAAGTCCTGGGCGAGAGAATGCATTACGTTG**
**ACGTGGGTCCGAGAGATGGAACTCCGGTCCTTTTTCTGCACGGGAATCCTACAAGCTCTTATGTTTGGC**
**GCAATATCATCCCTCATGTAGCTCCGACGCATCGCTGTATTGCGCCGGACCTGATTGGTATGGGAAAAT**
**CTGATAAACCAGACCTGGGTTACTTTTTCGATGATCATGTGCGTTTCATGGATGCCTTCATTGAGGCAT**
**TAGGGCTTGAAGAAGTCGTCTTGGTGATTCATGATTGGGGCTCAGCTCTGGGATTTCACTGGGCTAAA**
**AGAAATCCTGAACGCGTAAAAGGCATCGCGTTTATGGAGTTCATTCGTCCAATTCCGACTTGGGATGA**
**ATGGCCTGAGTTCGCGAGAGAAACATTTCAAGCATTTCGCACGACCGATGTAGGCCGGAAGTTAATCA**
**TCGATCAGAATGTCTTTATCGAAGGGACATTGCCGATGGGAGTCGTTCGTCCGTTAACAGAAGTCGAA**
**ATGGATCACTATAGAGAACCTTTTCTTAATCCTGTGGACAGAGAGCCGCTGTGGCGGTTTCCGAACGA**
**ACTGCCGATTGCAGGCGAGCCTGCTAACATTGTAGCGCTGGTTGAAGAGTATATGGATTGGCTTCATC**
**AGTCTCCAGTTCCGAAGTTATTGTTTTGGGGTACGCCTGGCGTGCTTATTCCACCGGCCGAAGCGGCAC**
**GTTTGGCAAAAAGCCTGCCAAATTGCAAAGCCGTTGACATTGGCCCTGGACTTAACTTGCTTCAAGAG**
**GATAACCCGGACTTAATCGGGAGCGAAATTGCCCGGTGGCTTTCTACCTTAGAAATCAGCGGCGGTTC**
**AGGTGGATCTGGTTCT**
