# Supplementary material for: A set of Saccharomyces cerevisiae integration vectors for fluorescent dye labeling of proteins
Source: G3 (Bethesda). 2022 Aug 9;12(10):jkac201. doi: 10.1093/g3journal/jkac201 (PMC9526040; doi:10.1093/g3journal/jkac201)
Supplement: jkac201_Supplemental_Material_Legends [file jkac201_supplemental_material_legends.docx]

**Supplementary Tables (attached Excel file).**

Table S1: Oligonucleotides used in this study

Table S2: Yeast strains used in this study

Table S3: Plasmids created in this study

**Supplemental Data:** Maps and sequences of tagging plasmids
